# Integrated multi-omics profiling identifies genetic loci of African swine fever resistance in pigs

**DOI:** 10.1093/gigascience/giag066

**Published:** 2026-05-30

**Authors:** Xiaowei Ye, Qinqin Xie, Caiyun Cao, Shuang Liu, Wenbo Sun, Zhe Zhang, Qishan Wang, Yuchun Pan, Zhen Wang

**Affiliations:** Zhejiang Key Laboratory of Nutrition and Breeding for High-Quality Animal Products, College of Animal Sciences, Zhejiang University, Hangzhou, Zhejiang 310058, China; Zhejiang Key Laboratory of Nutrition and Breeding for High-Quality Animal Products, College of Animal Sciences, Zhejiang University, Hangzhou, Zhejiang 310058, China; Zhejiang Key Laboratory of Nutrition and Breeding for High-Quality Animal Products, College of Animal Sciences, Zhejiang University, Hangzhou, Zhejiang 310058, China; Zhejiang Key Laboratory of Nutrition and Breeding for High-Quality Animal Products, College of Animal Sciences, Zhejiang University, Hangzhou, Zhejiang 310058, China; Shandong Key Laboratory of Animal Disease Control and Breeding, Institute of Animal Science and Veterinary Medicine, Shandong Academy of Agricultural Sciences, Jinan, Shandong 250100, China; Zhejiang Key Laboratory of Nutrition and Breeding for High-Quality Animal Products, College of Animal Sciences, Zhejiang University, Hangzhou, Zhejiang 310058, China; Hainan Institute, Zhejiang University, Yongyou Industrial Park, Yazhou Bay Sci-Tech City, Sanya 572000, China; Zhejiang Key Laboratory of Nutrition and Breeding for High-Quality Animal Products, College of Animal Sciences, Zhejiang University, Hangzhou, Zhejiang 310058, China; Hainan Institute, Zhejiang University, Yongyou Industrial Park, Yazhou Bay Sci-Tech City, Sanya 572000, China; Zhejiang Key Laboratory of Nutrition and Breeding for High-Quality Animal Products, College of Animal Sciences, Zhejiang University, Hangzhou, Zhejiang 310058, China; Hainan Institute, Zhejiang University, Yongyou Industrial Park, Yazhou Bay Sci-Tech City, Sanya 572000, China; Zhejiang Key Laboratory of Nutrition and Breeding for High-Quality Animal Products, College of Animal Sciences, Zhejiang University, Hangzhou, Zhejiang 310058, China; Hainan Institute, Zhejiang University, Yongyou Industrial Park, Yazhou Bay Sci-Tech City, Sanya 572000, China

**Keywords:** African swine fever, multi-omics, disease resistance, pig breeding, host genetics

## Abstract

**Background:**

African swine fever (ASF) remains a persistent threat to global pig production, with no licensed vaccines or effective treatments available. Observations of surviving individuals within low-virulence infected herds suggest that host genetic resistance plays a crucial role.

**Results:**

Here, we present a multi-dimensional integrative analysis to uncover host genomic variants associated with ASF resistance. Combining genome-wide association studies (GWAS), genetic differentiation, and functional genomic approaches, including TWAS, SMR, colocalization, and Bayesian network GWAS, we prioritized 135 high-priority candidate resistance genes from an initial gene set of 1,102 candidates. These prioritized genes are enriched in immune-related pathways, such as chemokine signaling and IL-15-mediated activation. Heritability enrichment and transcriptomic analyses further revealed tissue- and cell-type-specific expression patterns, particularly in peripheral immune organs and pulmonary alveolar macrophages. Dynamic infection-responsive genes, including *CXCL10, CXCL11*, and *IL15*, exhibited robust antiviral signatures, which highlighted Mac_CD163 as key cellular mediators in the immune response to ASF. Moreover, multiple genes (such as *SOS1, FCGR2B, FCGR3*) converged on the PI3K-AKT and Fcγ receptor signaling axes pathways, underscoring their functional importance. Finally, we developed a polygenic resistance score using 40 prioritized independent SNPs, which effectively discriminates phenotypic outcomes and showed a positive correlation with health traits such as platelet distribution width.

**Conclusions:**

These findings provided a genomic foundation for the precision breeding of ASF-resistant pigs and inform host-targeted disease control strategies.

## Introduction

African swine fever (ASF) is a highly contagious and often lethal viral disease that affects both domestic and wild pigs. Caused by the African swine fever virus (ASFV), the disease is characterized by severe hemorrhagic fever, with case fatality rates reaching nearly 100% in acute infections and 30%∼70% in subacute or chronic forms [1]. ASF outbreaks have caused devastating losses in swine populations worldwide, posing a substantial threat to global food security. Between 2005 and January 2025, ASF outbreaks were reported in 83 countries [2]. From 2014 to 2017, nearly 8 million pigs in Eastern Europe and the Russian Federation were lost due to ASF [3]. By 2019, the disease had resulted in the culling or death of nearly 5 million pigs in Asia [4]. The economic repercussions of ASF have been staggering. Russia reported losses of 267 million USD during the 2011 outbreaks [5]. In 2022, ASF-related disruptions cost France’s export market an estimated 168∼389 million USD [6]. For 2023, projections suggested potential losses of 2,500 million USD in Australia [7] and, in the event of an outbreak, economic modeling estimates potential losses of up to 50,000 million USD in the USA, assuming the disease persists for 10 years and leads to a prolonged suspension of exports [8].

The emergence of ASF in China in 2018 had particularly profound effects on the national pig industry, given China’s large-scale swine production [9, 10]. With nearly half of the global pig population located in China [3, 9, 11], the outbreak led to dramatic reductions in herd sizes, severe disruptions in pork supply chains, loss of valuable genetic resources, and sharp increases in pork prices [11]. Within a single year (August 2018–July 2019), outbreaks of ASF in China resulted in economic losses exceeding 100,000 million USD [12].

In response, considerable research efforts have focused on understanding ASFV biology, modes of transmission, and the development of effective vaccines and therapeutics [13–15]. However, ASF continues to be the most critical threat to the global pig industry. The absence of commercially licensed vaccines, the virus’s ability to persist in diverse environmental reservoirs, and the high genetic variability across ASFV strains greatly complicate control efforts [16–19].

Intriguingly, field observations from recent outbreaks have revealed variable clinical outcomes among pigs within the same herd. While many individuals succumb to infection, some individuals survive and exhibit seroconversion without detectable viremia: testing negative for ASFV antigens but positive for ASFV-specific antibodies. Such findings suggest the existence of a potential natural resistance genetic basis, drawing attention to host genetic factors as critical determinants of ASF susceptibility, which likely modulates immune activation, coordination, and regulation to limit immunopathology and promote survival or infection tolerance [20]. In this study, we define “resistance” broadly as the host’s capacity to survive infection, encompassing both the ability to limit viral replication and to mitigate disease severity. Host immune responses play a central role in shaping the heterogeneous clinical outcomes of ASFV infection, reflecting complex virus–host interactions. For example, asymptomatic pigs exhibit higher NK cell activity but lower IgA and virus-specific antibody levels compared with susceptible individuals [21]. Functional studies further demonstrate that innate antiviral effectors, such as MxA and IFITM proteins, can directly inhibit ASFV replication [22, 23]. This shift in focus from a pathogen-centered to host-centered perspective presents new possibilities for ASF control strategies. Genetic resistance traits, in particular, offer a sustainable and long-term strategy for ASF management, especially given the current lack of effective vaccines or antiviral treatments. Moreover, identifying and leveraging these traits could aid in the preservation of indigenous pig breeds, many of which are renowned for their natural resistance to disease.

Current research on ASF host resistance has largely focused on identifying candidate genes, particularly through interspecies comparisons. The contrasting responses between warthogs and domestic pigs represent a well-established model: despite similar viral replication levels, clinical outcomes differ markedly, highlighting a dominant role for host factors [24]. Genomic studies have revealed adaptive divergence in immune-related genes, including *Mx1, Mx2*, and *PTGS2*, implicating ASF as a selective pressure in warthog evolution [25]. Comparative and functional analyses have further identified candidate resistance genes such as *ISG15, HERC5, TRIM21*, and *RELA*, as well as structural variants affecting loci like *LDHB* and TRIM family genes that modulate viral replication [26, 27]. In domestic pigs, emerging evidence also supports a genetic basis for variation in ASF outcomes, with distinct disease progression observed across breeds such as Ugandan pigs and Xiang pigs [28, 29]. However, most studies rely on limited sample sizes or single-omics approaches, resulting in inconsistent findings and incomplete genetic insight. A comprehensive, large-scale, multi-omics framework is therefore required to systematically resolve the genetic architecture and molecular basis underlying host variation in ASF infection.

To systematically identify the genetic basis of these resistance traits, we conducted integrative genomic analyses combining whole-genome sequencing (WGS) of resistant and susceptible pigs, functional annotation of candidate variants, and transcriptomic profiling using publicly available RNA-seq datasets related to ASFV infection. The analyses leveraged resources from the FarmGTEx consortium, which provides a multi-species framework for transcriptomic and multi-omics profiling in livestock [30]. Within this initiative, PigGTEx [31] systematically characterizes gene expression across diverse tissues and developmental stages in pigs, while PigBiobank [32] extends these efforts by integrating large-scale phenotypic and omics data from multiple pig populations. Together, these datasets offer a comprehensive reference for investigating gene regulation and functional variation in pigs, thereby supporting the identification of genomic variants, biological pathways, and candidate genes underlying resistance to ASF. Unlike previous studies, which primarily focused on association signals, our study bridges variant discovery with functional analyses and predictive modeling, offering more biological insights of ASF resistance, establishing a genomic foundation for selective breeding, genomic prediction, and potentially host-directed ASF control strategies. These results have broad implications for improving swine health and enhancing herd resilience against future ASF outbreaks.

## Results

### ASF-resistance candidate genes

To characterize genomic differences underlying ASF infection outcomes, we analyzed 474 pigs with antigen/antibody phenotyping and WGS (Supplementary Table S1). In a herd naturally exposed to low-virulence ASFV, 108 individuals died, 222 survived, and 144 remained uninfected (Table 1). These divergent phenotypes, observed under consistent exposure conditions and supported by longitudinal sampling, indicate a substantial host genetic contribution to disease variability.

**Table 1:** Grouping of individuals in experimental pig herds.

Group	Antigen^a^	Antibody^b^	Situations	Meaning	Sample size
A	/	+	Dead	Antigen infection → immune response → production of antibodies → pigs die	108
B	-	+	Alive	Antigen infection → immune response → production of antibodies → antigen clearance.	222
C	-	-	Alive	1 Antigen infection → immune response → antibody → antigen clearance → antibody inactivation	144
				2 Antigen uninfected → no immune response → no antibody	
D	/	/	/	The remaining control varieties in PHARP v2 (except experimental pigs)	1,730

a ASFV antigen test: detected via the RAA fluorescence method targeting the *KP177R* (p22 protein), − for negative and + for positive.

b ASFV antibody test: detected via indirect ELISA targeting structural proteins P32, P62, and P72, − for negative and + for positive.

For genomic analysis, the WGS dataset yielded 23,290,599 common variants after quality control (minor allele frequency [MAF] > 0.05, Table 1). The experimental cohort displays an admixed genetic background, with ancestry predominantly derived from East Asian (specifically East Chinese) pigs, forming a unique cluster distinct from European commercial breeds (Supplementary Fig. S1). Principal component analysis (PCA) within the experimental population showed minimal stratification by breed and extensive overlap among disease phenotypes, indicating weak population structure and no evident confounding by genetic background (Supplementary Fig. S1). Together, these results support the suitability of this cohort for genome-wide association studies (GWAS).

**Figure 1:**
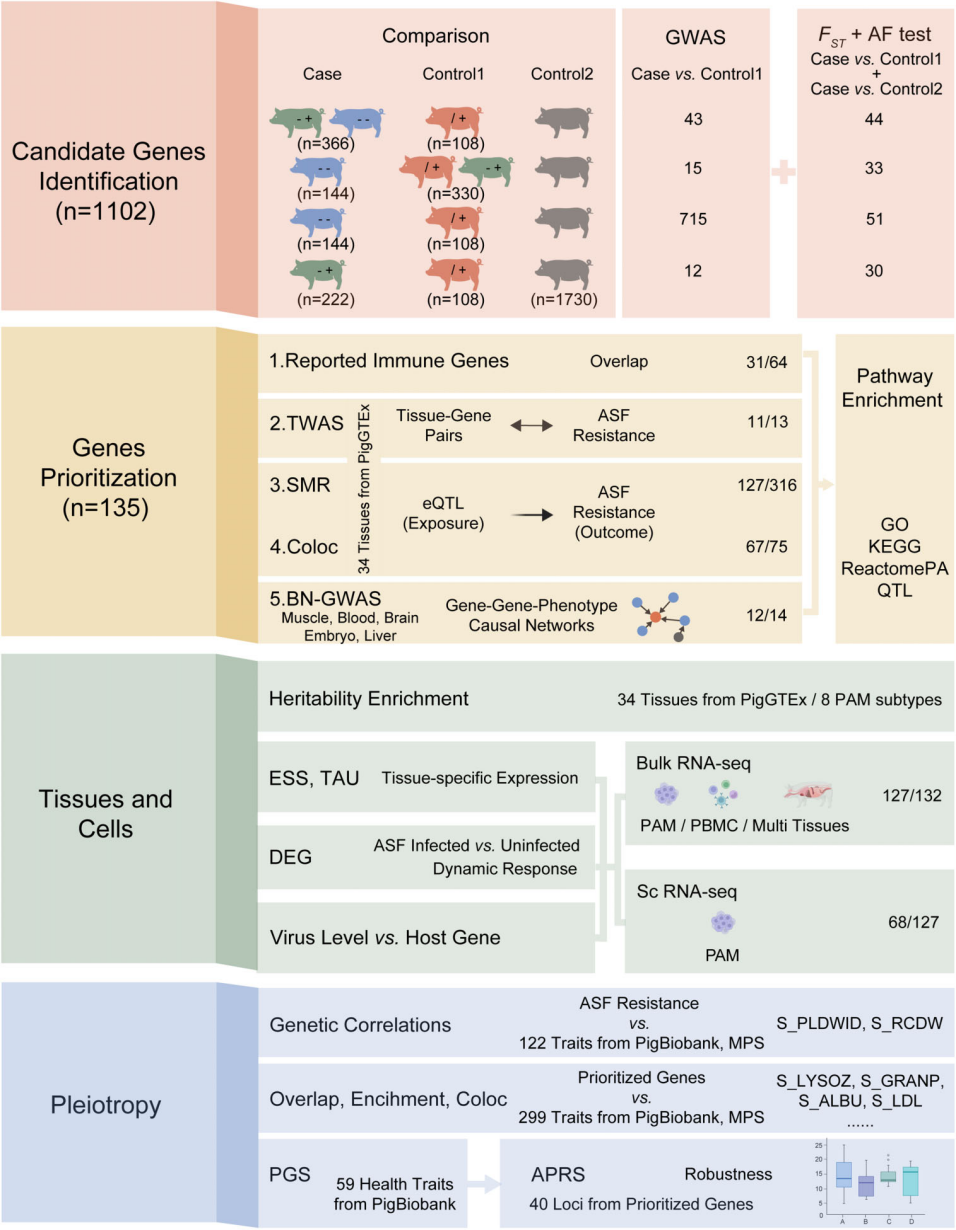
Study overview. A schematic representation of the study design and analytical framework: experimental grouping and comparative analyses (1st): summarizes experimental groupings, comparisons, and analyses, including GWAS, *F*_ST_, AF tests, and gene identification. Pig icons are color-coded by clinical phenotype: blue for uninfected (antigen−/antibody−), green for survived (antigen−/antibody+), and red for deceased (antigen+). Gray icons represent a background population used for *F*_ST_ and AF tests. The “+” sign indicates the integration of association signals from GWAS and population genetic differentiation. Gene prioritization (2nd): ASF resistance candidate genes were prioritized using 5 independent methods—overlap with reported immune genes, TWAS, SMR, Coloc, and BN-GWAS. These analyses identified 135 high-priority genes, which were further analyzed through pathway enrichment to elucidate their biological roles. Numerical ratios (e.g., 31/64) indicate the yield of each method: the numerator denotes prioritized genes, while the denominator represents total candidates processed by that analysis. The double-headed arrow (**⟷**) in TWAS denotes associations between gene expression and traits; the single-headed arrow (**→**) in SMR/Coloc indicates potential causal or regulatory effects from eQTL to phenotype. The network icon in BN-GWAS represents inferred gene–gene-phenotype causal relationships. Tissue and cell-specific analyses (3rd): Heritability enrichment pinpointed tissues and cell types (e.g., PAM) associated with ASF resistance. Bulk and single-cell transcriptomics data integration revealed basal tissue-specific expression patterns (ESS, TAU) and dynamic gene responses (DEG) post-infection. The meaning of numerical ratios is the same as in the yellow section. Polygenic associations (4th): Global genetic correlations between ASF resistance and 269 other pig traits were investigated. Shared genomic regions, trait enrichment, and cross-trait colocalization analyses highlighted the polygenic nature of prioritized genes. Associations between the APRS and PGS for 59 pig health traits were evaluated to assess the biological consistency and robustness of the genetic prediction. AF test: allele frequency chi-square test. GWAS: genome-wide association study. *F*_ST_: fixation index. TWAS: transcriptome-wide association study. SMR: summary-based Mendelian randomization. Coloc: colocalization. BN-GWAS: Bayesian network GWAS. PAM: porcine alveolar macrophage. ESS: expression specificity scores. TAU: tissue-specific gene expression. DEG: differentially expressed genes. PAM: porcine alveolar macrophage. PBMC: peripheral blood mononuclear cell. PGS: polygenic score. APRS: African swine fever resistance prediction score.

**Figure 2:**
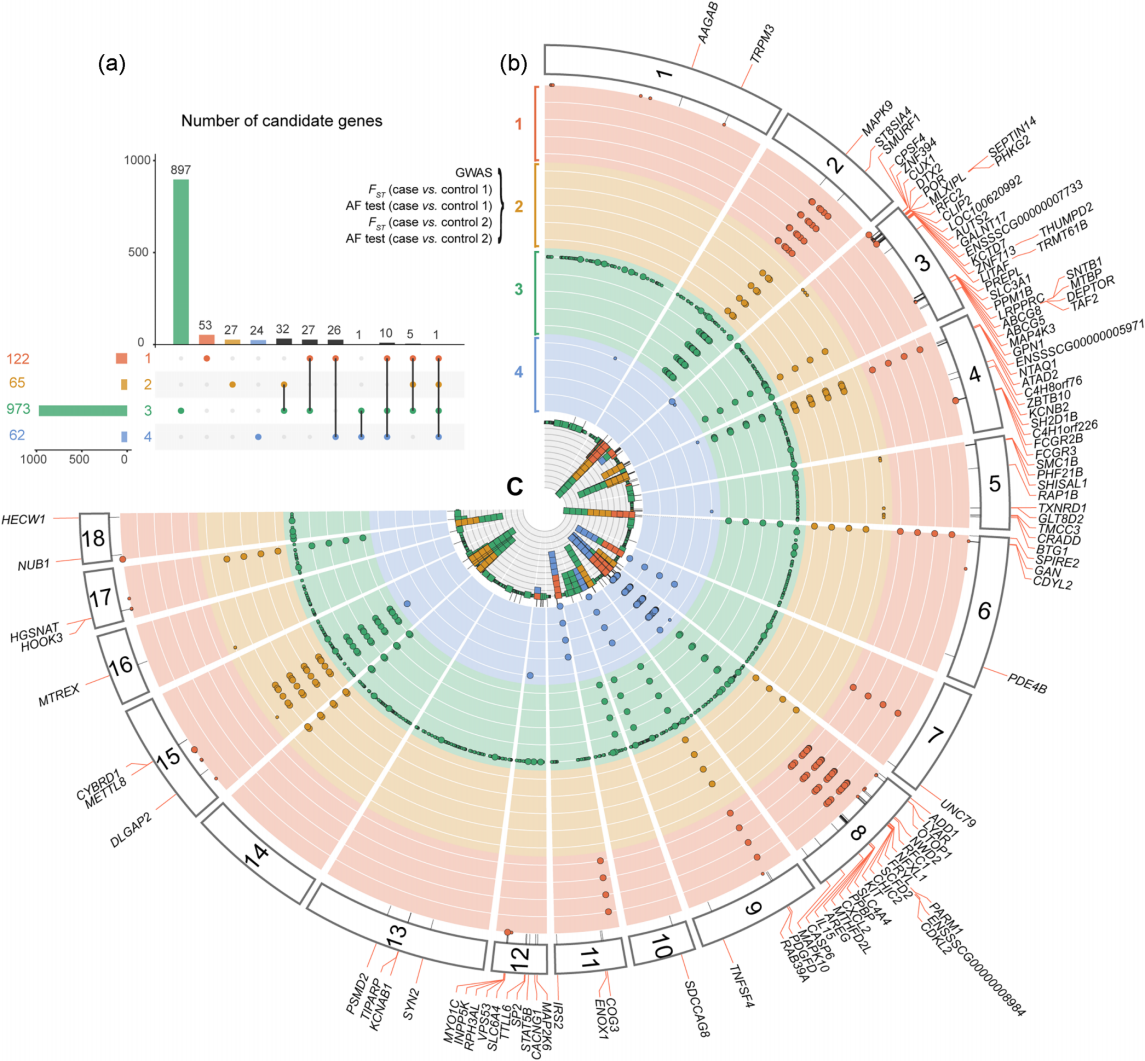
Integrative identification and genomic landscape of candidate genes associated with ASF resistance. (A) Overlap of candidate genes across analytical comparisons. The Upset plot illustrates the intersection of genes identified in 4 clinical comparisons (1–4). The vertical bars indicate the number of genes unique to or shared between comparisons, as indicated by the connected dots below. (B) Fuji plot showing the genomic distribution of identified loci. The outermost circle displays chromosomes with annotated prioritized genes. In the middle layers, each dot represents a significant locus associated with a specific comparison (1–4). Within each categorical track, the concentric rings (from outer to inner) represent 5 distinct statistical methods: GWAS, *F*_ST_ (case vs. control 1), AF test (case vs. control 1), *F*_ST_ (case vs. control 2), and AF test (case vs. control 2). (C) Methodological support for candidate loci. The inner stacked bar plot summarizes the cumulative number of statistical methods (out of the 5 mentioned above) supporting the candidate loci within each genomic region. The height and color composition of each bar directly correspond to the loci displayed in part (b). Comparison 1: (uninfected + survived) vs. dead (general resistance/protection); Comparison 2: uninfected vs. (survived + dead) (pathogen recognition/avoidance); Comparison 3: uninfected vs. dead (strict protection); Comparison 4: survived vs. dead (adaptive immunity/recovery). GWAS: genome-wide association study; *F*_ST_: fixation index; AF test: allele frequency chi-square test.

To maximize the identification of candidate loci associated with ASF resistance, individuals were stratified into 4 groups for comparative analyses, each based on different potential gene-related objectives (Fig. 1, Tables 2 and 3). GWAS and genetic differentiation analyses (fixation index, *F*_ST_), combined with allele frequency (AF) tests (hereinafter referred to as the *F*_ST_ method), were employed to identify loci that may have undergone and contribute to ASF resistance. Ultimately, 1,102 non-redundant genes were identified as significantly associated with ASF resistance (GWAS: *P*-values < $\frac{1}{{Me}}$; *F*_ST_: *P*-values < $\frac{{0.05}}{N}$) (Fig. 2, Supplementary Fig. S2, Supplementary Tables S2 and S3).

**Table 2:** Grouping of experimental comparisons between groups.

		Control			
Comparison	Case	1	2	Phenotype	Potential meaning
1	B+C	A	D	(Uninfected + survived infection) vs. (dead)	General resistance, protection
2	C	A+B	D	(Uninfected) vs. (survived infection + dead)	Pathogen recognition, avoidance
3	C	A	D	(Uninfected) vs. (dead)	Strict protection
4	B	A	D	(Survived infection) vs. (dead)	Adaptive immunity, recovery

**Table 3:** Statistical overview of candidate loci associated with African swine fever.

			Candidate loci		Genes	
Comparison ^a^	*N* ^b^	Me^c^	GWAS^d^	*F*_ST_^e^	GWAS	*F*_ST_
1	23,290,599	2,581,021	719	3,490	43	44
2	23,290,599	2,581,021	56	5,163	15	33
3	23,160,238	2,537,788	2,445	9,453	715	51
4	23,403,868	2,571,190	72	1,848	12	30

a Comparisons 1–4 are defined as follows based on clinical outcomes: 1, (uninfected + survived) vs. dead; 2, uninfected vs. (survived + dead); 3, uninfected vs. dead; 4, survived vs. dead. Detailed groupings are provided in Table 2.

b The number of all loci.

c The number of loci after LD pruning.

d The number of loci identified by GWAS method.

e The number of loci identified by *F*_ST_ method.

### ASF-resistance gene prioritization

**Figure 3:**
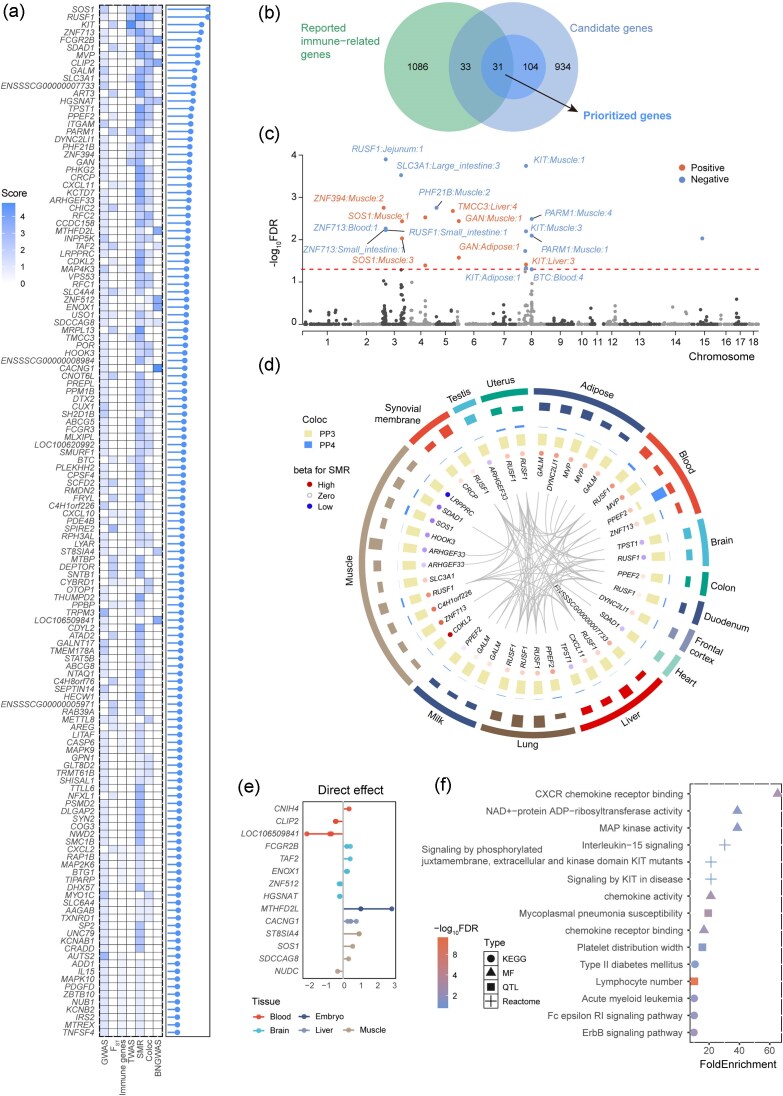
Gene prioritization and functional analyses. (a) Overview of the gene prioritization framework for ASF resistance. Genes are ranked by their aggregate prioritization scores (from high to low). The heatmap displays individual scores across various genomic methods (GWAS, *F*_ST_, reported immune genes, TWAS, SMR, colocalization, BN-GWAS), with color intensity representing score values. The lollipop plot illustrates the aggregate prioritization score, calculated by summing individual scores. Genes were identified as prioritized candidates if they achieved an aggregate score ≥3.5 or were supported by 2 or more independent methods. (b) Venn diagram illustrating the overlap among 1,102 candidate genes, 135 prioritized genes, and 1,150 previously reported immune-related genes. (c) Manhattan plot of 13 candidate genes identified by transcriptome-wide association study (TWAS) (FDR < 0.05, red dashed line), with 11 categorized as prioritized genes. The *y*-axis shows −log_10_FDR, the *x*-axis represents genomic locations, and dot colors indicate correlation direction. (d) 42 causal pairs supported by colocalization (coloc) and summary-based Mendelian randomization (SMR) analyses, demonstrating a causal relationship between gene expression in tissues and ASF resistance. Outer to inner rings represent tissue sectors, −log10 *P*-value from SMR, PP4 from coloc, PP3 from coloc, SMR beta value (red for positive, blue for negative), and a cross-tissue gene network. PP4: posterior probability of hypothesis 4; PP3: posterior probability of hypothesis 3. (e) Inference of 14 candidate genes by Bayesian network GWAS (BN-GWAS), with 12 identified as prioritized genes. The *x*-axis shows the effect size. (f) Bubble plot showing significant enrichment of 135 prioritized genes. The *x*-axis represents enrichment fold, bubble color denotes −log_10_FDR, and bubble shape reflects database categories.

To prioritize genes associated with ASF resistance, we assessed 1,102 candidate genes by integrating 5 independent lines of evidence to assign prioritization scores: reported immune genes (Supplementary Table S5), transcriptome-wide association studies (TWAS), summary-data-based Mendelian randomization (SMR), colocalization, and Bayesian network genome-wide association studies (BN-GWAS) (see the “Methods” section, Table 4). As a result, 135 high-priority candidate genes (hereafter referred to as “prioritized genes”) were identified based on prioritization scores in the top 10% (≥3.5) or inference by at least 2 statistical methods (Fig. 3a and Supplementary Table S4). Among these prioritized genes, 31 were previously implicated in immune functions, such as members of the chemokine family (*CXCL2, CXCL7, CXCL10, CXCL11*) [33] and the MAP kinase family (*MAP4K3, MAPK9, MAP2K6, MAPK10*) [34], which are integral to immune regulation, inflammatory responses, and cell signaling. Additionally, *IL15* was highlighted for its roles in T and NK cell activation and maintenance of memory CD8⁺ T cells [35], emphasizing its potential involvement in ASF resistance.

**Table 4:** Scoring framework for ASF resistance gene prioritization.

Method	Repeat count	Score range	Scoring formula
GWAS	1 per group	0~3	Count * 1
*F*_ST_ + AF test	1 per group	0~3	Count * 1
Previously reported immune genes	/	0~1	Count * 0.5
TWAS	1 per group, per tissue	0~4	Count * 1.2
SMR	1 per group, per tissue	0~43	log_2_(count +1) * 0.8
Colocalization	1 per group, per tissue	0~25	log_2_(count +1) * 0.8
Bayesian network GWAS	1 per group, per tissue	0~4	Count * 1.2
Independent inference	/	0~3	2 methods supported: +1; 3~4 methods supported: +2; ≥5 methods supported: +3

#### Tissue-specific associations via TWAS

To prioritize candidate genes based on the aggregate association between predicted expression levels and ASF resistance, we first performed TWAS across 34 tissues [31] (Supplementary Table S6). This analysis revealed that 11 of the 135 prioritized genes exhibited significant tissue-specific associations (FDR < 0.05). Notably, *SOS1, GAN, ZNF394, KIT*, and *TMCC3* exhibited positive associations in liver and muscle tissues (Fig. 3c, Supplementary Fig. S3 and Supplementary Table S7). The liver is a primary site of viral replication, involving both resident Kupffer cells and hepatocytes [36], whereas signals in muscle likely reflect the systemic nature of infection, capturing the responses of secondary target cells, such as vascular endothelium [37], alongside the infiltration of primary target cells like monocytes and macrophages [38]. These findings align with the systemic pathology of ASFV, where genetic variation in both primary and secondary target tissues modulates overall host resistance. Furthermore, genes including *ZNF713, RUSF1*, and *KIT* exhibited negative associations in the blood and intestine (Fig. 3c, Supplementary Fig. S3 and Supplementary Table S7). Together, these tissue-dependent contexts provide high-confidence targets for functional prioritization and the development of ASF-resistant breeding programs.

#### Inference of putative regulatory genes via SMR and colocalization

In parallel, to identify high-confidence genes by testing the potential mediation effects of specific top eQTLs, we employed SMR and colocalization analysis. Using SMR analysis across 34 tissues from PigGTEx [31], we identified 1,490 candidate causal pairs involving 316 candidate genes, with 127 classified as prioritized (Supplementary Tables S6 and S8). Colocalization analysis further supported 42 of these pairs, suggesting the presence of shared potential causal variants associated with ASF resistance (Fig. 3d). For example, *PPEF2* expression in blood was found to share a potential causal variant with ASF resistance (posterior probabilities under Hypothesis 4, PP4 > 0.75) (Supplementary Fig. S4). Functionally, *PPEF2* inhibits ASK1, an MAP kinase involved in apoptosis regulation, and modulates CD8⁺ cDC1 antigen presentation [39, 40], implicating it in immune processes relevant to ASF resistance. Additionally, 41 pairs showed strong posterior probabilities under Hypothesis 3 (PP3 > 0.75), such as *SOS1* (muscle), *SLC3A1* (muscle), *RUSF1* (multiple tissues), *GALM* (milk, adipose), *CXCL11* (liver), and *MVP* (blood, adipose), supporting associations with distinct potential causal variants (Fig. 3d and Supplementary Table S9).

#### Regulatory network inference via BN-GWAS

BN-GWAS [41] was employed to infer causal networks linking candidate genes to ASF resistance. To ensure robust causal inference, we utilized RNA-seq data from 5 tissues (muscle, blood, brain, embryo, and liver) with sample sizes exceeding 300, as sufficient power is critical for stable expression imputation. These tissues represent both primary viral replication sites (liver and blood) and organs that reflect the systemic physiological disruptions caused by ASFV [42, 43]. We identified 12 prioritized genes with putative regulatory effects (Fig. 3e and Supplementary Table S10). Notable genes include *SOS1* (muscle), *FCGR2B* (brain), and *SDCCAG8* (muscle), which showed positive effects, and *HGSNAT* (brain), *LOC106509841* (blood), and *CLIP2* (blood), which exhibited negative effects.

#### Integration and cross-evaluation of prioritized genes

Among all prioritized genes, *SOS1* achieved the highest prioritization score (13.7), supported by all 5 lines of evidence. As a regulator of the MAPK and PI3K/JAK cellular signaling pathways and tumorigenesis [44, 45], *SOS1* was identified as having a positive effect on ASF resistance. Additionally, 13 other genes (*RUSF1, KIT, ZNF713, FCGR2B, SDAD1, MVP, CLIP2, SLC3A1, HGSNAT, PHF21B, ZNF394, CXCL11*, and *SDCCAG8*) were supported by 3 independent lines of evidence, such as combinations of TWAS, SMR, colocalization, highlighting the robustness of their prioritization (Fig. 3a and Supplementary Table S4). Notably, *SOS1* (muscle), *RUSF1* (blood, liver, lung), *SLC3A1* (muscle), *ZNF394* (liver), and *FCGR2B* (brain) showed consistent tissue-specific activity.

### Enriched pathways of prioritized genes

Pathway enrichment analysis of the prioritized genes revealed 41 immune-related pathways significantly associated with ASF resistance (FDR < 0.05) (Fig. 3f and Supplementary Table S11). Notably, the most enriched functional clusters, including CXCR chemokine receptor binding (FDR = 1.7 × 10^−3^), MAP kinase activity (FDR = 2.3 × 10^−2^), interleukin-15 signaling (FDR = 3.2 × 10^−2^), and lymphocyte number (FDR = 7.7 × 10^−15^), exhibit high concordance with the characterized pathological features of ASFV infection, particularly regarding the systemic inflammatory cascades and the profound lymphopenia that define acute ASF [46–48]. By pinpointing these pathways, our results underscore the critical role of maintaining immune homeostasis and cellular signaling integrity in mediating host resistance to ASFV.

### Resistance-associated tissues and cells

**Figure 4:**
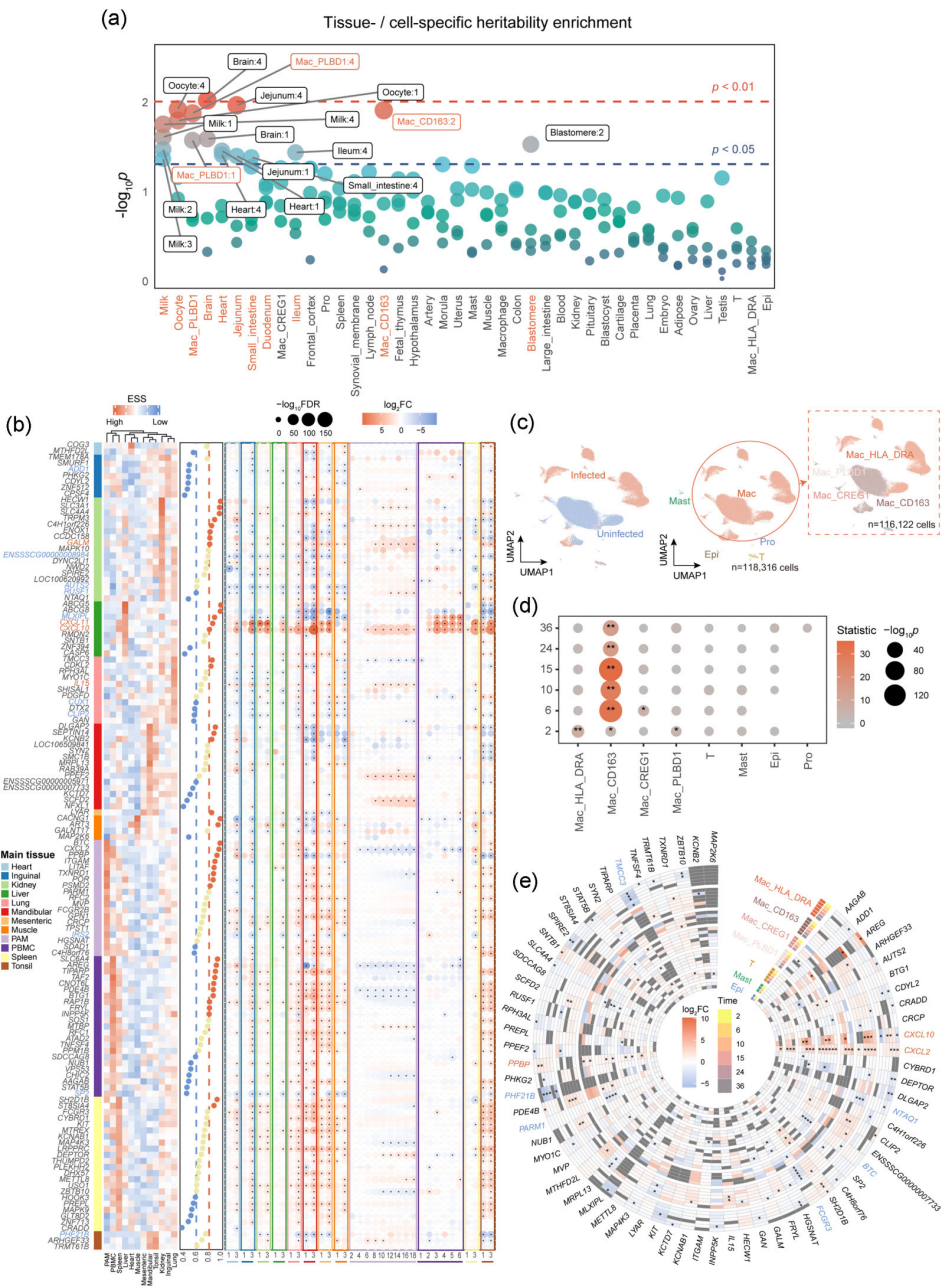
Tissue- and cell-specificity and gene expression analyses. (a) Manhattan plot depicting genetic enrichment significance for 4 comparisons across 34 tissues and 8 PAM cell types. The *y*-axis shows –log10*P*-value, and the *x*-axis represents tissues/cell types. Dashed lines indicate significance thresholds (*P*-value < 0.05 and *P*-value < 0.01, respectively). Significant tissues and cell types are highlighted and labeled. (b) Baseline expression and dynamic response of prioritized genes to ASF infection in bulk RNA data. The *y*-axis lists prioritized genes, while the *x*-axis, from left to right, displays primary tissue expression distribution (color-coded squares); expression specificity scores (ESS) values across tissues, with heatmap colors indicating magnitude; tissue-specific gene expression (TAU) values, where TAU > 0.8 denotes high tissue specificity, and TAU < 0.6 indicates widespread expression; differential expression of prioritized genes at various infection time points, with point size representing −log_10_FDR and color indicating log_2_FC. Time units are hours post-infection (hpi) for PAM and days post-infection (dpi) for other tissues. (c) UMAP visualization of cell types in PAM scRNA-seq data. (d) Module scoring of the prioritized gene subset in PAM scRNA-seq. The score represents the aggregate expression difference of 127 prioritized genes (a detectable subset of the 135 total prioritized genes) pre- and post-infection. Results are derived using a two-sided Welch *t*-test (*P-value < 0.001, **P-value < 1 × 10^−8^). The *y*-axis indicates infection time (hpi), the *x*-axis shows cell types, dot size reflects −log_10_*P*-value, and dot color represents statistical measures. (e) Differential expression of prioritized genes across PAM cell types. Square colors represent log_2_FC values, with significance marked as *P-value < 0.05 and |log_2_FC| > 1. Mac: macrophages. Mast: mast cells. T: T cells. Pro: proliferating cells. Epi: epithelial cells. Four macrophage subtypes: Mac_CD163, Mac_HLA_DRA, Mac_CREG1, and Mac_PLBD1.

To identify tissues and cells associated with ASF resistance, we conducted heritability enrichment analysis across 34 tissues and 8 porcine alveolar macrophage (PAM) subtypes using linkage disequilibrium (LD) score regression. Significant heritability enrichment was observed in PAM subtypes (Mac_CD163 and Mac_PLBD1) (Fig. 4a and Supplementary Table S12), consistent with the established role of PAMs as the primary targets of ASFV infection [49]. Interestingly, heritability enrichment was also detected in non-immune tissues, including the small intestine, brain, heart, and milk. This suggests that genetic contributions to ASF resistance may extend beyond primary immune sites, potentially reflecting a systemic, cross-tissue defensive architecture.

RNA-seq analyses from ASFV-infected and healthy pig tissues retained 132 prioritized genes after basal expression filtering (TPM > 0.1 in ≥20% of samples), revealing tissue-specific gene expression patterns, with 41 genes (31.1%) exhibiting high tissue specificity (tissue-specific gene expression, TAU > 0.8) in peripheral blood mononuclear cells (PBMCs), kidney, and PAM, while 36 genes (27.3%) showed broad expression across multiple tissues (TAU < 0.6, Fig. 4c, Supplementary Fig. S5 and Supplementary Tables S1 and S13), suggesting a general role in systemic immune and inflammatory responses. Moreover, differential gene expression and time-series analyses revealed 127 prioritized genes were significantly differentially expressed (FDR < 0.05, |log₂FC| > 1) in at least one tissue at various time points post-ASFV infection (Fig. 4c). The mandibular (90 genes), tonsil (78 genes), and mesenteric (76 genes) tissues harbored the highest number of responsive genes, consistent with their central roles in lymphoid immune responses [50] (Supplementary Tables S13 and S14). Prioritized genes such as *GALM, CXCL11, CXCL10*, and *IL15* were consistently upregulated across multiple tissues (Fig. 4c, Supplementary Fig. S5, Supplementary Tables S13–S15), indicating a sustained antiviral immunity activation during ASFV progression. Conversely, genes like *ADD1, CLIP2, PHF21B* were downregulated (Fig. 4c, Supplementary Tables S13–S15), potentially reflecting virus-induced suppression of host immune pathways. Notably, *ADD1* plays a critical role in vesicle packaging and viral endocytosis, with its depletion demonstrating antiviral effects [51].

**Figure 5:**
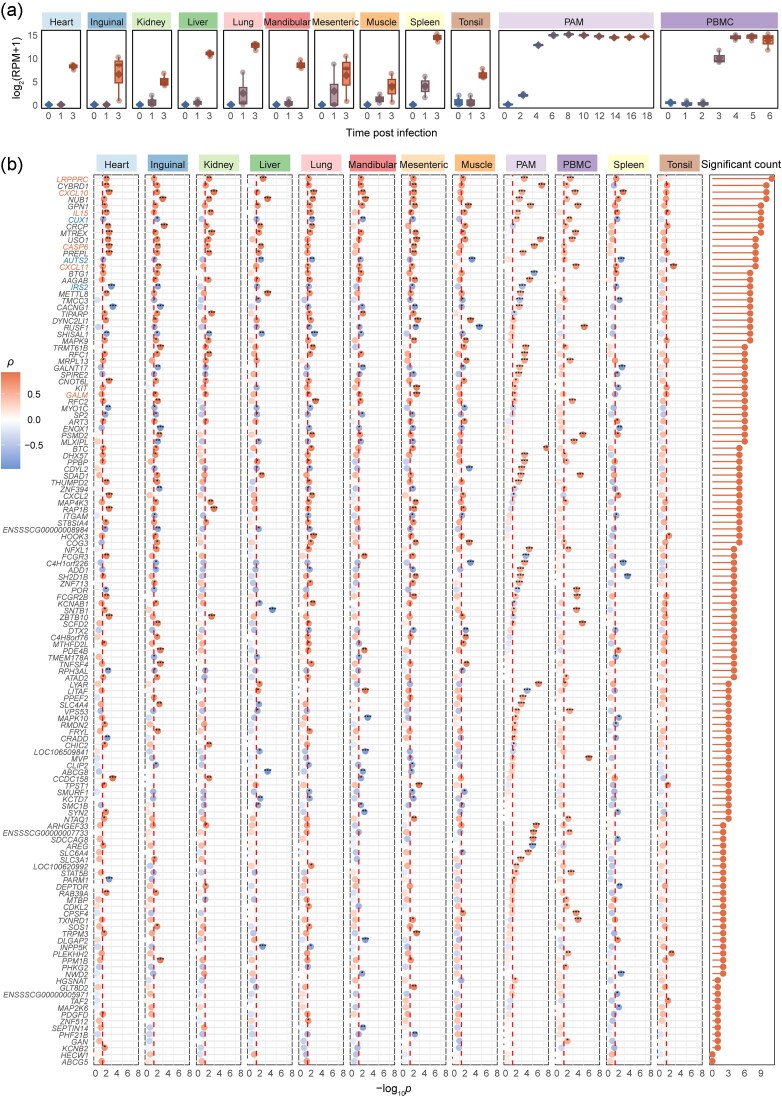
Analyses of ASFV replication and host correlations. (a) ASFV load, measured by RNA reads per million (RPM), across multiple tissues at various time points post-infection. (b) Spearman correlation analysis between ASFV RPM and the expression (TPM) of prioritized host genes.

The dynamics of ASFV replication, as quantified by viral RNA (reads per million, RPM), were both tissue-specific and time-dependent. While all tissues supported progressive viral replication, PAMs were distinguished by the earliest and most rapid replication, showing significant accumulation by 4 h post-infection. A widespread, marked increase in other tissues became evident by 3 days post-infection. Spatially, viral abundance was stratified, with the highest titers (≈20,000 RPM) in primary target tissues (PAM, spleen, PBMC). The lung displayed intermediate levels (≈9,000 RPM), while the heart and various lymph nodes (inguinal, mandibular, mesenteric) exhibited the lowest levels, at several hundred to ∼1,000 RPM (Fig. 5a). To further characterize host–pathogen interactions, we performed Spearman correlation analysis between viral transcript abundance (RPM) and host gene expression (TPM). A subset of consistently upregulated genes, including those involved in host defense and immune signaling (e.g., *LRPPRC* [52], *CXCL10, CXCL11* [53], *CASP6* [54], *IL15* [35]), showed strong positive correlations with viral RPM (Fig. 5b). In contrast, consistently downregulated genes involved in critical host functions, such as transcriptional regulation (*CUX1* [55]) and metabolic homeo stasis (*IRS2* [56]), exhibited significant negative correlations (Fig. 5b). This coordinated, abundance-dependent response indicates that these prioritized genes are not merely reactive but are likely integral to ASFV-driven biological processes, representing key determinants of host resistance and disease severity.

Single-cell RNA-seq analyses of PAMs [49] (target cells for ASFV) identified 127 prioritized genes from a subset of 135 total prioritized genes, filtered for stable expression in the scRNA-seq dataset (Supplementary Tables S1 and S17). Among various cell types post-infection, the Mac_CD163 cells, a transcriptionally distinct PAM subpopulation characterized by high baseline expression of *CD163, MARCO, S100A8*, and *S100A9* [49], exhibited sustained upregulation of 127-gene prioritized module throughout ASFV infection course (Fig. 4e and Supplementary Table S16). This persistent response highlights their specialized role in orchestrating innate immune defenses within the lung, consistent with prior functional descriptions of this subset [49]. Other subtypes of macrophage cells, such as Mac_HLA_DRA, Mac_CREG1, and Mac_PLBD1 also show high expression of prioritized genes early in the infection (Fig. 4e and Supplementary Table S16). Our differential expression analysis specifically focused on this gene set further revealed that 68 prioritized genes were significantly modulated in at least one cell type at specific time points (Fig. 4f), emphasizing their dynamic roles in ASFV infection and immune modulation. For example, *CXCL2*, highly upregulated in the early phase; *CXCL10*, peaking during the mid-phase; and *PPBP* (also known as *CXCL7*), predominantly upregulated in the mid-to-late phase, across multiple immune cell populations (Fig. 4f, Supplementary Fig. S18 and Supplementary Table S17). Conversely, genes such as *TMCC3* (Mac_HLA_DRA), *PHF21B* (Mac_HLA_DRA), *FCGR3* (Mac_CD163), *NTAQ1* (Mac_CD163), *BTC* (Mac_PLBD1), and *PARM1* (Mac_PLBD1) (Fig. 4f, Supplementary Fig. S18 and Supplementary Table S17) exhibited consistent downregulation, potentially reflecting virus-induced suppression signatures targeting these genetically prioritized host pathways.

### Genetic correlations of ASF resistance with other traits

**Figure 6:**
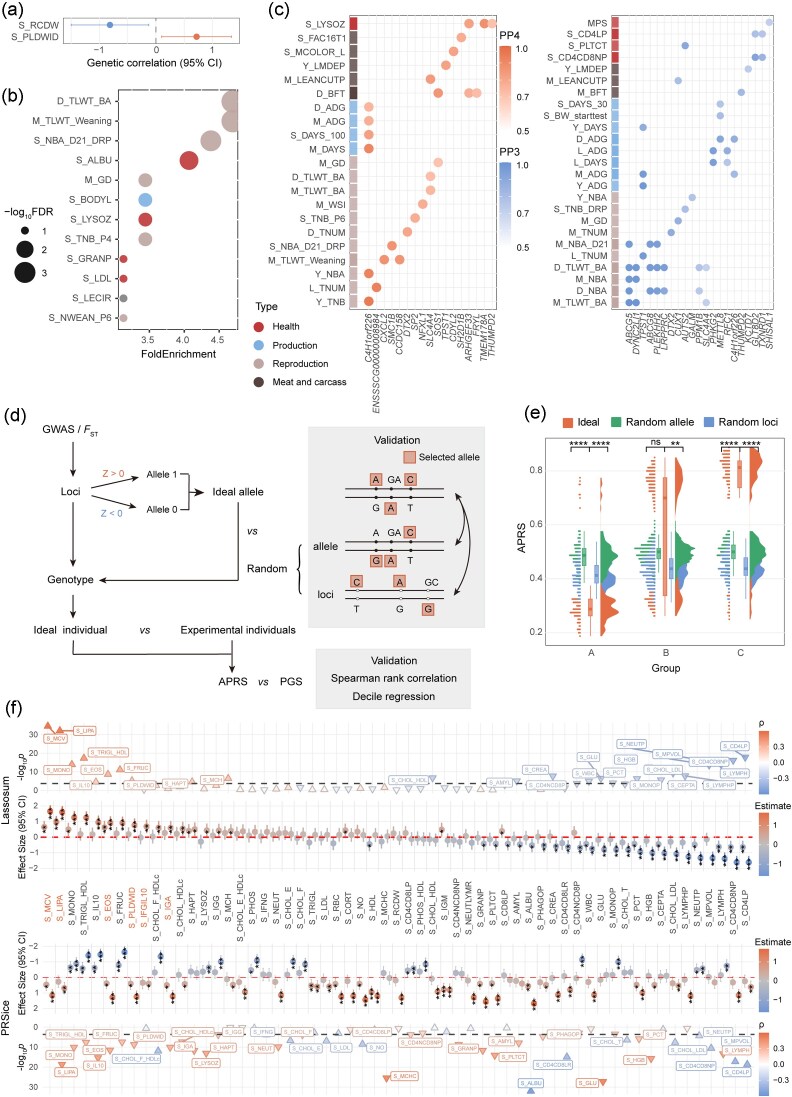
Pleiotropic associations. (a) Forest plot illustrating traits with significant genome-wide genetic correlations with ASF resistance (*P*-value < 0.05). Orange dots indicate positive correlations, and blue dots indicate negative correlations. Each dot represents the effect value, and lines indicate the 95% confidence intervals (CIs). S_PLDWID: platelet distribution width. S_RCDW: red cell distribution width. (b) Bubble plot illustrating the enrichment of prioritized genes in various pig traits. The *y*-axis lists traits, while the *x*-axis represents the enrichment fold. Bubble size corresponds −log_10_FDR, and bubble color reflects the trait categories. (c) Cross-trait colocalization analysis highlighting genes shared between ASF resistance and other pig traits. The *y*-axis lists traits, and the *x*-axis represents genes. Shades of orange denote PP4 values (PP4 > 0.7, evidence for shared causal variants), while shades of blue denote PP3 values (PP3 > 0.7, evidence for alternative causal variants). PP4: posterior probability of hypothesis 4; PP3: posterior probability of hypothesis 3. (d) Schematic representation of the construction and evaluation process for the ASF resistance prediction score (APRS) and polygenic scores (PGS) related to other pig health traits. (e) Comparative analysis of “ideal” APRS vs. randomly constructed APRS. The Wilcoxon rank-sum test with Bonferroni correction was used (**P.adjusted < 0.01, ^****^P.adjusted < 1 × 10^−5^, ns: not significant). The *y*-axis shows APRS scores, and the *x*-axis differentiates experimental groupings. Colors distinguish APRS methods: Ideal (ideal APRS), Random allele (APRS using random alleles), and Random loci (APRS using random loci). (f) Phenome-wide association study (PheWAS) comparing APRS and PGS for pig health traits. The *x*-axis lists health traits, while the *y*-axis displays association metrics: Outer Plot (Panels 1 and 4): The *y*-axis shows −log_10_  *P*-values from Spearman rank correlation analysis. Dot colors indicate association direction (red for positive, blue for negative), with shades reflecting correlation strength (|ρ|). The black dashed line represents the significance threshold (*P*-value < 0.05/236, Bonferroni correction). Significant traits are labeled. Inner Plot (Panels 2 and 3): Regression analysis of PGS values for individuals grouped by APRS deciles (top 10%, mid-50%, bottom 10%). The *y*-axis represents effect estimates, with dots showing effect sizes (*P-value < 0.05, **P-value < 0.01, ***P-value < 0.05/236, Bonferroni correction). Line segments represent 95% confidence intervals (CIs). Dot colors indicate effect magnitude (red for positive, blue for negative), and the red dashed line marks an effect of 0. Trait abbreviations and details are provided in the PigBiobank database.

We assessed the genetic correlations between ASF resistance and 122 traits related to health, growth, and reproduction using publicly available GWAS summary data (121 traits from PigBiobank [32] and one trait concerning Mycoplasmal Pneumonia of Swine, MPS [57]). Notably, ASF resistance showed significant correlations with specific hematological parameters: a positive correlation with platelet distribution width (S_PLDWID) and a negative correlation with red cell distribution width (S_RCDW, *P*-value < 0.05, Fig. 6a and Supplementary Table S18). The positive correlation with S_PLDWID suggests that genetic basis influencing platelet size variability is shared with those conferring ASF resistance. As a marker of platelet size variability, S_PLDWID is linked to immune activation and systemic inflammation, processes that may enhance tolerance against ASF infection [58]. Conversely, the negative correlation with S_RCDW, an indicator of erythrocyte size variability, implies a connection between stable red cell morphology and reduced susceptibility to inflammation-induced damage during ASF infection [59]. Lower S_RCDW may reflect diminished oxidative stress and a more regulated inflammatory response, contributing to ASF resistance. These findings identify S_PLDWID and S_RCDW as potential phenotypic markers for breeding strategies aimed at enhancing ASF resistance while maintaining overall health and performance in pig populations. Incorporating these markers into selective breeding programs could facilitate the development of ASF-resistant breeds, thereby support sustainable disease management and improve animal welfare.

### Pleiotropic associations of prioritized genes

To explore the pleiotropic effects of ASF resistance-prioritized genes, we analyzed their effects with 299 traits derived from 298 PigBiobank GWAS studies [32] and one trait related to MPS [57]. This analysis identified 1,151 gene-trait pairs across 286 GWAS studies, involving 133 prioritized genes (Supplementary Table S19). Notably, 12 traits exhibited significant enrichment, including 4 health-related traits: lysozyme levels (S_LYSOZ), granulocyte phagocytosis (S_GRANP), blood albumin level (S_ALBU), and low-density lipoprotein (S_LDL) (Fig. 6b and Supplementary Table S20). These traits are associated with systemic immunity and inflammation, suggesting shared genetic regions between ASF resistance and immune responses.

Cross-trait colocalization analysis revealed shared potential causal variants (PP4 > 0.7) between ASF resistance and lysozyme levels in genes such as *TMEM178A, ARHGEF33*, and *THUMPD2*, suggesting their involvement in antiviral defense pathways (Fig. 6c and Supplementary Table S21). Conversely, distinct potential causal variants (PP3 > 0.7) were identified for traits like CD4^+^ leukocyte (*GLT8D2, TXNRD1*), platelet counts (*AUST2*), and MPS (*SHISAL1*), indicating genetic linkage, highlighting the locus-specific complexity of the genetic architecture of ASF resistance (Fig. 6c).

Beyond immune-related traits, associations were also observed with reproductive traits (e.g., litter weight), production traits (e.g., body length), and meat traits (e.g., meat quality) (Fig. 6b and c). Key genes implicated in these associations include *C4H1orf226, TPST1*, and *ABCG5*, indicating that ASF resistance signatures may intersect with growth and reproductive traits (Fig. 6b and c). These findings underscore the importance of further studies to balance disease resistance with production performance in breeding programs.

### Construction and application of ASF polygenic resistance score

To evaluate the practical implementation of our findings, we constructed an ASF polygenic resistance score (APRS) based on the ASF-resistance prioritized loci identified in this study and tested its performance within our discovery cohort (the experimental case-control population, *n* = 474). The APRS was constructed by aggregating favorable alleles at independent lead loci within prioritized gene regions, with each individual’s score calculated based on identity-by-state (IBS) similarity to an ideal resistant genotype (Fig. 6d).

Our APRS metric demonstrated strong discriminatory power, effectively separating resistant, susceptible, and deceased pigs (Fig. 6e). To further infer that this performance was not a byproduct of random chance or cohort-specific bias, we compared APRS to scores derived from randomly selected loci or alleles. The randomized scores failed to differentiate between resistant and deceased individuals (Fig. 6e and Supplementary Tables S21), confirming that the predictive power of APRS arises from biologically prioritized loci rather than stochastic inflation, and that it meaningfully contributes to the observed resistance phenotype.

To evaluate the reliability of the APRS, we calculated its association with polygenic scores (PGS) for health traits derived from PigBiobank [32] and subsequently applied to our experimental cohort. PGS for 59 traits overlapping with prioritized gene regions were constructed using lassosum [60], with parameter optimization performed through PUMAS [61]. For consistency test, PGS for the same traits were independently generated using PRSice-2 [62] with the clumping and thresholding (C+T) method.

Our analysis revealed a significant positive correlation between APRS and the PGS for platelet distribution width (S_PLDWID), consistent with the results from the genetic correlation analysis (Fig. 6a, f, Supplementary Tables S18 and S22). Individuals in the top decile of APRS exhibited significantly higher PGS__S_PLDWID_ values compared to those in the bottom decile. This is highly relevant as ASFV infection typically leads to thrombocytopenia and disseminated intravascular coagulation (DIC) [63], suggesting that a genetic predisposition for robust coagulation regulation may support resistance. Additionally, APRS positively correlated with PGS for traits critical to viral defense and host homeostasis, such as IgA levels (S_IGA), which serve as a primary mucosal barrier [64], and the IFN-γ/IL-10 ratio (S_IFGIL10), a marker of the pro- vs. anti-inflammatory balance [65] often disrupted by ASFV-induced cytokine storms [63, 66]. We also observed correlations with mean corpuscular volume (S_MCV) and eosinophil counts (S_EOS), traits known to be impacted by ASFV-mediated vascular damage and leukopenia [63, 66] (Fig. 6f and Supplementary Table S22). The concordance between APRS and independent immune-related traits indicates that APRS captures a biologically meaningful signal of host resistance rather than cohort-specific noise. Collectively, these findings support its utility as a robust genomic metric for evaluating ASF resistance potential and highlight its promise for application in marker-assisted breeding programs.

## Discussion

ASF continues to pose a major threat to global swine production, yet our understanding of host genetic basis conferring resistance remains limited [9, 13]. While acute infections with wild-type ASFV typically result in near-complete mortality within 2 weeks [42, 67, 68], the high proportion of antibody-positive (P32, P62, P72) but antigen-negative (p22) individuals observed among survivors in this study suggests exposure to low-virulence, gene-deleted, or variant strains rather than classical virulent isolates. Such attenuated strains represent a “hidden” epidemiological challenge: they cause milder symptoms and prolonged infection courses, complicating detection and control. Elucidating host genetic responses under these conditions is therefore critical for developing effective breeding strategies in endemic settings.

In this context, our study delineates the genetic architecture underlying host responses to low-virulence ASFV infection. “Resistance” refers to the host’s ability to prevent or control viral infection, including reducing the likelihood of infection or limiting viral replication and spread once infected [69]. In contrast, “tolerance” describes the host’s ability to withstand the effects of established infection, with a tolerant individual experiencing attenuated disease impact [69]. In this study, “resistance” is defined as the host’s ability to survive infection.

By integrating WGS with functional and transcriptomic annotations, alongside predictive modeling, we established a comprehensive framework to dissect the polygenic nature of ASF resistance in pigs, reflected in the diverse genomic distribution of these genes. This approach identified 1,102 candidate genes associated with ASF resistance, which were further refined to 135 high-priority genes through a rigorous prioritization framework. Enrichment analysis revealed their significant roles in immune-related pathways, aligning with known responses to ASF infection, such as chemokine upregulation [47], MAPK signaling activation [48], and immune cell apoptosis, particularly in myeloid and lymphoid lineages [46].

Chemokines play a critical role in orchestrating immune cell recruitment and activation, contributing to both protective immune responses and pathological outcomes [70]. Our study identified chemokines, including *CXCL2, CXCL7, CXCL10*, and *CXCL11*, as key contributors to ASF resistance. *F*_ST_ analysis and AF differences revealed significant genetic differentiation in these genes between ASF-resistant and susceptible populations, highlighting their potential evolutionary importance in shaping resistance traits. Additionally, SMR analysis demonstrated that the expression levels of these chemokines in specific tissues potentially mediate ASF resistance, while DEG profiling further confirmed their dynamic response to ASF infection. Collectively, these findings underscore their dual roles in resistance basis: functioning as putative determinants of genetic predisposition and mediators of infection responses.

Our study also highlights the critical roles of the FcγR (*FCGR2B, FCGR3*) and PI3K-AKT (*SOS1* [45], *MVP* [71, 72], *PARM1* [73], *TMCC3* [74], *SLC3A1* [75], *BTC* [76]) signaling pathways in ASFV infection. The FcγR family mediates immune regulation through IgG binding, with FCGR3 functioning as an activating receptor and FCGR2B as the sole inhibitory receptor [77], exhibiting opposing immunomodulatory effects. Our analyses revealed a negative correlation between *FCGR3* expression and resistance (supported by SMR, coloc, and downregulation in PAM following infection), while *FCGR2B* expression showed a positive correlation with resistance (supported by SMR, colocalization, BN-GWAS, and upregulation in multiple tissues post-infection). As a regulator of the MAPK, PI3K/JAK cellular signaling pathways, and tumorigenesis [44, 45], *SOS1* was identified as having a positive effect on ASF resistance. *MVP* mediates immune signaling through JAK/STAT and MAPK pathways [71, 72], and *SLC3A1* activates AKT signaling, contributing to tumorigenesis [75].

In addition, functional insights into genes supported by at least 3 independent lines of evidence (drawn from a pool of 5 methods: reported immune genes, TWAS, SMR, colocalization, and BN-GWAS) suggest diverse biological roles: *KIT* is implicated in tumor cell evasion of TGF-β-mediated growth inhibition [78]. *SDAD1* modulates microglial inflammatory responses via NF-κB [79], and counteracting porcine reproductive and respiratory syndrome virus infection [80]. *HGSNAT*, encoding a lysosomal membrane acetyltransferase, has been implicated in lysosomal and inflammatory dysregulation when misfolded in mice [81], whereas *ZNF394*, a transcriptional repressor in MAPK pathway, has prognostic value in lung squamous cell carcinoma [82, 83]. Other genes, such as *ZNF713* [84, 85], *CLIP2* (also known as *CYLN2*) [86], *PHF21B* [87, 88], and *SDCCAG8* [89–91], are associated with neurological disorders and reproductive traits, further demonstrating the pleiotropic nature of these loci. These findings suggest that ASFV may exploit these pathways to suppress host immune responses.

ASFV infection triggers a severe systemic crisis, characterized by a cytokine storm and multi-organ hemorrhaging [42, 43, 92]. Clinical manifestations such as depression (brain), hydropericardium (heart), and diarrhea (intestines) underscore the widespread nature of the disease [42, 43, 92]. Our time-course transcriptomic data support this systemic involvement, with the majority (127/132) of prioritized genes exhibiting dynamic expression changes post-infection. While these responses were most robust in lymphoid tissues, viral quantification revealed that non-canonical tissues harbored substantially lower ASFV RNA levels compared to primary targets like the spleen or PAMs. Consequently, the gene expression changes in these non-target tissues likely reflect physiological responses to systemic inflammatory signaling rather than local viral replication. This is supported by the identification of several prioritized genes such as *CXCL10* and *IL15*, which encode secreted proteins that can act as systemic mediators of inter-organ communication [35, 93]. The presence of low-level viral RNA in these tissues is likely a secondary effect of viremia. Crucially, the significant heritability enrichment observed in non-immune organs, including the intestines, brain, and heart, provides genetic support for a resistance network that extends beyond canonical immune sites. Furthermore, approximately 27% (36/132) of the prioritized genes (e.g., *ADD1, ZNF* family members, and *RUSF1*) were broadly expressed across all examined tissues. Together, these findings suggest that ASF resistance is not solely a localized immune event but involves a multi-organ genetic architecture that maintains systemic integrity. Such a cross-tissue network, potentially involving mucosal immunity in the intestine [94] or neuro-endocrine-immune crosstalk [95], warrants further functional investigation to fully elucidate the basis of host resistance.

At the cellular level, macrophages are well established as primary targets of ASFV infection and replication [38]. Our study extends this understanding by demonstrating that the heritability of ASF resistance is specifically enriched in macrophage subtypes, particularly Mac_CD163 and Mac_PLBD1. Consistent with Zheng et al.’s cellular landscape [49], we found the Mac_CD163 subpopulation to be pivotal in the host response. Despite their initial sharp decline in prevalence post-infection, this subset demonstrated a remarkable ability to restrict viral replication, characterized by a low proportion of high-viral-load cells [49]. Our analysis adds a critical layer to this observation: the robust antiviral capacity of Mac_CD163 cells is coupled with the sustained activation of our prioritized resistance genes. Specifically, the continuous upregulation of *CXCL2* and *CXCL10* within this subset reinforces their role as key innate immune effectors [96, 97]. Furthermore, our basal expression analysis identified genes like *CXCL2, CXCL7*, and *BTC* as highly specific to PAMs, which are critical sites for ASFV entry [49]. By linking these specific genetic candidates to established cellular responders, we show that Mac_CD163 cells are not just transcriptomic markers of infection, but are the primary cellular vehicles through which these prioritized resistance factors operate. Collectively, these findings emphasize the interplay between tissue-specific genetic architecture and systemic immune responses in driving ASFV resistance.

ASFV infection is also associated with hemostasis abnormalities and thrombocytopenia [42, 98], findings that are supported by our genetic analysis. Genetic correlation analysis revealed a positive association between ASF resistance and platelet distribution width (S_PLDWID) and a negative association with red blood cell distribution width (S_RCDW). These results suggest that variations in blood cell distribution indices may play a role in shaping the genetic architecture of ASF resistance. Furthermore, the positive correlation between PGS__S_PLDWID_ and APRS underscores the critical role of platelet variability and immune activation in resistance basis. For example, shared genetic signals were observed between ASF resistance and traits such as lysozyme levels (S_LYSOZ), granulocyte phagocytosis (S_GRANP), blood albumin levels (S_ALBU), and low-density lipoprotein (S_LDL). These associations suggest that loci contributing to ASF resistance also influence diverse biological pathways. Elevated lysozyme levels, for instance, may enhance mucosal immunity [99], while improved granulocyte function likely strengthens the innate immune response [100], providing a robust first line of defense against ASFV infection.

Complex traits are inherently polygenic, and the finite number of genomic variants gives rise to widespread genetic overlap and pleiotropy across traits. Many phenotypes are associated with hundreds to thousands of loci, implying the presence of shared causal variants [101]. Consistently, genetic correlation analyses indicate that some variants exert concordant effects on multiple traits [102]. The overlapping signals for ASF resistance and MPS at the *SHISAL1* locus provide insight into multi-trait genetic architecture. Despite both diseases involving respiratory pathology [42, 57, 103], colocalization analysis strongly supports the PP3 model (PP3 = 0.7153, PP4 = 0.0085), indicating that the associations are driven by distinct potential causal variants in close proximity. This suggests that the *SHISAL1* region represents a multi-trait locus with independent regulatory elements modulating responses to different stressors (bacterial for MPS and viral for ASF), rather than a shared genetic signature. Consistent with this, *SHISAL1* is primarily implicated in Wnt and FGF signaling during development, rather than canonical immune pathways [104]. Together, these findings indicate that the observed overlap reflects local linkage within a complex regulatory landscape rather than true pleiotropy.

Beyond immune-related functions, we observed that ASF resistance-prioritized genes are also associated with reproductive (e.g., litter weight) and production traits (e.g., body length). These genetic associations mirror the clinical manifestations of chronic ASFV infection, where affected pigs often exhibit progressive emaciation, growth stunting, and reproductive disturbances such as abortion [42]. The involvement of key genes like *TPST1* (TPST1-deficient mice experience decreased body weight and reproductive performance [105]) and *ABCG5* (mediates cholesterol metabolism [106]) suggests that the physiological trade-offs observed during infection may have a partially shared genetic architecture. Our findings provide a genetic framework for understanding how disease resistance signatures may intersect with host fitness and performance. This underscores the necessity of balanced selection in breeding programs to enhance antiviral resilience without compromising essential production and reproductive outcomes.

To translate these insights into actionable breeding tools, we developed the APRS, leveraging IBS similarity to an “ideal” ASF-resistant genotype. The APRS effectively distinguished resistant, susceptible, and deceased individuals, outperforming randomized controls and demonstrating robustness and predictive capability. Its utility is further supported by concordance with the polygenic architecture of traits relevant to ASFV pathogenesis. PGS capture the cumulative effects of multiple variants and are widely used to estimate genetic predisposition, stratify risk, and interrogate trait associations [101, 107, 108]. The positive correlation of APRS with S_PLDWID and mean corpuscular volume (S_MCV) PGS suggests a genetic link between ASF resistance and the maintenance of hemostatic and vascular integrity, counteracting virus-induced coagulopathy and endothelial damage [63]. Associations with IgA levels (S_IGA) and the IFN-γ/IL-10 ratio (S_IFGIL10) PGS highlight the importance of coordinated immune responses, integrating mucosal defense and cytokine balance to limit viral entry and immunopathology [64, 65]. Moreover, given ASFV-induced lymphopenia and leukopenia, including reduced eosinophils (S_EOS) [66], the positive correlation between APRS and leukocyte-related PGS indicates that resistant individuals may harbor more resilient hematopoietic and immune systems. Collectively, these findings support pleiotropic effects of prioritized loci across multiple physiological systems, contributing to a multilayered defense against ASFV.

Current ASF control strategies, focused on pre-emptive biosecurity and culling [13], necessarily constrain the collection of large-scale biological samples from natural outbreaks. This has limited the scope of genomic investigations into host resistance. Our study, representing the largest cohort-based analysis under natural infection conditions to date, provides critical insights into the host genetic response to ASFV. While the sample size remains modest compared to standard GWAS and may limit power to detect variants of small effect, we mitigated this constraint through a multidimensional analytical framework that integrated diverse omics datasets. Future studies with expanded cohorts will be crucial to validate these findings and refine the genetic architecture of ASF resistance across diverse populations.

It is important to acknowledge that the genetic signals identified in this study were captured under natural ASFV exposure, where environmental complexities and potential co-infections cannot be fully excluded. Consequently, the candidates identified may represent a combination of ASFV-specific resistance and general disease resistance. While traditional research often focuses on “specific resistance” against a single pathogen [109, 110], the quest for “general resistance” remains a major challenge in swine breeding. Our findings, particularly genes involved in the regulation of inflammatory responses and interferon pathways, reflect the host’s capacity to maintain immune homeostasis under multi-pathogen challenges. By integrating these diverse candidate genes into a unified regulatory network, this study provides a systematic map of the host’s genetic defense, spanning from “avoiding infection” and “controlling viral replication” to “tolerating tissue damage and preventing mortality.” This holistic perspective underscores the potential of these loci in breeding programs aimed at improving overall host fitness in complex, real-world production environments.

Functional validation also remains an essential next step. While computational analyses consistently supported the prioritized genes and pathways, approaches such as CRISPR-Cas9 or transgenic models are needed to confirm causal roles. Recent work by Pannhorst et al. [111] demonstrated that the porcine SLA class II complex is indispensable for ASFV infection, with knockout of *SLA-DMA, SLA-DMB*, and *RFXANK* leading to profound replication defects across ASFV isolates. In contrast, our study did not identify SLA-related genes. This discrepancy likely reflects the complex architecture, poor annotation, and high polymorphism of the SLA locus, which together complicate the detection of gene-specific signals in genome-wide analyses.

Our findings, derived from a specific population under controlled conditions, may not fully capture the genetic and environmental diversity in global pig populations. Comparative insights from wild suids help contextualize this limitation. African warthogs, which show natural resistance to ASFV, harbor adaptive introgressed immune loci, including MHC and FCGR loci [25], reflecting pathogen-driven selection. Our results independently highlight FCGR genes in domestic pigs, suggesting conserved protective functions. Additional overlap was observed for the IL, HERC, and SLC [26, 27] gene families, while other wild suid-specific candidates such as *PPEF2* [26], *CFAP69* [26], and *JAKMIP1* [25] appeared in our gene sets to varying degrees. By contrast, genes including *LDHB* [27], *RELA* [112], and members of the TRIM family [27] were not detected in our study population. These comparisons illustrate the value of integrating domestic and wild suid datasets to strengthen biological plausibility.

Lastly, while the APRS demonstrated high discriminatory power in our study, we acknowledge that this evaluation was conducted within the discovery cohort due to the inherent difficulty in obtaining independent validation samples during active ASF outbreaks. To mitigate potential performance inflation and ensure the scientific validity of the APRS, we employed a multi-layered verification strategy. First, our integrated multi-dimensional prioritization strategy (rather than relying solely on GWAS *P*-values) ensures that the included loci possess intrinsic biological relevance. Second, the failure of randomized loci to replicate this performance further supports that the APRS captures specific genetic signals rather than stochastic variation. Third, high APRS scores correspond to a higher statistical likelihood of maintaining immune homeostasis, although absolute survival remains contingent upon the intensity of the viral challenge and environmental stressors. Future studies utilizing independent validation populations or diverse pig breeds will be essential to confirm the cross-population portability of this scoring system and its long-term utility in genomic selection programs. These limitations underscore the importance of continued research to enhance ASF resistance breeding and improve preparedness for ASFV outbreaks.

Our findings lay the foundation for host-targeted ASF control strategies by providing both theoretical insights and practical tools for selective breeding. The integration of ASF-resistance genetic markers into breeding programs could facilitate the development of ASF-resistant pig lines, enhance sustainable swine production, and improve outbreak preparedness. Future research should focus on the functional validation of candidate genes through *in vitro* and *in vivo* assays and the genomic optimization of resistance loci to refine breeding indices. Collectively, this study advances our understanding of the genetic basis of ASF resistance and offers actionable strategies to mitigate the impact of this devastating disease on the swine industry.

## Methods

### Experimental population

The experimental population comprised 474 domestic pigs naturally exposed to ASFV across 4 standardized commercial farms under the same enterprise in Shandong, China. The cohort included a local indigenous breed (LWU, *n* = 90), a developed commercial breed (LLA, *n* = 355), and a hybrid population (DLL, *n* = 29), consisting of 240 boars and 234 sows. All individuals were raised under uniform environmental and management conditions representative of large-scale commercial production.

To ensure accurate classification, infection outcomes were determined through repeated testing: pigs that succumbed to infection were sampled at the point of imminent death, whereas resistant individuals survived for at least 2 months post-outbreak. Several individuals’ status was confirmed via a second round of sampling conducted 2∼3 months after the initial test, with 100% concordance.

Blood and ear tissue (0.5 cm^2^) were collected for phenotyping and genomic DNA extraction, respectively, by the Institute of Animal Science and Veterinary Medicine, Shandong Academy of Agricultural Sciences (Shandong, China). All samples were heat-inactivated at 70°C for 30 min before transport and analysis. After experimental procedures, all materials were autoclaved and disposed of in accordance with biosafety regulations of the China Animal Health and Epidemiology Center.

#### Phenotyping

To assess the infection status and the nature of the circulating ASFV strain, we employed a diagnostic strategy combining broad-spectrum antibody profiling with targeted antigen DNA detection.

Antibody detection was performed using the ID Screen African Swine Fever Indirect ELISA kit (ID. Vet, France) according to the manufacturer’s instructions. This assay targets the ASFV structural proteins P32, P62, and P72, which are highly immunogenic and conserved across a wide range of ASFV lineages, serving as reliable markers for both wild-type and attenuated strain exposure.

Antigen DNA detection was conducted using the RAA fluorescence quantification method (AMPLIFICATION FUTURE, WLE8202KIT). The assay specifically targeted the *KP177R* sequence within the p22 coding region, derived from the ASFV strain China/2018/AnhuiXCGQ (MK128995.1). While p22 is a highly conserved structural protein in wild-type ASFV, the *KP177R* is a recognized hotspot for deletion or mutation in various low-virulence variants and gene-deleted strains. Therefore, the inclusion of this specific target allows for both high-sensitivity detection of viral and the identification of potential gene-deleted variants based on the presence or absence of the p22 genomic fragment. Rapid DNA amplification and fluorescence-based probe detection were employed to ensure maximum sensitivity and specificity in distinguishing ASFV infection patterns.

Based on serological and antigen testing, 474 pigs from an indigenous population were classified into 3 phenotypic groups (Table 1 and Supplementary Table S1): susceptible-dead pigs (Group A, *n* = 108), susceptible-resistant pigs (Group B, *n* = 222), and double-negative pigs (Group C, *n* = 144).

Concurrent testing of environmental samples from the same farm consistently yielded negative results, indicating that viral exposure was primarily mediated through pig-to-pig transmission rather than environmental contamination. Although differences in viral dose or strain cannot be completely excluded, the contrasting outcomes observed among individuals within a shared environment, together with longitudinal monitoring, support the presence of inherent host resistance differences.

#### Genotyping

We used whole-genome re-sequencing approach to genotype each individual, achieving an average sequencing depth of 14.3× and a coverage of 0.98 (Table 1 and Supplementary Table S1). Genomic DNA was isolated from ear tissue of each individual using the CTAB method. Sequencing was conducted on the DNBSEQ-T7 platform (MGI, Shenzhen, China), generating 150-bp paired-end reads with an insert size of 350 bp. For variants calling and quality control, we followed a pipeline as in our previous study [113]. Briefly, the raw FASTQ data underwent quality control, read filtering, and base correction using fastp v0.20.0 with default parameters [114]. High-quality reads were then aligned to the Sscrofa11.1 [115] reference genome using BWA v0.7.17 [116] with the MEM algorithm and parameters optimized for paired-end data. Subsequent processing involved converting SAM files into BAM format and sorting them with samtools v1.10 [117]. Duplicate and unmapped reads were removed with sambamba v0.7.1 [118]. We calculated coverage and depth for each individual with Mosdepth v0.2.9 [119]. Next, we applied GATK v4.1.6 [120] with the HaplotypeCaller function (–read-filter GoodCigarReadFilter) to each sample, generating an intermediate GVCF file, which was then employed in GenotypeGVCFs function for joint genotyping across all samples. The resulting variants were filtered with VCFtools v0.1.13 [121] (–maf 0.05, –max missing 0.9), yielding a total of 23,403,868 variants (including SNPs and indels). Finally, genotypes were phased using BEAGLE v4.1 [122] with default parameters.

To provide a representative genetic background for population-level comparison, 1,730 pigs from the PHARP database [113] were included as controls (Group D, Table 1 and Supplementary Table S1). These individuals were not tested for antibodies or antigens but served as a reference cohort capturing natural allelic variation across diverse commercial and local pig populations, thereby enabling detection of population-specific selection and differentiation signals associated with ASF resistance.

### Population structure analysis

Population structure of the experimental pigs was assessed using PCA, neighbor-joining (NJ) tree construction, and ADMIXTURE analysis. After pruning variants for LD with PLINK v1.9 [123] (–indep-pairwise 50 5 0.4), PCA was conducted (–pca). The NJ tree was built using MEGA v11 [124] and visualized with iTOL v6 [125], and genetic ancestry was inferred via neural-admixture v1.6.3 [126].

### Genomic-based identification of ASF-resistance candidate genes

We employed 4 complementary genomic comparison scenarios to dissect the multifactorial nature of ASFV resistance (Fig. 1 and Table 2). These contrasts targeted specific defense landscape, from pathogen recognition to adaptive immunity, to minimize phenotypic ambiguity and maximize detection power for potential causal loci under natural infection. To identify potential genetic loci under selection due to ASF infection, we employed 2 primary methodologies: GWAS and genetic differentiation analyses combined with AF examination. Initially, GWAS was utilized to identify variants associated with ASF resistance. This method involved scanning the genomes of both resistant and susceptible pigs to identify genetic markers that correlate with resistance to ASF. Recognizing that different resistance or susceptibility to ASFV can shape the genome, we further focused on identifying genomic regions with significant differences in allele frequencies between the resistant and susceptible groups. Specifically, we targeted genomic regions characterized by (i) high differentiation (*F*_ST_): genomic regions showing high differentiation between ASF-resistant and ASF-susceptible groups, indicating strong selective pressure. (ii) Inverse AF pattern: alleles that display opposite frequency trends between ASF-resistant and ASF-susceptible groups, suggesting divergent selection. (iii) Replicable AF pattern: consistent AF trends observed when comparing resistant pigs to control pigs from other breeds that have not experienced ASF outbreaks (e.g., Group D). This replication across different populations strengthens the validity of the identified loci. Variant annotation was performed using R GALLO v1.5 [127].

#### Genome-wide association analysis

To identify potential genetic loci associated with ASF resistance, we conducted a GWAS comparing the Case group to Control group 1 within the experimental pig population. We used GCTA v1.92.4 software (–make-grm) [128] to calculate the kinship matrix. GWAS was performed using GEMMA v0.98.5 software (-lmm 1) [129] with MLMA-LOCO [130] approach. The statistical model applied was


\begin{eqnarray*}
{{y}} = {\mathrm{\mu }} + {{Xb}} + {{Zc}} + \mathop \sum \limits_k {{w}_{ik}}{{u}_k} + e,
\end{eqnarray*}

where *y* denotes the grouping of an individual (1 for control, 2 for case); *μ* is the fixed intercept; *X* is the genotype variable for a SNP (encoded as 0, 1, and 2, corresponding to the 3 allelic states); *b* is the fixed effect that is a function of the difference in allele frequencies between the 2 populations; *Z* is the design matrix of additional fixed-effect covariates; *c* is the vector of effects for sex (2 levels) and farm (4 levels); $\mathop \sum \limits_k {{w}_{ik}}{{u}_k}$ is the fit term for all SNPs on the other chromosomes to control for population differentiation; ${{w}_k}$ is the standardized genotype variable for an SNP *k*; ${{u}_k}$ is the corresponding effect size of SNP *k*, assuming to follow a normal distribution with variance proportional to *p*_0_(1 − *p*_0_)*F_ST_*, where *p*_0_ denotes the AF in the ancestral population; and *e* is the residual error term, assumed to follow a normal distribution [130]. This modeling framework effectively accounts for population structure and provides greater statistical power than conventional linear regression approaches [131]. To further mitigate proximal contamination, a LOCO (leave one chromosome out) scheme was implemented, in which SNPs on all other chromosomes were included as random effects when testing the SNP of interest. We retained loci that met the significance threshold of *P-*value < $\frac{1}{{Me}}$, where *Me* represents the total number of loci obtained post-LD pruning. The LD pruning was executed using PLINK v1.9 with parameters –indep-pairwise 50 5 0.4 [123].

#### Fixation index

We calculated *F*_ST_ values for each genomic segment to measure genetic differentiation using VCFtools v0.1.13 with parameters –fst-window-size 100000 –fst-window-step 10,000. To assess differences in allele frequencies at each locus, we also conducted a chi-square test for allele frequencies at each locus using PLINK v1.9 [123] (–assoc). We considered those that met the following 4 conditions to be candidate loci: (i) the top 1% of highly differentiated segments were retained based on the *F*_ST_ values; (ii) selected loci with a chi-square test *P-*value < $\frac{{0.05}}{N}$, where *N* is the total number of loci; (iii) ensured that loci met both criteria (i) and (ii) in both comparisons: Case vs. Control 1 and Case vs. Control 2; (iv) Consistent direction of AF changes across comparisons. Namely, AF_Case_ > AF_Control1_ and AF_Case_ > AF_Control2_ or AF_Case_ < AF_Control1_ and AF_Case_ < AF_Control2_.

### Gene prioritization

#### Transcriptome-wide association analysis

To prioritize candidate genes by aggregating the cumulative effects of multiple *cis*-variants on predicted expression levels, we conducted TWAS analysis. Using the FarmGTEx TWAS-server [132], we evaluated associations between candidate genes and ASF resistance across 34 tissues from PigGTEx [31] (Supplementary Table S6). Significant associations were identified using a false discovery rate (FDR) < 0.05.

#### Summary-data-based Mendelian randomization and colocalization with gene expression

To identify potential regulatory links by testing the mediation effect of top eQTL signals on ASF resistance, we employed the SMR framework. We used SMR v1.3.1 [133] (–smr-multi) to assess the effect of expression quantitative trait loci (eQTL) (exposure) on ASF resistance (outcome). Instrumental variables were selected based on *P-*value < 0.005 and LD *r*² < 0.3 in the *cis*-region. Significance was defined by *P*_SMR_ < 0.05, *P*_SMR.MULTI_ < 0.05, and *P*_HEIDI_ > 0.05 to exclude heterogeneity.

To further refine prioritized genes by distinguishing shared genetic control from mere genomic linkage, we performed colocalization analysis. Using the R coloc v5.2.3 [134], we tested variant loci within ±1 Mb of significant eQTLs. Signal pairs with posterior probability of hypothesis 4 (PP4) > 0.75 were considered co-localized (shared variants), while those with posterior probability of hypothesis 3 (PP3) > 0.75 indicated independent variants driving the 2 signals.

Both SMR and colocalization utilized pooled *cis*-eQTL data from PigGTEx (34 tissues) [31] (Supplementary Table S6).

#### Bayesian network genome-wide association study

We employed a Bayesian network genome-wide association study (BN-GWAS) to evaluate the network of potential causal relationships between candidate gene expression and ASF resistance traits [41]. BN-GWAS constructs directed gene–gene-phenotype potential causal networks using imputed expression profiles from GWAS and raw expression data from a reference dataset. For this analysis, we used raw expression data from PigGTEx [31] for 5 tissues with sample sizes exceeding 300: muscle, blood, brain, embryo, and liver (Supplementary Table S6).

#### Omnibus gene prioritization score

We integrated the above inference methods to construct a comprehensive gene prioritization score, which ranks candidate genes based on their potential importance for ASF resistance (Table 4). The following criteria were applied:

- A base score of 1 was assigned to each gene identified by GWAS and *F*_ST_ methods, with an additional score of 1 for each repetition in a comparison subgroup.
- An additional 0.5 score was awarded if the gene was previously reported as an immune gene [49, 135] (Supplementary Table S5).
- A score of 1.2 was given if the gene was supported in TWAS or BN-GWAS, with an additional 1.2 score for each repetition in different tissues.
- A count of 1 was assigned if the gene was supported by SMR or coloc, with an additional count for each tissue repetition. Due to the multi-tissue analysis, SMR and coloc inference were log-transformed and weighted by 0.8 to prevent over-representation.
- Independent inference:
  - An additional 3 scores were assigned if a gene was supported in all 5 methods.
  - An additional 2 scores were given if the gene was supported in 3 or 4 methods.
  - An additional 1 score was awarded if the gene was supported in 2 methods.

### Pathway enrichment analysis

Pathway enrichment analyses were performed on the prioritized gene sets using Gene Ontology (GO), Kyoto Encyclopedia of Genes and Genomes (KEGG), Reactome, and QTL databases. GO and KEGG enrichment were carried out using R clusterProfiler v4.6.2 [136], Reactome enrichment was done with R ReactomePA v1.42.0 [137], and QTL annotation and enrichment were conducted using R GALLO v1.5 [127]. All statistical analyses were corrected for multiple comparisons using the Benjamini–Hochberg (BH) method, and results with *P*. adj < 0.05 were considered significant.

### Tissue- and cell-type heritability enrichment analysis

We employed the LDSC-SEG [138] model to assess genetic heritability enrichment across 34 tissues from the PigGTEx dataset [31] (Supplementary Table S6). Recognizing that PAMs are primary target cells for ASF infection, we further extended the analysis to 8 cell types identified in PAMs. These cell types were characterized using single-cell transcriptomic data from 118,316 cells derived from 13 *in vitro* samples [49].

The LD reference panel was constructed using PGRP v1 [31], comprising genomic data from 1,602 individuals representing over 100 breeds. Tissue- and cell-type-specific gene regions were defined based on the top 1,000 most highly expressed genes in each tissue or cell type, with an additional 100 kb window to capture surrounding regulatory elements [32, 138].

### Transcriptome annotation

#### Multi-tissue bulk transcriptomic analyses

To annotate the prioritized genes within a broader biological context, we analyzed 2 publicly available bulk RNA-sequencing datasets retrieved from the NCBI SRA [139] (PRJNA960638) and CNCB-NGDC GSA [140, 141] (PRJCA003613 [142]) databases (Supplementary Table S1). These datasets encompass transcriptomic profiles from 12 pig tissues and cell types at multiple time points after ASFV infection vs. uninfected controls, including PAMs, PBMCs, heart, kidney, liver, lung, inguinal, mandibular, mesenteric, muscle, spleen, and tonsils.

##### Quality control and read mapping

Raw RNA sequencing reads underwent quality control using fastp v0.20.0 [114] with default parameters. High-quality reads were then mapped to the Sscrofa11.1 reference genome using HISAT2 v2.1.0 [143]. Read counts were quantified using featureCounts v2.0.3 (-t exon -g gene_id) [144], and gene expression was quantified at the transcriptional level in transcripts per million (TPM). Genes were considered expressed if TPM > 0.1 in at least 20% of samples. After applying this threshold, 23,331 genes remained available for downstream analyses, including 132 prioritized genes, which were retained for further investigation.

##### Tissue-specific expression analysis

We assessed the tissue specificity of the prioritized genes using tissue-specific gene expression (TAU) and expression specificity scores (ESS) indices:

TAU index quantifies the specificity of gene expression across tissues, ranging from 0 to 1, where values closer to 1 indicate higher tissue specificity [145]. It was calculated as


\begin{eqnarray*}
\mathrm{ TAU}{\mathrm{\ }} = \frac{{\mathop \sum \nolimits_{i = 1}^n \left( {1 - \frac{{{{x}_i}}}{{{{x}_{\mathrm{ max}}}}}} \right)}}{{n - 1}},
\end{eqnarray*}

where *n* is the number of tissues, ${{x}_i}$ represents the expression level of a gene in a given tissue, and ${{x}_{\mathrm{ max}}}$ denotes its highest expression value across all tissues.

ESS Index measures the degree to which a gene is preferentially expressed in a specific tissue, also ranging from 0 to 1, with values closer to 1 indicating stronger expression bias [146]. It was computed as

$\mathrm{ ESS} = \frac{{\mathrm{ med}( {{{\mathrm{ log}}_2}\mathrm{ TPM}} )}}{{\sum \mathrm{ med}( {{{\mathrm{ log}}_2}\mathrm{ TPM}} )}}.$

##### DEG identification

To identify differentially expressed prioritized genes across different infection time points, we performed differential expression analysis using DESeq2 v1.34.0 [147, 148] for each tissue separately, incorporating time as a factor in the experimental design. Genes were considered significantly differentially expressed if they met the following criteria: FDR < 0.05 and the absolute log2FoldChange (|log₂FC|) > 1.

##### Time-series analysis of dynamic gene expression

Given that transcriptomic data were collected at multiple post-infection time points, we conducted time-series analysis using R maSigPro v1.66.0 [149]. To account for temporal trends, we applied polynomial regression models with the “backward” variable selection method, setting different degrees for different tissues:

- PAM: degree = 4.
- PBMC: degree = 5.
- Other tissues: degree = 2.

The selection of polynomial degrees was based on the complexity of the time-course experimental design. Specifically, PAM and PBMC datasets had a more intricate temporal structure compared to other tissues, necessitating higher-degree polynomials to better capture gene expression dynamics. Preliminary testing indicated that the chosen degrees provided an optimal balance between model fit and the number of significantly dynamic genes identified. Genes were classified as dynamically responsive genes if they satisfied the following thresholds: FDR < 0.05 and coefficient of determination (*R*²) > 0.5.

#### Viral transcriptomic analysis

Unmapped reads from the host alignment were aligned to ASFV reference sequences (MK333180.1.fa, MT748042.2.fa) using Bowtie2 v2.3.5.1 [150]. Viral read counts were generated with featureCounts v2.0.3 [144] (-t CDS -g gene_name) and normalized to RPM to quantify viral load. Spearman’s rank correlation coefficient was used to assess the relationship between viral RPM and host prioritized gene expression (TPM) across tissues.

#### Single-cell transcriptomic analyses of PAM

##### Data processing

We retrieved ASFV-infected PAM single-cell RNA sequencing data from the NCBI SRA (PRJNA706032 [49]) (Supplementary Table S1). The raw sequencing data were processed using Cell Ranger v7.0.1 [151] with the Sscrofa11.1 reference genome [115]. To ensure high data quality, we applied stringent filtering criteria:

- Mitochondrial RNA content < 10% of total RNA.
- Number of detected genes per cell: between 500 and 7,500 [49].

After applying this threshold, 118,316 cells and 14,871 genes remained for downstream analyses, including 127 prioritized genes selected for further investigation. Subsequent analyses were performed using R Seurat v5.0.1 [152]. Cell clustering was performed using the FindClusters function with a resolution parameter of 0.2, ensuring a biologically relevant granularity of clusters. Based on marker genes from Zheng et al. [49], cells were classified into 5 major populations: macrophages (Mac), mast cells (Mast), T cells (T), proliferating cells (Pro), and epithelial cells (Epi). The macrophage population was further subclustered into 4 subtypes: Mac_HLA_DRA, Mac_CD163, Mac_CREG1, and Mac_PLBD1 (Fig. 3d).

##### Prioritized gene expression scoring

To quantify the expression levels of prioritized genes across different cell types, we employed the AddModuleScore function in Seurat v5.0.1 [152], which calculates a module score for a predefined gene set within individual cells. To assess infection-induced changes, module scores of infected cells (categorized by cell type and infection time) were compared to control cells using a 2-sided Welch’s *t*-test [153].

##### Differential gene expression analysis

Differential expression analysis of prioritized genes was conducted using the FindMarkers function in Seurat v5.0.1 [152]. Comparisons were conducted between infected and uninfected groups across different cell types and infection time points. Significantly DEGs were defined as those meeting the following criteria: FDR < 0.05 and |log₂FC| > 1.

### Genetic correlation analysis

We utilized the LD score regression model in LDSC v1.0.1 [102] to estimate the genetic correlation between ASF resistance and other pig traits. A total of 122 GWAS summary datasets were analyzed, encompassing 121 datasets from PigBiobank [32] (initially 268, with 147 excluded due to insufficient sample size or low heritability) and one dataset from a published GWAS on MPS [57].

The LD reference panel was constructed using PGRP v1 [31], ensuring a comprehensive representation of SNP linkage patterns. Each GWAS summary dataset was standardized by aligning alleles (A1, A2) and effect sizes. Quality control filters were applied, retaining SNP loci with |*Z*| < 5 to exclude potential outliers and enhance result robustness.

### Pleiotropy annotation

To evaluate the pleiotropic effects of ASF resistance-associated genes, we analyzed the overlap between the prioritized gene set for ASF resistance and significant genes identified in other phenotypes. This analysis incorporated data from 298 meta-GWAS studies in the PigBiobank [32] and a published GWAS on MPS [57].

The significance of gene overlaps with phenotypes was determined using hypergeometric tests. For overlapping gene-phenotype pairs (from 268 traits), we applied co-localization analysis using the R coloc v5.2.3 [134]. Genetic variants within a 50-kb window upstream and downstream of lead SNPs were examined. Signal pairs with a posterior probability of hypothesis 4 (PP4 > 0.7) were considered co-localized, indicating potential shared causal variants. Conversely, pairs with posterior probability of hypothesis 3 (PP3 > 0.7) were interpreted as significantly associated but driven by distinct potential causal variants.

### Construction of APRS using prioritized loci

#### Definition of APRS

IBS refers to the condition where 2 individuals share the same allele. In this context, IBS states are categorized as 0, 1, or 2 based on the number of shared alleles. Here, we define the IBS-based distance between an experimental and an “ideal” individual as the APRS (Fig. 6d). The APRS evaluates the genetic similarity between an individual and an ideal ASF-resistant genotype, enabling prediction of ASF resistance. The IBS distance is calculated as follows:


\begin{eqnarray*}
\mathrm{ IBS} = \frac{{\left( {{\mathrm{ Number}}\ \mathrm{ of}\ \mathrm{ IBS}2} \right) + \left( {0.5*{\mathrm{ Number}}\ \mathrm{ of}\ \mathrm{ IBS}1} \right)}}{{{\mathrm{ Total}}\ {\mathrm{ Number}}\ \mathrm{ of}\ {\mathrm{ SNPs}}}},
\end{eqnarray*}

where IBS2 represents loci where both alleles match the ideal genotype, and IBS1 represents loci where only one allele matches.

#### Design of ideal genotype

A total of 40 independent loci were selected to define the ideal ASF-resistant genotype. Specifically, within 135 high-confidence gene regions, the most significantly associated (*P*-values) SNPs were first identified as candidate loci. LD clumping (PLINK v1.9 [123], –indep-pairwise 50 5 0.1) was then applied to retain independent, non-redundant lead SNPs for inclusion in the final APRS model. The assignment of ideal alleles was based on statistical evidence from GWAS and *F*_ST_ analyses (Fig. 6d). The specific criteria for ideal allele selection were as follows:

##### GWAS-derived loci

- If the *Z*-score of the SNP was positive (*Z* > 0), the effect allele (minor allele) was designated as the ideal allele.
- If the *Z*-score was negative (*Z* < 0), the reference allele (major allele) was assigned as the ideal allele.

The *Z*-score for each SNP was calculated as


\begin{eqnarray*}
{{Z}} = \frac{\beta }{{\mathrm{ SE}}},
\end{eqnarray*}

where *β* represents the effect size, and SE is the standard error.

##### *F*_ST_-derived loci

- The *Z*-score was defined as the sum of the standardized statistics from 2 independent comparisons (case/control1 and case/control2).
- The sign of the *Z*-score (positive or negative) was determined based on the odds ratio (OR), ensuring alignment with the direction of selection pressure.

Once the ideal alleles were assigned, the IBS distance between each individual and the ideal genotype was computed using PLINK v1.9 [123] (–cluster-matrix).

#### Statistical robustness and indirect validation of APRS

Given the logistical and biosecurity constraints of collecting large-scale independent cohorts during active ASF outbreaks, we implemented a multi-layered framework to evaluate the robustness and biological consistency of the APRS within the study population.

- APRS construction incorporated multi-dimensional evidence, relying on loci prioritized through an integrative framework rather than solely on GWAS significance, thereby ensuring independent functional support for each included locus.
- Internal robustness was assessed via permutation testing.
- Biological consistency was examined by correlating APRS with PGS for 59 independent health-related traits relevant to ASF resistance, including hematological and immune parameters.

#### Permutation by randomized comparison

To assess the specificity and robustness of the APRS, we performed a randomized permutation within our discovery cohort (the experimental case-control population, *n* = 474). This involved:

- Selecting an equal number of random loci using PLINK v1.9 [123] (–thin-count parameter).
- Generating “alternative ideal individuals” by disrupting the ideal alleles at the defined loci.

The resistance scores obtained from these random loci served as benchmarks against the APRS values derived from the prioritized ASF-resistance-associated loci. This approach ensured that the predictive power of APRS was attributable to ASF-specific genetic variation rather than random genomic background noise.

### PGS analysis

Based on the pleiotropy annotation results, 59 health traits from PigBiobank [32] were identified as overlapping with prioritized gene regions.

#### PGS construction and parameter optimization

PGS for these traits were generated using lassosum [60], with PGRP v1 [31] as the LD reference panel. To ensure model robustness and prevent overfitting, PUMAS [61] was used to subsample PigBiobank GWAS summary statistics, implementing a training-testing data split, cross-validation, and repeated learning. This approach optimized the shrinkage coefficient (*s*) and lambda (λ) parameters to achieve maximum *R*².

To substantiate consistency, PGS were also constructed using PRSice-2 [62] with the “clumping and thresholding” (C+T) method (–bar-levels 5e-8, 1e-5, 0.001, 0.05, 0.1, 0.5), allowing direct comparison between the 2 approaches.

#### Association analysis in the experimental cohort

These externally trained PGS models were subsequently applied to the pigs in our experimental case-control population. The association between the externally derived PGS and our internally constructed APRS was evaluated using the Spearman rank correlation coefficient. To enhance interpretability and highlight extreme genetic profiles, the experimental population was divided into deciles based on APRS values. Linear regression analysis was conducted using APRS as the response variable and standardized PGS as the predictor variable, focusing on statistical significance in the highest decile compared to the fifth and the lowest (bottom) deciles.

## Additional files

**Supplementary Figure S1:** Genetic structure of the pig population. (a)–(c). Analyses based on the combined population (experimental + control); (d)–(e). Analyses restricted to the experimental population. (a) Principal component analysis (PCA) based on genome-wide SNPs illustrating genetic relationships among individuals. (b) Neighbor-joining (NJ) tree depicting phylogenetic clustering based on genetic distances. (c) ADMIXTURE analysis showing ancestral components at the optimal K. (d) PCA colored by breed. (e) PCA colored by disease phenotype.

**Supplementary Figure S2:** Overview of ASF resistance candidate genes across subgroups. (a) Summary of loci and genes identified via GWAS across subgroups. (b) Summary of loci and genes identified through *F*_ST_ and allele frequency testing across subgroups.

**Supplementary Figure S3:** Gene prioritization scoring. (a) Distribution of gene prioritization scores, showing the number of genes across different score ranges. (b) TWAS *Z*-values for the top 10 prioritized genes.

**Supplementary Figure S4:** Expression of *PPEF2* in blood and its role in ASF resistance. (a) Colocalization analysis of *PPEF2* eQTLs in blood with ASF resistance GWAS results. (b) SMR analysis of *PPEF2* eQTLs in blood with ASF resistance GWAS. (c) Positive correlation between *PPEF2* eQTLs in blood and ASF resistance GWAS signals.

**Supplementary Figure S5:** Bulk RNA analysis of priority genes. (a) Expression distribution (TPM) of prioritized genes across tissues. (b) Distribution of TAU scores and the number of prioritized genes across major tissue types. (c) Correlation between TAU scores and maximum ESS scores. (d) Expression levels of *GALM, CXCL10, IL15*, and *CXCL11* across different tissues under various infection conditions. (e) Correlation between ASFV load (RPM) and host prioritized gene expression (TPM).

**Supplementary Figure S6:** Temporal expression analysis of priority genes in PAM.

**Supplementary Figure S7:** Temporal expression analysis of priority genes in PBMC.

**Supplementary Figure S8:** Temporal expression analysis of priority genes in heart.

**Supplementary Figure S9:** Temporal expression analysis of priority genes in inguinal.

**Supplementary Figure S10:** Temporal expression analysis of priority genes in kidney.

**Supplementary Figure S11:** Temporal expression analysis of priority genes in liver.

**Supplementary Figure S12:** Temporal expression analysis of priority genes in lung.

**Supplementary Figure S13:** Temporal expression analysis of priority genes in mandibular.

**Supplementary Figure S14:** Temporal expression analysis of priority genes in mesenteric.

**Supplementary Figure S15:** Temporal expression analysis of priority genes in muscle.

**Supplementary Figure S16:** Temporal expression analysis of priority genes in spleen.

**Supplementary Figure S17:** Temporal expression analysis of priority genes in tonsil.

**Supplementary Figure S18:** Differential expression of prioritized genes in PAM subtypes under various infection states.

**Supplementary Figure S19:** Genes co-localized with ASF resistance and lysozyme levels.

**Supplementary Table S1:** Individual data information used in this project, including data cited from PHARP and public data downloaded from NCBI.

**Supplementary Table S2:** ASF-resistance candidate genes identified by the GWAS method.

**Supplementary Table S3:** ASF-resistance candidate genes identified by the *F_ST_* method.

**Supplementary Table S4:** Gene prioritization scoring for ASF-resistance candidate genes.

**Supplementary Table S5:** Immune-related genes reported in previous studies.

**Supplementary Table S6:** Summary of 34 pig tissues and sample size included from the PigGTEx project.

**Supplementary Table S7:** Significant ASF-resistance candidate genes that function in immune-related tissues identified by TWAS method.

**Supplementary Table S8:** Causal associations between candidate gene expression in specific tissues and ASF resistance in SMR.

**Supplementary Table S9:** Pairs of entries with significant association between candidate gene expression in specific tissues and ASF resistance in co-localization analysis.

**Supplementary Table S10:** Candidate genes with causal effect on ASF resistance in BN-GWAS.

**Supplementary Table S11:** GO, KEGG, Reactome and QTL pathway enrichment for prioritized genes.

**Supplementary Table S12:** Tissue and cell heritability enrichment results.

**Supplementary Table S13:** ESS and TAU values of prioritized genes.

**Supplementary Table S14:** Differential expression of prioritized genes across tissues after ASF infection.

**Supplementary Table S15:** Time-series expression profiles of prioritized genes in tissues.

**Supplementary Table S16:** Scores of prioritized genes in cellular and disease processes.

**Supplementary Table S17:** Differential expression of prioritized genes in cell types within PAM.

**Supplementary Table S18:** Pheno-genetic correlation between ASF resistance and other traits from PigBiobank and MPS.

**Supplementary Table S19:** Overlap between prioritized genes and GWAS-identified genes from PigBiobank and MPS.

**Supplementary Table S20:** Enrichment analysis of prioritized genes in GWAS datasets from PigBiobank.

**Supplementary Table S21:** Cross-trait colocalization analysis results.

**Supplementary Table S22:** Comparison of ideal IBS constructs with randomized models.

## Supplementary Material

giag066_Supplemental_Files

giag066_Authors_Response_To_Reviewer_Comments_original_submission

giag066_GIGA-D-26-00031_original_submission

giag066_GIGA-D-26-00031_revision_1

giag066_Reviewer_1_Report_original_submission Reviewer 1 -- 3/1/2026

giag066_Reviewer_2_Report_original_submission Reviewer 2 -- 3/30/2026

giag066_Reviewer_2_Report_revision_1 Reviewer 2 -- 5/18/2026

## Data Availability

The whole-genome sequencing data for the 474 experimental pigs generated in this study are available in the NCBI SRA under accession PRJNA1290525. The processed SNP genotype data (VCF format) central to our analysis have been submitted to the GVM under accession PRJCA057587. Additionally, genomic data for other pig breeds are available at PHARP [113]. The public transcriptome datasets utilized in this study can be accessed through the Sequence Read Archive (SRA) and the Gene Expression Omnibus (GEO), as specified in the Methods section. EQTL data can be accessed via PigGTEx [31], and GWAS summary statistics for pleiotropic analyses are available upon request from PigBiobank [32].
