# Supplementary material for: Integrated multi-omics profiling identifies genetic loci of African swine fever resistance in pigs
Source: Gigascience. 2026 May 30;15:giag066. doi: 10.1093/gigascience/giag066 (PMC13289747; doi:10.1093/gigascience/giag066)
Supplement: giag066_Supplemental_Files [file giag066_supplemental_files.zip › Supplementary information-revised.docx]

# Integrated multi-omics profiling identifies genetic loci of African swine fever resistance in pigs

Xiaowei Ye a, Qinqin Xie a, Caiyun Cao a, Shuang Liu a, Wenbo Sun b, Zhe Zhang a, c, Qishan Wang a, c, Yuchun Pan a, c, *, Zhen Wang a, c, *

a Zhejiang Key Laboratory of nutrition and breeding for high-quality animal products, College of Animal Sciences, Zhejiang University, Hangzhou, Zhejiang 310058, China

^b^ Shandong Key Laboratory of Animal Disease Control and Breeding, Institute of Animal Science and Veterinary Medicine, Shandong Academy of Agricultural Sciences, Jinan, Shandong 250100, China

^c^ Hainan Institute, Zhejiang University, Yongyou Industrial Park, Yazhou Bay Sci-Tech City, Sanya 572000, China

*Corresponding author: Zhen Wang (wangzhen20@zju.edu.cn) and Yuchun Pan (panyc@zju.edu.cn)

Short running title: Resistance loci for African swine fever

E-mail addresses:

XW. Y: ye_xw@zju.edu.cn

QQ. X: qinqin.xie@zju.edu.cn

CY. C: ccyun@zju.edu.cn

S. L: liushuang9917@zju.edu.cn

WB. S: sunwenbo@saas.ac.cn

Z. Z: zhe_zhang@zju.edu.cn

QS. W: wangqishan@zju.edu.cn

YC. P: [panyc@zju.edu.cn](mailto:panyc@zju.edu.cn)

Z. W: [wangzhen20@zju.edu.cn](mailto:wangzhen20@zju.edu.cn)


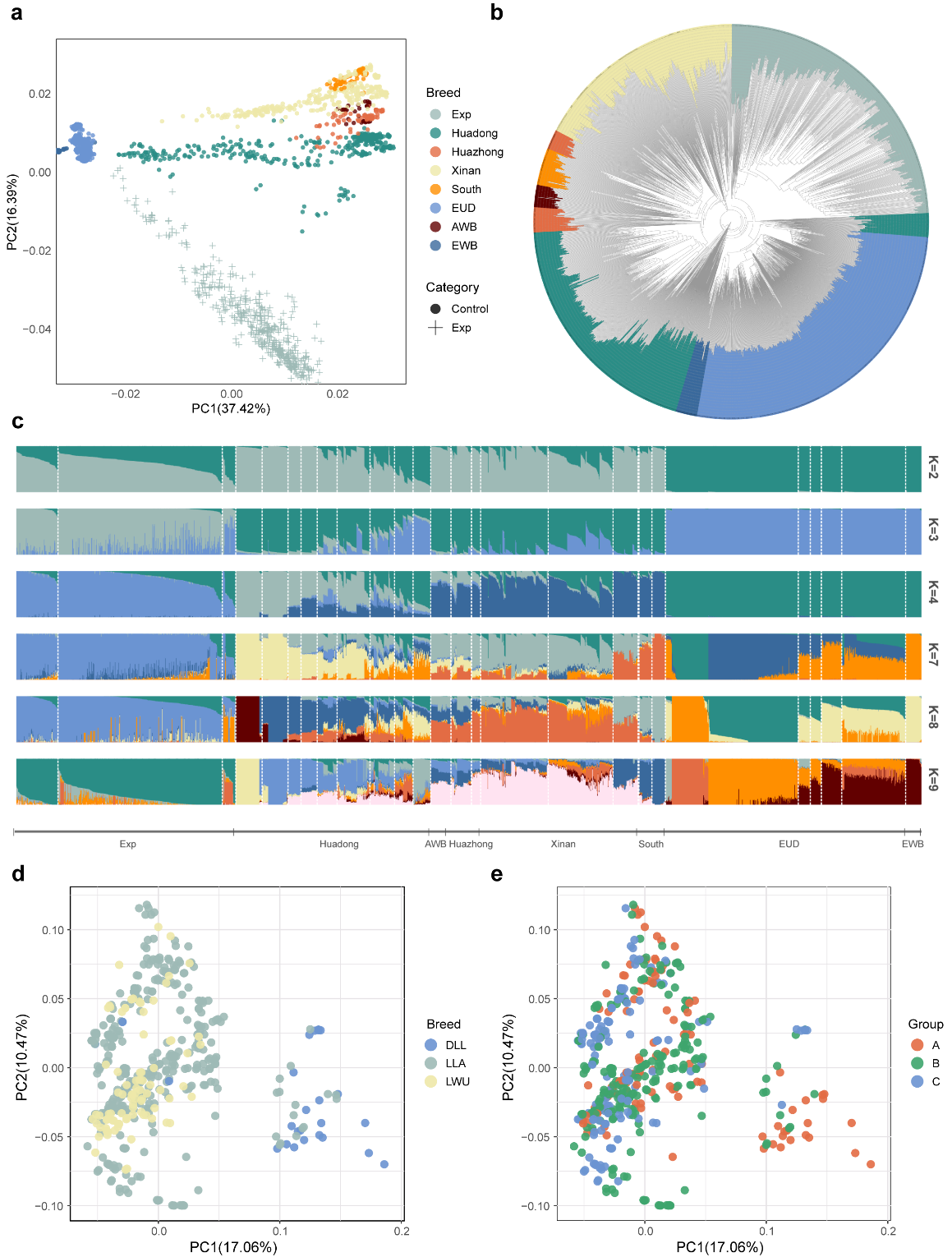


##### Supplementary Figure S1. Genetic structure of the pig population.

**a-c. Analyses based on the combined population (experimental + control);**

**d-e. Analyses restricted to the experimental population.**

**a.** Principal component analysis (PCA) based on genome-wide SNPs illustrating genetic relationships among individuals.

**b.** Neighbor-joining (NJ) tree depicting phylogenetic clustering based on genetic distances.

**c.** ADMIXTURE analysis showing ancestral components at the optimal K.

**d.** PCA colored by breed.

**e.** PCA colored by disease phenotype.


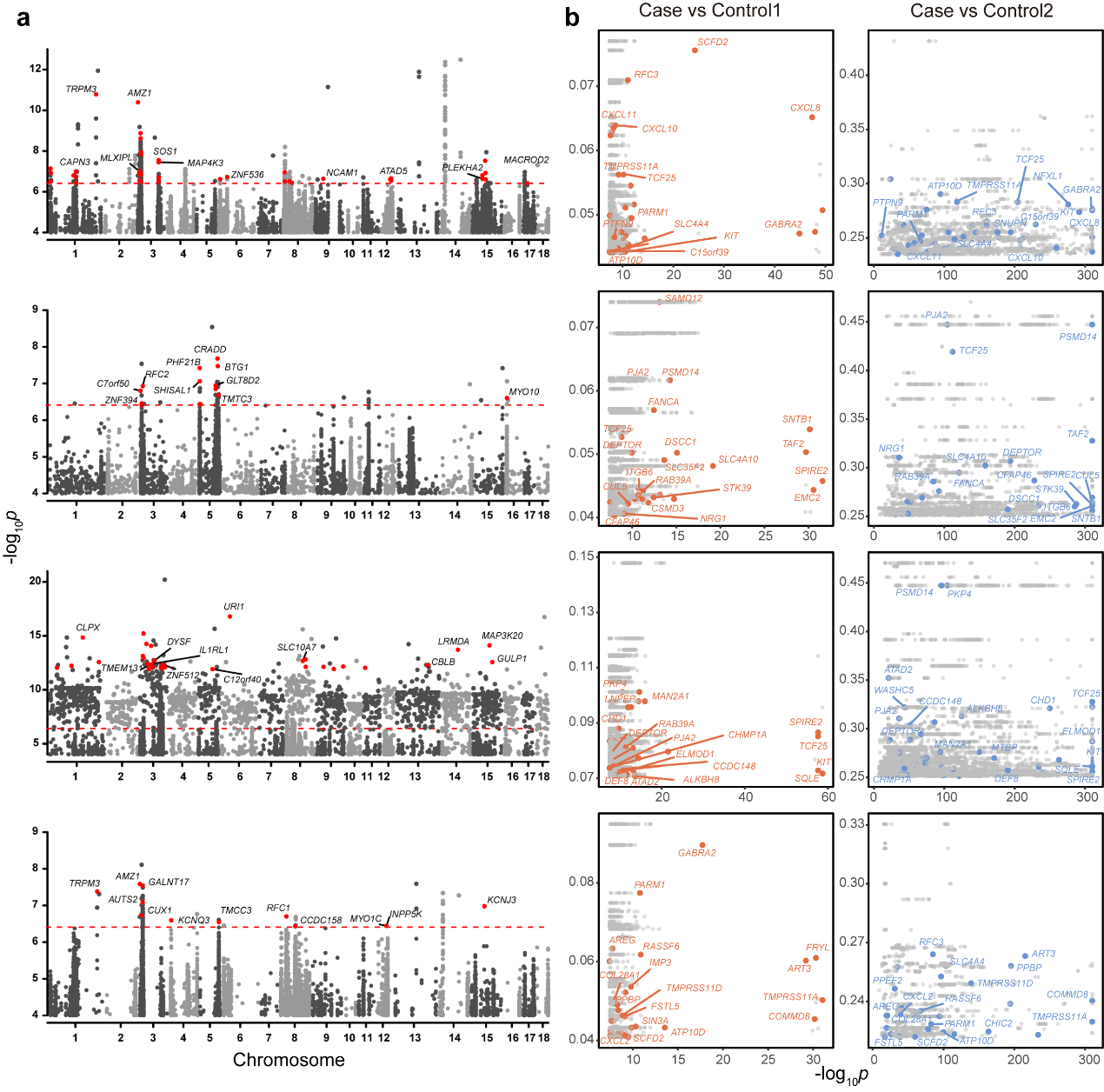


##### Supplementary Figure S2. Overview of ASF resistance candidate genes across subgroups.

**a.** Summary of loci and genes identified via GWAS across subgroups.

**b.** Summary of loci and genes identified through F_ST_ and allele frequency testing across subgroups.


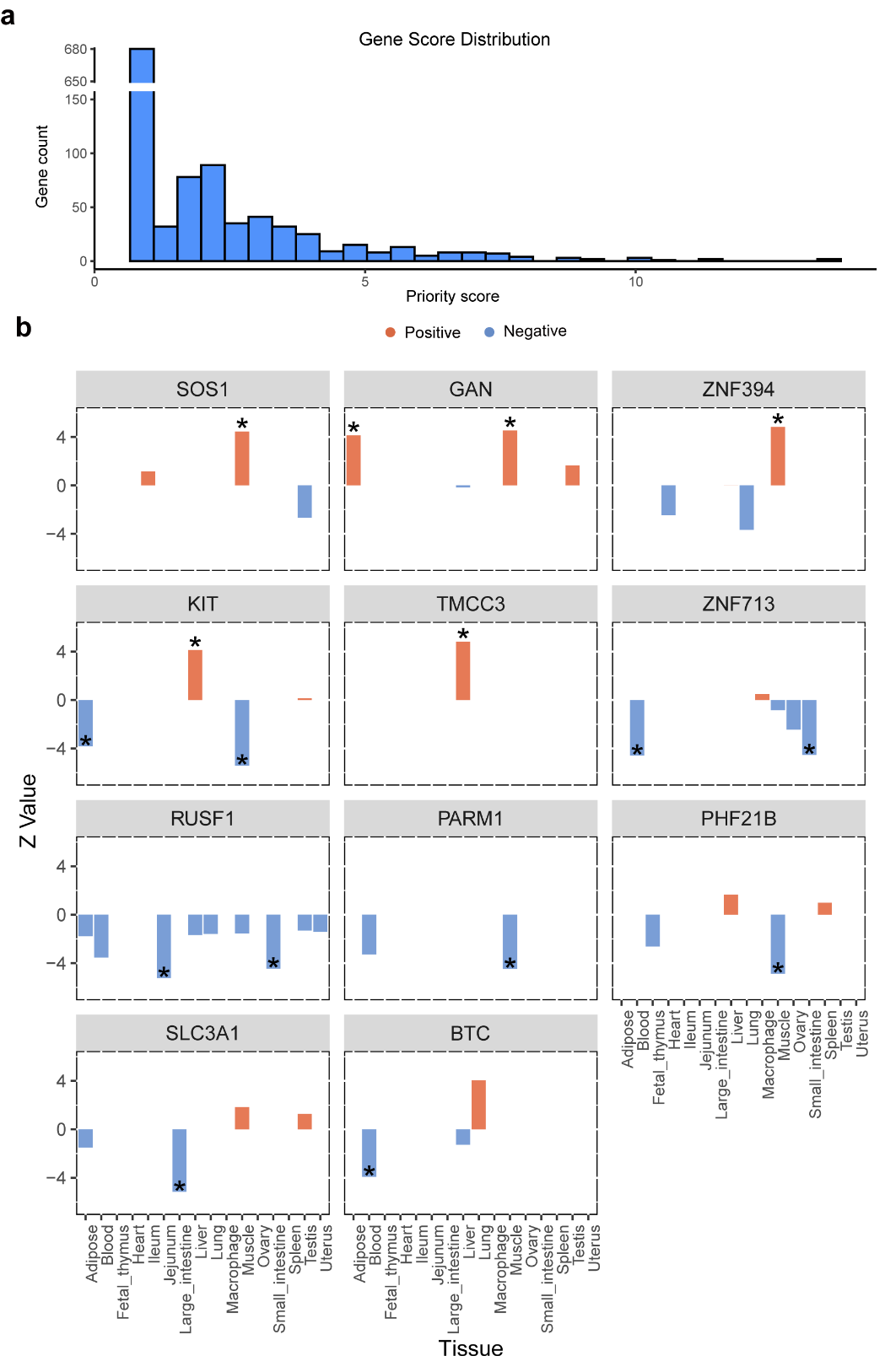


##### Supplementary Figure S3. Gene prioritization scoring.

**a.** Distribution of gene prioritization scores, showing the number of genes across different score ranges.

**b.** TWAS Z-values for the top 10 prioritized genes.


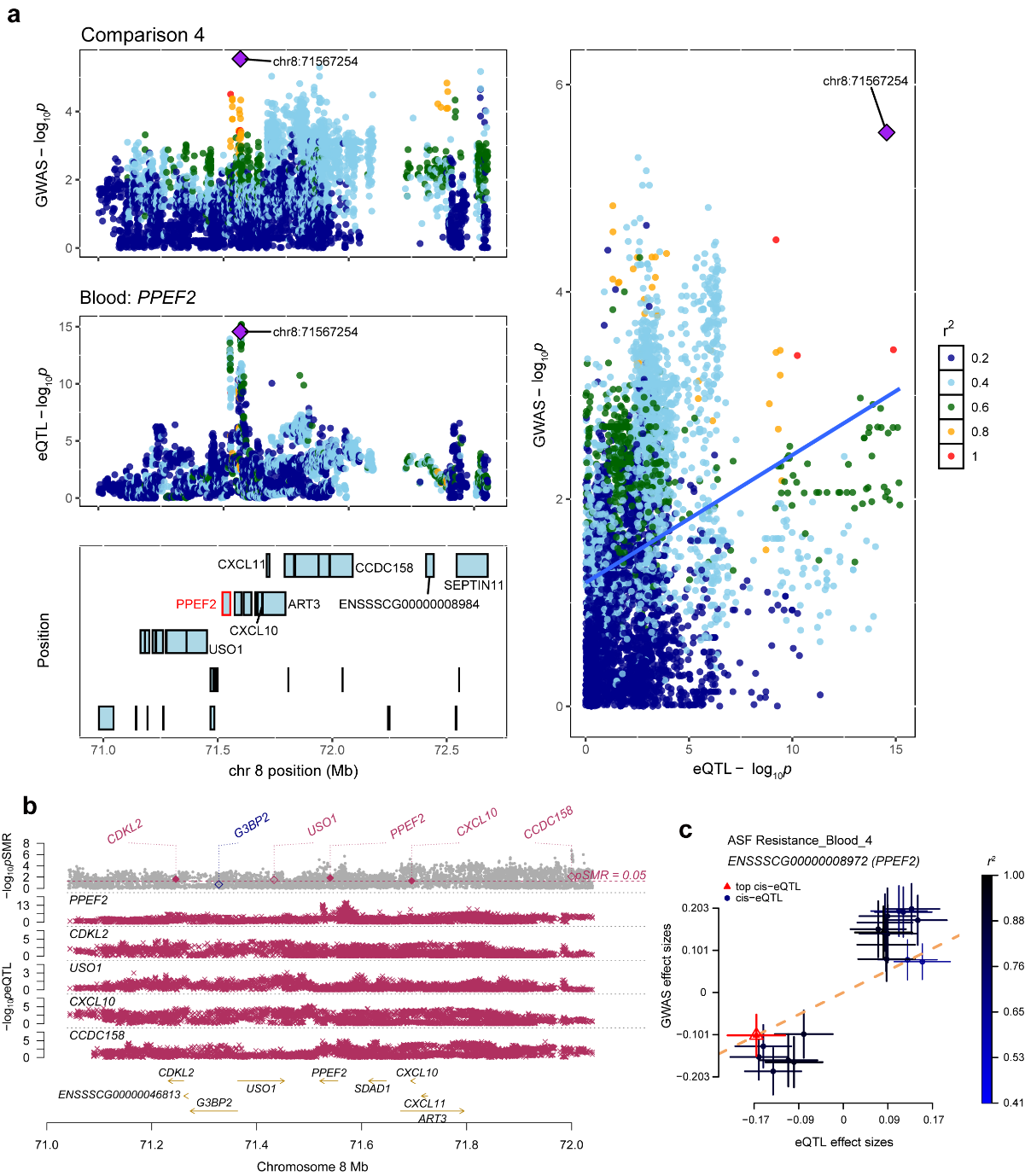


##### Supplementary Figure S4. Expression of *PPEF2* in blood and its role in ASF resistance.

**a.** Colocalization analysis of *PPEF2* eQTLs in blood with ASF resistance GWAS results.

**b.** SMR analysis of *PPEF2* eQTLs in blood with ASF resistance GWAS.

**c.** Positive correlation between *PPEF2* eQTLs in blood and ASF resistance GWAS signals.


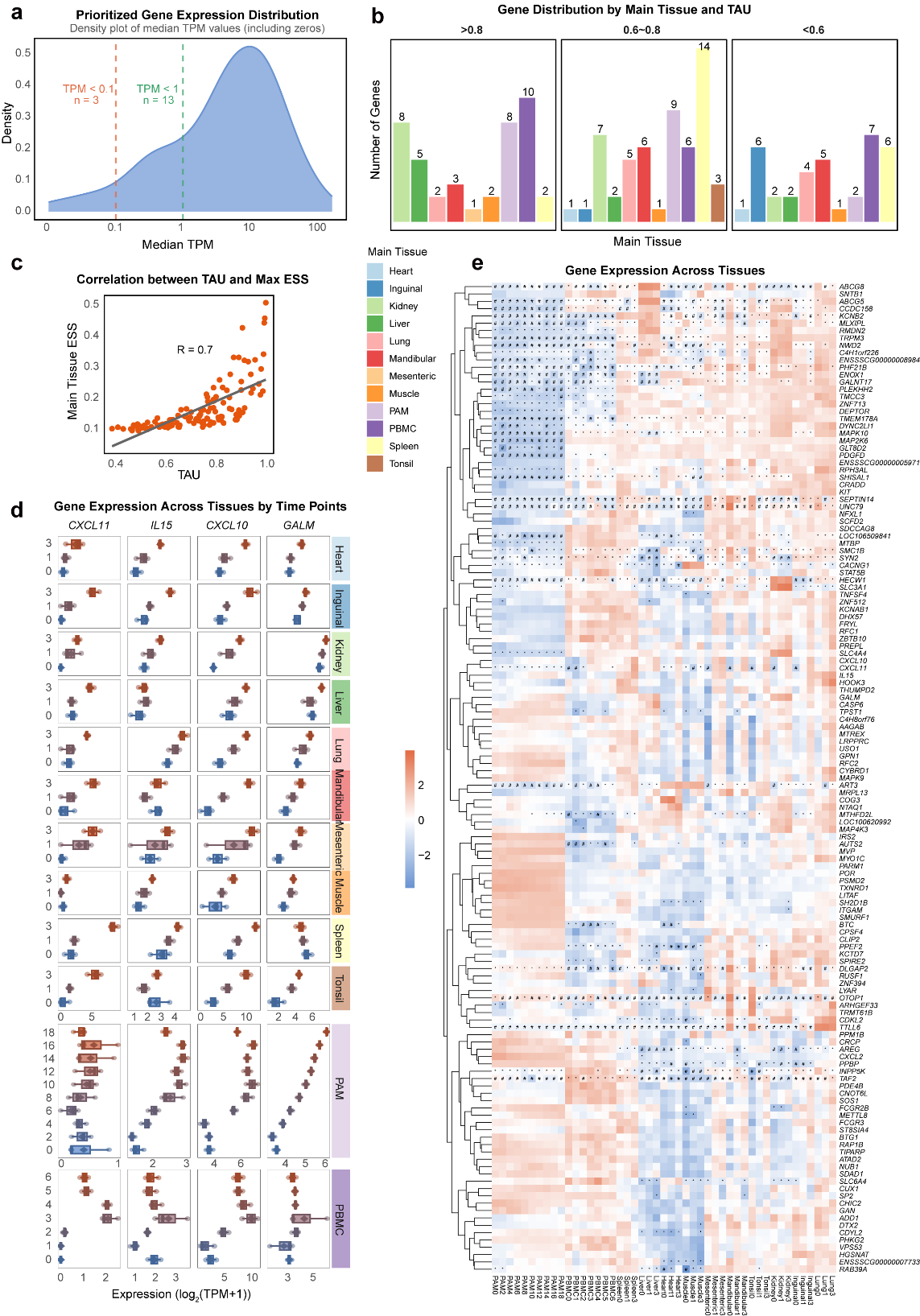


##### Supplementary Figure S5. Bulk RNA analysis of priority genes.

**a.** Expression distribution (TPM) of prioritized genes across tissues.
**b.** Distribution of TAU scores and the number of prioritized genes across major tissue types.
**c.** Correlation between TAU scores and maximum ESS scores.

**d.** Expression levels of *GALM*, *CXCL10*, *IL15*, and *CXCL11* across different tissues under various infection conditions.

**e.** Correlation between ASFV load (RPM) and host prioritized gene expression (TPM).
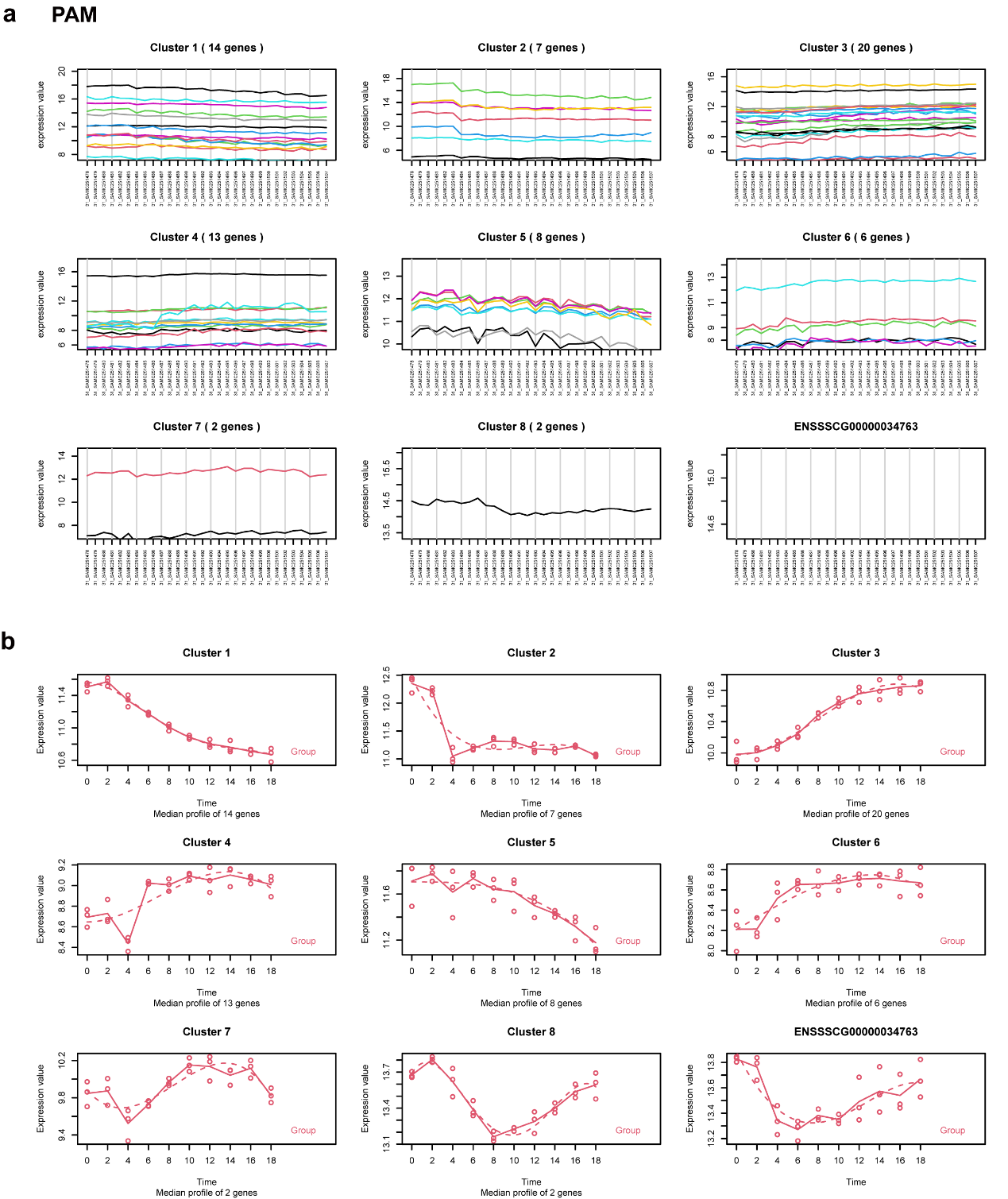


##### Supplementary Figure S6. Temporal expression analysis of priority genes in PAM.


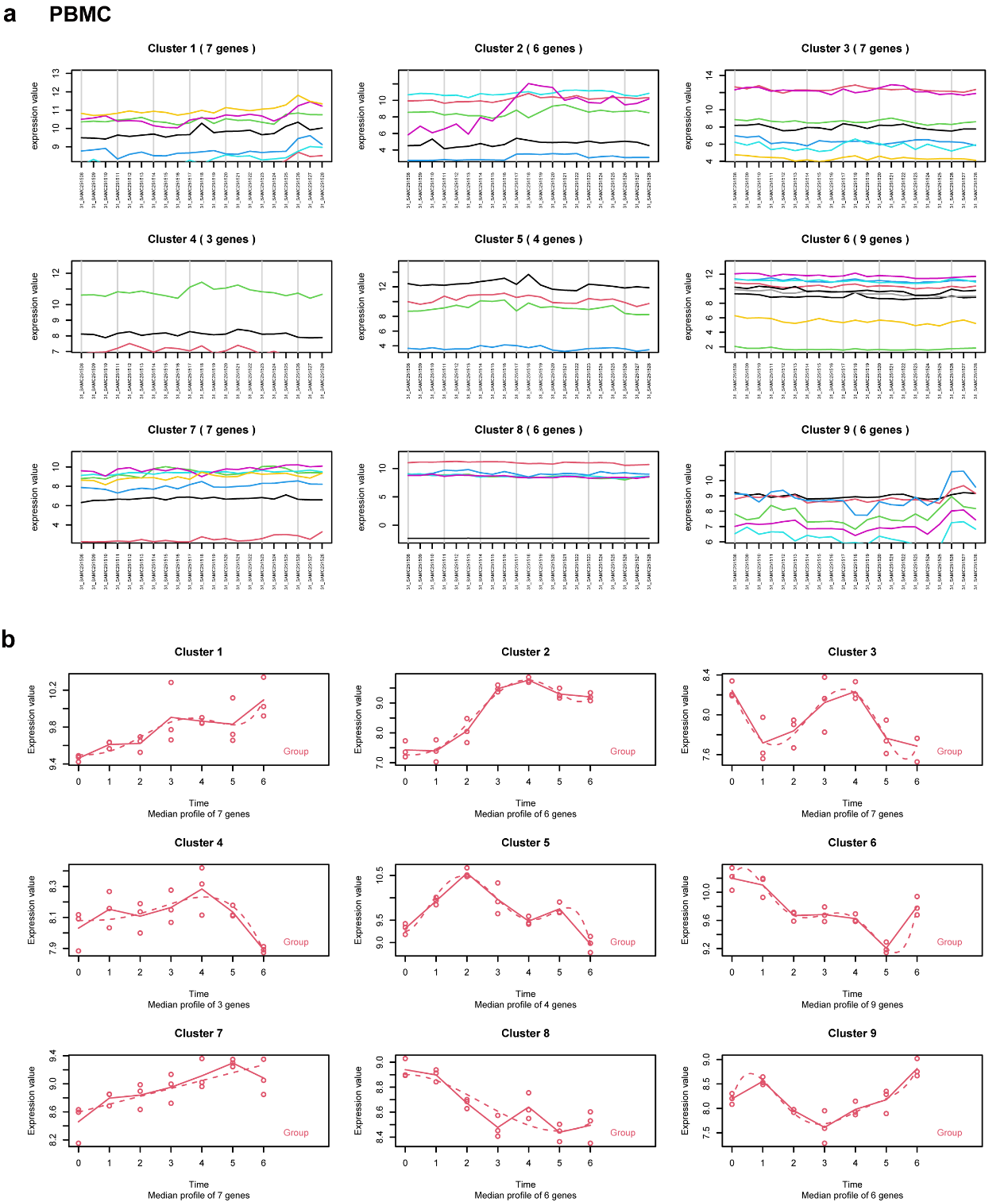


##### Supplementary Figure S7. Temporal expression analysis of priority genes in PBMC.


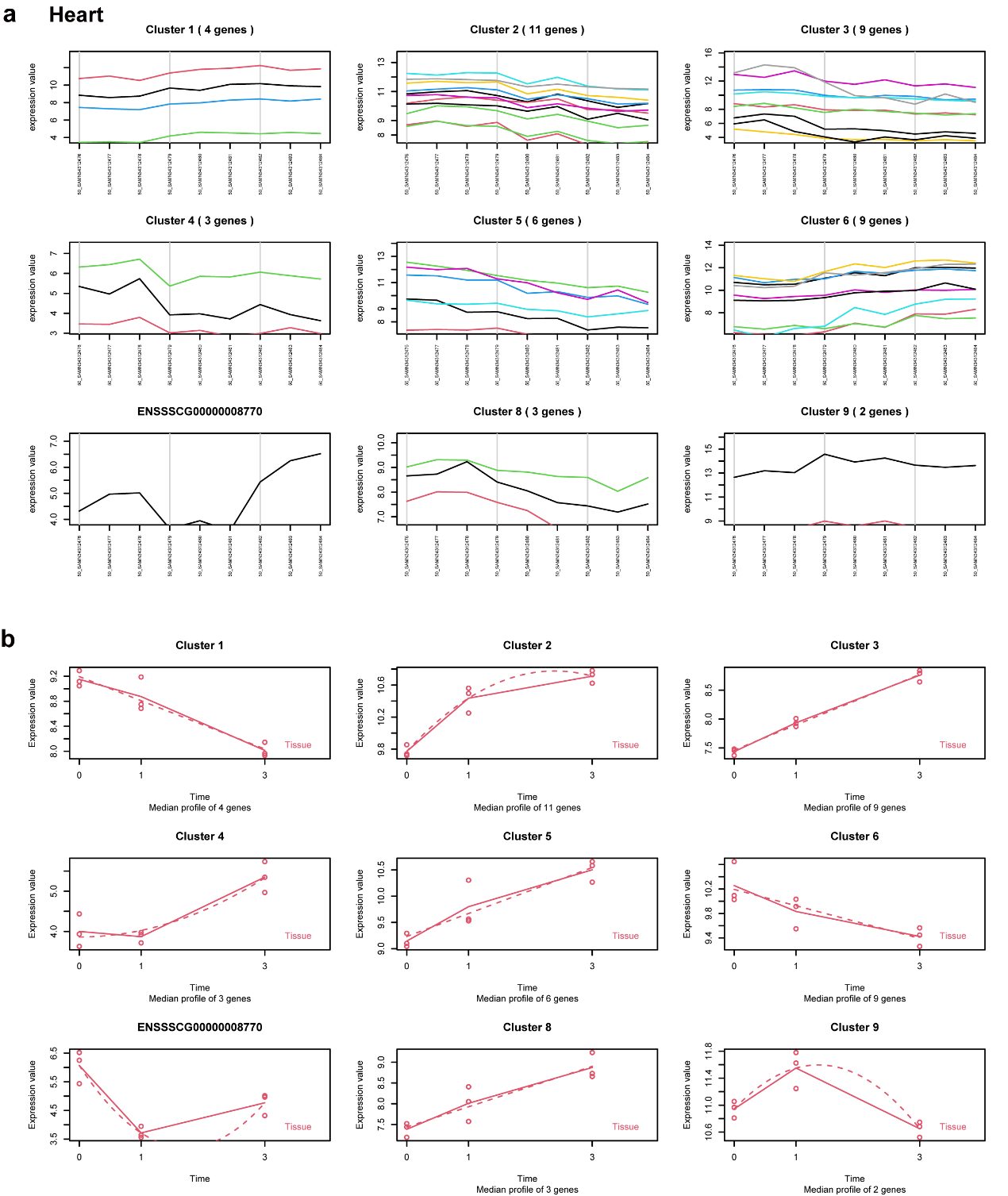


##### Supplementary Figure S8. Temporal expression analysis of priority genes in heart.


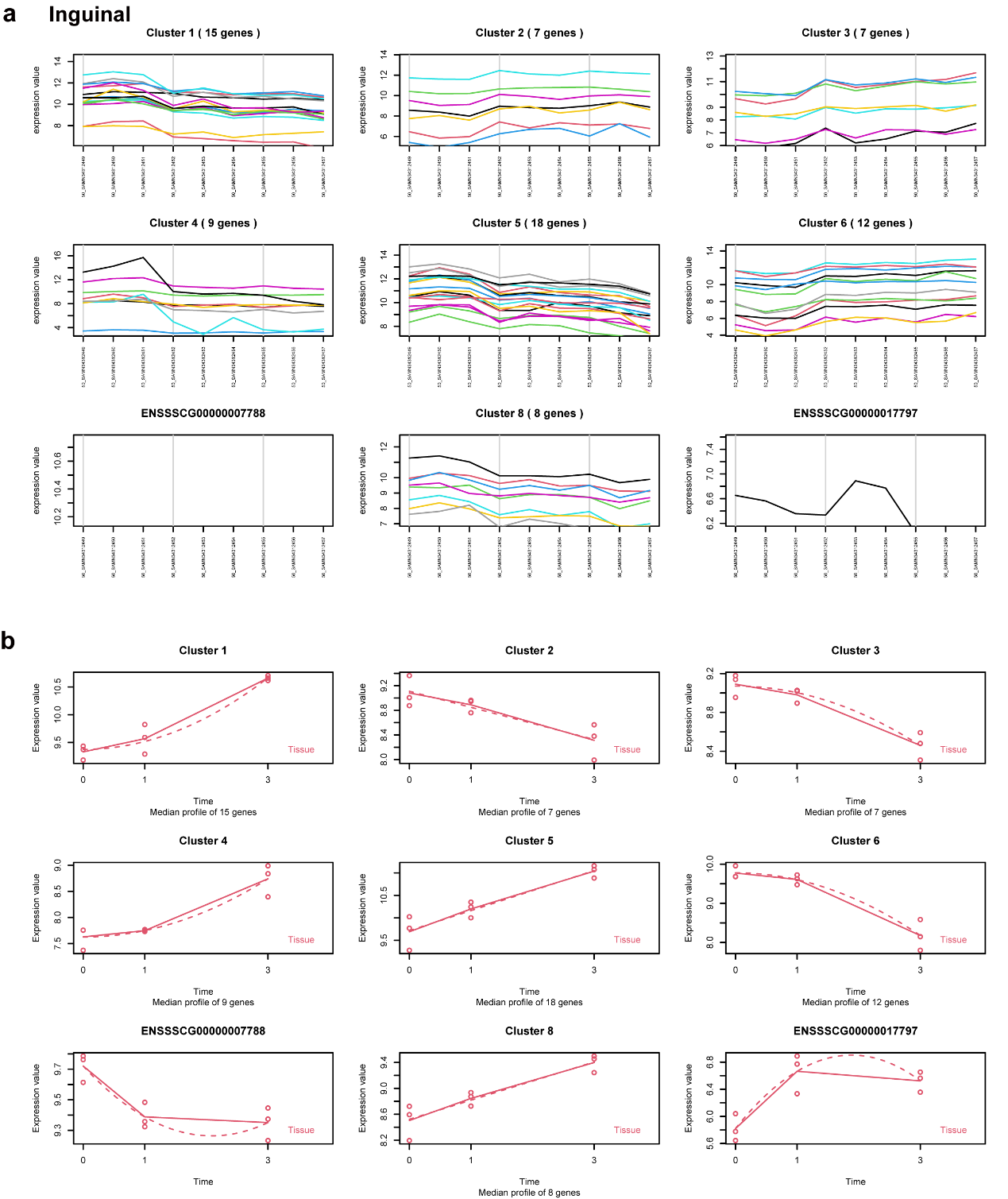


##### Supplementary Figure S9. Temporal expression analysis of priority genes in inguinal.


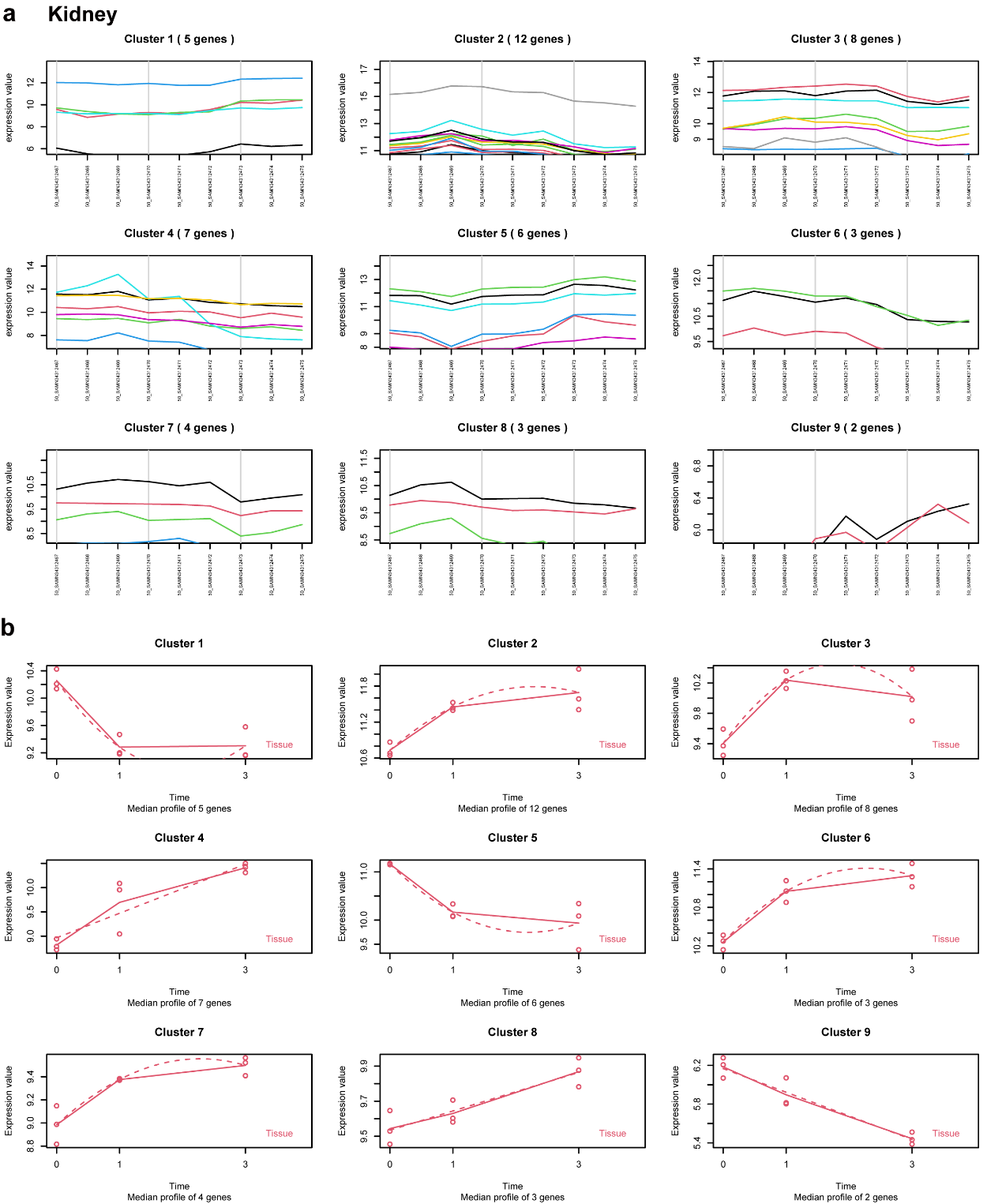


##### Supplementary Figure S10. Temporal expression analysis of priority genes in kidney.


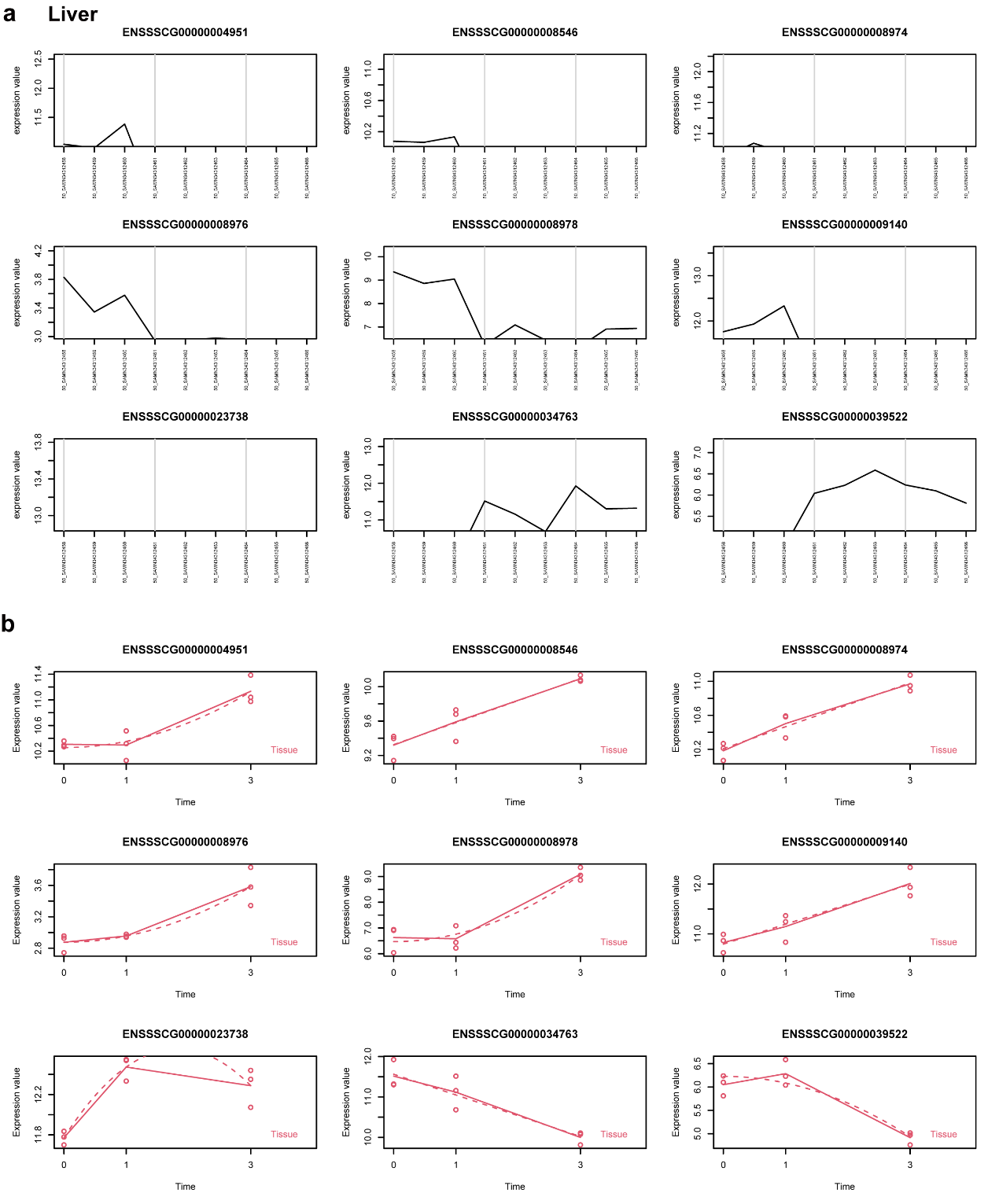


##### Supplementary Figure S11. Temporal expression analysis of priority genes in liver.


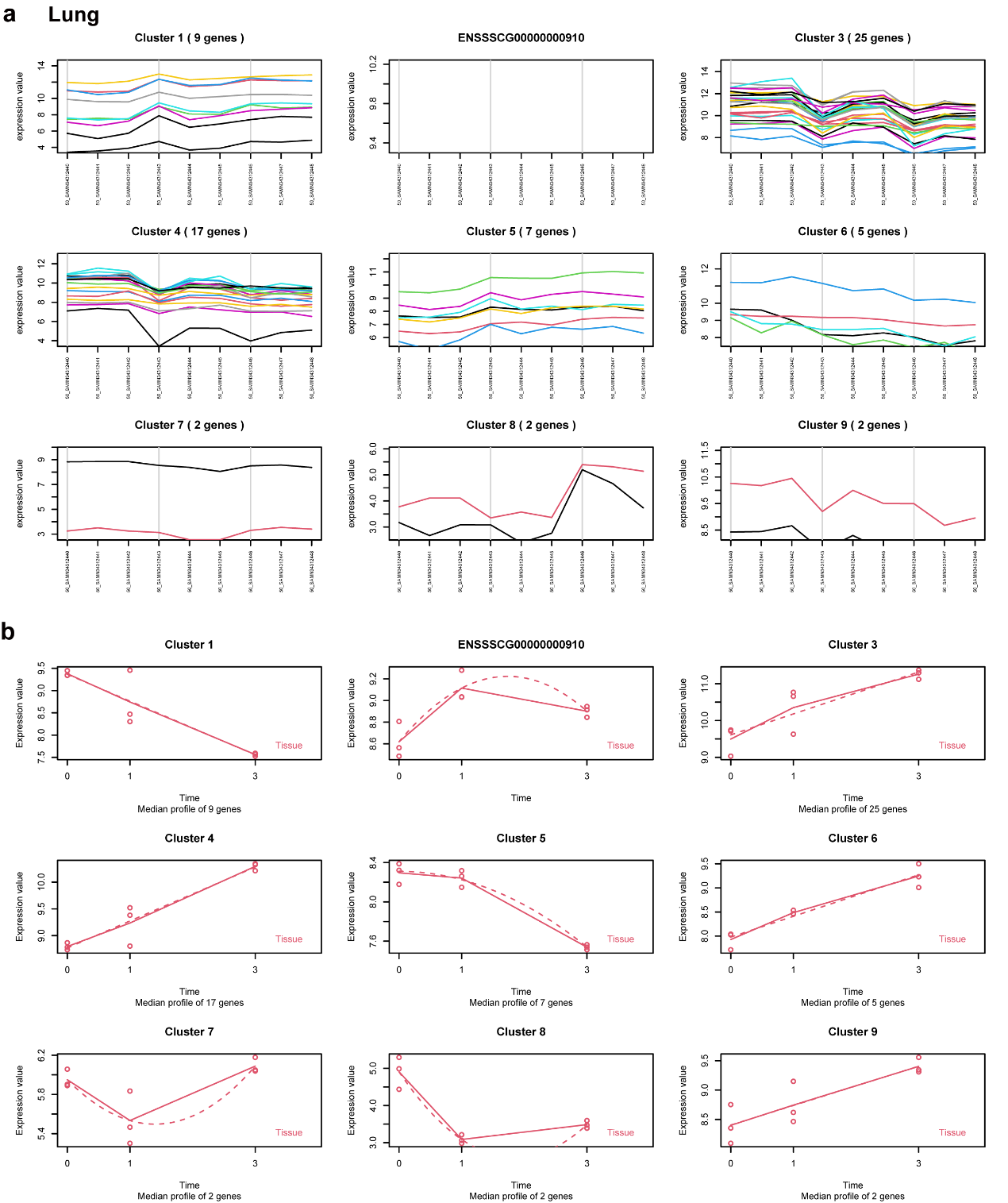


##### Supplementary Figure S12. Temporal expression analysis of priority genes in lung.


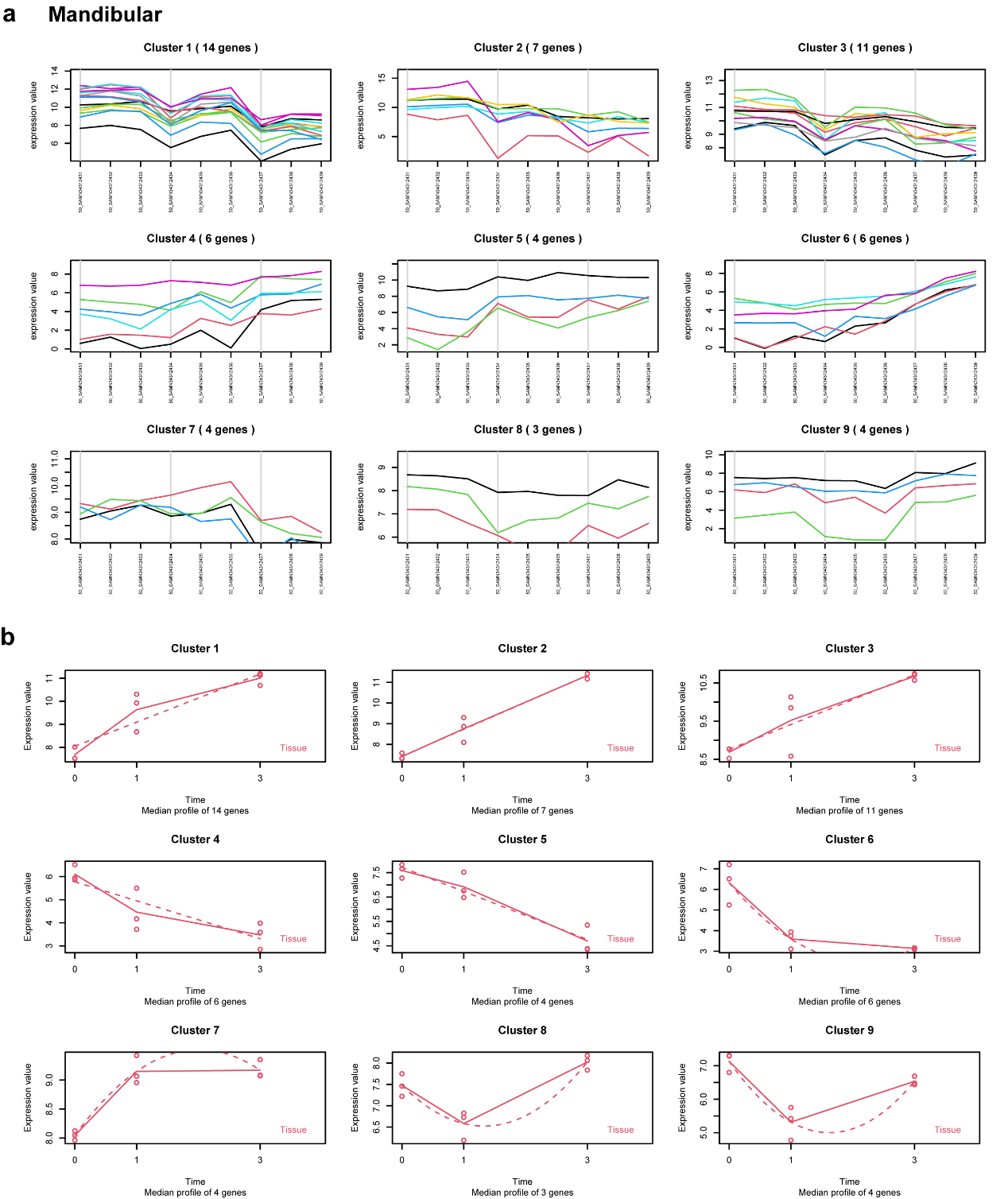


##### Supplementary Figure S13. Temporal expression analysis of priority genes in mandibular.


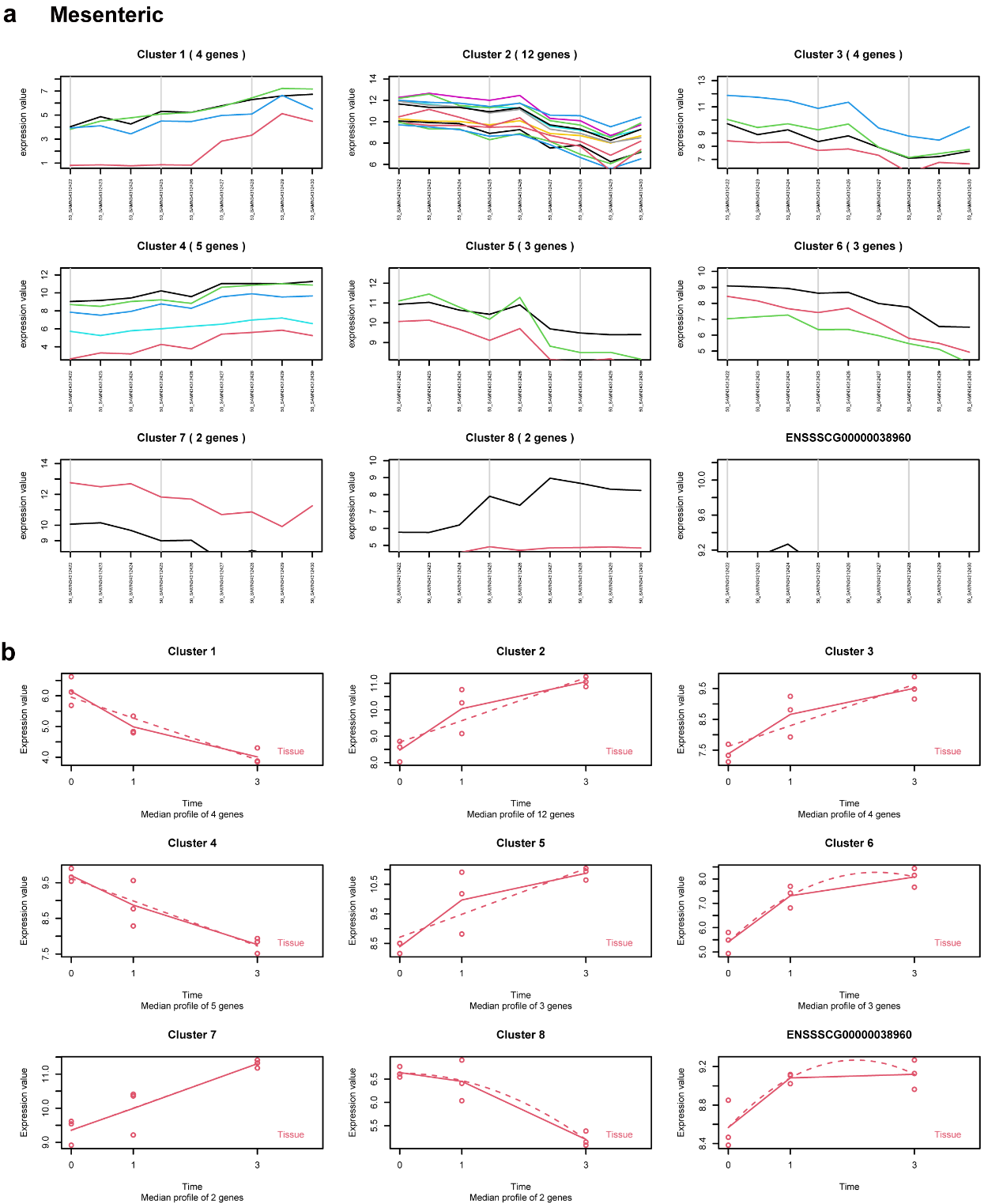


##### Supplementary Figure S14. Temporal expression analysis of priority genes in mesenteric.


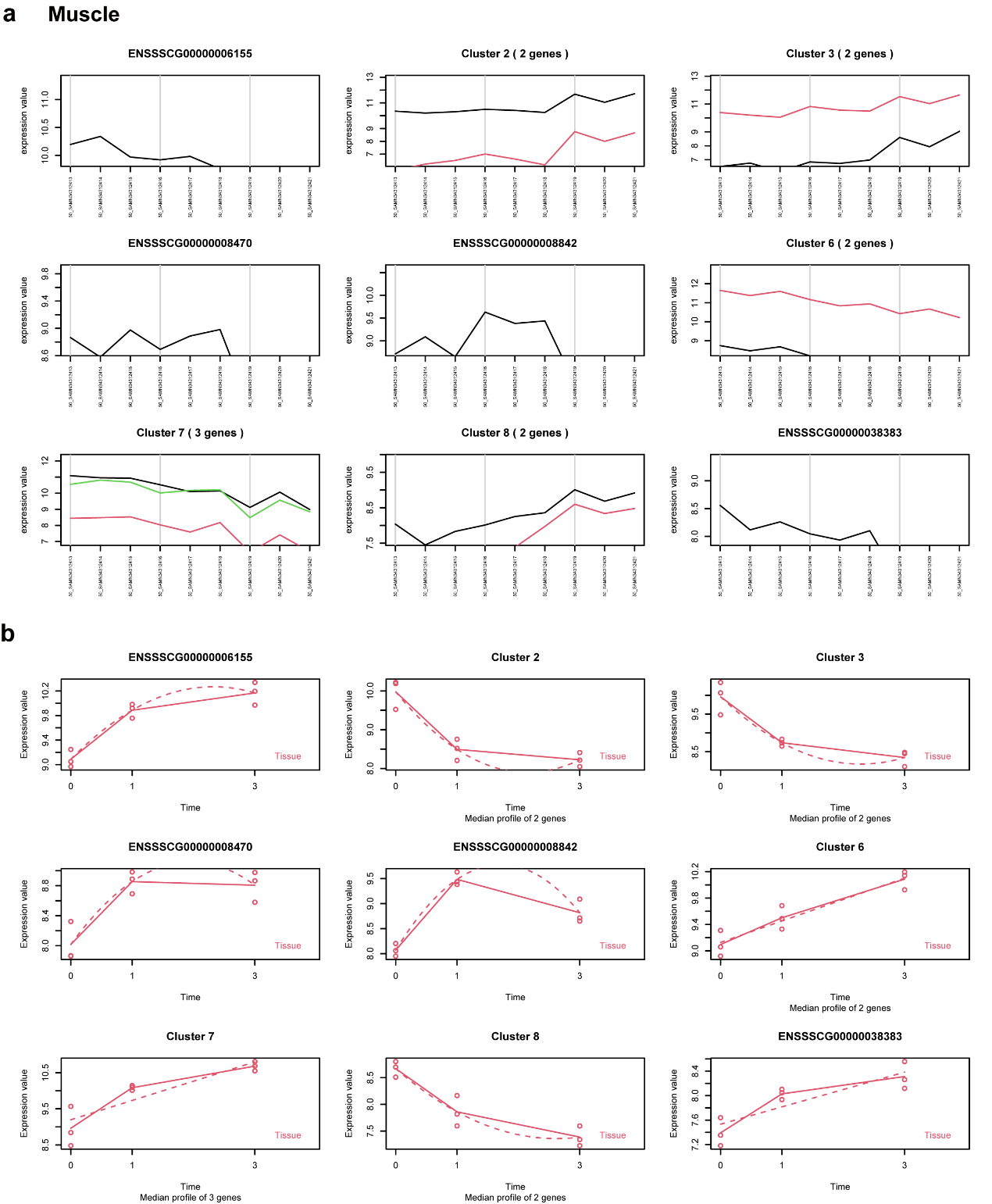


##### Supplementary Figure S15. Temporal expression analysis of priority genes in muscle.


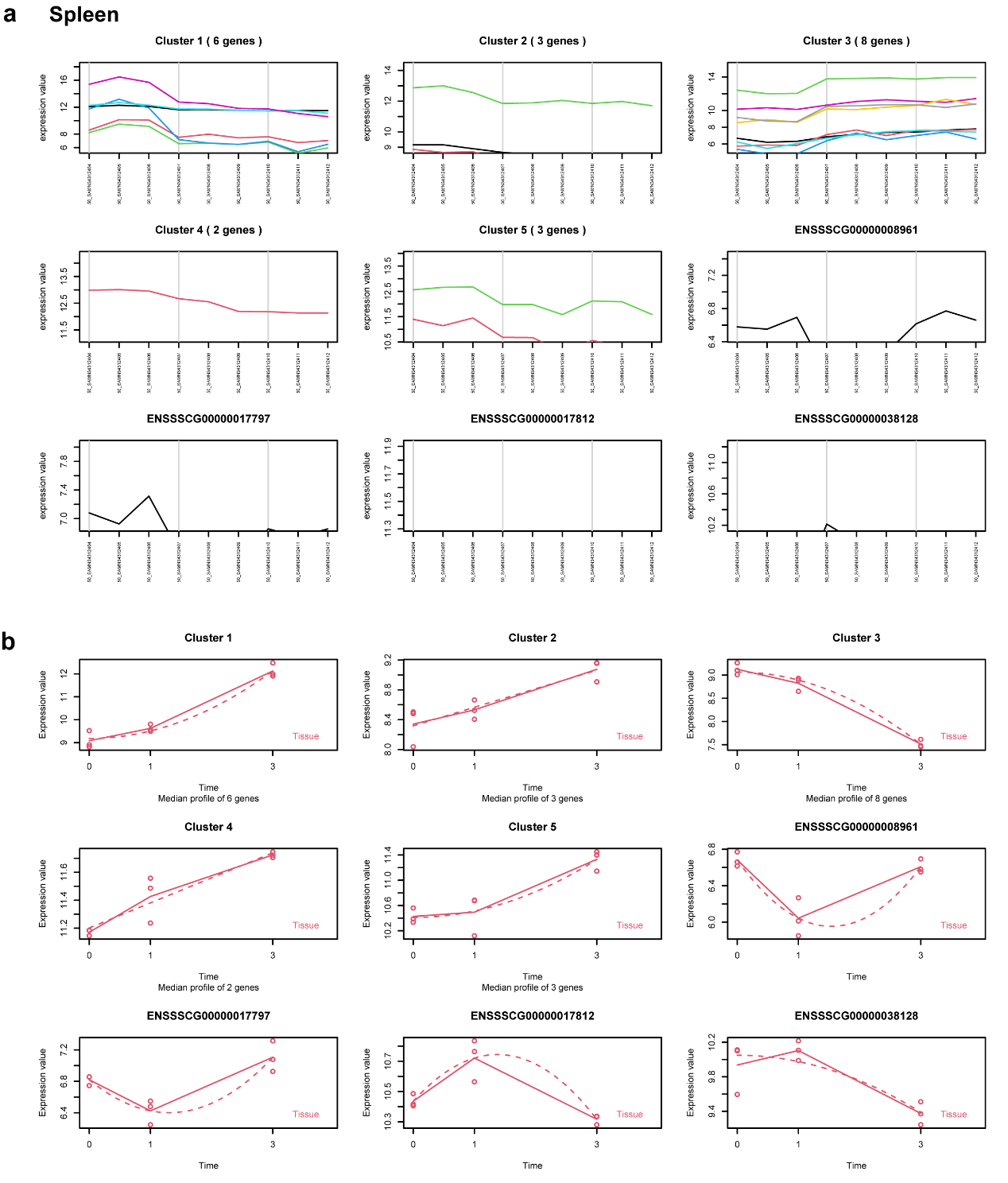


##### Supplementary Figure S16. Temporal expression analysis of priority genes in spleen.

**
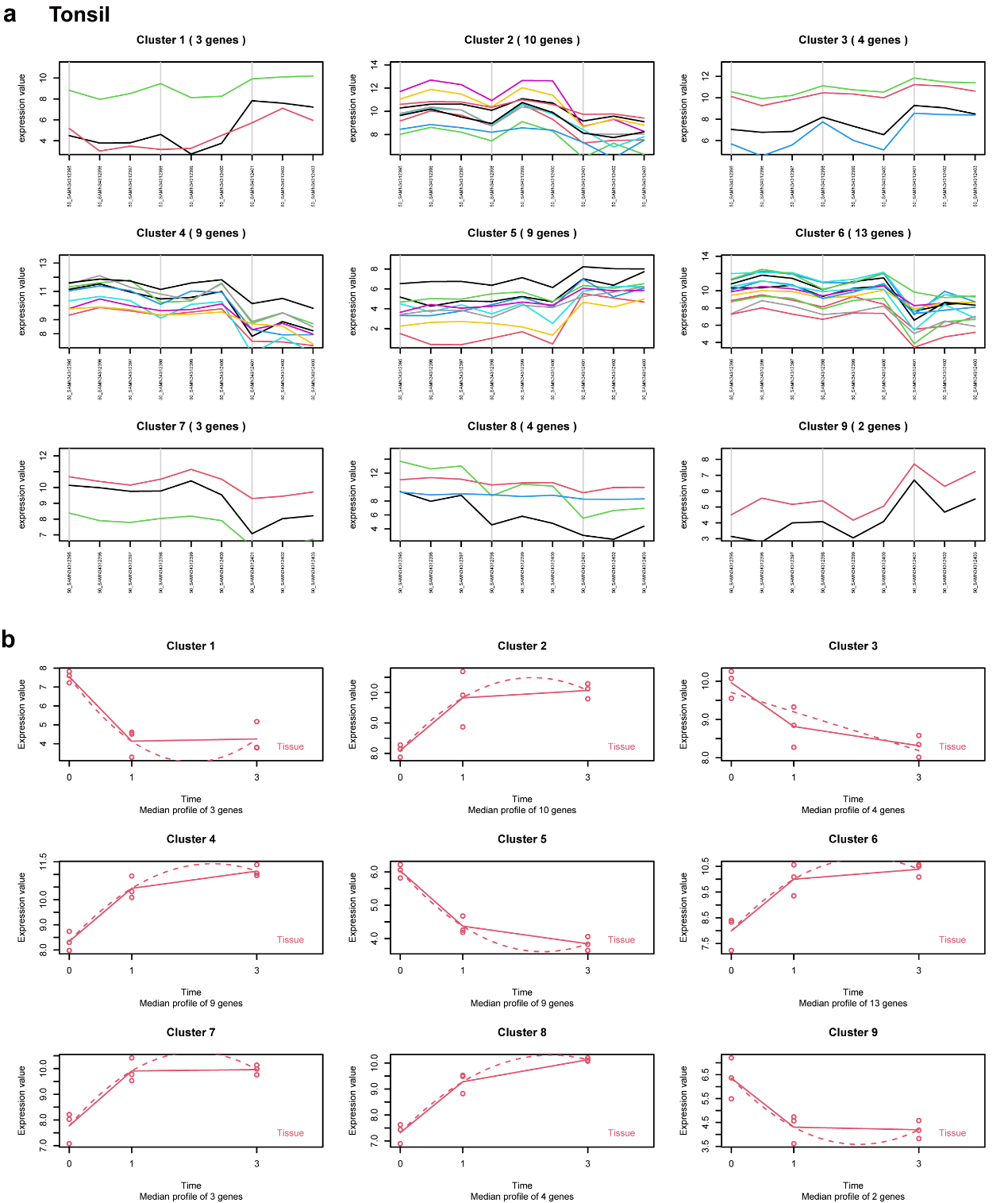
**

##### Supplementary Figure S17. Temporal expression analysis of priority genes in tonsil.


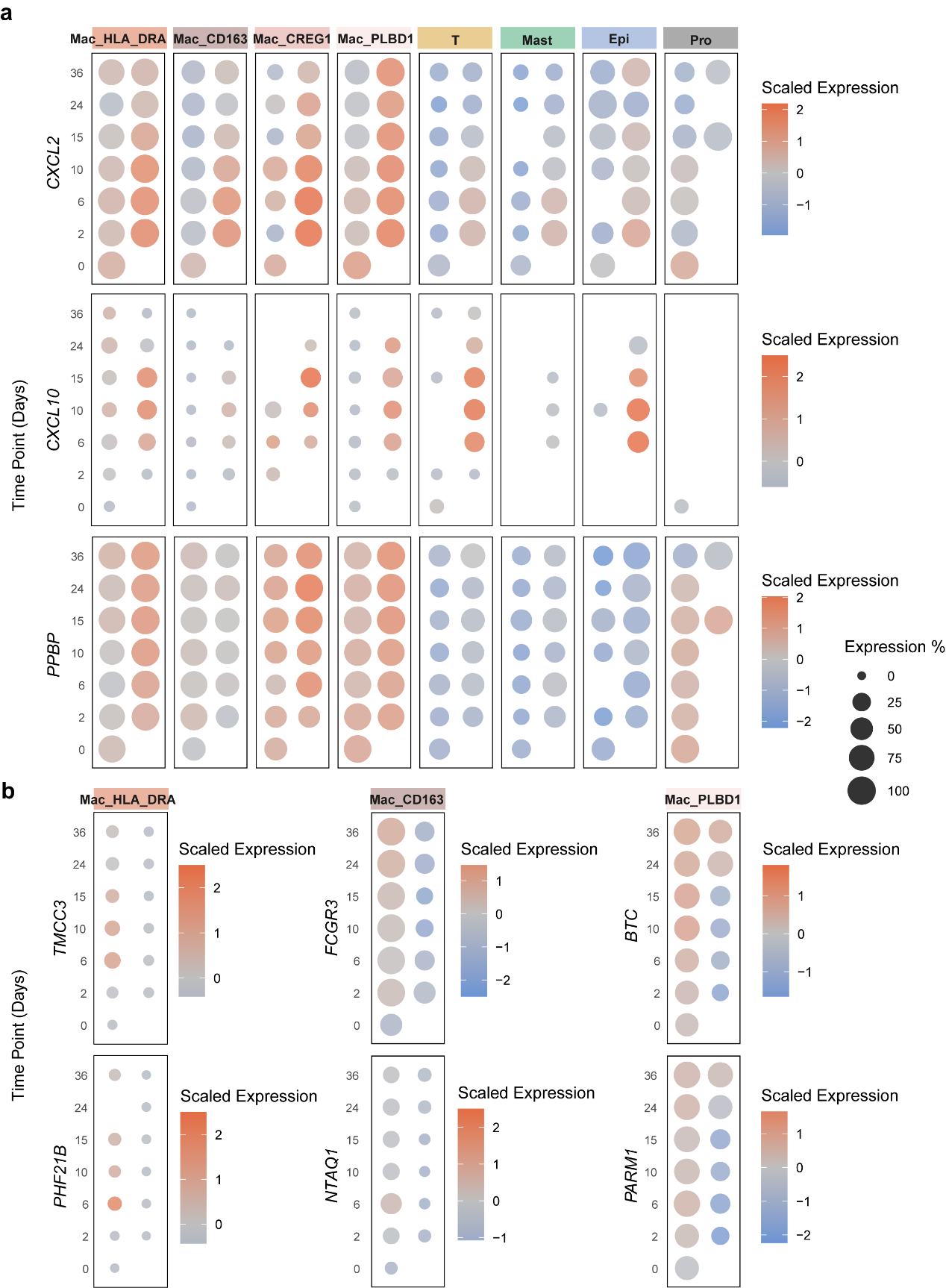


##### Supplementary Figure S18. Differential expression of prioritized genes in PAM subtypes under various infection states.


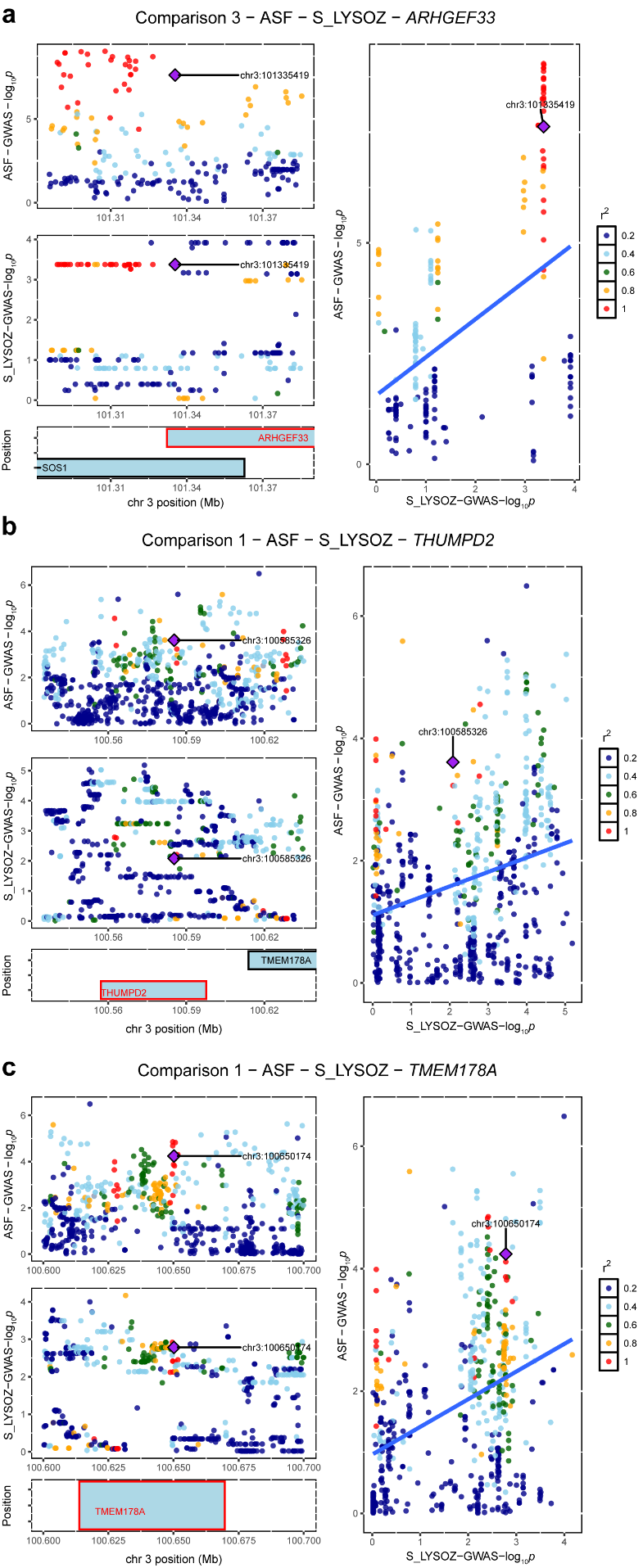


##### Supplementary Figure S19. Genes co-localized with ASF resistance and lysozyme levels.
