# Supplementary material for: Integrated multi-omics profiling identifies genetic loci of African swine fever resistance in pigs
Source: Gigascience. 2026 May 30;15:giag066. doi: 10.1093/gigascience/giag066 (PMC13289747; doi:10.1093/gigascience/giag066)
Supplement: giag066_GIGA-D-26-00031_original_submission [file giag066_giga-d-26-00031_original_submission.pdf]

GigaScience

Integrated multi-omics profiling identifies genetic loci of African swine fever resistance in pigs

--Manuscript Draft--

**Manuscript Number:**	GIGA-D-26-00031
**Full Title:**	Integrated multi-omics profiling identifies genetic loci of African swine fever resistance in pigs
**Article Type:**	Research
**Funding Information:**	Agricultural Variety Improvement Project of Shandong Province (2021LZGC001) — Prof. Zhen Wang<br>National Natural Science Foundation of China (31941007) — Prof. Yuchun Pan
**Abstract:**	African swine fever (ASF) remains a persistent threat to global pig production, with no licensed vaccines or effective treatments available. Observations of surviving individuals within infected herds suggest that host genetic resistance plays a crucial role. Here, we present a multi-dimensional integrative analysis to uncover host genomic variants associated with ASF resistance. Combining whole-genome sequencing, genome-wide association studies, selection scans, and functional genomic approaches, including TWAS, SMR, colocalization, and Bayesian network GWAS, we prioritized 135 high-confidence resistance genes from an initial gene set of 1,102 candidates. These prioritized genes are enriched in immune-related pathways, such as chemokine signaling and IL-15-mediated activation. Heritability enrichment and transcriptomic analyses further revealed tissue- and cell-type-specific expression patterns, particularly in peripheral immune organs and pulmonary alveolar macrophages. Dynamic infection-responsive genes, including CXCL10, CXCL11 and IL15, exhibited robust antiviral signatures, which highlighted Mac_CD163 as key cellular mediators in the immune response to ASF. Moreover, multiple ASF-resistance genes (such as SOS1, FCGR2B, FCGR3) converged on the PI3K-AKT and Fcγ receptor signaling axes pathways, underscoring their functional importance. Finally, we developed a polygenic resistance score using 40 functional SNPs, which effectively discriminates phenotypic outcomes and showed a positive correlation with health traits such as platelet distribution width. These findings provided a genomic foundation for the precision breeding of ASF-resistant pigs and inform host-targeted disease control strategies.
**Corresponding Author:**	ZHEN WANG<br>Zhejiang University<br>Hangzhou, Zhejiang CHINA
**Corresponding Author Secondary Information:**	
**Corresponding Author's Institution:**	Zhejiang University
**Corresponding Author's Secondary Institution:**	
**First Author:**	Xiaowei Ye
**First Author Secondary Information:**	
**Order of Authors:**	Xiaowei Ye<br>Qinqin Xie<br>Caiyun Cao<br>Shuang Liu<br>Wenbo Sun<br>Zhe Zhang

Powered by Editorial Manager® and ProduXion Manager® from Aries Systems Corporation

	Qishan Wang
	Yuchun Pan
	Zhen Wang
**Order of Authors Secondary Information:**	
**Additional Information:**	
**Question**	**Response**
Are you submitting this manuscript to a special series or article collection?	No
**Experimental design and statistics**<br><br>Full details of the experimental design and statistical methods used should be given in the Methods section, as detailed in our Minimum Standards Reporting Checklist. Information essential to interpreting the data presented should be made available in the figure legends.<br><br>Have you included all the information requested in your manuscript?	Yes
**Resources**<br><br>A description of all resources used, including antibodies, cell lines, animals and software tools, with enough information to allow them to be uniquely identified, should be included in the Methods section. Authors are strongly encouraged to cite Research Resource Identifiers (RRIDs) for antibodies, model organisms and tools, where possible.<br><br>Have you included the information requested as detailed in our Minimum Standards Reporting Checklist?	Yes
**Availability of data and materials**<br><br>All datasets and code on which the conclusions of the paper rely must be either included in your submission or deposited in publicly available repositories (where available and ethically	Yes

Powered by Editorial Manager® and ProduXion Manager® from Aries Systems Corporation

appropriate), referencing such data using a unique identifier in the references and in the "Availability of Data and Materials" section of your manuscript.<br><br>Have you have met the above requirement as detailed in our Minimum Standards Reporting Checklist?	
GigaScience has policies and guidelines in place for the use of generative AI-writing tools such as ChatGPT. If you have used such writing tools to assist with writing the manuscript this must be declared and cited in the text. Authors should not list AI-writing tools and other AI-assisted technologies as an author or co-author and should acknowledge that they are fully responsible for text generated or refined by AI-writing tools.<p><br><br>A summary of use (particularly in the introduction or among methods) needs to be included at the end of the paper, and the outputs should also be included as a supplementary file hosted in GigaDB or other open repositories. Please read our guidelines for more information. <p><br><br>By submitting to GigaScience, you are aware of the journal's AI-writing tools policy, and if you have declared use of such tools below, you have acknowledged this where appropriate in your manuscript and have made a summary of use and outputs available. <p><br><b>AI-assisted writing tools have been used in the preparation of this manuscript?	No

Manuscript Click here to access/download;Manuscript;Main.docx

Integrated multi-omics profiling identifies genetic loci of African swine fever resistance in pigs

Xiaowei Ye [a], Qinqin Xie [a], Caiyun Cao [a], Shuang Liu [a], Wenbo Sun [b], Zhe Zhang [a, c], Qishan Wang [a, c], Yuchun Pan [a, c, *], Zhen Wang [a, c, *]

[a] Zhejiang Key Laboratory of nutrition and breeding for high-quality animal products, College of Animal Sciences, Zhejiang University, Hangzhou, Zhejiang 310058, China
[b] Shandong Key Laboratory of Animal Disease Control and Breeding, Institute of Animal Science and Veterinary Medicine, Shandong Academy of Agricultural Sciences, Jinan, Shandong 250100, China
[c] Hainan Institute, Zhejiang University, Yongyou Industrial Park, Yazhou Bay Sci-Tech City, Sanya 572000, China

*Corresponding author: Zhen Wang (wangzhen20@zju.edu.cn) and Yuchun Pan (panyc@zju.edu.cn)

Short running title: Resistance loci for African swine fever

E-mail addresses:
XW. Y: ye_xw@zju.edu.cn [0009-0005-0079-3930]
CY. C: ccyun@zju.edu.cn
QQ. X: qinqin.xie@zju.edu.cn
S. L: liushuang9917@zju.edu.cn
WB. S: sunwenbo@saas.ac.cn
Z. Z: zhe_zhang@zju.edu.cn
QS. W: wangqishan@zju.edu.cn
YC. P: panyc@zju.edu.cn
Z. W: wangzhen20@zju.edu.cn [0000-0002-1896-3716]

Abstract

African swine fever (ASF) remains a persistent threat to global pig production, with no licensed vaccines or effective treatments available. Observations of surviving individuals within infected herds suggest that host genetic resistance plays a crucial role. Here, we present a multi-dimensional integrative analysis to uncover host genomic variants associated with ASF resistance. Combining whole-genome sequencing, genome-wide association studies, selection scans, and functional genomic approaches, including TWAS, SMR, colocalization, and Bayesian network GWAS, we prioritized 135 high-confidence resistance genes from an initial gene set of 1,102 candidates. These prioritized genes are enriched in immune-related pathways, such as chemokine signaling and IL-15-mediated activation. Heritability enrichment and transcriptomic analyses further revealed tissue- and cell-type-specific expression patterns, particularly in peripheral immune organs and pulmonary alveolar macrophages. Dynamic infection-responsive genes, including *CXCL10*, *CXCL11* and *IL15*, exhibited robust antiviral signatures, which highlighted Mac_CD163 as key cellular mediators in the immune response to ASF. Moreover, multiple ASF-resistance genes (such as *SOS1*, *FCGR2B*, *FCGR3*) converged on the PI3K-AKT and Fcγ receptor signaling axes pathways, underscoring their functional importance. Finally, we developed a polygenic resistance score using 40 functional SNPs, which effectively discriminates phenotypic outcomes and showed a positive correlation with health traits such as platelet distribution width. These findings provided a genomic foundation for the precision breeding of ASF-resistant pigs and inform host-targeted disease control strategies.

Keywords: African swine fever, multi-omics, disease resistance, pig breeding, host genetics

Introduction

African swine fever (ASF) is a highly contagious and often lethal viral disease that affects both domestic and wild pigs. Caused by the African swine fever virus (ASFV), the disease is characterized by severe hemorrhagic fever, with case fatality rates reaching nearly 100% in acute infections and 30–70% in subacute or chronic forms [1]. ASF outbreaks have caused devastating losses in swine populations worldwide, posing a substantial threat to global food security. Between 2005 and January 2025, ASF outbreaks were reported in 83 countries [2]. From 2014 to 2017, nearly 800,000 pigs in Eastern Europe and the Russian Federation were lost due to ASF [3]. By 2019, the disease had resulted in the culling or death of nearly 5 million pigs in Asia [4]. The economic repercussions of ASF have been staggering. Russia reported losses of $267 million during the 2011 outbreaks [5]. In 2022, ASF-related disruptions cost France's export market an estimated $168–389 million USD [6], while projections for 2023 suggested

potential losses of 2.5 billion USD in Australia [7] and up to 50 billion USD in the United States [8].

The emergence of ASF in China in 2018 had particularly profound effects on the national pig industry, given China's large-scale swine production [9,10]. With nearly half of the global pig population located in China [3,9,11], the outbreak led to dramatic reductions in herd sizes, severe disruptions in pork supply chains, loss of valuable genetic resources, and sharp increases in pork prices [11]. Within a single year (August 2018–July 2019), outbreaks of ASF in China resulted in economic losses exceeding 100 billion USD [12].

In response, considerable research efforts have focused on understanding ASFV biology, modes of transmission, and the development of effective vaccines and therapeutics [13–15]. However, ASF continues to be the most critical threat to the global pig industry. The absence of commercially licensed vaccines, the virus's ability to persist in diverse environmental reservoirs, and the high genetic variability across ASFV strains greatly complicate control efforts [16–19].

Intriguingly, field observations from recent outbreaks have revealed variable clinical outcomes among pigs within the same herd. While many individuals succumb to infection, some individuals survive and exhibit seroconversion without detectable viremia—testing negative for ASFV antigens but positive for ASFV-specific antibodies. Such findings suggest the existence of potential natural resistance mechanisms, drawing attention to host genetic factors as critical determinants of ASF susceptibility. This shift in focus—from a pathogen-centered to host-centered perspective—presents new possibilities for ASF control strategies. Genetic resistance traits, in particular, offer a sustainable and long-term strategy for ASF management, especially given the current lack of effective vaccines or antiviral treatments. Moreover, identifying and leveraging these traits could aid in the preservation of indigenous pig breeds, many of which are renowned for their natural resistance to disease.

To explore this hypothesis, we conducted an integrative genomic analysis combining whole-genome sequencing (WGS) of resistant and susceptible pigs, functional annotation of candidate variants, and transcriptomic profiling using publicly available RNA-seq datasets related to ASFV infection. The analysis leveraged resources from the FarmGTEx consortium, which provides a multi-species framework for transcriptomic and multi-omics profiling in livestock [20]. Within this initiative, PigGTEx [21] systematically characterizes gene expression across diverse tissues and developmental stages in pigs, while PigBiobank [22] extends these efforts by integrating large-scale phenotypic and omics data from multiple pig populations. Together, these datasets offer a comprehensive reference for investigating gene regulation and functional variation in

pigs, thereby supporting the identification of genomic variants, biological pathways, and candidate genes underlying resistance to ASF. Unlike previous studies, which primarily focused on association signals, our study bridges variant discovery with functional validation and predictive modeling, offering a more mechanistic understanding of ASF resistance. Our findings provide novel insights into the genetic architecture of ASF resistance, establishing a genomic foundation for selective breeding, genomic prediction, and potentially host-directed ASF control strategies. These results have broad implications for improving swine health and enhancing herd resilience against future ASF outbreaks.

Results

ASF-resistance candidate genes

We analyzed a genomic dataset comprising 474 individuals, retaining 23,290,599 common variants after quality control (minor allele frequency [MAF] > 0.05, **Table 1**). The experimental cohort displays an admixed genetic background, with ancestry predominantly derived from East Asian (specifically East Chinese) pigs, forming a unique cluster distinct from European commercial breeds (**Supplementary Fig. S1.** To maximize the identification of candidate loci associated with ASF-resistance, individuals were stratified into four groups for comparative analyses, each based on different potential gene-related objectives (**Fig.1**, **Tables 2** and **3**). Genome-wide association studies (GWAS) and selection signal analyses (fixaxion index, F_{ST}), combined with allele frequency tests (hereinafter referred to as the F_{ST} method), were employed to identify loci that may have undergone and contribute to ASF resistance. Ultimately, 1,102 non-redundant genes were identified as significantly associated with ASF resistance (GWAS: p-values < $\frac{1}{Me}$; F_{ST}: p-values < $\frac{0.05}{N}$) (**Fig. 2**, **Supplementary Fig. S2**, **Supplementary Tables S2** and **S3**).

ASF-resistance gene prioritization

To prioritize genes associated with ASF resistance, we assessed the functional evidence for 1,102 candidate genes by integrating five independent validation methods to assign prioritization scores: reported immune genes, transcriptome-wide association studies (TWAS), summary-data-based Mendelian randomization (SMR), colocalization, and Bayesian network genome-wide association studies (BN-GWAS) (see **Methods**, **Table 4**). As a result, 135 high-priority genes were identified based on prioritization scores in the top 10% (⩾3.5) or validation by at least two methods (**Fig. 3a** and **Supplementary Table S4**). Among these high-priority genes, 31 were previously implicated in immune functions, such as members of the chemokine family (*CXCL2*, *CXCL7*, *CXCL10*, *CXCL11*) [23] and the MAP kinase family (*MAP4K3*, *MAPK9*, *MAP2K6*, *MAPK10*) [24],

which are integral to immune regulation, inflammatory responses, and cell signaling. Additionally, *IL15* was highlighted for its roles in T and NK cell activation and maintenance of memory CD8$^+$ T cells [25], emphasizing its potential involvement in ASF resistance.

Tissue-specific associations via TWAS

TWAS analysis revealed significant tissue-specific associations for 11 prioritized genes across muscle, liver, blood, and intestinal tissues (FDR < 0.05). Notably, *SOS1*, *GAN*, *ZNF394*, *KIT*, and *TMCC3* exhibited positive associations in muscle and liver tissues, while *ZNF713*, *RUSF1*, *KIT*, *PARM1*, *PHF21B*, *SLC3A1*, and *BTC* showed negative associations in various tissues including the blood and intestine (**Fig. 3c**, **Supplementary Fig. S3** and **Supplementary Table S7**). These findings highlight the tissue-dependent genetic mechanisms that may contribute to the host's resistance to ASF, providing valuable targets for further functional studies and breeding programs.

Causal relationships identification via SMR and colocalization

Using SMR analysis across 34 tissues from PigGTEx [21], we identified 1,490 candidate causal pairs involving 316 candidate genes, with 127 classified as prioritized (**Supplementary Table S6** and **S8**). Colocalization analysis further validated 42 of these pairs, confirming the presence of shared causal variants associated with ASF resistance (**Fig. 3d**). For example, the *PPEF2* gene in blood was found to share a causal variant with ASF resistance (**Supplementary Fig. S4**). Functionally, *PPEF2* inhibits ASK1, a MAP kinase involved in apoptosis regulation, and modulates CD8$^+$ cDC1 antigen presentation [26,27], implicating it in immune processes relevant to ASF resistance. Additionally, 41 pairs showed strong posterior probabilities (>75%) under Hypothesis 3, such as *SOS1* (muscle), *SLC3A1* (muscle), *RUSF1* (multiple tissues), *GALM* (milk, adipose), *CXCL11* (liver), and *MVP* (blood, adipose), supporting associations with distinct causal variants (**Fig. 3d** and **Supplementary Table S9**).

Causal effects of ASF-resistance gene validating via BN-GWAS analysis

BN-GWAS [28] was employed to infer causal networks linking candidate genes to ASF resistance phenotypes. Using RNA-seq data from five tissues (muscle, blood, brain, embryo, and liver) with sample sizes exceeding 300, we identified 12 prioritized genes with direct or indirect causal effects (**Fig. 3e** and **Supplementary Table S10**). Notable genes include *SOS1* (muscle), *FCGR2B* (brain) and *SDCCAG8* (muscle), which showed positive causal effects, and *HGSNAT* (brain), *LOC106509841* (blood), and *CLIP2* (blood), which exhibited negative effects.

Integration and validation of prioritized genes

Among all prioritized genes, *SOS1* achieved the highest prioritization score (13.7), validated by all five methods. As a regulator of the MAPK, PI3K/JAK cellular signaling pathways and tumorigenesis [29,30], *SOS1* was identified as having a positive effect on ASF resistance. Additionally, 13 other genes (*RUSF1*, *KIT*, *ZNF713*, *FCGR2B*, *SDAD1*, *MVP*, *CLIP2*, *SLC3A1*, *HGSNAT*, *PHF21B*, *ZNF394*, *CXCL11*, and *SDCCAG8*) were supported by three validation methods, highlighting their robustness (**Fig. 3a**). Notably, *SOS1* (muscle), *RUSF1* (blood, liver, lung), *SLC3A1*(muscle), *ZNF394* (liver) and *FCGR2B* (brain) showed consistent tissue-specific activity.

Enriched pathways of prioritized genes

Pathway enrichment analysis of the prioritized genes revealed 41 immune-related pathways significantly associated with ASF resistance (FDR < 0.05) (**Fig. 3f** and **Supplementary Table S11**). Notably, pathways such as CXCR chemokine receptor binding (FDR = 1.7×10^{-3}), MAP kinase activity (FDR = 2.3×10^{-2}), and interleukin-15 signaling (FDR = 3.2×10^{-2}) were among the most significantly enriched. These pathways, previously implicated immune gene prioritization, underscore the critical roles of chemokine signaling, MAP kinases, and IL-15 in mediating ASF resistance. Additionally, signaling by KIT in disease (FDR = 4.9×10^{-2}) and lymphocyte number (FDR= 7.7×10^{-15}) emerged as key pathways potentially driving immune responses against ASF. These findings reinforce the involvement of specific immune signaling mechanisms in ASF resistance.

Resistance-associated tissues and cells

To identify tissues and cells associated with ASF resistance, we conducted heritability enrichment analysis across 34 tissues and 8 porcine alveolar macrophage (PAM) subtypes using linkage disequilibrium (LD) score regression. Significant enrichment was observed in PAM subtypes (Mac_CD163 and Mac_PLBD1) and the small intestine (including jejunum and ileum) (**Fig. 4a** and **Supplementary Table S12**). As the primary target of ASF, PAMs play a central role in infection [31], while the intestine is vital for mucosal immunity and host-microbiota interactions that may influence viral attachment and invasion [32]. These results align with established ASF infection mechanisms [31,33]. Interestingly, enrichment in non-traditional tissues such as the brain, heart, and milk suggested a potential cross-tissue resistance network, highlighting broader systemic contributions to ASF resistance beyond primary immune tissues.

Spatial transcriptomic mapping of prioritized ASF-resistance genes in mouse embryos at embryonic day 16.5 [34] (comprising 115 prioritized genes) revealed widespread gene expression across various tissues (**Fig. 4b**), mirroring the systemic lesions observed during ASF infection in pigs [33,35] .This underscores the potential involvement of these

genes in systemic immune responses.

RNA-seq analysis from ASFV-infected and healthy pig tissues retained 132 prioritized genes after basal expression filtering (TPM > 0.1 in ⩾20% of samples), revealing tissue-specific gene expression patterns, with 41 genes (31.1%) exhibited high tissue specificity (TAU > 0.8) in peripheral blood mononuclear cells (PBMCs), kidney, and PAM, while 36 genes (27.3%) showed broad expression across multiple tissues ((**Fig. 4c**, **Supplementary Fig. S5** and **Supplementary Table S13**), suggesting a general role in systemic immune and inflammatory responses. Moreover, differential gene expression and time-series analyses revealed 127 prioritized genes were significantly differentially expressed (FDR < 0.05, $|\log_2 FC| > 1$) in at least one tissue at various time points post-ASFV infection (**Fig. 4c**). The mandibular (90 genes), tonsil (78 genes), and mesenteric (76 genes) tissues harbored the highest number of responsive genes, consistent with their central roles in lymphoid immune responses [36] (**Supplementary Tables S13** and **S14**). Prioritized genes such as *GALM*, *CXCL11*, *CXCL10,* and *IL15* were consistently upregulated across multiple tissues (**Fig. 4c**, **Supplementary Fig. S5**, **Supplementary Tables S13** and **S14**), indicating a sustained antiviral immunity activation during ASFV progression. Conversely, genes like *ADD1*, *CLIP2*, *PHF21B* were downregulated (**Fig. 4c**, **Supplementary Tables S13** and **S14**), potentially reflecting virus-induced suppression of host immune pathways. Notably, *ADD1* plays a critical role in vesicle packaging and viral endocytosis, with its depletion demonstrating antiviral effects [37].

The dynamics of ASFV replication, as quantified by viral RNA (RPM), were both tissue-specific and time-dependent. While all tissues supported progressive viral replication, PAM were distinguished by the earliest and most rapid replication, showing significant accumulation by 4 hours post-infection (hpi). A widespread, marked increase in other tissues became evident by 3 days post-infection (dpi). Spatially, viral abundance was stratified, with the highest titers (≈20,000 RPM) in primary target tissues (PAM, spleen, PBMCs). The lung displayed intermediate levels (≈9,000 RPM), while the heart and various lymph nodes (inguinal, mandibular, mesenteric) exhibited the lowest levels, at several hundred to ~1,000 RPM (**Fig. 5a**). To explore host-pathogen interactions, a Spearman correlation was performed, identifying positive associations between viral RPM and the transcriptional expression (TPM) of *GALM*, *CXCL10*, *IL15*, and *CXCL11* in several tissues (**Fig. 5b**).

Single-cell RNA-seq analysis in PAMs [31] (target cells for ASFV) in identified 127 prioritized genes in various celltypes post-infection. The Mac_CD163 cells, a transcriptionally distinct PAM subpopulation characterized by high baseline expression of *CD163*, *MARCO*, *S100A8*, and *S100A9* [31], exhibited sustained upregulation of the prioritized genes throughout ASFV infection (**Fig. 4e** and **Supplementary Table S16**).

This persistent response highlights their specialized role in orchestrating innate immune defenses within the lung, consistent with prior functional descriptions of this subset [31]. Other subtypes of macrophage cells, such as Mac_HLA_DRA, Mac_CREG1 and Mac_PLBD1 also show high expression of prioritized genes early in the infection (**Fig. 4e** and and **Supplementary Table S16**). Differential expression analysis further revealed that 68 prioritized genes were significantly differentially expressed in at least one cell type at specific time points (**Fig. 3f**), emphasizing their dynamic roles in ASFV infection and immune modulation. For examples, gene *CXCL2*, highly upregulated in the early phase; gene *CXCL10*, peaking during the mid-phase; and gene *PPBP* (also known as *CXCL7*), predominantly upregulated in the mid-to-late phase, across multiple immune cell populations (**Fig. 4f**, **Supplementary Fig. S18** and **Supplementary Table S17**). Conversely, genes such as *TMCC3* (Mac_HLA_DRA), *PHF21B* (Mac_HLA_DRA), *FCGR3* (Mac_CD163), *NTAQ1* (Mac_CD163), *BTC* (Mac_PLBD1) and *PARM1* (Mac_PLBD1) (**Fig. 4f**, **Supplementary Fig. S18** and **Supplementary Table S17**) exhibited consistent downregulation, potentially reflecting virus-induced suppression mechanisms targeting host immune pathways.

Genetic correlations of ASF resistance with other traits

We assessed the genetic correlations between ASF resistance and 122 traits related to health, growth, and reproduction using publicly available GWAS summary data (121 traits from PigBiobank [22] and one trait concerning Mycoplasmal Pneumonia of Swine, MPS [38]). Notably, ASF resistance showed significant correlations with specific hematological parameters: a positive correlation with platelet distribution width (S_PLDWID) and a negative correlation with red cell distribution width (S_RCDW, p-value < 0.05, **Fig. 6a** and **Supplementary Table S18**). The positive correlation with S_PLDWID suggests that genetic mechanisms influencing platelet size variability are shared with those conferring ASF resistance. As a marker of platelet size variability, S_PLDWID is linked to immune activation and systemic inflammation, processes that may enhance resilience against ASF infection [39]. Conversely, the negative correlation with S_RCDW, an indicator of erythrocyte size variability, implies a connection between stable red cell morphology and reduced susceptibility to inflammation-induced damage during ASF infection [40]. Lower S_RCDW may reflect diminished oxidative stress and a more regulated inflammatory response, contributing to ASF resistance. These findings identify S_PLDWID and S_RCDW as potential phenotypic markers for breeding strategies aimed at enhancing ASF resistance while maintaining overall health and performance in pig populations. Incorporating these markers into selective breeding programs could facilitate the development of ASF-resistant breeds, thereby supporting sustainable disease management and improving animal welfare.

Pleiotropic associations of prioritized genes

To explore the pleiotropic effects of ASF resistance-prioritized genes, we analyzed their effects with 299 traits derived from 298 PigBiobank GWAS studies [22] and one trait related to MPS [38]. This analysis identified 1,151 gene-trait pairs across 286 GWAS studies, involving 133 prioritized genes (**Supplementary Table S19**). Notably, 12 traits exhibited significant enrichment, including four health-related traits: lysozyme levels (S_LYSOZ), granulocyte phagocytosis (S_GRANP), blood albumin level (S_ALBU), and low-density lipoprotein (S_LDL) (**Fig. 6b** and **Supplementary Table S20**). These traits are associated with systemic immunity and inflammation, suggesting shared genetic regions between ASF resistance and immune responses.

Cross-trait colocalization analysis revealed shared causal variants (PP4 > 0.7) for ASF resistance and lysozyme levels in genes such as *TMEM178A*, *ARHGEF33*, and *THUMPD2*, suggesting their involvement in antiviral defense mechanisms (**Fig. 6c** and **Supplementary Table. S21**). Conversely, distinct causal variants (PP3 > 0.7) were identified for traits like $CD4^+$ leukocyte (*GLT8D2*, *TXNRD1*), platelet counts (*AUST2*), and MPS traits (*SHISAL1*), highlighting the complex genetic interactions underlying ASF resistance (**Fig. 6c**).

Beyond immune-related traits, associations were also observed with reproductive traits (e.g., litter weight), production traits (e.g., body length), and meat traits (e.g., meat quality) (**Fig. 6b** and **c**). Key genes implicated in these associations include *C4H1orf226*, *TPST1*, and *ABCG5*, indicating that ASF resistance mechanisms may intersect with growth and reproductive traits (**Fig. 6b** and **c**). These findings underscore the importance of further studies to balance disease resistance with production performance in breeding programs.

Construction and application of ASF polygenic resistance score

To facilitate the practical implementation of our findings in genetic improvement programs for ASF resistance, we constructed an ASF polygenic resistance score (APRS) based on the ASF-resistance prioritized loci identified in this study. The APRS was defined by aggregating favorable alleles across prioritized loci and calculating each individual pig's score based on its identity-by-state (IBS) similarity to this ideal resistant genotype (**Fig. 6d**).

Our APRS metric demonstrated strong discriminatory power, effectively separating resistant, susceptible, and deceased pigs (**Fig. 6e**). To evaluate the robustness of our scoring model, we compared APRS based on selected loci to scores derived from randomly selected loci or genotypes. Randomized APRS scores failed to differentiate between resistant and deceased individuals (**Fig. 6e** and **Supplementary Tables S21**),

confirming that the identified loci significantly contribute to ASF resistance and are not influenced by random variation.

To validate the reliability of the APRS, we evaluated its association with polygenic scores (PGS) for health traits derived from PigBiobank [22]. PGS for 59 traits overlapping with prioritized gene regions were constructed using Lassosum [41], with parameter optimization performed through PUMAS [42]. For consistency validation, PGS for the same traits were independently generated using PRSice-2 [43] with the clumping and thresholding (C+T) method.

Our analysis revealed a significant positive correlation between the PGS for platelet distribution width (S_PLDWID) and APRS, consistent with the results from the genetic correlation analysis (**Fig. 6a**, **f**, **Supplementary Table S18** and **S22**). Individuals in the top decile of APRS exhibited significantly higher $PGS_{\_S\_PLDWID}$ values compared to those in the bottom decile. Additionally, positive correlations were observed between APRS and the PGS for several other traits, including mean corpuscular volume (S_MCV), lipases (S_LIPA), eosinophil number (S_EOS), IFG-IL10 ratio (S_IFGIL10), and IgA level (S_IGA), as determined by both Lassosum and PRSice-2 (**Fig. 6f** and **Supplementary Table S22**).

These results highlight the utility of APRS in identifying traits associated with ASF resistance and provide indirect validation of its potential role in disease resistance breeding programs. Furthermore, our findings demonstrate that APRS is a robust genomic tool for evaluating ASF resistance potential, offering a promising approach for marker-assisted selection in pig breeding programs.

Discussion

ASF continues to pose a major threat to global swine production, yet our understanding of host genetic mechanisms conferring resistance remains limited [9,13]. To address this gap, our study employed a multi-dimensional strategy integrating whole-genome sequencing, functional validation, transcriptomic annotation, and predictive modeling to dissect the genomic architecture of ASF resistance in pigs. Specifically, we developed an ASF Polygenic Resistance Score (APRS) based on identified resistance-associated loci. Our findings provide both theoretical insights and practical tools for developing ASF-resistant pig lines, offering significant implications for sustainable swine production and outbreak preparedness.

Our comprehensive approach led to identify 1,102 candidate genes associated with ASF resistance, which were refined to 135 high-confidence genes through a rigorous prioritization framework. This supports the polygenic nature of ASF resistance,

reflected in the diverse genomic distribution of these genes. Enrichment analyses revealed their significant roles in immune-related pathways, aligning with known responses to ASF infection, such as chemokine upregulation [44], MAPK signaling activation [45], and immune cell apoptosis, particularly in myeloid and lymphoid lineages [46].

Chemokines play a critical role in orchestrating immune cell recruitment and activation, contributing to both protective immune responses and pathological outcomes [47]. Our study identified chemokines, including *CXCL2*, *CXCL7*, *CXCL10*, and *CXCL11*, as key contributors to ASF resistance. F_{ST} analysis and allele frequency differences revealed significant genetic differentiation in these genes between ASF-resistant and susceptible populations, highlighting their potential evolutionary importance in shaping resistance traits. Additionally, SMR analysis demonstrated that the expression levels of these chemokines in specific tissues causally influence ASF resistance, while DEG profiling further confirmed their dynamic response to ASF infection. Collectively, these findings underscore their dual roles in resistance mechanisms: functioning as determinants of genetic predisposition and mediators of infection responses.

Our study also highlights the critical roles of the FcγR (*FCGR2B*, *FCGR3*) and PI3K-AKT (*SOS1* [30], *MVP* [48,49], *PARM1* [50], *TMCC3* [51], *SLC3A1* [52], *BTC* [53]) signaling pathways in ASFV infection. The FcγR family mediates immune regulation through IgG binding, with FCGR3 functioning as an activating receptor and FCGR2B as the sole inhibitory receptor [54], exhibiting opposing immunomodulatory effects. Our analyses revealed a negative correlation between *FCGR3* expression and ASF resistance (supported by SMR, coloc, and downregulation in PAM following infection), while *FCGR2B* expression showed a positive correlation with resistance (validated by SMR, coloc, BN-GWAS, and upregulation in multiple tissues post-infection). As a regulator of the MAPK, PI3K/JAK cellular signaling pathways and tumorigenesis [29,30], *SOS1* was identified as having a positive effect on ASF resistance. *MVP* mediates immune signaling through JAK/STAT and MAPK pathways [48,49], *SLC3A1* activates AKT signaling, contributing to tumorigenesis [52].

In addition, functional insights into genes validated by three methods suggest diverse biological roles: *KIT* is implicated in tumor cell evasion of TGF-β-mediated growth inhibition [55]. *SDAD1* modulates microglial inflammatory responses via NF-κB [56], and counteracting porcine reproductive and respiratory syndrome virus (PRRSV) infection [57]. *HGSNAT*, encoding a lysosomal membrane acetyltransferase, has been implicated in lysosomal and inflammatory dysregulation when misfolded in mice [58], whereas *ZNF394*, a transcriptional repressor in MAPK pathway, has prognostic value in lung squamous cell carcinoma [59,60]. Other genes, such as *ZNF713* [61,62], *CLIP2* (also known as *CYLN2*) [63], *PHF21B* [64,65], and *SDCCAG8* [66–68], are associated with neurological

disorders and reproductive traits, further demonstrating the pleiotropic nature of these loci. These findings suggest that ASFV may exploit these pathways to suppress host immune responses.

ASFV infection manifests as a severe systemic disease, marked by extensive hemorrhages and lesions across multiple organs [33,35]. Our time-course transcriptomic data confirm a widespread host response, with the majority of prioritized genes (127/132) exhibiting dynamic expression changes post-infection. While these responses were most prominent in lymphoid tissues, such as mandibular lymph nodes, tonsils, and mesenteric lymph nodes, underscoring the central role of immune organs, viral quantification revealed a distinct spatial pattern. Specifically, tissues not primarily linked to immune functions harbored substantially lower ASFV RNA levels compared to primary targets like the spleen and PAMs. Consequently, the elevated expression of immune-related genes in these non-target tissues likely reflects systemic inflammatory signaling rather than significant local viral replication. The detection of low-level virus in these regions is plausibly due to secondary seeding via viremia as the infection progresses. This model is further supported by the annotation of our prioritized genes, which, via mouse embryonic spatial transcriptomic, revealed their widespread expression across diverse tissues. The heritability enrichment in non-immune organs like the intestines, brain, and heart suggests the existence of a cross-tissue resistance network that extends beyond canonical immune sites. Notably, approximately 27% of the prioritized genes (36/132), including *ADD1*, ZNF family members, and *RUSF1*, were broadly expressed across tissues, providing molecular support for a systemic and interconnected host defense mechanism.

At the cellular level, macrophages are well-established as primary targets of ASFV infection and replication [69]. Consistently, our heritability enrichment analysis identified Mac_CD163 and Mac_PLBD1 subtypes as critical mediators of resistance mechanisms. Consistent with Zheng et al.'s findings, the Mac_CD163 subpopulation is pivotal in the early host defense against ASFV, characterized by a massive transcriptomic response and dynamic population shifts [31]. Despite an initial sharp decline in prevalence post-infection, this subset demonstrated a remarkable ability to restrict viral replication, with a consistently low proportion of cells carrying a high viral load [31]. This coordinated response, featuring profound DEG enrichment and effective viral control, establishes Mac_CD163 cells as a key mediator of innate antiviral immunity. Single-cell transcriptomic profiling [31] revealed that robust and sustained activation of Mac_CD163 in response to ASFV infection, with *CXCL2* and *CXCL10* genes showing continuous upregulation in these cells, reinforcing their roles as key innate immune effectors [70,71]. Basal expression analysis also identified genes such as *CXCL2*, *CXCL7*, and *BTC* as exhibiting high specificity to PAM, which are critical sites for viral entry and replication [31]. Collectively, these findings emphasize the interplay between tissue-specific and

systemic immune responses in driving ASFV resistance.

ASFV infection is also associated with hemostasis abnormalities and thrombocytopenia [33,72], findings that are supported by our genetic analysis. Genetic correlation analysis revealed a positive association between ASF resistance and platelet distribution width (S_PLDWID) and a negative association with red blood cell distribution width (S_RCDW). These results suggest that variations in blood cell distribution indices may play a role in shaping the genetic architecture of ASF resistance. Furthermore, the positive correlation between PGS_{S_PLDWID} and APRS underscores the critical role of platelet variability and immune activation in resistance mechanisms. For example, shared genetic signals were observed between ASF resistance and traits such as lysozyme levels (S_LYSOZ), granulocyte phagocytosis (S_GRANP), blood albumin levels (S_ALBU), and low-density lipoprotein (S_LDL). These associations suggest that loci contributing to ASF resistance also influence diverse biological pathways. Elevated lysozyme levels, for instance, may enhance mucosal immunity [73], while improved granulocyte function likely strengthens the innate immune response [74], providing a robust first line of defense against ASFV infection.

To translate these insights into actionable breeding tools, we developed the APRS, leveraging identity-by-state similarity to an “ideal” ASF-resistant genotype. The APRS effectively distinguished resistant, susceptible, and deceased individuals, outperforming randomized controls and demonstrating robustness and predictive capability. The pleiotropic effects of the prioritized genes and the utility of the APRS were further validated through the association between APRS and polygenic scores (PGS) derived from various health-related traits. Notably, APRS exhibited significant correlations with PGS for traits such as S_PLDWID, mean corpuscular volume (S_MCV), lipase activity (S_LIPA), eosinophil counts (S_EOS), the IFG-IL10 ratio (S_IFGIL10), and IgA levels (S_IGA). These traits reflect key aspects of systemic immune regulation, metabolic stability, and adaptive immune responses, reinforcing the notion that loci associated with ASF resistance exert pleiotropic effects across multiple biological systems.

Current ASF control strategies, focused on pre-emptive biosecurity and culling [13], necessarily constrain the collection of large-scale biological samples from natural outbreaks. This has limited the scope of genomic investigations into host resistance. Our study, representing the largest cohort-based analysis under natural infection conditions to date, provides critical insights into the host genetic response to ASFV. While the sample size remains modest compared to standard GWAS and may limit power to detect variants of small effect, we mitigated this constraint through a multidimensional analytical framework that integrated diverse omics datasets. Future studies with expanded cohorts will be crucial to validate these findings and refine the

genetic architecture of ASF resistance across diverse populations.

Functional validation also remains an essential next step. While computational analyses consistently supported the prioritized genes and pathways, approaches such as CRISPR-Cas9 or transgenic models are needed to confirm causal roles. Recent work by Pannhorst et al [75]. demonstrated that the porcine SLA class II complex is indispensable for ASFV infection, with knockout of *SLA-DMA*, *SLA-DMB*, and *RFXANK* leading to profound replication defects across ASFV isolates. In contrast, our study did not identify SLA-related genes. This discrepancy likely reflects the complex architecture, poor annotation, and high polymorphism of the SLA locus, which together complicate the detection of gene-specific signals in genome-wide analyses.

Our findings, derived from a specific population under controlled conditions, may not fully capture the genetic and environmental diversity in global pig populations. Comparative insights from wild suids help contextualize this limitation. African warthogs, which show natural resistance to ASFV, harbor adaptive introgressed immune loci, including MHC and FCGR locus [76], reflecting pathogen-driven selection. Our results independently highlight *FCGR* genes in domestic pigs, suggesting conserved protective functions. Additional overlap was observed for the IL, HERC, and SLC [77,78] gene families, while other wild suid – specific candidates such as *PPEF2* [78], *CFAP69* [78], and *JAKMIP1* [76] appeared in our gene sets to varying degrees. By contrast, genes including *LDHB* [77], *RELA* [79], and members of the TRIM family [77] were not detected in our study population. These comparisons illustrate the value of integrating domestic and wild suid datasets to strengthen biological plausibility.

Lastly, the APRS, though robust, focuses on known SNPs and may overlook contributions from rare variants or epigenetic factors, which future research should address through rare variant analysis and epigenomic profiling. These limitations underscore the importance of continued research to enhance ASF resistance breeding and improve preparedness for ASFV outbreaks.

Our findings lay the foundation for host-targeted ASF control strategies by providing both theoretical insights and practical tools for selective breeding. The integration of ASF-resistance genetic markers into breeding programs could facilitate the development of ASF-resistant pig lines, enhancing sustainable swine production and improving outbreak preparedness. Future research should focus on the functional validation of candidate genes through in vitro and in vivo assays and the genomic optimization of resistance loci to refine breeding indices. Collectively, this study advances our understanding of the genetic basis of ASF resistance and offers actionable strategies to mitigate the impact of this devastating disease on the swine industry.

Methods

Experimental population and ethics approval

All pigs were maintained under standardized management and environmental conditions representative of commercial production. Blood and ear samples were systematically collected for antibody and antigen phenotyping by the Institute of Animal Science and Veterinary Medicine, Shandong Academy of Agricultural Sciences (Shandong, China). Before transport and analysis, all veterinary samples were heat-inactivated at 70 °C for 30 min. After experimental procedures, all materials were autoclaved and disposed of in accordance with biosafety regulations. Ethical approval for animal sampling was obtained from the Institutional Animal Care and Use Committee of Zhejiang University (ZJU2021LZGC001), and all procedures involving animals complied with institutional and national ethical guidelines.

Phenotyping

Blood samples were collected from 474 individuals of an indigenous pig population for phenotypic testing. Antibody and antigen detection were performed using the Indirect ELISA colloidal gold test strip method (ID. Vet, France) and the RAA fluorescence quantification method (AMPLIFICATION FUTURE, WLE8202KIT), respectively, following the manufacturers' instructions. For antibody detection, the ID. Vet Indirect ELISA kit (https://www.innovative-diagnostics.com/produit/id-screen-african-swine-fever-indirect/) was used to target ASFV structural proteins P32, P62, and P72, which are highly conserved and widely adopted in ASF diagnostics. For viral DNA detection, the assay targeted the *KP177R* sequence within the *p22* coding region of the ASFV strain China/2018/AnhuiXCGQ (MK128995.1), a region known for its high sequence conservation. Amplification was conducted using a rapid DNA amplification kit, and fluorescence-based probe detection was employed for confirmation and quantification, ensuring high sensitivity and specificity in ASFV identification.

Based on serological and antigen testing, 474 pigs from an indigenous population were classified into three phenotypic groups (**Table 1** and **Supplementary Table S1**): susceptible–dead pigs (Group A, n = 108), susceptible–resistant pigs (Group B, n = 222), and double-negative pigs (Group C, n = 144). Sampling was conducted after extended observation periods: pigs that succumbed to infection were sampled at the point of imminent death, whereas resistant individuals survived for at least one week or longer thereafter. Several individuals underwent repeated testing, and final classifications were determined based on the latest confirmed results to ensure accuracy.

Concurrent testing of environmental samples from the same farm consistently yielded negative results, indicating that viral exposure was primarily mediated through pig-to-

pig transmission rather than environmental contamination. Although differences in viral dose or strain cannot be completely excluded, the contrasting outcomes observed among individuals within a shared environment, together with longitudinal monitoring, support the presence of inherent host resistance differences.

Genotyping

We used whole-genome re-sequencing approach to genotype each individual, achieving an average sequencing depth of 14.3× and a coverage of 0.98 (**Table 1** and **Supplymentary Table S1**). Genomic DNA was isolated from ear tissue of each individual using the CTAB method. Sequencing was conducted on the DNBSEQ-T7 platform, generating 150-bp paired-end reads with an insert size of 350 bp. For variants calling and quality control, we followed a pipeline as in our previous study [80]. Briefily, the raw FASTQ data underwent quality control, read filtering, and base correction using fastp v0.20.0 with default parameters [81]. High-quality reads were then aligned to the Sscrofa11.1 [82] reference genome using BWA v0.7.17 [83] with the MEM algorithm and parameters optimized for paired-end data. Subsequent processing involved converting SAM files into BAM format and sorting them with samtools v1.10 [84]. Duplicate and unmapped reads were removed with sambamba v0.7.1 [85]. We calculated coverage and depth for each individual with Mosdepth v0.2.9 [86]. Next, we applied GATK v4.1.6 [87] with the HaplotypeCaller function (--read-filter GoodCigarReadFilter) to each sample, generating an intermediate GVCF file, which was then employed in GenotypeGVCFs function for joint genotyping across all samples. The resulting variants were filtered with VCFtools v0.1.13 [88] (--maf 0.05, --max missing 0.9), yielding a total of 23,403,868 variants (including SNPs and indels). Finally, genotypes were phased using BEAGLE v4.1 [89] with default parameters.

To provide a representative genetic background for population-level comparison, 1,730 pigs from the PHARP database [80] were included as controls (Group D, **Table 1** and **Supplementary Table S1**). These individuals were not tested for antibodies or antigens but served as a reference cohort capturing natural allelic variation across diverse commercial and local pig populations, thereby enabling detection of population-specific selection and differentiation signals associated with ASF resistance.

Population structure analysis

Population structure of the experimental pigs was assessed using principal component analysis (PCA), neighbor-joining (NJ) tree construction, and ADMIXTURE analysis. After pruning variants for linkage disequilibrium (LD) with PLINK v1.9 [90] (--indep-pairwise 50 5 0.4), PCA was conducted (--pca). The NJ-tree was built using MEGA v11 [91] and visualized with iTOL v6 [92], and genetic ancestry was inferred via neural-admixture v1.6.3 [93].

Genomic-based identification of ASF-resistance candidate genes

We employed four complementary genomic comparison scenarios to dissect the multifactorial nature of ASFV resistance (**Fig. 1** and **Table 2**). These contrasts targeted specific defense mechanisms, from pathogen recognition to adaptive immunity, to minimize phenotypic ambiguity and maximize detection power for causal loci under natural infection. To identify potential genetic loci under selection due to ASF infection, we employed two primary methodologies: genome-wide association studies (GWAS) and selection signal analysis combined with allele frequency examination. Initially, GWAS was utilized to identify variants associated with ASF resistance. This method involved scanning the genomes of both resistant and susceptible pigs to identify genetic markers that correlate with resistance to ASF. Recognizing that different resistance or susceptibility to ASFV can shape the genome, we further focused on identifying genomic regions with significant differences in allele frequencies between the resistant and susceptible groups. Specifically, we targeted genomic regions characterized by: i) High differentiation (F_{ST}): genomic regions showing high differentiation between ASF-resistant and ASF-susceptible groups, indicating strong selective pressure. ii) Inverse allele frequency pattern: alleles that display opposite frequency trends between ASF-resistant and ASF-susceptible groups, suggesting divergent selection. iii) Replicable allele frequency pattern: consistent allele frequency trends observed when comparing resistant pigs to control pigs from other breeds that have not experienced ASF outbreaks (e.g., Group D). This replication across different populations strengthens the validity of the identified loci. Variant annotation was performed using R GALLO v1.3.

Genome-wide association analysis

To identify potential genetic loci associated with ASF resistance, we conducted a GWAS comparing the Case group to Control group 1 within the experimental pig population. We used GCTA v1.92.4 software (--make-grm) [94] to calculate the kinship matrix. GWAS was performed uisng GEMMA v0.98.5 software (-lmm 1) [95] with MLMA-LOCO [96] approach. The statistical model applied was:

$$y = \mu + \mathrm{x}\mathrm{b} + \textstyle\sum_k w_{ik}\, u_k + e,$$

where y denotes the grouping of an individual (1 for control, 2 for case), μ is the fixed mean term, x is the genotype variable for a SNP, b is the fixed effect that is a function of the difference in allele frequencies between the two populations, $\sum_k w_{ik}\, u_k$ is the fit term for all SNPs on the other chromosomes to control for population differentiation, w_k is the standardized genotype variable for an SNP k, u_k is the corresponding effect size of SNP k, and e is the residual error term [97]. We retained loci that met the significance threshold of p-value < $\frac{1}{Me}$, where *Me* represents the total number of loci obtained post-LD pruning. The LD pruning was executed using PLINK v1.9 with parameters --indep-pairwise 50 5 0.4 [90].

Fixation index

We calculated F_{ST} values for each genomic segment to measure genetic differentiation using VCFtools v0.1.13 with parameters --fst-window-size 100000 --fst-window-step 10000. To assess differences in allele frequencies at each locus, we also conducted a chi-square test for allele frequencies at each locus using PLINK v1.9 [90] (--assoc). We considered those that met the following four conditions to be candidate loci: i) the top 1% of highly differentiated segments were retained based on the F_{ST} values; ii) Selected loci with a chi-square test *p*-value < $\frac{0.05}{N}$, were *N* is the total number of loci; iii) Ensured that loci met both criteria (i) and (ii) in both comparisons: Case *vs.* Control 1 and Case *vs.* Control 2; iv) Consistent direction of allele frequency (*f*) changes across comparisons. Namely, $f_{Case} > f_{Control1}$ and $f_{Case} > f_{Control2}$ or $f_{Case} < f_{Control1}$ and $f_{Case} < f_{Control2}$.

Gene prioritization

Transcriptome-wide association analysis

To evaluate the association between our candidate genes and ASF across various tissue types (including 34 tissues from PigGTEx [21], **Supplementary Table S6**) and to elucidate their functional roles, we conducted transcriptome-wide association studies (TWAS) analysis using the online tool FarmGTEx TWAS-server [98] (https://twas.farmgtex.org/). Genomic loci demonstrating significant associations were identified by retaining those with a false discovery rate (FDR) < 0.05.

Summary-data-based mendelian randomization and colocalization with gene expression

We used SMR v1.3.1 [99] (--smr-multi) to assess the causal effect of expression quantitative trait loci (eQTL) (exposure) of candidate genes on ASF resistance (outcome). Variants with *p*-value < 0.005 and LD $r^2 < 0.3$ in the cis-region were selected as instrumental variables. Outcomes were considered to have a significant causal effect if they satisfied all of the following criteria: i) $p_{SMR} < 0.05$, ii) $p_{SMR.MULTI} < 0.05$, and iii) $p_{HEIDI} > 0.05$ (indicating no significant heterogeneity among the instrumental variables).

R coloc v5.2.3 [100] was used to test whether the genetic associations of candidate gene expression in specific tissues with ASF resistance were driven by the same genetic variants. Colocalization analyses were performed on variant loci within 1 Mb upstream and downstream of the gene's significant eQTL locus. Signal pairs with posterior probability of hypothesis 4 (PP4) > 0.75 were considered co-localized, while those with posterior probability of hypothesis 3 (PP3) > 0.75 were treated as being significantly

correlated but driven by distinct causal variant loci.

Both SMR and coloc utilized pooled cis-eQTL data from the PigGTEx database (34 tissues) [21]. Details of the tissue types and sample size are summarized in **Supplementary Table S6** and were originally described in the PigGTEx [21].

Bayesian Network genome-wide association study

We employed a Bayesian Network Genome-Wide Association Study (BN-GWAS) to evaluate the network of causal relationships between candidate gene expression and ASF resistance traits [28]. BN-GWAS constructs directed gene-gene-phenotype causal networks using imputed expression profiles from GWAS and raw expression data from a reference dataset. For this analysis, we used raw expression data from PigGTEx [21] for five tissues with sample sizes exceeding 300: muscle, blood, brain, embryo, and liver (**Supplementary Table S6**).

Omnibus gene prioritization score

We integrated the above validation methods to construct a comprehensive gene prioritization score, which ranks candidate genes based on their potential importance for ASF resistance (**Table 4**). The following criteria were applied:
i) A base score of 1 was assigned to each gene identified by GWAS and F_{ST} methods, with an additional score of 1 for each repetition in a comparison subgroup.
ii) An additional 0.5 score were awarded if the gene was previously reported as an immune gene [31,101].
iii) A score of 1.2 was given if the gene was validated in TWAS or BN-GWAS, with an additional 1.2 score for each repetition in different tissues.
iv) A count of 1 was assigned if the gene was validated by SMR or coloc, with an additional count for each tissue repetition. Due to the multi-tissue analysis, SMR and coloc validations were log-transformed and weighted by 0.8 to prevent over-representation.
v) Independent validation:
- An additional 3 scores were assigned if a gene was validated in all 5 methods.
- An additional 2 scores were given if the gene was validated in 3 or 4 methods.
- An additional 1 score was awarded if the gene was validated in 2 methods.

Pathway enrichment analysis

Pathway enrichment analyses were performed on the prioritized gene sets using Gene Ontology (GO), Kyoto Encyclopedia of Genes and Genomes (KEGG), Reactome, and QTL databases. GO and KEGG enrichment were carried out using R clusterProfiler v4.6.2 [102], Reactome enrichment was done with R ReactomePA v1.42.0 [103], and QTL

annotation and enrichment were conducted using R GALLO v1.5 [104]. All statistical analyses were corrected for multiple comparisons using the Benjamini-Hochberg (BH) method, and results with *p.adj* < 0.05 were considered significant.

Tissue- and cell-type heritability enrichment analysis

We employed the LDSC-SEG [105] model to assess genetic heritability enrichment across 34 tissues from the PigGTEx dataset [21] (**Supplementary Table S6**). Recognizing that porcine alveolar macrophages (PAMs) are primary target cells for ASF infection, we further extended the analysis to eight cell types identified in PAMs. These cell types were characterized using single-cell transcriptomic data from 118,316 cells derived from 13 in vitro samples [31].

The LD reference panel was constructed using PGRP v1 [21], comprising genomic data from 1,602 individuals representing over 100 breeds. Tissue- and cell-type-specific gene regions were defined based on the top 1,000 most highly expressed genes in each tissue or cell type, with an additional 100 kb window to capture surrounding regulatory elements [22,105].

Transcriptome annotation

Mouse embryo spatial transcriptomic data

To annotate the spatial expression pattern of the prioritized genes, we utilized a public mouse embryonic (E16.5) spatial transcriptomics dataset (MOSTA project [34]) from STOmicsDB [106] (STDS0000058 [34]). The data were processed with Scanpy v1.9.1 [107], wherein a composite expression score for the prioritized gene set was computed (sc.tl.score_genes) and visualized (sc.pl.spatial) to map their spatial distribution across tissues.

Multi-tissue bulk transcriptomic analyses

To annotate the prioritized genes within a broader biological context, we analyzed two publicly available bulk RNA-sequencing datasets retrieved from the NCBI SRA [108] (PRJNA960638) and CNCB-NGDC GSA [109,110] (PRJCA003613 [111]) databases (**Supplementary Table S1**). These datasets encompass transcriptomic profiles from 12 pig tissues and cell types at multiple time points after ASFV infection versus uninfected controls, including PAMs, peripheral blood mononuclear cells (PBMC), heart, kidney, liver, lung, inguinal, mandibular, mesenteric, muscle, spleen, and tonsils**.**

i. Quality control and read mapping

Raw RNA sequencing reads underwent quality control using fastp v0.20.0 [81] with

default parameters. High-quality reads were then mapped to the Sscrofa11.1 reference genome using HISAT2 v2.1.0 [112]. Read counts were quantified using featureCounts v2.0.3 (-t exon -g gene_id) [113], and gene expression was quantified at the transcriptional level in transcripts per million (TPM). Genes were considered expressed if TPM > 0.1 in at least 20% of samples. After applying this threshold, 23,331 genes remained available for downstream analyses, including 132 prioritized genes, which were retained for further investigation.

ii. Tissue-specific expression analysis

We assessed the tissue specificity of the prioritized genes using tissue-specific gene expression (TAU) and expression specificity scores (ESS) indices:
TAU Index quantifies the specificity of gene expression across tissues, ranging from 0 to 1, where values closer to 1 indicate higher tissue specificity [114]. It was calculated as:

$$TAU = \frac{\sum_{i=1}^{n}(1-\frac{x_i}{x_{max}})}{n-1},$$

Where n is the number of tissues, x_i represents the expression level of a gene in a given tissue, and x_{max} denotes its highest expression value across all tissues.

ESS Index measures the degree to which a gene is preferentially expressed in a specific tissue, also ranging from 0 to 1, with values closer to 1 indicating stronger expression bias [115]. It was computed as:

$$ESS = \frac{med\ (log_2 TPM)}{\sum med\ (log_2 TPM)}.$$

iii. Differentially expressed genes (DEG) identification

To identify differentially expressed prioritized genes across different infection time points, we performed differential expression analysis using DESeq2 v1.34.0 [116,117] for each tissue separately, incorporating time as a factor in the experimental design. Genes were considered significantly differentially expressed if they met the following criteria: FDR < 0.05 and the absolute log2FoldChange ($|log_2FC|$) > 1.

iv. Time-series analysis of dynamic gene expression

Given that transcriptomic data were collected at multiple post-infection time points, we conducted time-series analysis using R maSigPro v1.66.0 [118]. To account for temporal trends, we applied polynomial regression models with the "backward" variable selection method, setting different degrees for different tissues:

- PAM: degree = 4
- PBMC: degree = 5
- Other tissues: degree = 2

The selection of polynomial degrees was based on the complexity of the time-course experimental design. Specifically, PAM and PBMC datasets had a more intricate temporal structure compared to other tissues, necessitating higher-degree polynomials to better capture gene expression dynamics. Preliminary testing indicated that the chosen degrees provided an optimal balance between model fit and the number of significantly dynamic genes identified. Genes were classified as dynamically responsive genes if they satisfied the following thresholds: FDR < 0.05 and Coefficient of determination (R^2) > 0.5.

Viral transcriptomic analysis

Unmapped reads from the host alignment were aligned to ASFV reference sequences (MK333180.1.fa, MT748042.2.fa) using Bowtie2 v2.3.5.1 [119]. Viral read counts were generated with featureCounts v2.0.3 [113] (-t CDS -g gene_name) and normalized to reads per million (RPM) to quantify viral load. Spearman's rank correlation coefficient was used to assess the relationship between viral RPM and host prioritized gene expression (TPM) across tissues.

Single-cell transcriptomic analyses of PAM

i. Data processing

We retrieved ASFV-infected PAM single-cell RNA sequencing data from the NCBI SRA (PRJNA706032 [31]) (**Supplementary Table S1**). The raw sequencing data were processed using Cell Ranger v7.0.1 [120] with the Sscrofa11.1 reference genome [82]. To ensure high data quality, we applied stringent filtering criteria:

- Mitochondrial RNA content < 10% of total RNA
- Number of detected genes per cell: between 500 and 7,500 [31].

After applying this threshold, 118,316 cells and 14,871 genes remained for downstream analyses, including 127 prioritized genes selected for further investigation. Subsequent analyses were performed using R Seurat v5.0.1 [121]. Cell clustering was performed using the FindClusters function with a resolution parameter of 0.2, ensuring a biologically relevant granularity of clusters. Based on marker genes from Zheng et al. [31], cells were classified into five major populations: macrophages (Mac), mast cells (Mast), T cells (T), proliferating cells (Pro), and epithelial cells (Epi). The macrophage population was further subclustered into four subtypes: Mac_HLA_DRA, Mac_CD163, Mac_CREG1, and Mac_PLBD1 (**Fig. 3d**).

ii. Prioritized gene expression scoring

To quantify the expression levels of prioritized genes across different cell types, we employed the AddModuleScore function in Seurat v5.0.1 [121], which calculates a module

score for a predefined gene set within individual cells. To assess infection-induced changes, module scores of infected cells (categorized by cell type and infection time) were compared to control cells using a two-sided Welch's t-test [122].

iii. Differential gene expression analysis

Differential expression analysis of prioritized genes was conducted using the FindMarkers function in Seurat v5.0.1 [121]. Comparisons were conducted between infected and uninfected groups across different cell types and infection time points. Significantly differentially expressed genes were defined as those meeting the following criteria: FDR < 0.05 and $|\log_2 FC| > 1$.

Genetic correlation analysis

We utilized the LD score regression model in LDSC v1.0.1 [123] to estimate the genetic correlation between ASF resistance and other pig traits. A total of 122 GWAS summary datasets were analyzed, encompassing 121 datasets from PigBiobank [22] (initially 268, with 147 excluded due to insufficient sample size or low heritability) and one dataset from a published GWAS on MPS [38].

The LD reference panel was constructed using PGRP v1 [21] , ensuring a comprehensive representation of SNP linkage patterns. Each GWAS summary dataset was standardized by aligning alleles (A1, A2) and effect sizes. Quality control filters were applied, retaining SNP loci with $|Z| < 5$ to exclude potential outliers and enhance result robustness.

Pleiotropy annotation

To evaluate the pleiotropic effects of ASF resistance-associated genes, we analyzed the overlap between the prioritized gene set for ASF resistance and significant genes identified in other phenotypes. This analysis incorporated data from 298 meta-GWAS studies in the PigBiobank [22] and a published GWAS on MPS [38].

The significance of gene overlaps with phenotypes was determined using hypergeometric tests. For overlapping gene-phenotype pairs (from 268 traits), we applied co-localization analysis using the R coloc v5.2.3 [100]. Genetic variants within a 50-kb window upstream and downstream of lead SNPs were examined. Signal pairs with a posterior probability of hypothesis 4 (PP4 > 0.7) were considered co-localized, indicating shared causal variants. Conversely, pairs with posterior probability of hypothesis 3 (PP3 > 0.7) were interpreted as significantly associated but driven by distinct causal variants.

Construction of ASF polygenic resistance score (APRS) using prioritized loci

Definition of APRS

Identity by state (IBS) refers to the condition where two individuals share the same allele. In this context, IBS states are categorized as 0, 1, or 2 based on the number of shared alleles. Here, we define the IBS-based distance between an experimental and an "ideal" individual as the ASF polygenic resistance score (APRS) (**Fig. 6d**). The APRS evaluates the genetic similarity between an individual and an ideal ASF-resistant genotype, enabling prediction of ASF resistance. The IBS distance is calculated as follows:

$$IBS = \frac{(Number\ of\ IBS2) + (0.5 * Number\ of\ IBS1)}{Total\ Number\ of\ SNPs},$$

where *IBS2* represents loci where both alleles match the ideal genotype, and *IBS1* represents loci where only one allele matches.

Design of ideal genotype

A total of 40 independent loci corresponding to the prioritized genes were selected to define the ideal ASF-resistant genotype. The assignment of ideal alleles was based on statistical evidence from GWAS and F_{ST} analyses (**Fig. 6d**). The specific criteria for ideal allele selection were as follows:

i. GWAS-derived loci:

- If the Z-score of the SNP was positive (Z > 0), the effect allele (minor allele) was designated as the ideal allele.
- If the Z-score was negative (Z < 0), the reference allele (major allele) was assigned as the ideal allele.

The Z-score for each SNP was calculated as:

$$Z = \frac{\beta}{SE},$$

where β represents the effect size, and *SE* is the standard error.

ii. F_{ST}-derived loci:

- The Z-score was defined as the sum of the standardized statistics from two independent comparisons (case/control1 and case/control2).
- The sign of the Z-score (positive or negative) was determined based on the Odds Ratio (OR), ensuring alignment with the direction of selection pressure.

Once the ideal alleles were assigned, the IBS distance between each individual and the ideal genotype was computed using PLINK v1.9 [90] (--cluster-matrix).

Validation by randomized comparison

To assess the specificity and robustness of the APRS, we performed a randomized validation. This involved:
i. Selecting an equal number of random loci using PLINK v1.9 [90] (--thin-count parameter).
ii. Generating "alternative ideal individuals" by disrupting the ideal alleles at the defined loci.
The resistance scores obtained from these random loci served as benchmarks against the APRS values derived from the prioritized ASF-resistance associated loci. This approach ensured that the predictive power of APRS was attributable to ASF-specific genetic variation rather than random genomic background noise.

Polygenic score analysis

Based on the pleiotropy annotation results, 59 health traits from PigBiobank [22] were identified as overlapping with prioritized gene regions. Polygenic scores (PGS) for these traits were generated using Lassosum [41], with PGRP v1 [21] as the LD reference panel. To ensure model robustness, PUMAS [42] was used to subsample GWAS summary statistics, implementing a training-testing data split, cross-validation, and repeated learning. This approach optimized the shrinkage coefficient (s) and lambda (λ) parameters to achieve maximum R^2.

To validate consistency, PGS were also constructed using PRSice-2 [43] with the "clumping and thresholding" (C+T) method (--bar-levels 5e-8, 1e-5, 0.001, 0.05, 0.1, 0.5), allowing direct comparison between the two approaches. The association between PGS and the APRS was evaluated using the Spearman rank correlation coefficient. To further examine the relationship, APRS values were divided into deciles, which highlights extreme phenotypes for better interpretability. Linear regression analysis was conducted using APRS as the response variable and standardized PGS as the predictor variable, focusing on statistical significance in the highest decile compared to the fifth and the lowest (bottom) deciles.

Data availability

The whole-genome sequencing data for the 474 experimental pigs generated in this study are available in the NCBI SRA under accession PRJNA1290525. Additionally, genomic data for other pig breeds are available at PHARP: https://alphaindex.zju.edu.cn/PHARP/index.php. The public transcriptome datasets utilized in this study can be accessed through the Sequence Read Archive (SRA) and the Gene Expression Omnibus (GEO), as specified in the Methods section. EQTL data can be accessed via PigGTEx (https://piggtex.farmgtex.org/), and GWAS summary

statistics for pleiotropic analyses are available upon request from PigBiobank (http://pigbiobank.farmgtex.org/).

References

1. Dixon, L. K., Sun, H. & Roberts, H. African swine fever. *Antiviral Research* **165**, 34–41 (2019).
2. Situation reports for African swine fever (ASF). *WOAH - World Organisation for Animal Health* https://www.woah.org/en/disease/african-swine-fever/ (2025).
3. Sánchez-Cordón, P. J., Montoya, M., Reis, A. L. & Dixon, L. K. African swine fever: A re-emerging viral disease threatening the global pig industry. *The Veterinary Journal* **233**, 41–48 (2018).
4. ASF in Asia: One year on, close to 5 million pigs lost. *Professional Pig Community* https://www.pig333.com/latest_swine_news/asf-in-asia-one-year-on-close-to-5-million-pigs-lost_15239/ (2019).
5. Russia: economic impact of African Swine Fever. *Professional Pig Community* https://www.pig333.com/latest_swine_news/russia-economic-impact-of-african-swine-fever_5019/ (2011).
6. IFIP. ASF draws dangerously close to France: Possible effects on pork trade. *Professional Pig Community* https://www.pig333.com/articles/asf-draws-dangerously-close-to-france-impact-on-pork-trade_18542/ (2022).
7. ASF could cost Australia $2.5 billion. *Professional Pig Community* https://www.pig333.com/latest_swine_news/potential-economic-consequences-of-african-swine-fever-in-australia_19171/ (2023).
8. Carriquiry, M., Elobeid, A., Swenson, D. & Hayes, D. Impact of the Introduction of African Swine Fever in the United States. *Professional Pig Community* https://www.pig333.com/latest_swine_news/if-asf-reached-the-us-it-would-cost-$50-billion_16043/ (2020).
9. Wang, T., Sun, Y. & Qiu, H.-J. African swine fever: an unprecedented disaster and challenge to China. *Infectious Diseases of Poverty* **7**, 111 (2018).
10. Zhou, X. *et al.* Emergence of African Swine Fever in China, 2018. *Transboundary and Emerging Diseases* **65**, 1482–1484 (2018).
11. Liu, J. *et al.* Prevalence of African Swine Fever in China, 2018-2019. *Journal of Medical Virology* **92**, 1023–1034 (2020).
12. You, S. *et al.* African swine fever outbreaks in China led to gross domestic product and economic losses. *Nat Food* **2**, 802–808 (2021).
13. Dixon, L. K., Stahl, K., Jori, F., Vial, L. & Pfeiffer, D. U. African Swine Fever Epidemiology and Control. *Annual Review of Animal Biosciences* **8**, 221–246 (2020).
14. Galindo, I. & Alonso, C. African Swine Fever Virus: A Review. *Viruses* **9**, 103 (2017).
15. Wu, K. *et al.* Current State of Global African Swine Fever Vaccine Development

under the Prevalence and Transmission of ASF in China. *Vaccines (Basel)* **8**, 531 (2020).
16. Mazur-Panasiuk, N., Żmudzki, J. & Woźniakowski, G. African Swine Fever Virus – Persistence in Different Environmental Conditions and the Possibility of its Indirect Transmission. *J Vet Res* **63**, 303–310 (2019).
17. Wang, T., Luo, R., Sun, Y. & Qiu, H.-J. Current efforts towards safe and effective live attenuated vaccines against African swine fever: challenges and prospects. *Infectious Diseases of Poverty* **10**, (2021).
18. Rock, D. L. Challenges for African swine fever vaccine development—"… perhaps the end of the beginning." *Veterinary Microbiology* **206**, 52–58 (2017).
19. Wang, Y. *et al.* Structure of African Swine Fever Virus and Associated Molecular Mechanisms Underlying Infection and Immunosuppression: A Review. *Front. Immunol.* **12**, (2021).
20. Fang, L. *et al.* The Farm Animal Genotype–Tissue Expression (FarmGTEx) Project. *Nat Genet* **57**, 786–796 (2025).
21. Teng, J. *et al.* A compendium of genetic regulatory effects across pig tissues. *Nat Genet* **56**, 112–123 (2024).
22. Zeng, H. *et al.* PigBiobank: a valuable resource for understanding genetic and biological mechanisms of diverse complex traits in pigs. *Nucleic Acids Research* **52**, D980–D989 (2024).
23. Nagarsheth, N., Wicha, M. S. & Zou, W. Chemokines in the cancer microenvironment and their relevance in cancer immunotherapy. *Nat Rev Immunol* **17**, 559–572 (2017).
24. Dong, C., Davis, R. J. & Flavell, R. A. MAP Kinases in the Immune Response. *Annual Review of Immunology* **20**, 55–72 (2002).
25. Waldmann, T. A. The biology of interleukin-2 and interleukin-15: implications for cancer therapy and vaccine design. *Nat Rev Immunol* **6**, 595–601 (2006).
26. Zwick, M. *et al.* Expression of the Phosphatase Ppef2 Controls Survival and Function of CD8+ Dendritic Cells. *Front. Immunol.* **10**, (2019).
27. Kutuzov, M. A., Bennett, N. & Andreeva, A. V. Protein phosphatase with EF-hand domains 2 (PPEF2) is a potent negative regulator of apoptosis signal regulating kinase-1 (ASK1). *The International Journal of Biochemistry & Cell Biology* **42**, 1816–1822 (2010).
28. Yin, L. *et al.* Estimation of causal effects of genes on complex traits using a Bayesian-network-based framework applied to GWAS data. *Nat Mach Intell* **6**, 1231–1244 (2024).
29. Liu, K. *et al.* Nuclear EGFR impairs ASPP2-p53 complex-induced apoptosis by inducing SOS1 expression in hepatocellular carcinoma. *Oncotarget* **6**, 16507–16516 (2015).
30. Akan, D. T. *et al.* Small Molecule SOS1 Agonists Modulate MAPK and PI3K Signaling via Independent Cellular Responses. *ACS Chemical Biology* https://doi.org/10.1021/acschembio.8b00869 (2019) doi:10.1021/acschembio.8b00869.

31. Zheng, Y. *et al.* Transcriptome profiling in swine macrophages infected with African swine fever virus at single-cell resolution. *Proc Natl Acad Sci U S A* **119**, e2201288119 (2022).
32. Fawkner-Corbett, D. *et al.* Spatiotemporal analysis of human intestinal development at single-cell resolution. *Cell* **184**, 810-826.e23 (2021).
33. Salguero, F. J. Comparative Pathology and Pathogenesis of African Swine Fever Infection in Swine. *Front Vet Sci* **7**, 282 (2020).
34. Chen, A. *et al.* Spatiotemporal transcriptomic atlas of mouse organogenesis using DNA nanoball-patterned arrays. *Cell* **185**, 1777-1792.e21 (2022).
35. Zhu, Z. *et al.* Single-cell profiling of African swine fever virus disease in the pig spleen reveals viral and host dynamics. *Proceedings of the National Academy of Sciences* **121**, e2312150121 (2024).
36. Barreto de Albuquerque, J. *et al.* Microbial uptake in oral mucosa–draining lymph nodes leads to rapid release of cytotoxic CD8+ T cells lacking a gut-homing phenotype. *Science Immunology* **7**, eabf1861 (2022).
37. Jiang, M. *et al.* Adducin-1 Facilitates Influenza Virus Endosomal Trafficking and Uncoating by Regulating Branched Actin Dynamics and Myosin IIB Activity. *Advanced Science* **n/a**, 2417318.
38. Xu, Z. *et al.* Selection signature reveals genes associated with susceptibility loci affecting respiratory disease due to pleiotropic and hitchhiking effect in Chinese indigenous pigs. *Asian-Australas J Anim Sci* **33**, 187–196 (2020).
39. Izzi, B. *et al.* Platelet Distribution Width Is Associated with P-Selectin Dependent Platelet Function: Results from the Moli-Family Cohort Study. *Cells* **10**, 2737 (2021).
40. Salvagno, G. L., Sanchis-Gomar ,Fabian, Picanza ,Alessandra & and Lippi, G. Red blood cell distribution width: A simple parameter with multiple clinical applications. *Critical Reviews in Clinical Laboratory Sciences* **52**, 86–105 (2015).
41. Mak, T. S. H., Porsch, R. M., Choi, S. W., Zhou, X. & Sham, P. C. Polygenic scores via penalized regression on summary statistics. *Genetic Epidemiology* **41**, 469–480 (2017).
42. Zhao, Z. *et al.* PUMAS: fine-tuning polygenic risk scores with GWAS summary statistics. *Genome Biology* **22**, 257 (2021).
43. Choi, S. W. & O'Reilly, P. F. PRSice-2: Polygenic Risk Score software for biobank-scale data. *GigaScience* **8**, giz082 (2019).
44. Machuka, E. M. *et al.* Transcriptome profile of spleen tissues from locally-adapted Kenyan pigs (Sus scrofa) experimentally infected with three varying doses of a highly virulent African swine fever virus genotype IX isolate: Ken12/busia.1 (ken-1033). *BMC Genomics* **23**, 522 (2022).
45. Gao, Q. *et al.* Deoxycholic acid inhibits ASFV replication by inhibiting MAPK signaling pathway. *International Journal of Biological Macromolecules* **266**, 130939 (2024).
46. Tian, Y. *et al.* Immune cell early activation, apoptotic kinetic, and T-cell functional

impairment in domestic pigs after ASFV CADC_HN09 strain infection. *Front Microbiol* **15**, 1328177 (2024).

47. Ozga, A. J., Chow, M. T. & Luster, A. D. Chemokines and the immune response to cancer. *Immunity* **54**, 859–874 (2021).

48. Steiner, E. *et al.* The major vault protein is responsive to and interferes with interferon-γ-mediated STAT1 signals. *Journal of Cell Science* **119**, 459–469 (2006).

49. Losert, A. *et al.* The major vault protein mediates resistance to epidermal growth factor receptor inhibition in human hepatoma cells. *Cancer Letters* **319**, 164–172 (2012).

50. Charfi, C., Levros, L.-C., Edouard, E. & Rassart, E. Characterization and identification of PARM-1 as a new potential oncogene. *Mol Cancer* **12**, 84 (2013).

51. Wang, Y.-H. *et al.* Transmembrane and coiled-coil domain family 3 (TMCC3) regulates breast cancer stem cell and AKT activation. *Oncogene* **40**, 2858–2871 (2021).

52. Jiang, Y. *et al.* Cysteine transporter SLC3A1 promotes breast cancer tumorigenesis. *Theranostics* **7**, 1036–1046 (2017).

53. Shi, L. *et al.* Regulatory mechanisms of betacellulin in CXCL8 production from lung cancer cells. *J Transl Med* **12**, 70 (2014).

54. Galvez-Cancino, F. *et al.* Fcγ receptors and immunomodulatory antibodies in cancer. *Nat Rev Cancer* **24**, 51–71 (2024).

55. Küçükköse, E. *et al.* KIT promotes tumor stroma formation and counteracts tumor-suppressive TGFβ signaling in colorectal cancer. *Cell Death Dis* **13**, 617 (2022).

56. Chen, W. *et al.* Regulation of microglia inflammation and oligodendrocyte demyelination by Engeletin via the TLR4/RRP9/NF-κB pathway after spinal cord injury. *Pharmacological Research* **209**, 107448 (2024).

57. Wu, X. *et al.* Major Vault Protein Inhibits Porcine Reproductive and Respiratory Syndrome Virus Infection in CRL2843CD163 Cell Lines and Primary Porcine Alveolar Macrophages. *Viruses* **13**, 2267 (2021).

58. Pan, X. *et al.* Glucosamine amends CNS pathology in mucopolysaccharidosis IIIC mouse expressing misfolded HGSNAT. *Journal of Experimental Medicine* **219**, e20211860 (2022).

59. Wang, K., Li, Y., Wang, J., Chen, R. & Li, J. A novel 12-gene signature as independent prognostic model in stage IA and IB lung squamous cell carcinoma patients. *Clin Transl Oncol* **23**, 2368–2381 (2021).

60. Inhibition of transcriptional activities of AP-1 and c-Jun by a new zinc finger protein ZNF394. *Biochemical and Biophysical Research Communications* **320**, 1298–1305 (2004).

61. Metsu, S. *et al.* A CGG-Repeat Expansion Mutation in ZNF713 Causes FRA7A: Association with Autistic Spectrum Disorder in Two Families. *Human Mutation* **35**, 1295–1300 (2014).

62. Sun, L. *et al.* Peripheral Blood Mononuclear Cell Biomarkers for Major Depressive Disorder: A Transcriptomic Approach. *Depress Anxiety* **2024**, 1089236 (2024).

63. Meyer-Lindenberg, A. *et al.* Functional, structural, and metabolic abnormalities of the hippocampal formation in Williams syndrome. *J Clin Invest* **115**, 1888–1895 (2005).
64. Wong, M.-L. *et al.* The PHF21B gene is associated with major depression and modulates the stress response. *Mol Psychiatry* **22**, 1015–1025 (2017).
65. Li, Q. *et al.* PHF21B overexpression promotes cancer stem cell-like traits in prostate cancer cells by activating the Wnt/β-catenin signaling pathway. *Journal of Experimental & Clinical Cancer Research* **36**, 85 (2017).
66. Hamshere, M. L. *et al.* Genome-wide significant associations in schizophrenia to ITIH3/4, CACNA1C and SDCCAG8, and extensive replication of associations reported by the Schizophrenia PGC. *Mol Psychiatry* **18**, 708–712 (2013).
67. Liu, Z. *et al.* Comprehensive whole-genome resequencing unveils genetic diversity and selective signatures of the Xiangdong black goat. *Front. Genet.* **15**, (2024).
68. Nonneman, D., Lents, C. A., Rempel, L. A. & Rohrer, G. A. Potential functional variants in AHR signaling pathways are associated with age at puberty in swine. *Animal Genetics* **52**, 284–291 (2021).
69. Andrés, G. African Swine Fever Virus Gets Undressed: New Insights on the Entry Pathway. *J Virol* **91**, e01906-16 (2017).
70. Lv, Y. *et al.* CXCL2: a key player in the tumor microenvironment and inflammatory diseases. *Cancer Cell International* **25**, 133 (2025).
71. Liu, M. *et al.* CXCL10/IP-10 in infectious diseases pathogenesis and potential therapeutic implications. *Cytokine & Growth Factor Reviews* **22**, 121–130 (2011).
72. Zakaryan, H. *et al.* Evaluation of hemostaseological status of pigs experimentally infected with African swine fever virus. *Veterinary Microbiology* **174**, 223–228 (2014).
73. Bel, S. & and Hooper, L. V. Secretory autophagy of lysozyme in Paneth cells. *Autophagy* **14**, 719–721 (2018).
74. Kalafati, L. *et al.* Innate Immune Training of Granulopoiesis Promotes Anti-tumor Activity. *Cell* **183**, 771-785.e12 (2020).
75. Pannhorst, K. *et al.* The non-classical major histocompatibility complex II protein SLA-DM is crucial for African swine fever virus replication. *Sci Rep* **13**, 10342 (2023).
76. Garcia-Erill, G. *et al.* Warthog Genomes Resolve an Evolutionary Conundrum and Reveal Introgression of Disease Resistance Genes. *Mol Biol Evol* **39**, msac134 (2022).
77. Feng, W. *et al.* Comparative Genomic Analysis of Warthog and Sus Scrofa Identifies Adaptive Genes Associated with African Swine Fever. *Biology* **12**, 1001 (2023).
78. Xie, H.-B. *et al.* African Suid Genomes Provide Insights into the Local Adaptation to Diverse African Environments. *Mol Biol Evol* **39**, (2022).
79. Palgrave, C. J. *et al.* Species-Specific Variation in RELA Underlies Differences in NF-κB Activity: a Potential Role in African Swine Fever Pathogenesis ▿ . *J Virol* **85**, 6008–6014 (2011).
80. Wang, Z. *et al.* PHARP: a pig haplotype reference panel for genotype imputation. *Sci Rep* **12**, 12645 (2022).

81. Chen, S., Zhou, Y., Chen, Y. & Gu, J. fastp: an ultra-fast all-in-one FASTQ preprocessor. *Bioinformatics* **34**, i884–i890 (2018).
82. Warr, A. *et al.* An improved pig reference genome sequence to enable pig genetics and genomics research. *Gigascience* **9**, giaa051 (2020).
83. Li, H. & Durbin, R. Fast and accurate short read alignment with Burrows-Wheeler transform. *Bioinformatics* **25**, 1754–1760 (2009).
84. Li, H. *et al.* The Sequence Alignment/Map format and SAMtools. *Bioinformatics* **25**, 2078–2079 (2009).
85. Tarasov, A., Vilella, A. J., Cuppen, E., Nijman, I. J. & Prins, P. Sambamba: fast processing of NGS alignment formats. *Bioinformatics* **31**, 2032–2034 (2015).
86. Pedersen, B. S. & Quinlan, A. R. Mosdepth: quick coverage calculation for genomes and exomes. *Bioinformatics* **34**, 867–868 (2018).
87. Van der Auwera, G. A. & O'Connor, B. D. *Genomics in the Cloud*. (O'Reilly Media, 2020).
88. Danecek, P. *et al.* The variant call format and VCFtools. *Bioinformatics* **27**, 2156–2158 (2011).
89. Browning, S. R. & Browning, B. L. Rapid and accurate haplotype phasing and missing-data inference for whole-genome association studies by use of localized haplotype clustering. *Am J Hum Genet* **81**, 1084–1097 (2007).
90. Chang, C. C. *et al.* Second-generation PLINK: rising to the challenge of larger and richer datasets. *Gigascience* **4**, 7 (2015).
91. Tamura, K., Stecher, G. & Kumar, S. MEGA11: Molecular Evolutionary Genetics Analysis Version 11. *Mol Biol Evol* **38**, 3022–3027 (2021).
92. Letunic, I. & Bork, P. Interactive Tree Of Life (iTOL) v5: an online tool for phylogenetic tree display and annotation. *Nucleic Acids Res* **49**, W293–W296 (2021).
93. Dominguez Mantes, A., Mas Montserrat, D., Bustamante, C. D., Giró-i-Nieto, X. & Ioannidis, A. G. Neural ADMIXTURE for rapid genomic clustering. *Nat Comput Sci* **3**, 621–629 (2023).
94. Yang, J., Lee, S. H., Goddard, M. E. & Visscher, P. M. GCTA: A Tool for Genome-wide Complex Trait Analysis. *Am J Hum Genet* **88**, 76–82 (2011).
95. Zhou, X. & Stephens, M. Genome-wide efficient mixed-model analysis for association studies. *Nat Genet* **44**, 821–824 (2012).
96. Yang, J. *et al.* Genetic signatures of high-altitude adaptation in Tibetans. *Proc Natl Acad Sci U S A* **114**, 4189–4194 (2017).
97. Yang, J. *et al.* Genetic signatures of high-altitude adaptation in Tibetans. *Proceedings of the National Academy of Sciences* **114**, 4189–4194 (2017).
98. Zhang, Z. *et al.* FarmGTEx TWAS-server: An Interactive Web Server for Customized TWAS Analysis. *Genomics, Proteomics & Bioinformatics* qzaf006 (2025) doi:10.1093/gpbjnl/qzaf006.
99. Wu, Y. *et al.* Integrative analysis of omics summary data reveals putative mechanisms underlying complex traits. *Nat Commun* **9**, 918 (2018).

100. Wallace, C. A more accurate method for colocalisation analysis allowing for multiple causal variants. *PLOS Genetics* **17**, e1009440 (2021).
101. Klunk, J. *et al.* Evolution of immune genes is associated with the Black Death. *Nature* **611**, 312–319 (2022).
102. Yu, G., Wang, L.-G., Han, Y. & He, Q.-Y. clusterProfiler: an R Package for Comparing Biological Themes Among Gene Clusters. *OMICS* **16**, 284–287 (2012).
103. Yu, G. & He, Q.-Y. ReactomePA: an R/Bioconductor package for reactome pathway analysis and visualization. *Mol. BioSyst.* **12**, 477–479 (2016).
104. Fonseca, P. A. S., Suárez-Vega, A., Marras, G. & Cánovas, Á. GALLO: An R package for genomic annotation and integration of multiple data sources in livestock for positional candidate loci. *Gigascience* **9**, giaa149 (2020).
105. Finucane, H. K. *et al.* Heritability enrichment of specifically expressed genes identifies disease-relevant tissues and cell types. *Nat Genet* **50**, 621–629 (2018).
106. Xu, Z. *et al.* STOmicsDB: a comprehensive database for spatial transcriptomics data sharing, analysis and visualization. *Nucleic Acids Res* **52**, D1053–D1061 (2024).
107. Wolf, F. A., Angerer, P. & Theis, F. J. SCANPY: large-scale single-cell gene expression data analysis. *Genome Biology* **19**, 15 (2018).
108. Sayers, E. W. *et al.* Database resources of the national center for biotechnology information. *Nucleic Acids Research* **50**, D20–D26 (2022).
109. Chen, T. *et al.* The Genome Sequence Archive Family: Toward Explosive Data Growth and Diverse Data Types. *Genomics, Proteomics & Bioinformatics* **19**, 578–583 (2021).
110. CNCB-NGDC Members and Partners. Database Resources of the National Genomics Data Center, China National Center for Bioinformation in 2024. *Nucleic Acids Research* **52**, D18–D32 (2024).
111. Zhao, D. *et al.* Transcription regulation of African swine fever virus: dual role of M1249L. *Nat Commun* **15**, 10058 (2024).
112. Kim, D., Paggi, J. M., Park, C., Bennett, C. & Salzberg, S. L. Graph-Based Genome Alignment and Genotyping with HISAT2 and HISAT-genotype. *Nat Biotechnol* **37**, 907–915 (2019).
113. Liao, Y., Smyth, G. K. & Shi, W. featureCounts: an efficient general purpose program for assigning sequence reads to genomic features. *Bioinformatics* **30**, 923–930 (2014).
114. Yanai, I. *et al.* Genome-wide midrange transcription profiles reveal expression level relationships in human tissue specification. *Bioinformatics* **21**, 650–659 (2005).
115. Thériault, S. *et al.* Integrative genomic analyses identify candidate causal genes for calcific aortic valve stenosis involving tissue-specific regulation. *Nat Commun* **15**, 2407 (2024).
116. Leek, J. T., Johnson, W. E., Parker, H. S., Jaffe, A. E. & Storey, J. D. The sva package for removing batch effects and other unwanted variation in high-throughput

experiments. *Bioinformatics* **28**, 882–883 (2012).

117. Love, M. I., Huber, W. & Anders, S. Moderated estimation of fold change and dispersion for RNA-seq data with DESeq2. *Genome Biol* **15**, 550 (2014).

118. Nueda, M. J., Tarazona, S. & Conesa, A. Next maSigPro: updating maSigPro bioconductor package for RNA-seq time series. *Bioinformatics* **30**, 2598–2602 (2014).

119. Langmead, B. & Salzberg, S. L. Fast gapped-read alignment with Bowtie 2. *Nat Methods* **9**, 357–359 (2012).

120. Zheng, G. X. Y. *et al.* Massively parallel digital transcriptional profiling of single cells. *Nat Commun* **8**, 14049 (2017).

121. Hao, Y. *et al.* Dictionary learning for integrative, multimodal and scalable single-cell analysis. *Nat Biotechnol* 1–12 (2023) doi:10.1038/s41587-023-01767-y.

122. Edahiro, R. *et al.* Single-cell analyses and host genetics highlight the role of innate immune cells in COVID-19 severity. *Nat Genet* **55**, 753–767 (2023).

123. Bulik-Sullivan, B. *et al.* An atlas of genetic correlations across human diseases and traits. *Nat Genet* **47**, 1236–1241 (2015).

Acknowledgements

We thank the pig farms for providing the samples. This work was supported by the Agricultural Improved Varieties Project in Shandong Province of China (2021LZGC001), National Natural Science Foundation of China (31941007).

Author contributions

Conceptualization, Z. Wang and Y. C. Pan; methodology, Z. Wang; validation, X. W. Ye; formal analysis, X. W. Ye and Q. Q. Xie; resources, W.B. Sun; data curation, C. Y. Cao and Shuang Liu; writing—original draft preparation, X. W. Ye and Z. Wang; writing—review and editing, X. W. Ye and Z. Wang; visualization, X. W. Ye; supervision, Y. C. Pan, Q. S. Wang, Z. Zhang and Z. Wang; project administration, Y. C. Pan and Z. Wang; funding acquisition, Y. C. Pan and Z. Wang. All authors have read and agreed to the published version of the manuscript.

Declaration of interest

The authors declare that they have no competing interests.

Figure legends

Figure 1. Study overview.

A schematic representation of the study design and analytical framework:

Experimental grouping and comparative analyses (red): Summarizes experimental groupings, comparisons, and analyses, including GWAS, F_{ST}, allele frequency tests, and gene identification.

Gene prioritization (yellow): ASF resistance candidate genes were functionally validated using five independent methods—overlap with reported immune genes, TWAS, SMR, colocalization, and BN-GWAS. These analyses identified 135 high-priority genes, which were further analyzed through pathway enrichment to elucidate their biological roles.

Tissue and cell-Specific analyses (green): Heritability enrichment analysis pinpointed tissues and cell types (e.g., PAM) associated with ASF resistance. Mouse embryonic ST data provided annotation of prioritized gene expression. Bulk and single-cell transcriptomics data integration revealed basal tissue-specific expression patterns (ESS, TAU) and dynamic gene responses (DEG) post-infection.

Polygenic associations (blue): Global genetic associations between ASF resistance and other pig traits were investigated. Shared genomic regions, trait enrichment, and cross-trait colocalization analyses highlighted the polygenic nature of prioritized genes. Associations between the ASF resistance prediction score (APRS) and polygenic scores (PGS) for other pig health traits were constructed and assessed.

GWAS: Genome-wide association study. F_{ST}: fixaxion index. TWAS: transcriptome-wide association study. SMR: summary-based Mendelian randomization. Coloc: colocalization. BN-GWAS: Bayesian network GWAS. PAM: porcine alveolar macrophage. ST: Spatial transcriptomic. ESS: expression specificity scores. TAU: tissue-specific gene expression. DEG: differentially expressed genes.

Figure 2. Identification and characterization of candidate genes associated with ASF resistance.

a. Upset plot showing the overlap of candidate genes identified across different comparisons. The black lines and colored dots below the bar graph represent specific comparisons, while the bar graph indicates the number of candidate genes identified in those comparisons.

b. Genomic distribution of identified genetic loci. The outermost circle displays chromosomes with annotated prioritized genes. The middle layer uses different colors to represent different comparisons: red for comparison 1, yellow for comparison 2, green for comparison 3, and blue for comparison 4. Within each comparison group, loci identified by specific methods are depicted in sequential rings from outer to inner: GWAS, F_{ST} (case *vs.* control 1), MAF chi-square test (case *vs.* control 1), F_{ST} (case *vs.*

control 2), and MAF chi-square test (case *vs.* control 2).

c. Inner stacked plot summarizing the number of validation methods supporting candidate loci within each comparison group.

Figure 3. Gene prioritization and validation.

a. Gene prioritization overview for the top prioritized genes for ASF resistance. Genes are ranked by their total prioritization scores (high to low). The heat map displays scores across various validation methods, with the y-axis representing prioritized genes and the x-axis showing different scoring methods. Color intensity indicates the score value. The lollipop plot shows the total prioritization score for each gene. Genes with scores $\geqslant$ 3.5 or validated by two or more methods are classified as prioritized genes.

b. Venn diagram illustrating the overlap among 1,102 candidate genes (light blue), 135 prioritized genes (dark blue), and 1,150 previously reported immune-related genes (green).

c. Manhattan plot of 13 candidate genes identified by transcriptome-wide association study (TWAS) (FDR < 0.05, red dashed line), with 11 categorized as prioritized genes. The y-axis shows $-\log_{10}$FDR, the x-axis represents genomic locations, and dot colors indicate correlation direction: orange for positive and blue for negative.

d. 42 causal pairs validated by colocalization (coloc) and summary-based Mendelian randomization (SMR) analyses, demonstrating a causal relationship between gene expression in tissues and ASF resistance. Outer to inner rings represent: tissue sectors, -log10*p*-value from SMR, PP4 from coloc, PP3 from coloc, SMR beta value (red for positive, blue for negative), and a cross-tissue gene network.

e. Validation of 14 candidate genes by Bayesian network GWAS (BN-GWAS), with 12 identified as prioritized genes. The x-axis shows the effect size.

f. Bubble plot showing significant enrichment of 135 prioritized genes. The x-axis represents enrichment fold, bubble color denotes $-\log_{10}$FDR, and bubble shape reflects database categories.

Figure 4. Tissue- and cell-specificity and gene expression analyses.

a. Manhattan plot depicting genetic enrichment significance for four comparisons across 34 tissues and eight PAM cell types. The y-axis shows -log10*p*-value, and the x-axis represents tissues/cell types. Blue and red dashed lines indicate significance thresholds (*p*-value < 0.05 and *p*-value < 0.01, respectively). Significant tissues and cell types are highlighted in orange and labeled.

b. Prioritized gene scoring in mouse embryonic spatial transcriptomics. The dot colors represent gene expression scores across tissues.

c. Baseline expression and dynamic response of prioritized genes to ASF infection in bulk RNA data. The y-axis lists prioritized genes, while the x-axis, from left to right, displays: primary tissue expression distribution (color-coded squares); expression

specificity scores (ESS) values across tissues, with heatmap colors indicating magnitude (red: high, blue: low); tissue-specific gene expression (TAU) values, where TAU > 0.8 denotes high tissue specificity, and TAU < 0.6 indicates widespread expression; differential expression of prioritized genes at various infection time points, with point size representing $-\log_{10}$FDR and color indicating $\log_2$FC. Time units are hours post-infection (hpi) for PAM and days post-infection (dpi) for other tissues.

d. UMAP visualization of cell types in PAM scRNA-seq data, including macrophages (Mac), mast cells (Mast), T cells (T), proliferating cells (Pro), and epithelial cells (Epi).

e. Scoring of prioritized gene sets pre- and post-infection in PAM scRNA-seq. Results are derived using a two-sided Welch t-test (**p*-value < 0.001, ***p*-value < 1×10^{-8}). The y-axis indicates infection time (hpi), the x-axis shows cell types, dot size reflects -$\log_{10}p$-value, and dot color represents statistical measures.

f. Differential expression of prioritized genes across PAM cell types. Square colors represent $\log_2$FC values, with significance marked as **p*-value < 0.05 and $|\log_2\text{FC}| > 1$.

Figure 5. Analyses of ASFV replication and host correlations.

a. ASFV load, measured by RNA reads per million (RPM), across multiple tissues at various time points post-infection. **b.** Spearman correlation analysis between ASFV RPM and the expression (TPM) of prioritized host genes.

Figure 6. Pleiotropic associations.

a. Forest plot illustrating traits with significant genome-wide genetic correlations with ASF resistance (*p*-value < 0.05). Orange dots indicate positive correlations, and blue dots indicate negative correlations. Each dot represents the effect value, and lines indicate the 95% confidence intervals (CIs). S_PLDWID: platelet distribution width. S_RCDW: red cell distribution width.

b. Bubble plot illustrating the enrichment of prioritized genes in various pig traits. The y-axis lists traits, while the x-axis represents the enrichment fold. Bubble size corresponds $-\log_{10}$FDR, and bubble color reflects the trait categories.

c. Colocalization analysis highlighting genes shared between ASF resistance and other pig traits. The y-axis lists traits, and the x-axis represents genes. Shades of orange denote PP4 values (PP4 > 0.7, evidence for shared causal variants), while shades of blue denote PP3 values (PP3 > 0.7, evidence for alternative causal variants).

d. Schematic representation of the construction and validation process for the ASF resistance prediction score (APRS) and polygenic scores (PGS) related to other pig health traits.

e. Comparative analysis of "ideal" APRS versus randomly constructed APRS. The Wilcoxon rank-sum test with Bonferroni correction was used (***p*.adjusted < 0.01, *****p*.adjusted < 1×10^{-5}, ns: not significant). The y-axis shows APRS scores, and the x-axis differentiates experimental groupings. Colors distinguish APRS methods: Ideal

(ideal APRS), Random allele (APRS using random alleles), and Random loci (APRS using random loci).

f. Phenome-wide association study (PheWAS) comparing APRS and PGS for pig health traits. The x-axis lists health traits, while the y-axis displays association metrics: Outer Plot (Panels 1 and 4): The y-axis shows $-\log_{10} p$-values from Spearman rank correlation analysis. Dot colors indicate association direction (red for positive, blue for negative), with shades reflecting correlation strength (|ρ|). The black dashed line represents the significance threshold (p-value < 0.05/236, Bonferroni correction). Significant traits are labeled. Inner Plot (Panels 2 and 3): Regression analysis of PGS values for individuals grouped by APRS deciles (top 10%, mid-50%, bottom 10%). The y-axis represents effect estimates, with dots showing effect sizes (*p-value < 0.05, **p-value < 0.01, ***p-value < 0.05/236, Bonferroni correction). Line segments represent 95% confidence intervals (CIs). Dot colors indicate effect magnitude (red for positive, blue for negative), and the red dashed line marks an effect of 0. Trait abbreviations and details are provided in the PigBiobank database.

Tables

Table 1. Grouping of individuals in experimental pig herds.

Group	Antigen[1]	Antibody[2]	Situations	Meaning	Sample size
A	/	+	Dead	Antigen infection → immune response → production of antibodies → pigs die	108
B	-	+	Alive	Antigen infection → immune response → production of antibodies → antigen clearance.	222
C	-	-	Alive	1 Antigen uninfected → no immune response → no antibody<br>2 Antigen infection → immune response → antibody → antigen clearance → antibody inactivation	144
D	/	/	/	The remaining control varieties in PHARP (except Laiwu, Lulai and Duroc x Lulai pigs)	1730

[1] African swine fever virus antigen test, - for negative and + for positive.
[2] African swine fever virus antibody test, - for negative and + for positive.

Table 2. Grouping of experimental comparisons between groups.

Comparison	Case	Control		Phenotype	Potential Meaning
		1	2		
1	B+C	A	D	(Uninfected + survived infection) *vs.* (Dead)	Disease resistance, Protection

2	C	A+B	D	(Uninfected) *vs.* (Survived infection + dead)	Pathogen recognition
3	C	A	D	(Uninfected) *vs.* (Dead)	Protection
4	B	A	D	(Survived infection) *vs.* (Dead)	Adaptive immunity

Table 3. Statistical overview of candidate loci associated with African swine fever.

Comparison	N[1]	Me[2]	Candidate loci		Genes	
			GWAS[3]	F_{ST}[4]	GWAS	F_{ST}
1	23,290,599	2,581,021	719	3,490	43	44
2	23,290,599	2,581,021	56	5,163	15	33
3	23,160,238	2,537,788	2,445	9,453	715	51
4	23,403,868	2,571,190	72	1,848	12	30

[1]The number of all loci.
[2]The number of loci after LD pruning.
[3]The number of loci identified by GWAS method.
[4]The number of loci identified by F_{ST} method.

Table 4. Scoring framework for ASF resistance gene prioritization.

Method	Repeat count	Score Range	Scoring Formula
GWAS	1 per group	0-3	count * 1
F_{ST} + AF test	1 per group	0-3	count * 1
Previously Reported Immune Genes	/	0-1	count * 0.5
TWAS	1 per group, per tissue	0-4	count * 1.2
SMR	1 per group, per tissue	0-43	$\log_2$(count +1) * 0.8
Colocalization	1 per group, per tissue	0-25	$\log_2$(count +1) * 0.8
Bayesian Network GWAS	1 per group, per tissue	0-4	count * 1.2
Independent Validation	/	0-3	2 methods validated: +1;<br>3–4 methods validated: +2;<br>≥5 methods validated: +3

Supplementary files

Supplementary Figure S1-S19

Supplementary Table S1-S22

Figures

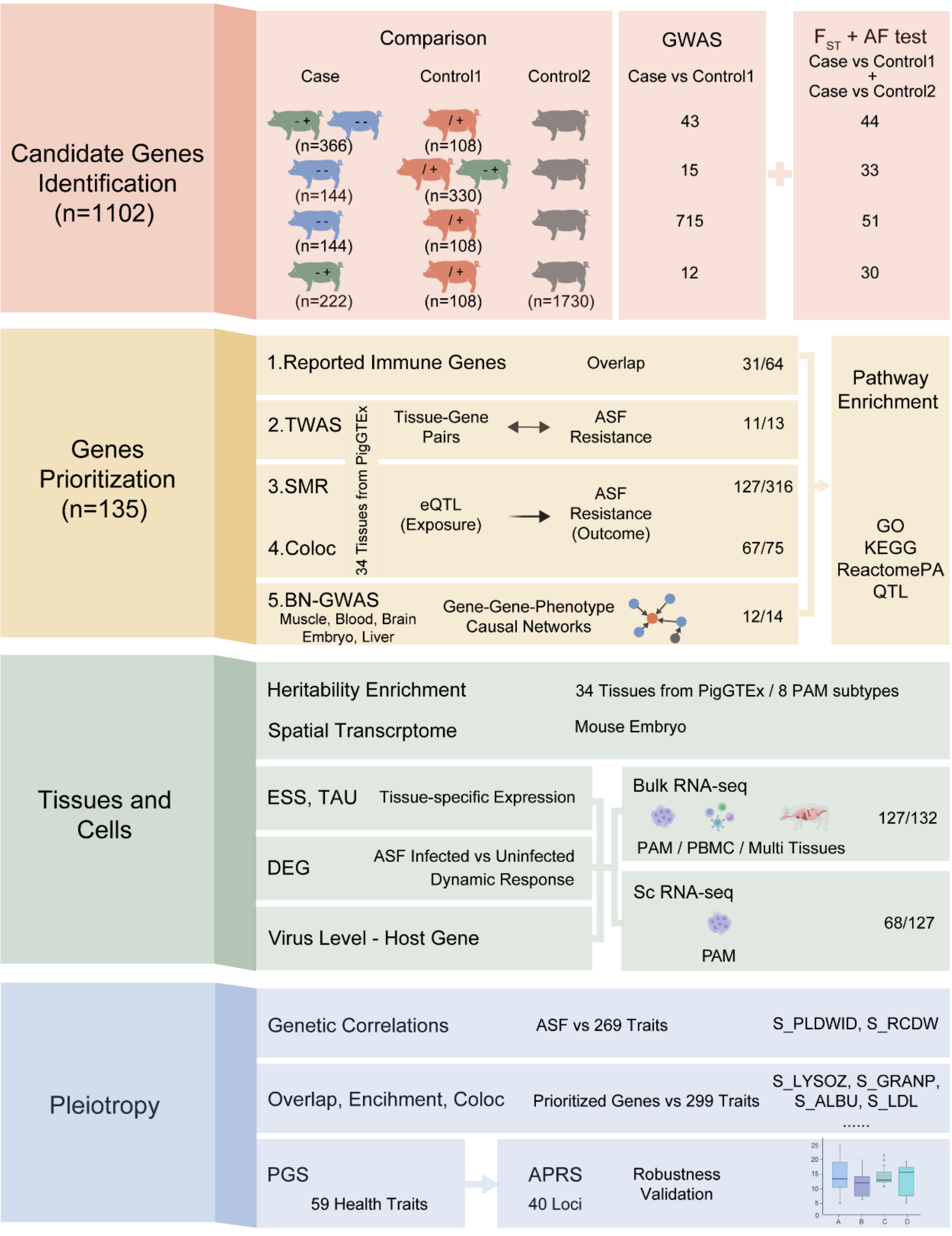

Figure 1.

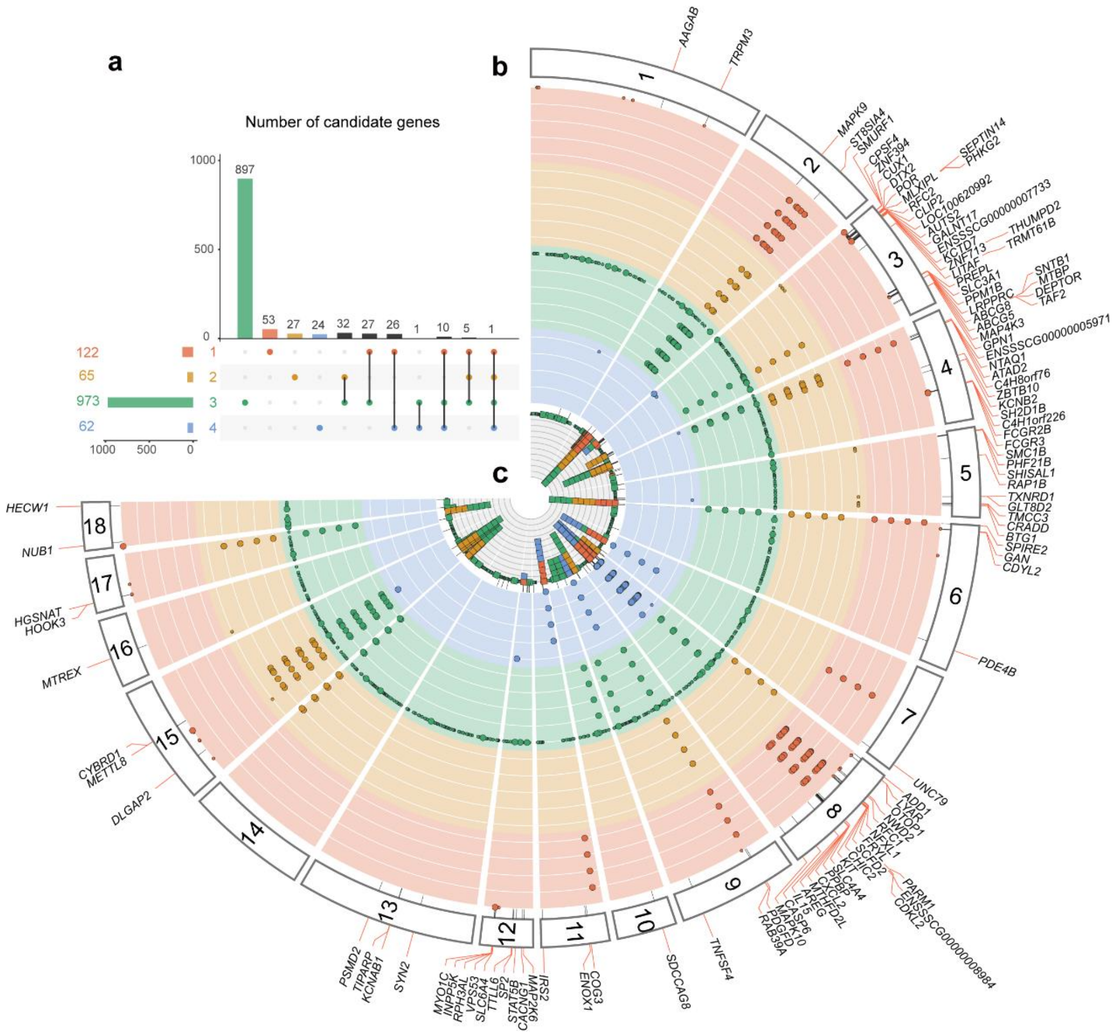
a
b
c
Number of candidate genes
1000
500
0
897
53
27
24
32
27
26
1
10
5
1
122
65
973
62
1000
500
0
1
2
3
4
5
6
7
8
9
10
11
12
13
14
15
16
17
18
AAGAB
TRPM3
MAPK9
ST8SIA4
SMURF1
SEPTIN14
PHKG2
THUMPD2
TRMT61B
SNTB1
MTBP
DEPTOR
TAF2
PDE4B
UNC79
SDCCAG8
TNFSF4
COG3
ENOX1
IRS2
SYN2
PSMD2
TIPARP
KCNAB1
DLGAP2
CYBRD1
METTL8
MTREX
HGSNAT
HOOK3
NUB1
HECW1

Figure 2.

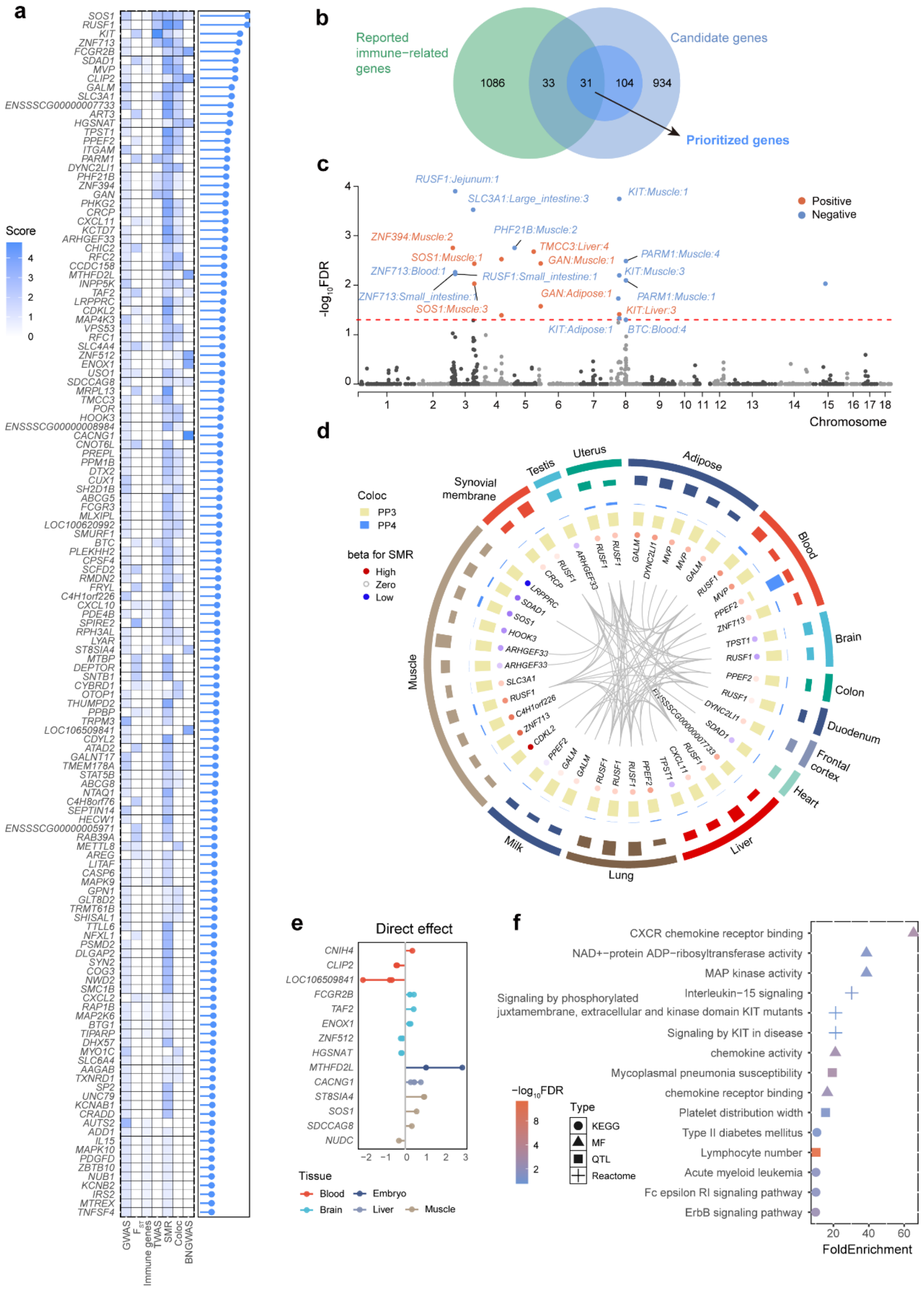
a
Score
GWAS
F_{ST}
Immune genes
TWAS
SMR
Coloc
BNGWAS
b
Reported immune−related genes
Candidate genes
Prioritized genes
1086
33
31
104
934
c
Positive
Negative
$-\log_{10}$FDR
Chromosome
d
Coloc
PP3
PP4
beta for SMR
High
Zero
Low
Synovial membrane
Testis
Uterus
Adipose
Blood
Brain
Colon
Duodenum
Frontal cortex
Heart
Liver
Lung
Milk
Muscle
e
Direct effect
Tissue
Blood
Embryo
Brain
Liver
Muscle
f
CXCR chemokine receptor binding
NAD+−protein ADP−ribosyltransferase activity
MAP kinase activity
Interleukin−15 signaling
Signaling by phosphorylated juxtamembrane, extracellular and kinase domain KIT mutants
Signaling by KIT in disease
chemokine activity
Mycoplasmal pneumonia susceptibility
chemokine receptor binding
Platelet distribution width
Type II diabetes mellitus
Lymphocyte number
Acute myeloid leukemia
Fc epsilon RI signaling pathway
ErbB signaling pathway
$-\log_{10}$FDR
Type
KEGG
MF
QTL
Reactome
FoldEnrichment

Figure 3.

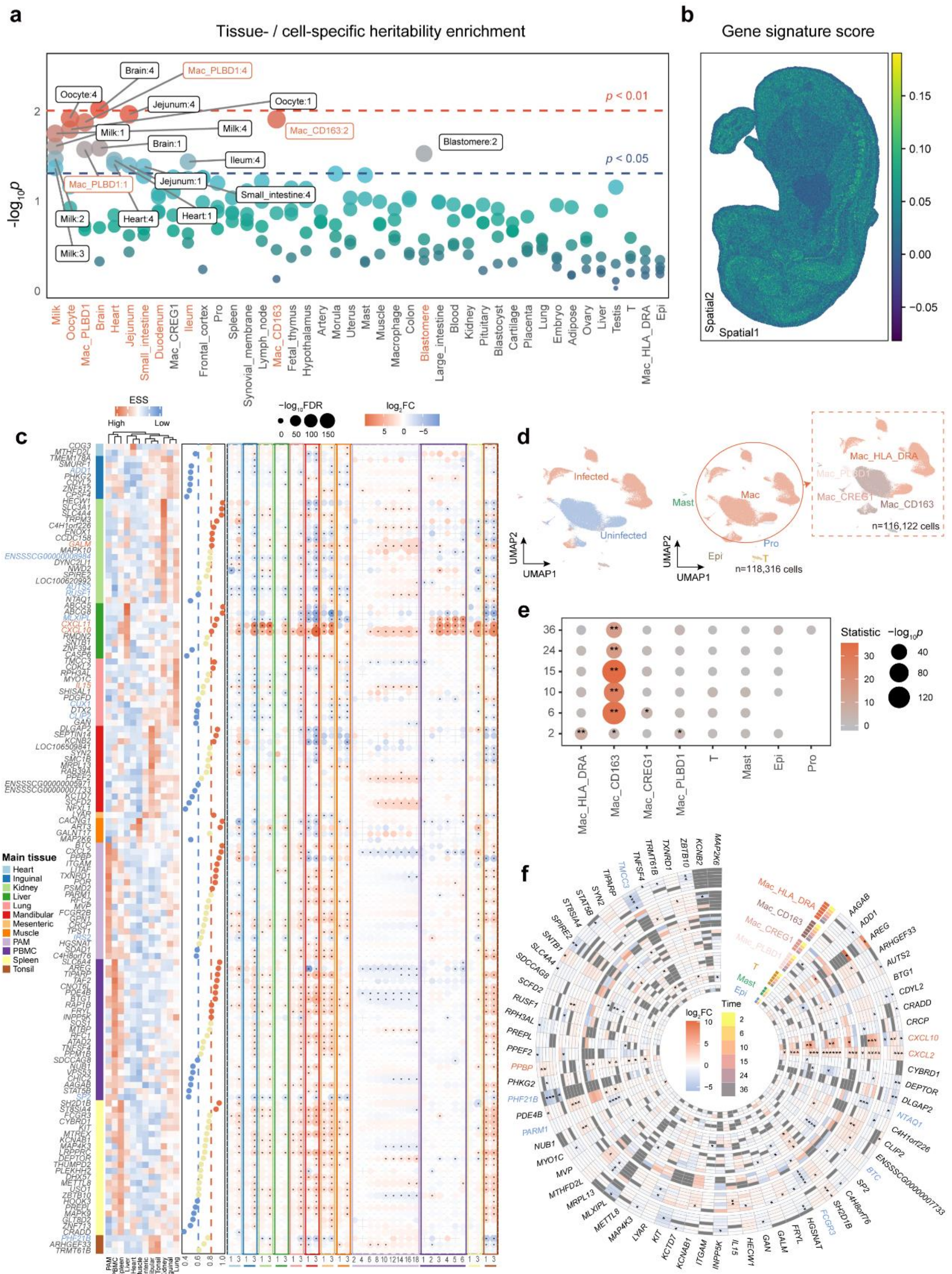

1398 **Figure 4.**

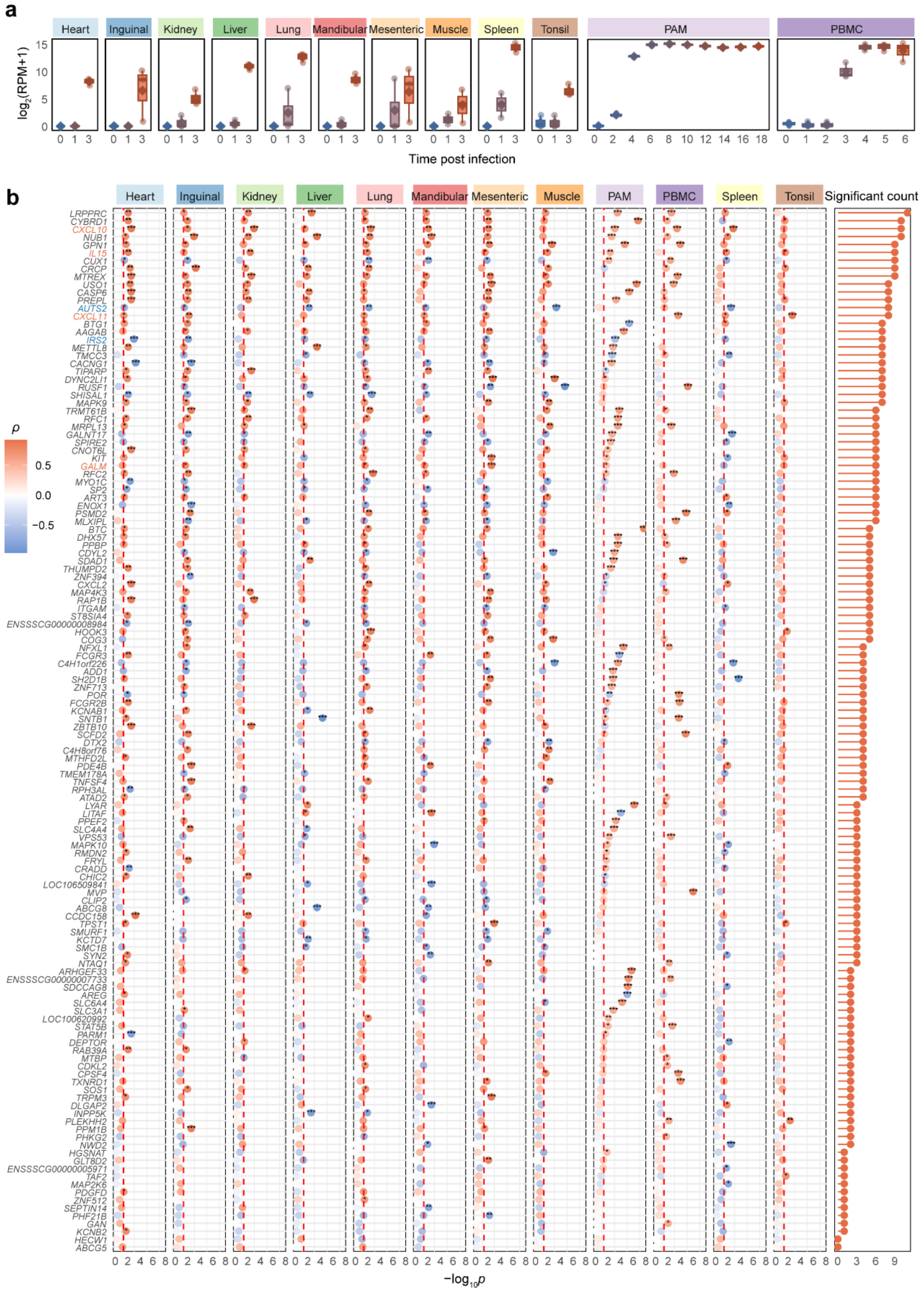
a
Heart
Inguinal
Kidney
Liver
Lung
Mandibular
Mesenteric
Muscle
Spleen
Tonsil
PAM
PBMC
log2(RPM+1)
Time post infection
b
ρ
0.5
0.0
−0.5
Significant count
−log10p

Figure 5.

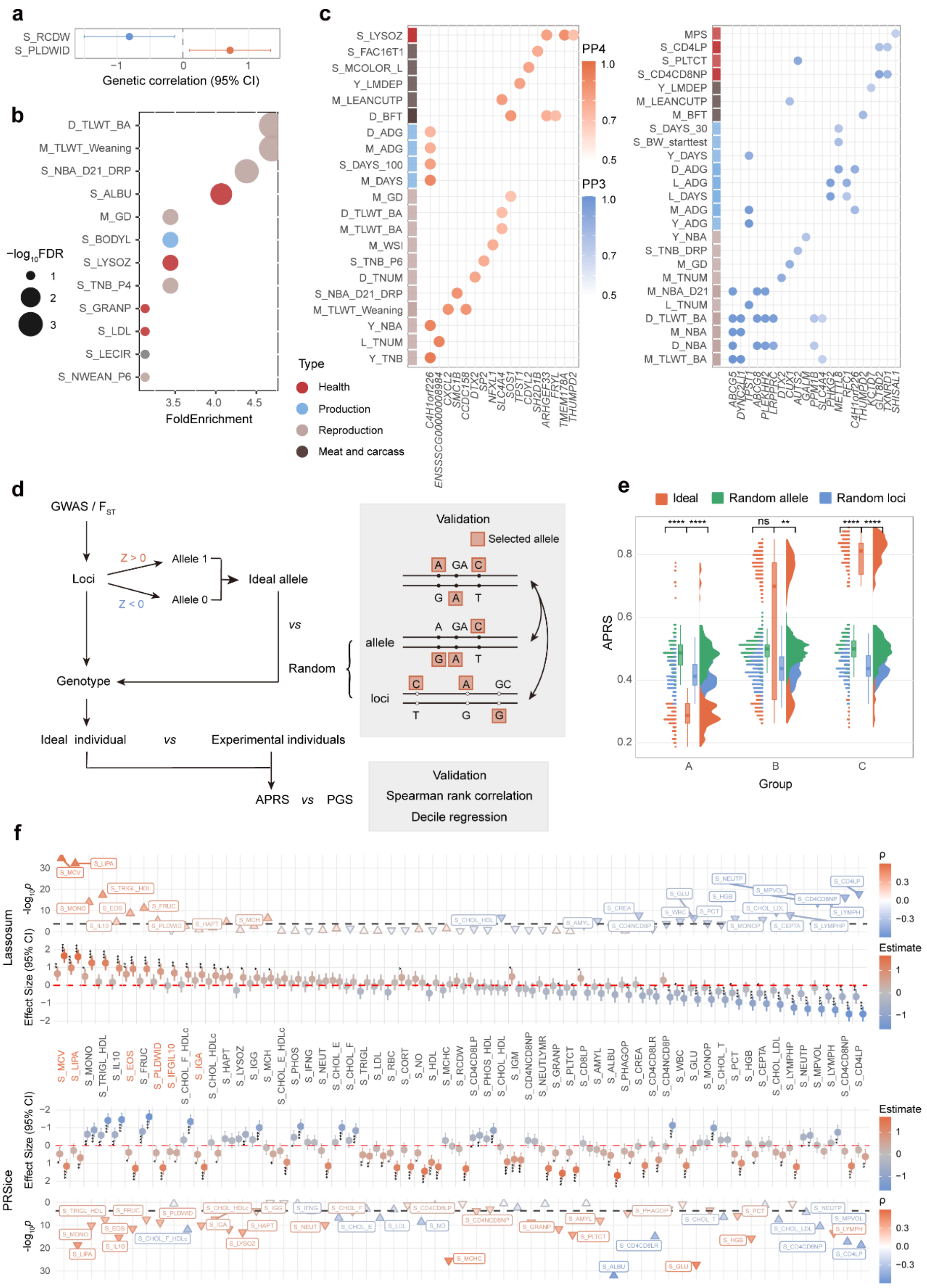

a
S_RCDW
S_PLDWID
Genetic correlation (95% CI)
b
D_TLWT_BA
M_TLWT_Weaning
S_NBA_D21_DRP
S_ALBU
M_GD
S_BODYL
S_LYSOZ
S_TNB_P4
S_GRANP
S_LDL
S_LECIR
S_NWEAN_P6
FoldEnrichment
c
PP4
PP3
Type
Health
Production
Reproduction
Meat and carcass
d
GWAS / F_ST
Loci
Z > 0
Z < 0
Allele 1
Allele 0
Ideal allele
vs
Random
Genotype
Ideal individual
vs
Experimental individuals
APRS
vs
PGS
Validation
Selected allele
allele
loci
Validation
Spearman rank correlation
Decile regression
e
Ideal
Random allele
Random loci
APRS
Group
f
Lassosum
PRSice
Effect Size (95% CI)
Estimate

Figure 6.

Click here to access/download
Supplementary Material
Supplementary information.docx

Click here to access/download
Supplementary Material
Supplementary table.xlsx

Click here to access/download;Personal Cover;Cover letter.docx

ZHEJIANG UNIVERSITY

Zhen Wang, PhD.
Professor
College of Animal Sciences

Zijingang Campus, Zhejiang University
866 Yuhangtang Road
College of Animal Sciences E215
Hangzhou, Zhejiang 310058
wangzhen20@zju.edu.cn

January 30, 2026

The Editor
GigaScience
Dear Editor,

We are pleased to submit our manuscript titled "**Integrated multi-omics profiling identifies genetic loci of African swine fever resistance in pigs**" for consideration in ***GigaScience.*** Given the critical role of pigs as a cornerstone species in global agriculture and their importance as a biomedical model, we believe our findings will resonate strongly with your journal's broad and interdisciplinary readership.

African swine fever (ASF) is a highly contagious and often lethal disease that has ravaged pig populations across 83 countries, causing hundreds of billions of dollars in **economic losses** and posing a critical threat to **global food security**. With case fatality rates reaching nearly 100% in acute infections and 30–70% in subacute or chronic forms, ASF outbreaks have disrupted food supply chains, depleted genetic resources, and driven up pork prices worldwide. The 2018 ASF outbreak in China, which accounts for nearly half of the world's pig population, led to massive herd reductions, severe economic fallout, and significant disruptions to the national pig industry. Despite extensive efforts to develop vaccines and understand ASF virus biology, effective prevention and treatment strategies remain elusive, highlighting the urgent need for innovative solutions to combat this ongoing global crisis.

Our research addresses these pressing challenges by **uncovering the genetic basis of ASF resistance** and offering practical solutions for disease management. Using **a comprehensive multi-omics approach** that **integrates genomic, transcriptomic, and functional analyses,** we have made the following novel contributions:

1. **A Groundbreaking Dataset**: We provide an unparalleled dataset of ASF-resistant and susceptible pigs, offering a unique and valuable resource for advancing ASF research globally.
2. **New Mechanistic Insights into ASF Resistance**:
   - Identifying ASF resistance as a polygenic trait influenced by multiple genes rather than a single dominant factor.
   - Prioritizing 135 resistance-associated genes that illuminate key pathways, including chemokine, MAPK, PI3K-AKT, and Fc γ signaling.
   - Demonstrating the central role of Mac_CD163 cells in resistance mechanisms, emphasizing ASF as a systemic disease.
   - Revealing the pleiotropic effects of ASF resistance, linking it to health, production, and reproductive traits, and providing insights into broader biological implications.
3. **Innovative Tools for Precision Breeding**: We developed a robust polygenic resistance score (APRS) that effectively distinguishes ASF-resistant and susceptible individuals. This predictive tool has transformative potential for breeding ASF-resistant pig populations, with implications that extend to other species.

These findings not only advance our **understanding of ASF resistance mechanisms** but also provide **actionable strategies for breeding programs**, offering a pathway toward sustainable swine production

and global food security. Our work represents a significant leap forward in addressing a global agricultural crisis with innovative, scalable solutions.

This research, representing original and unpublished work, sets the stage for future breakthroughs in ASF resistance and immune trait management. We are confident that our study makes a meaningful contribution to the fields of animal health, genetics, and disease resistance, aligning with the interdisciplinary and global scope of ***GigaScience***. We appreciate your consideration of our manuscript and look forward to the opportunity to share our work with the scientific community.

Sincerely,

Zhen Wang, Ph.D.
Professor of Animal Genetics
College of Animal Sciences, Zhejiang University, Hangzhou, China.
E-mail: wangzhen20@zju.edu.cn
