# Supplementary material for: Integrated multi-omics profiling identifies genetic loci of African swine fever resistance in pigs
Source: Gigascience. 2026 May 30;15:giag066. doi: 10.1093/gigascience/giag066 (PMC13289747; doi:10.1093/gigascience/giag066)
Supplement: giag066_GIGA-D-26-00031_revision_1 [file giag066_giga-d-26-00031_revision_1.pdf]

GigaScience

Integrated multi-omics profiling identifies genetic loci of African swine fever resistance in pigs

--Manuscript Draft--

**Manuscript Number:**	GIGA-D-26-00031R1
**Full Title:**	Integrated multi-omics profiling identifies genetic loci of African swine fever resistance in pigs
**Article Type:**	Research
**Funding Information:**	Agricultural Variety Improvement Project of Shandong Province (2021LZGC001) — Prof. Zhen Wang<br>National Natural Science Foundation of China (31941007) — Prof. Yuchun Pan
**Abstract:**	African swine fever (ASF) remains a persistent threat to global pig production, with no licensed vaccines or effective treatments available. Observations of surviving individuals within low-virulence infected herds suggest that host genetic resistance plays a crucial role. Here, we present a multi-dimensional integrative analysis to uncover host genomic variants associated with ASF resistance. Combining genome-wide association studies (GWAS), genetic differentiation, and functional genomic approaches, including TWAS, SMR, colocalization, and Bayesian network GWAS, we prioritized 135 high-priority candidate resistance genes from an initial gene set of 1,102 candidates. These prioritized genes are enriched in immune-related pathways, such as chemokine signaling and IL-15-mediated activation. Heritability enrichment and transcriptomic analyses further revealed tissue- and cell-type-specific expression patterns, particularly in peripheral immune organs and pulmonary alveolar macrophages. Dynamic infection-responsive genes, including CXCL10, CXCL11 and IL15, exhibited robust antiviral signatures, which highlighted Mac_CD163 as key cellular mediators in the immune response to ASF. Moreover, multiple genes (such as SOS1, FCGR2B, FCGR3) converged on the PI3K-AKT and Fcγ receptor signaling axes pathways, underscoring their functional importance. Finally, we developed a polygenic resistance score using 40 prioritized independent SNPs, which effectively discriminates phenotypic outcomes and showed a positive correlation with health traits such as platelet distribution width. These findings provided a genomic foundation for the precision breeding of ASF-resistant pigs and inform host-targeted disease control strategies.
**Corresponding Author:**	ZHEN WANG<br>Zhejiang University<br>Hangzhou, Zhejiang CHINA
**Corresponding Author Secondary Information:**	
**Corresponding Author's Institution:**	Zhejiang University
**Corresponding Author's Secondary Institution:**	
**First Author:**	Xiaowei Ye
**First Author Secondary Information:**	
**Order of Authors:**	Xiaowei Ye
	Qinqin Xie
	Caiyun Cao
	Shuang Liu
	Wenbo Sun
	Zhe Zhang

Powered by Editorial Manager® and ProduXion Manager® from Aries Systems Corporation

	Qishan Wang
	Yuchun Pan
	Zhen Wang
**Order of Authors Secondary Information:**	
**Response to Reviewers:**	Please refer to the Response file.<br><br>Reviewer #1:<br>Line 70-71: It would be good to clarify that the project losses for the US are hypothetical if ASFV ever arrived in the US (which it has not yet). As written, this is not clear.<br>Response: Thank you for this critical clarification. We agree that the previous phrasing did not sufficiently distinguish between historical costs and hypothetical projections. We have revised the text to explicitly state that the $50 billion figure for the US is an estimated loss based on a simulated outbreak scenario where the disease persists for ten years and halts exports.<br>Revision excerpts: In lines 70-74, we changed the following sentence<br>"…, while projections for 2023 suggested potential losses of 2.5 billion USD in Australia and up to 50 billion USD in the United States." to<br>"For 2023, projections suggested potential losses of 2,500 million USD in Australia 7 and, in the event of an outbreak, economic modeling estimates potential losses of up to 5,000 million USD in the United States, assuming the disease persists for ten years and leads to a prolonged suspension of exports 8."<br><br>Line 101: This is the start of a passage that states "to explore this hypothesis" in reference to the previous paragraph, but there is no hypothesis proposed before this statement. Please clarify the text here to clearly state the objectives of the study.<br>Response: Thank you for pointing this out. We have rephrased the transition to clearly state the objectives of the study. Instead of referring to a "hypothesis", we now specify that our goal was to systematically identify the genetic basis of resistance traits in indigenous pig breeds.<br>Revision excerpts: In line 130, we changed the following sentence "To explore this hypothesis, …" to "To systematically identify the genetic basis of these resistance traits, …"<br><br>Line 113-116: The text states, "our study bridges variant discovery with functional validation and predictive modeling, offering a more mechanistic understanding of ASF resistance." The authors need to be very careful here as neither functional no mechanistic data is used in this study. Only gene expression and GWAS data are used in the presented analyses. No assays that directly address viral or host immune function or gene function through knockout, enzymatic or true functional assays are used in this study. I understand what the authors are trying to say, but the analyses conducted are not functional or mechanistic, but instead based on functional enrichment or integrative, high-throughput omics analyses which is not the same as the targeted molecular biology assays implied by the text. Please update the language throughout to differentiate between causal statistical inference and candidate causal mutations.<br>Response: We sincerely thank the reviewer for this critical distinction. As suggested, we have thoroughly revised the manuscript to avoid the terms "functional validation" and "mechanistic understanding" when referring to our bioinformatic predictions. We have replaced these with more accurate terms such as "functional enrichment", "integrative prioritization", and "multi-dimentional inference". We have also clarified the distinction between candidate causal mutations and statistically inferred signals throughout the text.<br>Revision excerpts: In lines 143-144, we changed the following sentence<br>"…, our study bridges variant discovery with functional validation and predictive modeling, offering a more mechanistic understanding of ASF resistance." to<br>"…, our study bridges variant discovery with functional analyses and predictive modeling, offering more biological insights of ASF resistance."<br>We added "potential" before every instance of "causal" (e.g. lines 214, 223).<br>We changed "validation" to "inference/permutation/evaluation" (e.g. line 184).<br>We changed "validate" to "support/substantiate" (e.g. line 213).<br>We changed "mechanism" to "basis/signature/pathway" (e.g. line 342).

Powered by Editorial Manager® and ProduXion Manager® from Aries Systems Corporation

…

Line 151-172: Regarding the TWAS and SMR analysis, are these two methods truly independent analyses? They both appear to rely on the same pigGTEx gene expression data. Does this mean they destined to generate similar results because similar input information is used in these analyses? Or are they actually using different datasets? Please explain how these analyses compliment each other and provide unique (and not common) information in their results.

Response: Thank you for this insightful question regarding the relationship between TWAS and SMR. We agree that both methods utilize the PigGTEx expression data and our GWAS summary statistics. However, they are methodologically distinct and complementary rather than redundant, providing different layers of genetic evidence:

Different statistical principles: TWAS aggregates the effects of multiple SNPs into a single gene-level test. It aims to identify associations between the predicted expression levels of a gene and the resistance trait, which enhances power to detect genes with multiple weak eQTLs 1. SMR focuses on identifying whether the top eQTL and the top GWAS SNP at a locus share the same underlying causal variant using a Mendelian Randomization framework 2.

Complementary insights (association vs. inferred mediation): TWAS is an association-based tool that captures the overall genetic correlation at the gene level 1. SMR, when combined with HEIDI/Colocalization tests, is specifically designed to distinguish pleiotropy (a single variant affecting both gene expression and resistance trait) from linkage (different variants in LD) 2. TWAS cannot easily distinguish between these two scenarios.

Unique information: In our study, TWAS helped us identify genes where the cumulative effect of cis-variants is significant, even if no single SNP reaches genome-wide significance. SMR and colocalization provided a more stringent filter to prioritize “high-confidence” candidate genes (like PPEF2) where the evidence suggests a potential shared causal signature.

In summary, TWAS provides a broader screen for gene-trait associations, while SMR/colocalization provides a locus-specific refinement to prioritize candidate genes with evidence of shared genetic control. We have added a brief clarification in the Results and Methods section to explain how these two approaches complement each other.

Revision excerpts:

Result:

In lines 192-194, we added the following sentence

“To prioritize candidate genes based on the aggregate association between predicted expression levels and ASF resistance, we first performed TWAS across 34 tissues 31 (Supplementary Table S6).”

In lines 209-210, we added the following sentence

“In parallel, to identify high-confidence genes by testing the potential mediation effects of specific top eQTLs, we employed SMR and colocalization analysis.”

Method:

In lines 834-839, we changed the following sentence

“To evaluate the association between our candidate genes and ASF across various tissue types (including 34 tissues from PigGTEx, Supplementary Table S6) and to elucidate their functional roles, we conducted transcriptome-wide association studies (TWAS) analysis using the online tool FarmGTEx TWAS-server (https://twas.farmgtex.org/). Genomic loci demonstrating significant associations were identified by retaining those with a false discovery rate (FDR) < 0.05.” to

“To prioritize candidate genes by aggregating the cumulative effects of multiple cis-variants on predicted expression levels, we conducted TWAS analysis. Using the FarmGTEx TWAS-server 134 (https://twas.farmgtex.org/), we evaluated associations between candidate genes and ASF resistance across 34 tissues from PigGTEx 31 (Supplementary Table S6). Significant associations were identified using a false discovery rate (FDR) < 0.05.”

In lines 842-843, we added the following sentence

“To identify potential regulatory links by testing the mediation effect of top eQTL signals on ASF resistance, we employed the SMR framework.”

In lines 849-850, we added the following sentence

“To further refine prioritized genes by distinguishing shared genetic control from mere genomic linkage, we performed colocalization analysis.”

	Line 152: What does “prioritized genes” mean in this case? It looks like these are the top genes based on FDR significance from the TWAS. How are the genes identified based on muscle and liver gene expression justified in the context of ASFV? What biological roles do these tissues play in ASFV infection or how do they change in ASFV that would provide signal for incidence of ASF? Please update the text accordingly.<br>Response: Thank you for these insightful comments. We have addressed the concerns regarding the definition of “prioritized genes” and the biological relevance of liver and muscle:<br>We have clarified in the revised text that “prioritized genes” refers to the 135 high-priority candidates identified through our integrated prioritization framework (integrating TWAS, SMR, Coloc, etc.). The 11 genes mentioned in the TWAS section are those from this prioritized set that also reached significance in the TWAS analysis. We have updated the terminology to ensure consistency.<br>Regarding the biological justification for liver and muscle signals, we have updated the text to reflect the known pathogenesis of ASFV. ASFV predominantly infects macrophages and monocytes, but also targets secondary cells such as vascular endothelial cells, hepatocytes, and epithelial cells 3. The liver serves as a core replication hub (targeting Kupffer cells and hepatocytes) 4, while muscle signals likely reflect the physiological response of secondary target cells (e.g., microvascular endothelium 5) and the infiltration of primary target cells during systemic infection. This biological context justifies why gene expression variations in these tissues are statistically linked to the ASF resistance phenotype.<br>We have added these biological justifications to the Results section to better contextualize our findings.<br>Revision excerpts: In line 182, we added the “(hereafter referred to as “prioritized genes”)” after “high-priority candidate genes”.<br>In lines 194-195, we changed the following sentence “This analysis revealed significant tissue-specific associations for 11 prioritized genes across muscle, liver, blood, and intestinal tissues” to<br>“This analysis revealed that 11 of the 135 prioritized genes exhibited significant tissue-specific associations.”<br>In line 197-203, we added the following sentence “… (Fig. 3c, Supplementary Fig. S3 and Supplementary Table S7). The liver is a primary site of viral replication, involving both resident Kupffer cells and hepatocytes 36, whereas signals in muscle likely reflect the systemic nature of infection, capturing the responses of secondary target cells, such as vascular endothelium 37, alongside the infiltration of primary target cells like monocytes and macrophages 38. These findings align with the systemic pathology of ASFV, where genetic variation in both primary and secondary target tissues modulates overall host resistance. ...”<br><br>Line 164: The text states, “confirming the presence of shared causal variants associated with ASF resistance (Fig. 3d).” I can appreciate that causal statistical modeling is used within the analyses of this manuscript. However, it is very important to note there is not evidence presented to determine that these are causal genes. Causal genes and causal variants can only be identified through specific functional molecular biology assays linking specific gene functions to trait phenotypes (e.g., knockouts, gene editing, enzymatic assays, RNAi, etc. that alter sub processes of trait phenotypes or the trait phenotype itself). No such biological evidence is presented so the identified variants cannot be called causal variants. What is presented are enrichment or conditioned statistical tests to provide a probability that a gene may be a causal variant (i.e., to identify candidate causal variants). Please update the text where appropriate throughout the manuscript.<br>Response: We sincerely thank the reviewer for this critical comment regarding the use of the term “causal”. We completely agree that while colocalization analysis provides strong statistical support for shared genetic signals, definitive identification of causal genes or variants requires direct functional validation, which is beyond the scope of the current statistical study. In accordance with your suggestion, we have thoroughly revised the manuscript to ensure a more precise use of terminology.<br>Revision excerpts: In line 213-214, we changed the following sentence<br>“Colocalization analysis further validated 42 of these pairs, confirming the presence of shared causal variants associated with ASF resistance.” to<br>“Colocalization analysis further supported 42 of these pairs, suggesting the presence of shared potential causal variants associated with ASF resistance (Fig. 3d).”

In lines 178, 237, and 241, we changed “validation methods” to “lines of evidence”.
In line 182, we added “candidate” to “high-priority candidate genes”.
In line 208, we changed “Causal relationships identification via SMR and colocalization” to “Inference of putative regulatory genes via SMR and colocalization”.
In line 224, we changed “Causal effects of ASF-resistance gene validating via BN-GWAS analysis” to “Regulatory network inference via BN-GWAS”.
In line 231, we changed “direct or indirect causal” to “putative regulatory”.
In line 235, we changed “validation” to “cross-evaluation”.
In line 445, we changed “functional validation” to “functional annotation”.
In line 462, we changed “causally influence” to “potentially mediate”.

Line 175: Please explain in the context of ASFV how muscle, brain, embryo and liver gene expression data is relevant. We know from previous studies that immune tissues are relevant and that CD163 in cell types such as macrophage are important in ASFV, but do we know similar information for the other tissues?
Response: We appreciate the reviewer’s comment regarding the tissue selection for BN-GWAS. Our choice was guided by two primary considerations:
Statistical reliability: Bayesian Network (BN) inference is highly sensitive to sample size 6. To avoid false-positive regulatory links and ensure model stability, we restricted our analysis to tissues in the PigGTEx database with sample size > 300. Other immune-related tissues with smaller cohorts were excluded to maintain the rigor of the gene prioritization framework.
Systemic Nature of ASFV: While macrophages are the primary targets, ASFV infection is a systemic disease characterized by a cytokine storm, vascular damage, and multi-organ failure 7,8. Liver and blood: As the reviewer noted, these are primary sites for viral replication and immune response 4. Brain and embryo: ASFV can cross the blood-brain barrier and the placental barrier, leading to neurological symptoms and abortion, which are both hallmark clinical signs of acute ASF 7,8. Muscle: As a highly vascularized tissue, it serves as a sentinel for systemic inflammation and microvascular integrity 5.
By using high-quality data from these diverse tissues, we aimed to capture the broad regulatory landscape of the host’s response. We have updated the text to clarify this rationale.
Revision excerpts:
In lines 226-231, we changed “Using RNA-seq data from five tissues (muscle, blood, brain, embryo, and liver) with sample sizes exceeding 300, …” to
“To ensure robust causal inference, we utilized RNA-seq data from five tissues (muscle, blood, brain, embryo, and liver) with sample sizes exceeding 300, as sufficient power is critical for stable expression imputation. These tissues represent both primary viral replication sites (liver and blood) and organs that reflect the systemic physiological disruptions caused by ASFV 42,43.”

Line 187: Please list the 3 specific validation methods here to help the reader as there is so much information presented, it’s hard to keep the different analyses used clear.
Response: We appreciate the reviewer’s suggestion to clarify the specific methods for these 13 genes. Because these genes were prioritized based on different combinations of the five analytical lines (including reported immune genes, TWAS, SMR, colocalization, and BN-GWAS), listing every unique combination in the text would be cumbersome for the reader. To address this, we have:
Updated the text to clarify that these genes are supported by varying combinations of three evidence lines.
Added a reference to Supplementary Table S4 in line 243 to explicitly check off which specific methods support each gene. This allows the reader to easily track the exact evidence for any gene of interest at a glance.
Revision excerpts: In line 241-243, we changed the following sentence
“… were supported by three validation methods, highlighting their robustness (Fig. 3a).” to
“… were supported by three independent lines of evidence, such as combinations of TWAS, SMR, colocalization, highlighting the robustness of their prioritization (Fig. 3a and Supplementary Table S4).”

Line 198-201: The text states, “Additional, signaling by KIT in disease (FDR) and lymphocyte number (FDR) emerged as key pathways potentially driving immune responses against ASF. These findings reinforce the involvement of specific immune

Powered by Editorial Manager® and ProduXion Manager® from Aries Systems Corporation

signaling mechanisms in ASF resistance." I'm not sure what you are trying to say in the second sentence here. If the intent is to say this is a new signaling pathway identified, please make this more clear in the text. If the intent is to say this shows that immune functions are enriched and this is an example, then I recommend deleting this sentence as that is obvious from the result.

Response: We appreciate the reviewer's comment. Our intent was not to state the obvious fact that immune functions are enriched, but rather to highlight specific regulatory signatures that are critically relevant to ASFV pathology. We have revised the text to collectively present these key pathways, emphasizing their strong alignment with established ASFV-induced pathologies. Specifically, the enrichment of pathways like CXCR/MAPK signaling and lymphocyte number directly reflects the host's attempt to regulate the inflammatory response and counteract the profound lymphopenia characteristic of acute ASF. This consolidation clarifies how our prioritized genes are biologically relevant to the known infection mechanisms of the virus.

Revision excerpts: In lines 249-256, we changed the following sentence

"Notably, pathways such as CXCR chemokine receptor binding (FDR = 1.7×10-3), MAP kinase activity (FDR = 2.3×10-2), and interleukin-15 signaling (FDR = 3.2×10-2) were among the most significantly enriched. These pathways, previously implicated immune gene prioritization, underscore the critical roles of chemokine signaling, MAP kinases, and IL-15 in mediating ASF resistance. Additionally, signaling by KIT in disease (FDR = 4.9×10-2) and lymphocyte number (FDR= 7.7×10-15) emerged as key pathways potentially driving immune responses against ASF. These findings reinforce the involvement of specific immune signaling mechanisms in ASF resistance."to

"Notably, the most enriched functional clusters, including CXCR chemokine receptor binding (FDR = 1.7×10-3), MAP kinase activity (FDR = 2.3×10-2), interleukin-15 signaling (FDR = 3.2×10-2), and lymphocyte number (FDR= 7.7×10-15), exhibit high concordance with the characterized pathological features of ASFV infection, particularly regarding the systemic inflammatory cascades and the profound lymphopenia that define acute ASF 46–48. By pinpointing these pathways, our results underscore the critical role of maintaining immune homeostasis and cellular signaling integrity in mediating host resistance to ASFV."

Line 211-213: This text discusses potential cross-tissue resistance networks. Is this biologically founded? Correlations can be founded across many types of data despite a lack of causal relationships. Are there other examples of this in other biological processes? For instance, are there extracellular signaling molecules identified that would make sense as cross-tissue signaling molecules? Alternatively, maybe this passage can be worded in a slightly different way to highlight this may be worth future investigation. The best place to address this may be at lines 433-435 in the discussion.

Response: We thank the reviewer for this insightful comment. ASFV infection is a systemic disease characterized by a "cytokine storm" and multi-organ dysfunction 7, providing a biological basis for our findings.

The pathology of ASF is widespread: the brain is affected by neuro-inflammation (leading to apathy and depression), the heart by epicardial hemorrhage, and the intestines by severe mucosal damage7,9. We identified several prioritized genes, such as CXCL10 and IL15, which encode circulating cytokines/chemokines. These molecules act as systemic signals that coordinate the immune response across tissues, regardless of local viral replication levels 10,11. We believe these findings provide a valuable starting point for understanding the systemic nature of ASF.

We have revised the manuscript to frame the "cross-tissue resistance network" as a hypothesis where genetic variation modulates systemic homeostasis and inter-organ communication to ASF resistance.

Revision excerpts:

Result: In lines 260-266, we changed the following sentence

"Significant heritability enrichment was observed in PAM subtypes (Mac_CD163 and Mac_PLBD1) and the small intestine (including jejunum and ileum) (Fig. 4a and Supplementary Table S12). As the primary target of ASF, PAMs play a central role in infection 12, while the intestine is vital for mucosal immunity and host-microbiota interactions that may influence viral attachment and invasion 13. These results align with established ASF infection mechanisms 7,12. Interestingly, enrichment in non-traditional tissues such as the brain, heart, and milk suggested a potential cross-tissue resistance network, highlighting broader systemic contributions to ASF resistance beyond primary immune tissues." to

"Significant heritability enrichment was observed in PAM subtypes (Mac_CD163 and

Powered by Editorial Manager® and ProduXion Manager® from Aries Systems Corporation

	Mac_PLBD1) (Fig. 4a and Supplementary Table S12), consistent with the established role of PAMs as the primary targets of ASFV infection 49. Interestingly, heritability enrichment was also detected in non-immune tissues, including the small intestine, brain, heart, and milk. This suggests that genetic contributions to ASF resistance may extend beyond primary immune sites, potentially reflecting a systemic, cross-tissue defensive architecture."<br>Discussion: In lines 496-518, we changed the following sentence<br>"ASFV infection manifests as a severe systemic disease, marked by cytokine storm and extensive hemorrhages and lesions across multiple organs, such as apathy, anorexia, depression (brain), epicardium hemorrhage, hydropericardium (heart), diarrhea (intestinal), orchitis, and abortion (reproductive organs)14. Our time-course transcriptomic data confirm a widespread host response, with the majority of prioritized genes (127/132) exhibiting dynamic expression changes post-infection. While these responses were most prominent in lymphoid tissues, such as mandibular lymph nodes, tonsils, and mesenteric lymph nodes, underscoring the central role of immune organs, viral quantification revealed a distinct spatial pattern. Specifically, tissues not primarily linked to immune functions harbored substantially lower ASFV RNA levels compared to primary targets like the spleen and PAMs. Consequently, the elevated expression of immune-related genes in these non-target tissues likely reflects systemic inflammatory signaling rather than significant local viral replication, for example, several prioritized genes (e.g., CXCL10, IL15) encode secreted proteins that could potentially serve as mediators for such cross-tissue communication. The detection of low-level virus in these regions is plausibly due to secondary seeding via viremia as the infection progresses. The heritability enrichment in non-immune organs like the intestines, brain, and heart suggests the existence of a cross-tissue resistance network that extends beyond canonical immune sites. The intestine is vital for mucosal immunity and host-microbiota interactions that may influence viral attachment and invasion 13. Notably, approximately 27% of the prioritized genes (36/132), including ADD1, ZNF family members, and RUSF1, were broadly expressed across tissues, providing molecular support for a systemic and interconnected host defense mechanism." to<br>"ASFV infection triggers a severe systemic crisis, characterized by a cytokine storm and multi-organ hemorrhaging 42,43,92. Clinical manifestations such as depression (brain), hydropericardium (heart), and diarrhea (intestines) underscore the widespread nature of the disease 42,43,92. Our time-course transcriptomic data supports this systemic involvement, with the majority (127/132) of prioritized genes exhibiting dynamic expression changes post-infection. While these responses were most robust in lymphoid tissues, viral quantification revealed that non-canonical tissues harbored substantially lower ASFV RNA levels compared to primary targets like the spleen or PAMs. Consequently, the gene expression changes in these non-target tissues likely reflect physiological responses to systemic inflammatory signaling rather than local viral replication. This is supported by the identification of several prioritized genes such as CXCL10 and IL15, which encode secreted proteins that can act as systemic mediators of inter-organ communication 35,93. The presence of low-level viral RNA in these tissues is likely a secondary effect of viremia. Crucially, the significant heritability enrichment observed in non-immune organs, including the intestines, brain, and heart, provides genetic support for a resistance network that extends beyond canonical immune sites. Furthermore, approximately 27% (36/132) of the prioritized genes (e.g., ADD1, ZNF family members, and RUSF1) were broadly expressed across all examined tissues. Together, these findings suggest that ASF resistance is not solely a localized immune event but involves a multi-organ genetic architecture that maintains systemic integrity. Such a cross-tissue network, potentially involving mucosal immunity in the intestine 94 or neuro-endocrine-immune crosstalk 95, warrants further functional investigation to fully elucidate the basis of host resistance."<br><br>Line 249-252: What is the biological significance of these relationships between the viral gene expression and the 4 host transcripts listed? Please provide more explanation here of the context or meaning of the result. Is this meant to indicate these genes may play a role in host response?<br>Response: We appreciate the reviewer's request for a clearer explanation of the biological context of these results. Our analysis was designed to bridge the gap between genetic prioritization and biological function through a three-step logic:<br>Genetic priority & resistance: These genes were first identified as prioritized candidates influencing ASF resistance based on aggregate genetic evidence (TWAS / SMR / colocalization / BN-GWAS).

	Infection response: Their significant differential expression across multiple tissues confirms their active role in responding to ASFV, rather than being mere bystanders.<br>Viral coupling & biological context: The correlation with viral transcript abundance provides a “abundance-dependent response” dimension to their function: Positive Correlations: These genes (e.g., CXCL10, IL15, CASP6) represent the escalation of the host’s defensive and inflammatory signaling. As viral replication intensifies, the host ramps up these pathways, which often leads to a pathological cytokine storm and extensive cell death in the case of ASFV. Negative Correlations: These genes (e.g., IRS2, RUSF1) represent the progressive depletion of essential host functions. Their decline in direct proportion to viral increase reflects the systemic metabolic collapse and functional deprivation caused by the virus. On “contradictory” signals (e.g., autophagy/metabolism): The presence of both positive and negative correlations within the same functional category reflects the biological tug-of-war between host-mediated clearance and viral-driven subversion.<br>By demonstrating that these genes’ expression levels are precisely synchronized with viral transcript abundance, we provide strong evidence that they are core components of the host-pathogen interface and key drivers of the resistance phenotypes observed. We have updated the manuscript to incorporate these biological insights.<br>Revision excerpts: In lines 298-308, we changed the following sentence<br>“To explore host-pathogen interactions, a Spearman correlation was performed, identifying positive associations between viral RPM and the transcriptional expression (TPM) of GALM, CXCL10, IL15, and CXCL11 in several tissues (Fig. 5b).” to<br>“To further characterize host–pathogen interactions, we performed Spearman correlation analysis between viral transcript abundance (RPM) and host gene expression (TPM). A subset of consistently upregulated genes, including those involved in host defense and immune signaling (e.g., LRPPRC 52, CXCL10, CXCL11 53, CASP6 54, IL15 35), showed strong positive correlations with viral RPM (Fig. 5b). In contrast, consistently downregulated genes involved in critical host functions, such as transcriptional regulation (CUX1 55) and metabolic homeo stasis (IRS2 56), exhibited significant negative correlations (Fig. 5b). This coordinated, abundance-depe ndent response indicates that these prioritized genes are not merely reactive but are likely integral to ASFV-driven biological processes, representing key determinants of host resistance and disease severity.”<br><br>Line 258: Which “prioritized genes” are referred to here? There are so many lists of prioritized genes, it is hard to keep track of them all.<br>Response: We appreciate the reviewer’s comment regarding the clarity of our gene lists. To avoid confusion, we have clarified that the “prioritized genes” analyzed in the single-cell data refer to the final consensus set of 135 high-confidence candidates identified by integrating multiple genomic lines of evidence (e.g., TWAS, SMR, colocalizatin, and BN-GWAS), as defined in line 182.<br>Due to the inherent sparsity of scRNA-seq data, 127 out of these 135 genes passed our quality control and stable expression filters. We have updated the text and the legend of Fig. 4e to explicitly state that this analysis focuses on this 127-gene prioritized subset. This ensures consistency between our integrated genetic findings and the single-cell validation. We have also provided the full list of these 127 genes in Supplementary Table S17 for better tracking.<br>Revision excerpts:<br>Result: In lines 310-316, we changed the following sentence<br>“Single-cell RNA-seq analysis in PAMs (target cells for ASFV) identified 127 prioritized genes in various celltypes post-infection. The Mac_CD163 cells, a transcriptionally distinct PAM subpopulation characterized by high baseline expression of CD163, MARCO, S100A8, and S100A9, exhibited sustained upregulation of the prioritized genes throughout ASFV infection (Fig. 4e and Supplementary Table S16).” to<br>“Single-cell RNA-seq analysis of PAMs 49 (target cells for ASFV) identified 127 prioritized genes from a subset of 135 total prioritized genes, filtered for stable expression in the scRNA-seq dataset (Supplementary Tables S1 and S17). Among various celltypes post-infection, the Mac_CD163 cells, a transcriptionally distinct PAM subpopulation characterized by high baseline expression of CD163, MARCO, S100A8, and S100A9 49, exhibited sustained upregulation of 127-gene prioritized module throughout ASFV infection course (Fig. 4e and Supplementary Table S16).”<br>Figure legend: In lines 1612-1614, we changed the following sentence<br>“Scoring of prioritized gene sets pre- and post-infection in PAM scRNA-seq. …” to<br>“Module scoring of the prioritized gene subset in PAM scRNA-seq. The score

Powered by Editorial Manager® and ProduXion Manager® from Aries Systems Corporation

	represents the aggregate expression difference of 127 prioritized genes (a detectable subset of the 135 total prioritized genes) pre- and post-infection. …”<br><br>Line 254-274: Are these results novel or consistent with past studies? I apologize if I've missed commentary on this aspect of the identified results in the discussion.<br>Response: We thank the reviewer for the opportunity to clarify the novelty of our single-cell analysis. While we utilized a published scRNA-seq dataset 12 and adopted its established cell-type annotations, our findings provide novel insights distinct from the original study:<br>New genetic context: The original study focused on a general transcriptomic overview of ASFV infection. In contrast, our analysis overlayed a genetically-prioritized resistance gene set (135 genes) onto this cellular framework to identify specific “resistance modules”.<br>Novel candidates: We identified 127 prioritized genes as key cellular responders, highlighting candidates like CXCL2, CXCL10, TMCC3, and PHF21B that were not previously emphasized as resistance-related in the original publication.<br>Biological link: Our analysis bridges the gap between systemic genetic heritability and single-cell behavior, showing that the resistance alleles identified in our study are specifically active in the Mac_CD163 subpopulation.<br>We have refined the Results and Discussion sections to more clearly articulate how our targeted analysis of prioritized genes builds upon and extends the existing cellular knowledge of ASF.<br>Revision excerpts:<br>Result: In lines 321-323, we changed the following sentence<br>“Differential expression analysis further revealed that 68 prioritized genes were significantly differentially expressed in at least one cell type at specific time point.” to<br>“Our differential expression analysis specifically focused on this gene set further revealed that 68 prioritized genes were significantly modulated in at least one cell type at specific time points ...”<br>In lines 331-333, we changed the following sentence “…, potentially reflecting virus-induced suppression mechanisms targeting host immune pathways.” to<br>“…, potentially reflecting virus-induced suppression signatures targeting these genetically prioritized host pathways.”<br>Discussion: In lines 521-537, we changed the following sentence<br>“Consistently, our heritability enrichment analysis identified Mac_CD163 and Mac_PLBD1 subtypes as critical mediators of resistance mechanisms. Consistent with Zheng et al.'s findings, the Mac_CD163 subpopulation is pivotal in the early host defense against ASFV, characterized by a massive transcriptomic response and dynamic population shifts. Despite an initial sharp decline in prevalence post-infection, this subset demonstrated a remarkable ability to restrict viral replication, with a consistently low proportion of cells carrying a high viral load. This coordinated response, featuring profound DEG enrichment and effective viral control, establishes Mac_CD163 cells as a key mediator of innate antiviral immunity. Single-cell transcriptomic profiling revealed that robust and sustained activation of Mac_CD163 in response to ASFV infection, with CXCL2 and CXCL10 genes showing continuous upregulation in these cells, reinforcing their roles as key innate immune effectors. Basal expression analysis also identified genes such as CXCL2, CXCL7, and BTC as exhibiting high specificity to PAM, which are critical sites for viral entry and replication. Collectively, these findings emphasize the interplay between tissue-specific and systemic immune responses in driving ASFV resistance.” to<br>“Our study extends this understanding by demonstrating that the heritability of ASF resistance is specifically enriched in macrophage subtypes, particularly Mac_CD163 and Mac_PLBD1. Consistent with Zheng et al.'s cellular landscape 49, we found the Mac_CD163 subpopulation to be pivotal in the host response. Despite their initial sharp decline in prevalence post-infection, this subset demonstrated a remarkable ability to restrict viral replication, characterized by a low proportion of high-viral-load cells 49. Our analysis adds a critical layer to this observation: the robust antiviral capacity of Mac_CD163 cells is coupled with the sustained activation of our prioritized resistance genes. Specifically, the continuous upregulation of CXCL2 and CXCL10 within this subset reinforces their role as key innate immune effectors 97,98. Furthermore, our basal expression analysis identified genes like CXCL2, CXCL7, and BTC as highly specific to PAMs, which are critical sites for ASFV entry 49. By linking these specific genetic candidates to established cellular responders, we show that Mac_CD163 cells are not just transcriptomic markers of infection, but are the primary cellular vehicles

through which these prioritized resistance factors operate. Collectively, these findings emphasize the interplay between tissue-specific genetic architecture and systemic immune responses in driving ASFV resistance."

Line 296: The pleiotropy analysis is interesting, but how comparable is MPS to ASFV infection? There needs to be some discussion of this somewhere in the manuscript. As written, it's unclear if MPS is a relevant trait (thought it would seem it should be).
Response: We appreciate the reviewer's attention to the relationship between MPS and ASF resistance. While both diseases affect the porcine respiratory system, our analysis suggests that their shared genetic basis is highly localized and likely represents linkage rather than pleiotropy.
At the SHISAL1 locus, we observed a high PP3 (0.7153) but a near-zero PP4 (0.0085). This statistical pattern confirms that while both traits have significant signals in this region, they are driven by distinct potential causal variants. Our search of the SHISAL1 functional literature indicates its role in modulating Wnt and FGF signaling pathways, primarily during embryogenesis and head formation 15. There is currently limited evidence linking SHISAL1 directly to broad-spectrum anti-pathogen immunity.
The overlapping signals likely reflect the presence of multiple, independent regulatory elements within this genomic "hotspot" that separately influence lung-related phenotypes. Therefore, despite the superficial similarity between MPS and ASF as respiratory challenges, our results suggest their genetic architectures at this locus are largely independent. We have clarified this distinction in the revised Results and Discussion.
Revision excerpts:
Result: In lines 369-372, we changed the following sentence
"Conversely, distinct causal variants (PP3 > 0.7) were identified for traits like CD4+ leukocyte (GLT8D2, TXNRD1), platelet counts (AUST2), and MPS traits (SHISAL1), highlighting the complex genetic interactions underlying ASF resistance (Fig. 6c)." to
"Conversely, distinct potential causal variants (PP3 > 0.7) were identified for traits like CD4+ leukocyte (GLT8D2, TXNRD1), platelet counts (AUST2), and MPS traits (SHISAL1), indicating genetic linkage, highlighting the locus-specific complexity of the genetic architecture of ASF resistance (Fig. 6c)."
Discussion: In lines 559-570, we added the following sentence
"The overlapping signals for ASF resistance and MPS at the SHISAL1 locus provide insight into multi-trait genetic architecture. Despite both diseases involving respiratory pathology 42,57,104, colocalization analysis strongly supports the PP3 model (PP3 = 0.7153, PP4 = 0.0085), indicating that the associations are driven by distinct potential causal variants in close proximity. This suggests that the SHISAL1 region represents a multi-trait locus with independent regulatory elements modulating responses to different stressors (bacterial for MPS and viral for ASF), rather than a shared genetic signature. Consistent with this, SHISAL1 is primarily implicated in Wnt and FGF signaling during development, rather than canonical immune pathways 105. Together, these findings indicate that the observed overlap reflects local linkage within a complex regulatory landscape rather than true pleiotropy."

Line 315-318: Are these novel findings, or is there past evidence that ASF impacts these traits? (Realizing, you may never know for a trait like meat quality).
Response: We appreciate the reviewer's suggestion to contextualize these findings. While the specific genetic associations identified in our study are novel, they align well with known clinical observations of ASFV infection.
Past studies have documented that chronic or subacute ASFV infection often leads to persistent growth retardation, emaciation (impacting production and meat traits), and reproductive failures such as abortions (impacting reproductive traits) 7.
Our study provides a novel genetic basis for these observations by identifying specific prioritized genes (e.g., TPST1, ABCG5) that are associated with both ASF resistance and these production/reproductive traits. We argue that the extensive genetic overlap and pleiotropic effects typically seen in complex traits 16 provide a theoretical basis for our observation that ASF resistance profiles are intrinsically linked to growth and reproductive traits.
This suggests that the observed clinical symptoms in infected pigs might be partly mediated by the pleiotropic effects or genetic linkage of resistance-related loci. We have updated the Discussion to include this context.
Revision excerpts: In lines 555-559, we added the following sentence
"Complex traits are inherently polygenic, and the finite number of genomic variants

gives rise to widespread genetic overlap and pleiotropy across traits. Many phenotypes are associated with hundreds to thousands of loci, implying the presence of shared causal variants 102. Consistently, genetic correlation analyses indicate that some variants exert concordant effects on multiple traits 103."
In lines 572-584, we added the following sentence
"Beyond immune-related functions, we observed that ASF resistance-prioritized genes are also associated with reproductive (e.g., litter weight) and production traits (e.g., body length). These genetic associations mirror the clinical manifestations of chronic ASFV infection, where affected pigs often exhibit progressive emaciation, growth stunting, and reproductive disturbances such as abortion 42. The involvement of key genes like TPST1 (TPST1-deficient mice experience decreased body weight and reproductive performance 106) and ABCG5 (mediates cholesterol metabolism 107) suggests that the physiological trade-offs observed during infection may have a partially shared genetic architecture. Our findings provide a genetic framework for understanding how disease resistance signatures may intersect with host fitness and performance. This underscores the necessity of balanced selection in breeding programs to enhance antiviral resilience without compromising essential production and reproductive outcomes."

Line 330: Comment 1- It's a nice result the APRS differentiates the pigs into groups. I assume this analysis is conducted with the SNP case-control study population. The population used to test the discriminatory power of the APRS needs to be defined here in the text.
Response: We thank the reviewer for pointing out the need for a clearer definition of the study population. We have now explicitly stated in the Results and Methods section that the discriminatory power of the APRS was evaluated using the original experimental case-control study population (n = 474 pigs), which served as our discovery cohort.
Revision excerpts:
Result: In lines 382-385, we changed the following sentence
"To facilitate the practical implementation of our findings in genetic improvement programs for ASF resistance, we constructed an ASF polygenic resistance score (APRS) based on the ASF-resistance prioritized loci identified in this study." to
"To evaluate the practical implementation of our findings, we constructed an ASF polygenic resistance score (APRS) based on the ASF-resistance prioritized loci identified in this study and tested its performance within our discovery cohort (the experimental case-control population, n = 474)."
Method: In lines 1068-1070, we added the following sentence
"To assess the specificity and robustness of the APRS, we performed a randomized validation within our discovery cohort (the experimental case-control population, n = 474)."

Line 474: How were the SNPs selected within genes when there were multiple SNP? This was unclear to me. There is some explanation in the methods, but this seems lost in the results and discussion.
Response: We appreciate the reviewer's comment regarding the SNP selection process within the prioritized genes. We have now clarified this in the Results and Methods sections. Briefly, our strategy followed a two-step approach: We identified 135 prioritized genes through an integrated multi-dimensional framework. Within the genomic regions of these genes, we selected the lead SNP (based on the lowest p-value) and applied LD clumping to retain 40 independent loci for the final APRS construction. This ensures that the APRS is based on the most statistically significant and independent signals within our high-confidence gene set.
Revision excerpts:
Result: In lines 385-388, we changed the following sentence
"The APRS was defined by aggregating favorable alleles across prioritized loci and calculating each individual pig's score based on its identity-by-state (IBS) similarity to this ideal resistant genotype (Fig. 6d)." to
"The APRS was constructed by aggregating favorable alleles at independent lead loci within prioritized gene regions, with each individual's score calculated based on identity-by-state (IBS) similarity to an ideal resistant genotype (Fig. 6d)."
Method: In lines 1034-1037, we changed the following sentence
"A total of 40 independent loci corresponding to the prioritized genes were selected to define the ideal ASF-resistant genotype." to

Powered by Editorial Manager® and ProduXion Manager® from Aries Systems Corporation

	“A total of 40 independent loci were selected to define the ideal ASF-resistant genotype. Specifically, within 135 high-confidence gene regions, the most significantly associated (p-values) SNPs were first identified as candidate loci. LD clumping (PLINK v1.9 124, --indep-pairwise 50 5 0.1) was then applied to retain independent, non-redundant lead SNPs for inclusion in the final APRS model.”<br><br>Line 331-336: I do not find the randomization analysis useful unless it was being used in the same genomic regions to show selected SNPs outperform those simply in LD. A better comparison would be to compare the discriminatory power of a SNP set identified from a genomic selection model for ASFV resilience. Why would the randomization analysis identify a relationship with ASFV prevalence?<br>Response: We appreciate the reviewer’s professional rigor. However, we would like to clarify the fundamental logic of our APRS construction, which differs from conventional black-box prediction models like Genomic Selection (GS).<br>Why APRS is not redundant with LD signals: While many SNPs in a genomic region may show statistical association due to LD, our 40 loci were not selected solely based on their p-values. They are anchored in 135 prioritized functional genes identified through an integrated framework (TWAS, SMR, colocalization and BN-GWAS). By choosing the lead SNPs from these biologically-relevant regions, the APRS serves as a basically-grounded index rather than a mere statistical aggregator. Our randomization analysis (random genotypes and random loci) confirms that the discriminatory power is specific to this functional gene set, which cannot be achieved by capturing general genomic noise.<br>APRS vs. GS: The primary objective of APRS is not to outperform GS in total genomic prediction accuracy, but to provide a parsimonious and interpretable tool for early screening of high-resistance individuals. Unlike GS, which requires genome-wide high-density markers, our APRS relies on a curated set of 40 functional anchors. This makes it more practical for targeted breeding and demonstrates that our multi-dimensional prioritization successfully condensed complex polygenic resistance into a concentrated genetic score.<br>Revision excerpts:<br>In lines 393-397, we changed the following sentence<br>“Randomized APRS scores failed to differentiate between resistant and deceased individuals (Fig. 6e and Supplementary Tables S21), confirming that the identified loci significantly contribute to ASF resistance and are not influenced by random variation.”<br>to<br>“The randomized scores failed to differentiate between resistant and deceased individuals (Fig. 6e and Supplementary Tables S21), confirming that the the predictive power of APRS arises from biologically prioritized functional loci rather than stochastic inflation, and that it meaningfully contributes to the observed resistance phenotype.”<br><br>Line 338-341: Wasn’t the SNP set identified in the APRS developed using datasets from the PigBiobank? Were different studies used for validation or were the same datasets used for validation that were used to prioritize SNPs within the APRS?<br>Response: We appreciate the reviewer’s request for clarification on the relationship between the datasets. We have clarified this in the revised manuscript.<br>APRS: The SNPs used in APRS were derived from our experimental cohort, but their prioritization was based on a multi-dimensional framework integrating external functional evidence (e.g., TWAS, SMR, and etc., related to eQTL statistics from PigGTEx), ensuring that th...
**Additional Information:**	
**Question**	**Response**
Are you submitting this manuscript to a special series or article collection?	No
**Experimental design and statistics**<br><br><br>Full details of the experimental design and statistical methods used should be given in the Methods section, as detailed in our	Yes

Minimum Standards Reporting Checklist. Information essential to interpreting the data presented should be made available in the figure legends.<br><br>Have you included all the information requested in your manuscript?	
**Resources**<br><br>A description of all resources used, including antibodies, cell lines, animals and software tools, with enough information to allow them to be uniquely identified, should be included in the Methods section. Authors are strongly encouraged to cite Research Resource Identifiers (RRIDs) for antibodies, model organisms and tools, where possible.<br><br>Have you included the information requested as detailed in our Minimum Standards Reporting Checklist?	Yes
**Availability of data and materials**<br><br>All datasets and code on which the conclusions of the paper rely must be either included in your submission or deposited in publicly available repositories (where available and ethically appropriate), referencing such data using a unique identifier in the references and in the "Availability of Data and Materials" section of your manuscript.<br><br>Have you have met the above requirement as detailed in our Minimum Standards Reporting Checklist?	Yes
GigaScience has policies and guidelines in place for the use of generative AI-writing tools such as ChatGPT. If you have used such writing tools to assist with writing the manuscript this must be declared and cited in the text. Authors should not list AI-writing tools and other	No

AI-assisted technologies as an author or co-author and should acknowledge that they are fully responsible for text generated or refined by AI-writing tools.<p><br><br>A summary of use (particularly in the introduction or among methods) needs to be included at the end of the paper, and the outputs should also be included as a supplementary file hosted in GigaDB or other open repositories. Please read our guidelines for more information. <p><br><br>By submitting to GigaScience, you are aware of the journal's AI-writing tools policy, and if you have declared use of such tools below, you have acknowledged this where appropriate in your manuscript and have made a summary of use and outputs available. <p><br><b>AI-assisted writing tools have been used in the preparation of this manuscript?	

Manuscript

Click here to access/download;Manuscript;Main-revised-5.28.docx

Integrated multi-omics profiling identifies genetic loci of African swine fever resistance in pigs

Xiaowei Ye [a], Qinqin Xie [a], Caiyun Cao [a], Shuang Liu [a], Wenbo Sun [b], Zhe Zhang [a, c], Qishan Wang [a, c], Yuchun Pan [a, c, *], Zhen Wang [a, c, *]

[a] Zhejiang Key Laboratory of nutrition and breeding for high-quality animal products, College of Animal Sciences, Zhejiang University, Hangzhou, Zhejiang 310058, China
[b] Shandong Key Laboratory of Animal Disease Control and Breeding, Institute of Animal Science and Veterinary Medicine, Shandong Academy of Agricultural Sciences, Jinan, Shandong 250100, China
[c] Hainan Institute, Zhejiang University, Yongyou Industrial Park, Yazhou Bay Sci-Tech City, Sanya 572000, China

*Corresponding author: Zhen Wang (wangzhen20@zju.edu.cn) and Yuchun Pan (panyc@zju.edu.cn)

Short running title: Resistance loci for African swine fever

E-mail addresses:
XW. Y: ye_xw@zju.edu.cn
QQ. X: qinqin.xie@zju.edu.cn
CY. C: ccyun@zju.edu.cn
S. L: liushuang9917@zju.edu.cn
WB. S: sunwenbo@saas.ac.cn
Z. Z: zhe_zhang@zju.edu.cn
QS. W: wangqishan@zju.edu.cn
YC. P: panyc@zju.edu.cn
Z. W: wangzhen20@zju.edu.cn

ORCIDs:
Xiaowei Ye: 0009-0005-0079-3930
Qinqin Xie: 0009-0001-6678-8078
Caiyun Cao: 0009-0009-2797-6453
Shuang Liu: 0009-0006-9790-412X
Wenbo Sun: 0000-0001-8100-529X
Zhe Zhang: 0000-0001-5320-3125
Qishan Wang: 0000-0002-6475-0009

Yuchun Pan: 0000-0002-1163-5963
Zhen Wang: 0000-0002-1896-3716

Abstract

African swine fever (ASF) remains a persistent threat to global pig production, with no licensed vaccines or effective treatments available. Observations of surviving individuals within low-virulence infected herds suggest that host genetic resistance plays a crucial role. Here, we present a multi-dimensional integrative analysis to uncover host genomic variants associated with ASF resistance. Combining genome-wide association studies (GWAS), genetic differentiation, and functional genomic approaches, including TWAS, SMR, colocalization, and Bayesian network GWAS, we prioritized 135 high-priority candidate resistance genes from an initial gene set of 1,102 candidates. These prioritized genes are enriched in immune-related pathways, such as chemokine signaling and IL-15-mediated activation. Heritability enrichment and transcriptomic analyses further revealed tissue- and cell-type-specific expression patterns, particularly in peripheral immune organs and pulmonary alveolar macrophages. Dynamic infection-responsive genes, including *CXCL10*, *CXCL11* and *IL15*, exhibited robust antiviral signatures, which highlighted Mac_CD163 as key cellular mediators in the immune response to ASF. Moreover, multiple genes (such as *SOS1*, *FCGR2B*, *FCGR3*) converged on the PI3K-AKT and Fcγ receptor signaling axes pathways, underscoring their functional importance. Finally, we developed a polygenic resistance score using 40 prioritized independent SNPs, which effectively discriminates phenotypic outcomes and showed a positive correlation with health traits such as platelet distribution width. These findings provided a genomic foundation for the precision breeding of ASF-resistant pigs and inform host-targeted disease control strategies.

Keywords: African swine fever, multi-omics, disease resistance, pig breeding, host genetics

Introduction

African swine fever (ASF) is a highly contagious and often lethal viral disease that affects both domestic and wild pigs. Caused by the African swine fever virus (ASFV), the disease is characterized by severe hemorrhagic fever, with case fatality rates reaching nearly 100% in acute infections and 30%~70% in subacute or chronic forms [1]. ASF outbreaks have caused devastating losses in swine populations worldwide, posing a substantial threat to global food security. Between 2005 and January 2025, ASF outbreaks were reported in 83 countries [2]. From 2014 to 2017, nearly 8 million pigs in Eastern Europe and the Russian Federation were lost due to ASF [3]. By 2019, the disease had resulted in the culling or death of nearly 5 million pigs in Asia [4]. The economic repercussions of ASF have been staggering. Russia reported losses of 267 million USD during the 2011 outbreaks [5]. In 2022, ASF-related disruptions cost

France's export market an estimated 168~389 million USD [6]. For 2023, projections suggested potential losses of 2,500 million USD in Australia [7] and, in the event of an outbreak, economic modeling estimates potential losses of up to 50,000 million USD in the United States, assuming the disease persists for ten years and leads to a prolonged suspension of exports [8].

The emergence of ASF in China in 2018 had particularly profound effects on the national pig industry, given China's large-scale swine production [9,10]. With nearly half of the global pig population located in China [3,9,11], the outbreak led to dramatic reductions in herd sizes, severe disruptions in pork supply chains, loss of valuable genetic resources, and sharp increases in pork prices [11]. Within a single year (August 2018-July 2019), outbreaks of ASF in China resulted in economic losses exceeding 100,000 million USD [12].

In response, considerable research efforts have focused on understanding ASFV biology, modes of transmission, and the development of effective vaccines and therapeutics [13–15]. However, ASF continues to be the most critical threat to the global pig industry. The absence of commercially licensed vaccines, the virus's ability to persist in diverse environmental reservoirs, and the high genetic variability across ASFV strains greatly complicate control efforts [16–19].

Intriguingly, field observations from recent outbreaks have revealed variable clinical outcomes among pigs within the same herd. While many individuals succumb to infection, some individuals survive and exhibit seroconversion without detectable viremia: testing negative for ASFV antigens but positive for ASFV-specific antibodies. Such findings suggest the existence of potential natural resistance genetic basis, drawing attention to host genetic factors as critical determinants of ASF susceptibility, which likely modulates immune activation, coordination, and regulation to limit immunopathology and promote survival or infection tolerance [20]. In this study, we define "resistance" broadly as the host's capacity to survive infection, encompassing both the ability to limit viral replication and to mitigate disease severity. Host immune responses play a central role in shaping the heterogeneous clinical outcomes of ASFV infection, reflecting complex virus-host interactions. For example, asymptomatic pigs exhibit higher NK cell activity but lower IgA and virus-specific antibody levels compared with susceptible individuals [21]. Functional studies further demonstrate that innate antiviral effectors, such as MxA and IFITM proteins, can directly inhibit ASFV replication [22,23]. This shift in focus from a pathogen-centered to host-centered perspective, presents new possibilities for ASF control strategies. Genetic resistance traits, in particular, offer a sustainable and long-term strategy for ASF management, especially given the current lack of effective vaccines or antiviral treatments. Moreover, identifying and leveraging these traits could aid in the preservation of indigenous pig

breeds, many of which are renowned for their natural resistance to disease.

Current research on ASF host resistance has largely focused on identifying candidate genes, particularly through interspecies comparisons. The contrasting responses between warthogs and domestic pigs represent a well-established model: despite similar viral replication levels, clinical outcomes differ markedly, highlighting a dominant role for host factors [24]. Genomic studies have revealed adaptive divergence in immune-related genes, including *Mx1*, *Mx2*, and *PTGS2*, implicating ASF as a selective pressure in warthog evolution [25]. Comparative and functional analyses have further identified candidate resistance genes such as *ISG15*, *HERC5*, *TRIM21*, and *RELA*, as well as structural variants affecting loci like *LDHB* and TRIM family genes that modulate viral replication [26,27]. In domestic pigs, emerging evidence also supports a genetic basis for variation in ASF outcomes, with distinct disease progression observed across breeds such as Ugandan pigs and Xiang pigs [28,29]. However, most studies rely on limited sample sizes or single-omics approaches, resulting in inconsistent findings and incomplete genetic insight. A comprehensive, large-scale, multi-omics framework is therefore required to systematically resolve the genetic architecture and molecular basis underlying host variation in ASF infection.

To systematically identify the genetic basis of these resistance traits, we conducted integrative genomic analyses combining whole-genome sequencing (WGS) of resistant and susceptible pigs, functional annotation of candidate variants, and transcriptomic profiling using publicly available RNA-seq datasets related to ASFV infection. The analyses leveraged resources from the FarmGTEx consortium, which provides a multi-species framework for transcriptomic and multi-omics profiling in livestock [30]. Within this initiative, PigGTEx [31] systematically characterizes gene expression across diverse tissues and developmental stages in pigs, while PigBiobank [32] extends these efforts by integrating large-scale phenotypic and omics data from multiple pig populations. Together, these datasets offer a comprehensive reference for investigating gene regulation and functional variation in pigs, thereby supporting the identification of genomic variants, biological pathways, and candidate genes underlying resistance to ASF. Unlike previous studies, which primarily focused on association signals, our study bridges variant discovery with functional analyses and predictive modeling, offering more biological insights of ASF resistance, establishing a genomic foundation for selective breeding, genomic prediction, and potentially host-directed ASF control strategies. These results have broad implications for improving swine health and enhancing herd resilience against future ASF outbreaks.

Results

ASF-resistance candidate genes

To characterize genomic differences underlying ASF infection outcomes, we analyzed 474 pigs with antigen/antibody phenotyping and WGS (**Supplementary Table S1**). In a herd naturally exposed to low-virulence ASFV, 108 individuals died, 222 survived, and 144 remained uninfected (**Table 1**). These divergent phenotypes, observed under consistent exposure conditions and supported by longitudinal sampling, indicate a substantial host genetic contribution to disease variability.

For genomic analysis, the WGS dataset yielded 23,290,599 common variants after quality control (minor allele frequency [MAF] > 0.05, **Table 1**). The experimental cohort displays an admixed genetic background, with ancestry predominantly derived from East Asian (specifically East Chinese) pigs, forming a unique cluster distinct from European commercial breeds (**Supplementary Fig. S1)**. PCA within the experimental population showed minimal stratification by breed and extensive overlap among disease phenotypes, indicating weak population structure and no evident confounding by genetic background (**Supplementary Fig. S1**). Together, these results support the suitability of this cohort for genome-wide association studies (GWAS).

To maximize the identification of candidate loci associated with ASF-resistance, individuals were stratified into four groups for comparative analyses, each based on different potential gene-related objectives (**Fig.1**, **Tables 2** and **3**). GWAS and genetic differentiation analyses (fixation index, F_{ST}), combined with allele frequency (AF) tests (hereinafter referred to as the F_{ST} method), were employed to identify loci that may have undergone and contribute to ASF resistance. Ultimately, 1,102 non-redundant genes were identified as significantly associated with ASF resistance (GWAS: p-values $< \frac{1}{Me}$; F_{ST}: p-values $< \frac{0.05}{N}$) (**Fig. 2**, **Supplementary Fig. S2**, **Supplementary Tables S2** and **S3**).

ASF-resistance gene prioritization

To prioritize genes associated with ASF resistance, we assessed 1,102 candidate genes by integrating five independent lines of evidence to assign prioritization scores: reported immune genes (**Supplementary Table S5**), transcriptome-wide association studies (TWAS), summary-data-based Mendelian randomization (SMR), colocalization, and Bayesian network genome-wide association studies (BN-GWAS) (see **Methods**, **Table 4**). As a result, 135 high-priority candidate genes (hereafter referred to as "prioritized genes") were identified based on prioritization scores in the top 10% (⩾ 3.5) or inference by at least two statistical methods (**Fig. 3a** and **Supplementary Table**

S4). Among these prioritized genes, 31 were previously implicated in immune functions, such as members of the chemokine family (*CXCL2*, *CXCL7*, *CXCL10*, *CXCL11*) [33] and the MAP kinase family (*MAP4K3*, *MAPK9*, *MAP2K6*, *MAPK10*) [34], which are integral to immune regulation, inflammatory responses, and cell signaling. Additionally, *IL15* was highlighted for its roles in T and NK cell activation and maintenance of memory $CD8^+$ T cells [35], emphasizing its potential involvement in ASF resistance.

Tissue-specific associations via TWAS

To prioritize candidate genes based on the aggregate association between predicted expression levels and ASF resistance, we first performed TWAS across 34 tissues [31] (**Supplementary Table S6)**. This analysis revealed that 11 of the 135 prioritized genes exhibited significant tissue-specific associations (FDR < 0.05). Notably, *SOS1*, *GAN*, *ZNF394*, *KIT*, and *TMCC3* exhibited positive associations in liver and muscle tissues (**Fig. 3c**, **Supplementary Fig. S3** and **Supplementary Table S7**). The liver is a primary site of viral replication, involving both resident Kupffer cells and hepatocytes [36], whereas signals in muscle likely reflect the systemic nature of infection, capturing the responses of secondary target cells, such as vascular endothelium [37], alongside the infiltration of primary target cells like monocytes and macrophages [38]. These findings align with the systemic pathology of ASFV, where genetic variation in both primary and secondary target tissues modulates overall host resistance. Furthermore, genes including *ZNF713*, *RUSF1*, and *KIT* exhibited negative associations in the blood and intestine (**Fig. 3c**, **Supplementary Fig. S3** and **Supplementary Table S7**). Together, these tissue-dependent contexts provide high-confidence targets for functional prioritization and the development of ASF-resistant breeding programs.

Inference of putative regulatory genes via SMR and colocalization

In parallel, to identify high-confidence genes by testing the potential mediation effects of specific top eQTLs, we employed SMR and colocalization analysis. Using SMR analysis across 34 tissues from PigGTEx [31], we identified 1,490 candidate causal pairs involving 316 candidate genes, with 127 classified as prioritized (**Supplementary Table S6** and **S8**). Colocalization analysis further supported 42 of these pairs, suggesting the presence of shared potential causal variants associated with ASF resistance (**Fig. 3d**). For example, *PPEF2* expression in blood was found to share a potential causal variant with ASF resistance (posterior probabilities under Hypothesis 4, PP4 > 0.75) (**Supplementary Fig. S4**). Functionally, *PPEF2* inhibits ASK1, a MAP kinase involved in apoptosis regulation, and modulates $CD8^+$ cDC1 antigen presentation [39,40], implicating it in immune processes relevant to ASF resistance. Additionally, 41 pairs showed strong posterior probabilities under Hypothesis 3 (PP3 > 0.75), such as *SOS1* (muscle), *SLC3A1* (muscle), *RUSF1* (multiple tissues), *GALM* (milk, adipose), *CXCL11* (liver), and *MVP* (blood, adipose), supporting associations

with distinct potential causal variants (**Fig. 3d** and **Supplementary Table S9**).

Regulatory network inference via BN-GWAS

BN-GWAS [41] was employed to infer causal networks linking candidate genes to ASF resistance. To ensure robust causal inference, we utilized RNA-seq data from five tissues (muscle, blood, brain, embryo, and liver) with sample sizes exceeding 300, as sufficient power is critical for stable expression imputation. These tissues represent both primary viral replication sites (liver and blood) and organs that reflect the systemic physiological disruptions caused by ASFV [42,43]. We identified 12 prioritized genes with putative regulatory effects (**Fig. 3e** and **Supplementary Table S10**). Notable genes include *SOS1* (muscle), *FCGR2B* (brain), and *SDCCAG8* (muscle), which showed positive effects, and *HGSNAT* (brain), *LOC106509841* (blood), and *CLIP2* (blood), which exhibited negative effects.

Integration and cross-evaluation of prioritized genes

Among all prioritized genes, *SOS1* achieved the highest prioritization score (13.7), supported by all five lines of evidence. As a regulator of the MAPK, PI3K/JAK cellular signaling pathways and tumorigenesis [44,45], *SOS1* was identified as having a positive effect on ASF resistance. Additionally, 13 other genes (*RUSF1*, *KIT*, *ZNF713*, *FCGR2B*, *SDAD1*, *MVP*, *CLIP2*, *SLC3A1*, *HGSNAT*, *PHF21B*, *ZNF394*, *CXCL11*, and *SDCCAG8*) were supported by three independent lines of evidence, such as combinations of TWAS, SMR, colocalization, highlighting the robustness of their prioritization (**Fig. 3a** and **Supplementary Table S4**). Notably, *SOS1* (muscle), *RUSF1* (blood, liver, lung), *SLC3A1* (muscle), *ZNF394* (liver) and *FCGR2B* (brain) showed consistent tissue-specific activity.

Enriched pathways of prioritized genes

Pathway enrichment analysis of the prioritized genes revealed 41 immune-related pathways significantly associated with ASF resistance (FDR < 0.05) (**Fig. 3f** and **Supplementary Table S11**). Notably, the most enriched functional clusters, including CXCR chemokine receptor binding (FDR = 1.7×10^{-3}), MAP kinase activity (FDR = 2.3×10^{-2}), interleukin-15 signaling (FDR = 3.2×10^{-2}), and lymphocyte number (FDR= 7.7×10^{-15}), exhibit high concordance with the characterized pathological features of ASFV infection, particularly regarding the systemic inflammatory cascades and the profound lymphopenia that define acute ASF [46–48]. By pinpointing these pathways, our results underscore the critical role of maintaining immune homeostasis and cellular signaling integrity in mediating host resistance to ASFV.

Resistance-associated tissues and cells

To identify tissues and cells associated with ASF resistance, we conducted heritability enrichment analysis across 34 tissues and 8 porcine alveolar macrophage (PAM) subtypes using linkage disequilibrium (LD) score regression. Significant heritability enrichment was observed in PAM subtypes (Mac_CD163 and Mac_PLBD1) (**Fig. 4a** and **Supplementary Table S12**), consistent with the established role of PAMs as the primary targets of ASFV infection [49]. Interestingly, heritability enrichment was also detected in non-immune tissues, including the small intestine, brain, heart, and milk. This suggests that genetic contributions to ASF resistance may extend beyond primary immune sites, potentially reflecting a systemic, cross-tissue defensive architecture.

RNA-seq analyses from ASFV-infected and healthy pig tissues retained 132 prioritized genes after basal expression filtering (TPM > 0.1 in ⩾20% of samples), revealing tissue-specific gene expression patterns, with 41 genes (31.1%) exhibiting high tissue specificity (Tissue-specific gene expression, TAU > 0.8) in peripheral blood mononuclear cells (PBMCs), kidney, and PAM, while 36 genes (27.3%) showed broad expression across multiple tissues (TAU < 0.6, **Fig. 4c**, **Supplementary Fig. S5** and **Supplementary Tables S1** and **S13**), suggesting a general role in systemic immune and inflammatory responses. Moreover, differential gene expression and time-series analyses revealed 127 prioritized genes were significantly differentially expressed (FDR < 0.05, $|\log_2 FC| > 1$) in at least one tissue at various time points post-ASFV infection (**Fig. 4c**). The mandibular (90 genes), tonsil (78 genes), and mesenteric (76 genes) tissues harbored the highest number of responsive genes, consistent with their central roles in lymphoid immune responses [50] (**Supplementary Tables S13** and **S14**). Prioritized genes such as *GALM*, *CXCL11*, *CXCL10,* and *IL15* were consistently upregulated across multiple tissues (**Fig. 4c**, **Supplementary Fig. S5**, **Supplementary Tables S13-S15**), indicating a sustained antiviral immunity activation during ASFV progression. Conversely, genes like *ADD1*, *CLIP2*, *PHF21B* were downregulated (**Fig. 4c**, **Supplementary Tables S13-S15**), potentially reflecting virus-induced suppression of host immune pathways. Notably, *ADD1* plays a critical role in vesicle packaging and viral endocytosis, with its depletion demonstrating antiviral effects [51].

The dynamics of ASFV replication, as quantified by viral RNA (Reads per million, RPM), were both tissue-specific and time-dependent. While all tissues supported progressive viral replication, PAM were distinguished by the earliest and most rapid replication, showing significant accumulation by 4 hours post-infection (hpi). A widespread, marked increase in other tissues became evident by 3 days post-infection (dpi). Spatially, viral abundance was stratified, with the highest titers (≈20,000 RPM) in primary target tissues (PAM, spleen, PBMC). The lung displayed intermediate levels (≈9,000 RPM), while the heart and various lymph nodes (inguinal, mandibular,

mesenteric) exhibited the lowest levels, at several hundred to ~1,000 RPM (**Fig. 5a**). To further characterize host-pathogen interactions, we performed Spearman correlation analysis between viral transcript abundance (RPM) and host gene expression (TPM). A subset of consistently upregulated genes, including those involved in host defense and immune signaling (e.g., *LRPPRC* [52], *CXCL10*, *CXCL11* [53], *CASP6* [54], *IL15* [35]), showed strong positive correlations with viral RPM (**Fig. 5b**). In contrast, consistently downregulated genes involved in critical host functions, such as transcriptional regulation (*CUX1* [55]) and metabolic homeo stasis (*IRS2* [56]), exhibited significant negative correlations (**Fig. 5b**). This coordinated, abundance-dependent response indicates that these prioritized genes are not merely reactive but are likely integral to ASFV-driven biological processes, representing key determinants of host resistance and disease severity.

Single-cell RNA-seq analyses of PAMs [49] (target cells for ASFV) identified 127 prioritized genes from a subset of 135 total prioritized genes, filtered for stable expression in the scRNA-seq dataset (**Supplementary Tables S1** and **S17**). Among various celltypes post-infection, the Mac_CD163 cells, a transcriptionally distinct PAM subpopulation characterized by high baseline expression of *CD163*, *MARCO*, *S100A8*, and *S100A9* [49], exhibited sustained upregulation of 127-gene prioritized module throughout ASFV infection course (**Fig. 4e** and **Supplementary Table S16**). This persistent response highlights their specialized role in orchestrating innate immune defenses within the lung, consistent with prior functional descriptions of this subset [49]. Other subtypes of macrophage cells, such as Mac_HLA_DRA, Mac_CREG1 and Mac_PLBD1 also show high expression of prioritized genes early in the infection (**Fig. 4e** and **Supplementary Table S16**). Our differential expression analysis specifically focused on this gene set further revealed that 68 prioritized genes were significantly modulated in at least one cell type at specific time points (**Fig. 4f**), emphasizing their dynamic roles in ASFV infection and immune modulation. For example, *CXCL2*, highly upregulated in the early phase; *CXCL10*, peaking during the mid-phase; and *PPBP* (also known as *CXCL7*), predominantly upregulated in the mid-to-late phase, across multiple immune cell populations (**Fig. 4f**, **Supplementary Fig. S18** and **Supplementary Table S17**). Conversely, genes such as *TMCC3* (Mac_HLA_DRA), *PHF21B* (Mac_HLA_DRA), *FCGR3* (Mac_CD163), *NTAQ1* (Mac_CD163), *BTC* (Mac_PLBD1) and *PARM1* (Mac_PLBD1) (**Fig. 4f**, **Supplementary Fig. S18** and **Supplementary Table S17**) exhibited consistent downregulation, potentially reflecting virus-induced suppression signatures targeting these genetically prioritized host pathways.

Genetic correlations of ASF resistance with other traits

We assessed the genetic correlations between ASF resistance and 122 traits related to health, growth, and reproduction using publicly available GWAS summary data (121

traits from PigBiobank [32] and one trait concerning Mycoplasmal Pneumonia of Swine, MPS [57]). Notably, ASF resistance showed significant correlations with specific hematological parameters: a positive correlation with platelet distribution width (S_PLDWID) and a negative correlation with red cell distribution width (S_RCDW, p-value < 0.05, **Fig. 6a** and **Supplementary Table S18**). The positive correlation with S_PLDWID suggests that genetic basis influencing platelet size variability are shared with those conferring ASF resistance. As a marker of platelet size variability, S_PLDWID is linked to immune activation and systemic inflammation, processes that may enhance torlerance against ASF infection [58]. Conversely, the negative correlation with S_RCDW, an indicator of erythrocyte size variability, implies a connection between stable red cell morphology and reduced susceptibility to inflammation-induced damage during ASF infection [59]. Lower S_RCDW may reflect diminished oxidative stress and a more regulated inflammatory response, contributing to ASF resistance. These findings identify S_PLDWID and S_RCDW as potential phenotypic markers for breeding strategies aimed at enhancing ASF resistance while maintaining overall health and performance in pig populations. Incorporating these markers into selective breeding programs could facilitate the development of ASF-resistant breeds, thereby support sustainable disease management and improve animal welfare.

Pleiotropic associations of prioritized genes

To explore the pleiotropic effects of ASF resistance-prioritized genes, we analyzed their effects with 299 traits derived from 298 PigBiobank GWAS studies [32] and one trait related to MPS [57]. This analysis identified 1,151 gene-trait pairs across 286 GWAS studies, involving 133 prioritized genes (**Supplementary Table S19**). Notably, 12 traits exhibited significant enrichment, including four health-related traits: lysozyme levels (S_LYSOZ), granulocyte phagocytosis (S_GRANP), blood albumin level (S_ALBU), and low-density lipoprotein (S_LDL) (**Fig. 6b** and **Supplementary Table S20**). These traits are associated with systemic immunity and inflammation, suggesting shared genetic regions between ASF resistance and immune responses.

Cross-trait colocalization analysis revealed shared potential causal variants (PP4 > 0.7) between ASF resistance and lysozyme levels in genes such as *TMEM178A*, *ARHGEF33*, and *THUMPD2*, suggesting their involvement in antiviral defense pathways (**Fig. 6c** and **Supplementary Table. S21**). Conversely, distinct potential causal variants (PP3 > 0.7) were identified for traits like $CD4^+$ leukocyte (*GLT8D2*, *TXNRD1*), platelet counts (*AUST2*), and MPS (*SHISAL1*), indicating genetic linkage, highlighting the locus-specific complexity of the genetic architecture of ASF resistance (**Fig. 6c**).

Beyond immune-related traits, associations were also observed with reproductive traits (e.g., litter weight), production traits (e.g., body length), and meat traits (e.g., meat

quality) (**Fig. 6b** and **c**). Key genes implicated in these associations include *C4H1orf226*, *TPST1*, and *ABCG5*, indicating that ASF resistance signatures may intersect with growth and reproductive traits (**Fig. 6b** and **c**). These findings underscore the importance of further studies to balance disease resistance with production performance in breeding programs.

Construction and application of ASF polygenic resistance score

To evaluate the practical implementation of our findings, we constructed an ASF polygenic resistance score (APRS) based on the ASF-resistance prioritized loci identified in this study and tested its performance within our discovery cohort (the experimental case-control population, n = 474). The APRS was constructed by aggregating favorable alleles at independent lead loci within prioritized gene regions, with each individual's score calculated based on identity-by-state (IBS) similarity to an ideal resistant genotype (**Fig. 6d**).

Our APRS metric demonstrated strong discriminatory power, effectively separating resistant, susceptible, and deceased pigs (**Fig. 6e**). To further infer that this performance was not a byproduct of random chance or cohort-specific bias, we compared APRS to scores derived from randomly selected loci or alleles. The randomized scores failed to differentiate between resistant and deceased individuals (**Fig. 6e** and **Supplementary Tables S21**), confirming that the predictive power of APRS arises from biologically prioritized loci rather than stochastic inflation, and that it meaningfully contributes to the observed resistance phenotype.

To evaluate the reliability of the APRS, we calculated its association with polygenic scores (PGS) for health traits derived from PigBiobank [32] and subsequently applied to our experimental cohort. PGS for 59 traits overlapping with prioritized gene regions were constructed using lassosum [60], with parameter optimization performed through PUMAS [61]. For consistency test, PGS for the same traits were independently generated using PRSice-2 [62] with the clumping and thresholding (C+T) method.

Our analysis revealed a significant positive correlation between APRS and the PGS for platelet distribution width (S_PLDWID), consistent with the results from the genetic correlation analysis (**Fig. 6a**, **f**, **Supplementary Table S18** and **S22**). Individuals in the top decile of APRS exhibited significantly higher PGS_{S_PLDWID} values compared to those in the bottom decile. This is highly relevant as ASFV infection typically leads to thrombocytopenia and disseminated intravascular coagulation (DIC) [63], suggesting that a genetic predisposition for robust coagulation regulation may support resistance. Additionally, APRS positively correlated with PGS for traits critical to viral defense and host homeostasis, such as IgA levels (S_IGA), which serve as a primary mucosal barrier [64], and the IFN-γ/IL-10 ratio (S_IFGIL10), a marker of the pro- *vs.* anti-

inflammatory balance [65] often disrupted by ASFV-induced cytokine storms [63,66]. We also observed correlations with mean corpuscular volume (S_MCV) and eosinophil counts (S_EOS), traits known to be impacted by ASFV-mediated vascular damage and leukopenia [63,66] (**Fig. 6f** and **Supplementary Table S22**). The concordance between APRS and independent immune-related traits indicates that APRS captures a biologically meaningful signal of host resistance rather than cohort-specific noise. Collectively, these findings support its utility as a robust genomic metric for evaluating ASF resistance potential and highlight its promise for application in marker-assisted breeding programs.

Discussion

ASF continues to pose a major threat to global swine production, yet our understanding of host genetic basis conferring resistance remains limited [9,13]. While acute infections with wild-type ASFV typically result in near-complete mortality within two weeks [42,67,68], the high proportion of antibody-positive (P32, P62, P72) but antigen-negative (p22) individuals observed among survivors in this study suggests exposure to low-virulence, gene-deleted, or variant strains rather than classical virulent isolates. Such attenuated strains represent a "hidden" epidemiological challenge: they cause milder symptoms and prolonged infection courses, complicating detection and control. Elucidating host genetic responses under these conditions is therefore critical for developing effective breeding strategies in endemic settings.

In this context, our study delineates the genetic architecture underlying host responses to low-virulence ASFV infection. "Resistance" refers to the host's ability to prevent or control viral infection, including reducing the likelihood of infection or limiting viral replication and spread once infected [69]. In contrast, "tolerance" describes the host's ability to withstand the effects of established infection, with a tolerant individual experiencing attenuated disease impact [69]. In this study, "resistance" is defined as the host's ability to survive infection.

By integrating WGS with functional and transcriptomic annotations, alongside predictive modeling, we established a comprehensive framework to dissect the polygenic nature of ASF resistance in pigs, reflected in the diverse genomic distribution of these genes. This approach identified 1,102 candidate genes associated with ASF resistance, which were further refined to 135 high-priority genes through a rigorous prioritization framework. Enrichment analysis revealed their significant roles in immune-related pathways, aligning with known responses to ASF infection, such as chemokine upregulation [47], MAPK signaling activation [48], and immune cell apoptosis, particularly in myeloid and lymphoid lineages [46].

Chemokines play a critical role in orchestrating immune cell recruitment and activation, contributing to both protective immune responses and pathological outcomes [70]. Our study identified chemokines, including *CXCL2*, *CXCL7*, *CXCL10*, and *CXCL11*, as key contributors to ASF resistance. F_{ST} analysis and AF differences revealed significant genetic differentiation in these genes between ASF-resistant and susceptible populations, highlighting their potential evolutionary importance in shaping resistance traits. Additionally, SMR analysis demonstrated that the expression levels of these chemokines in specific tissues potentially mediate ASF resistance, while DEG profiling further confirmed their dynamic response to ASF infection. Collectively, these findings underscore their dual roles in resistance basis: functioning as putative determinants of genetic predisposition and mediators of infection responses.

Our study also highlights the critical roles of the FcγR (*FCGR2B*, *FCGR3*) and PI3K-AKT (*SOS1* [45], *MVP* [71,72], *PARM1* [73], *TMCC3* [74], *SLC3A1* [75], *BTC* [76]) signaling pathways in ASFV infection. The FcγR family mediates immune regulation through IgG binding, with FCGR3 functioning as an activating receptor and FCGR2B as the sole inhibitory receptor [77], exhibiting opposing immunomodulatory effects. Our analyses revealed a negative correlation between *FCGR3* expression and resistance (supported by SMR, coloc, and downregulation in PAM following infection), while *FCGR2B* expression showed a positive correlation with resistance (supported by SMR, colocalization, BN-GWAS, and upregulation in multiple tissues post-infection). As a regulator of the MAPK, PI3K/JAK cellular signaling pathways and tumorigenesis [44,45], *SOS1* was identified as having a positive effect on ASF resistance. *MVP* mediates immune signaling through JAK/STAT and MAPK pathways [71,72], *SLC3A1* activates AKT signaling, contributing to tumorigenesis [75].

In addition, functional insights into genes supported by at least three independent lines of evidence (drawn from a pool of five methods: reported immune genes, TWAS, SMR, colocalization, and BN-GWAS), suggest diverse biological roles: *KIT* is implicated in tumor cell evasion of TGF-β-mediated growth inhibition [78]. *SDAD1* modulates microglial inflammatory responses via NF-κB [79], and counteracting porcine reproductive and respiratory syndrome virus (PRRSV) infection [80]. *HGSNAT*, encoding a lysosomal membrane acetyltransferase, has been implicated in lysosomal and inflammatory dysregulation when misfolded in mice [81], whereas *ZNF394*, a transcriptional repressor in MAPK pathway, has prognostic value in lung squamous cell carcinoma [82,83]. Other genes, such as *ZNF713* [84,85], *CLIP2* (also known as *CYLN2*) [86], *PHF21B* [87,88], and *SDCCAG8* [89–91], are associated with neurological disorders and reproductive traits, further demonstrating the pleiotropic nature of these loci. These findings suggest that ASFV may exploit these pathways to suppress host immune responses.

ASFV infection triggers a severe systemic crisis, characterized by a cytokine storm and multi-organ hemorrhaging [42,43,92]. Clinical manifestations such as depression (brain), hydropericardium (heart), and diarrhea (intestines) underscore the widespread nature of the disease [42,43,92]. Our time-course transcriptomic data supports this systemic involvement, with the majority (127/132) of prioritized genes exhibiting dynamic expression changes post-infection. While these responses were most robust in lymphoid tissues, viral quantification revealed that non-canonical tissues harbored substantially lower ASFV RNA levels compared to primary targets like the spleen or PAMs. Consequently, the gene expression changes in these non-target tissues likely reflect physiological responses to systemic inflammatory signaling rather than local viral replication. This is supported by the identification of several prioritized genes such as *CXCL10* and *IL15*, which encode secreted proteins that can act as systemic mediators of inter-organ communication [35,93]. The presence of low-level viral RNA in these tissues is likely a secondary effect of viremia. Crucially, the significant heritability enrichment observed in non-immune organs, including the intestines, brain, and heart, provides genetic support for a resistance network that extends beyond canonical immune sites. Furthermore, approximately 27% (36/132) of the prioritized genes (e.g., *ADD1*, *ZNF* family members, and *RUSF1*) were broadly expressed across all examined tissues. Together, these findings suggest that ASF resistance is not solely a localized immune event but involves a multi-organ genetic architecture that maintains systemic integrity. Such a cross-tissue network, potentially involving mucosal immunity in the intestine [94] or neuro-endocrine-immune crosstalk [95], warrants further functional investigation to fully elucidate the basis of host resistance.

At the cellular level, macrophages are well-established as primary targets of ASFV infection and replication [96]. Our study extends this understanding by demonstrating that the heritability of ASF resistance is specifically enriched in macrophage subtypes, particularly Mac_CD163 and Mac_PLBD1. Consistent with Zheng et al.'s cellular landscape [49], we found the Mac_CD163 subpopulation to be pivotal in the host response. Despite their initial sharp decline in prevalence post-infection, this subset demonstrated a remarkable ability to restrict viral replication, characterized by a low proportion of high-viral-load cells [49]. Our analysis adds a critical layer to this observation: the robust antiviral capacity of Mac_CD163 cells is coupled with the sustained activation of our prioritized resistance genes. Specifically, the continuous upregulation of *CXCL2* and *CXCL10* within this subset reinforces their role as key innate immune effectors [97,98]. Furthermore, our basal expression analysis identified genes like *CXCL2*, *CXCL7*, and *BTC* as highly specific to PAMs, which are critical sites for ASFV entry [49]. By linking these specific genetic candidates to established cellular responders, we show that Mac_CD163 cells are not just transcriptomic markers of infection, but are the primary cellular vehicles through which these prioritized resistance factors operate. Collectively, these findings emphasize the interplay between

tissue-specific genetic architecture and systemic immune responses in driving ASFV resistance.

ASFV infection is also associated with hemostasis abnormalities and thrombocytopenia [42,99], findings that are supported by our genetic analysis. Genetic correlation analysis revealed a positive association between ASF resistance and platelet distribution width (S_PLDWID) and a negative association with red blood cell distribution width (S_RCDW). These results suggest that variations in blood cell distribution indices may play a role in shaping the genetic architecture of ASF resistance. Furthermore, the positive correlation between $PGS_{\_S\_PLDWID}$ and APRS underscores the critical role of platelet variability and immune activation in resistance basis. For example, shared genetic signals were observed between ASF resistance and traits such as lysozyme levels (S_LYSOZ), granulocyte phagocytosis (S_GRANP), blood albumin levels (S_ALBU), and low-density lipoprotein (S_LDL). These associations suggest that loci contributing to ASF resistance also influence diverse biological pathways. Elevated lysozyme levels, for instance, may enhance mucosal immunity [100], while improved granulocyte function likely strengthens the innate immune response [101], providing a robust first line of defense against ASFV infection.

Complex traits are inherently polygenic, and the finite number of genomic variants gives rise to widespread genetic overlap and pleiotropy across traits. Many phenotypes are associated with hundreds to thousands of loci, implying the presence of shared causal variants [102]. Consistently, genetic correlation analyses indicate that some variants exert concordant effects on multiple traits [103]. The overlapping signals for ASF resistance and MPS at the *SHISAL1* locus provide insight into multi-trait genetic architecture. Despite both diseases involving respiratory pathology [42,57,104], colocalization analysis strongly supports the PP3 model (PP3 = 0.7153, PP4 = 0.0085), indicating that the associations are driven by distinct potential causal variants in close proximity. This suggests that the *SHISAL1* region represents a multi-trait locus with independent regulatory elements modulating responses to different stressors (bacterial for MPS and viral for ASF), rather than a shared genetic signature. Consistent with this, *SHISAL1* is primarily implicated in Wnt and FGF signaling during development, rather than canonical immune pathways [105]. Together, these findings indicate that the observed overlap reflects local linkage within a complex regulatory landscape rather than true pleiotropy.

Beyond immune-related functions, we observed that ASF resistance-prioritized genes are also associated with reproductive (e.g., litter weight) and production traits (e.g., body length). These genetic associations mirror the clinical manifestations of chronic ASFV infection, where affected pigs often exhibit progressive emaciation, growth stunting, and reproductive disturbances such as abortion [42]. The involvement of key

genes like *TPST1* (TPST1-deficient mice experience decreased body weight and reproductive performance [106]) and *ABCG5* (mediates cholesterol metabolism [107]) suggests that the physiological trade-offs observed during infection may have a partially shared genetic architecture. Our findings provide a genetic framework for understanding how disease resistance signatures may intersect with host fitness and performance. This underscores the necessity of balanced selection in breeding programs to enhance antiviral resilience without compromising essential production and reproductive outcomes.

To translate these insights into actionable breeding tools, we developed the APRS, leveraging identity-by-state similarity to an "ideal" ASF-resistant genotype. The APRS effectively distinguished resistant, susceptible, and deceased individuals, outperforming randomized controls and demonstrating robustness and predictive capability. Its utility is further supported by concordance with the polygenic architecture of traits relevant to ASFV pathogenesis. Polygenic scores (PGS) capture the cumulative effects of multiple variants and are widely used to estimate genetic predisposition, stratify risk, and interrogate trait associations [102,108,109]. The positive correlation of APRS with S_PLDWID and mean corpuscular volume (S_MCV) PGS suggests a genetic link between ASF resistance and the maintenance of hemostatic and vascular integrity, counteracting virus-induced coagulopathy and endothelial damage [63]. Associations with IgA levels (S_IGA) and the IFG-IL10 ratio (S_IFGIL10) PGS highlight the importance of coordinated immune responses, integrating mucosal defense and cytokine balance to limit viral entry and immunopathology [64,65]. Moreover, given ASFV-induced lymphopenia and leukopenia, including reduced eosinophils (S_EOS) [66], the positive correlation between APRS and leukocyte-related PGS indicates that resistant individuals may harbor more resilient hematopoietic and immune systems. Collectively, these findings support pleiotropic effects of prioritized loci across multiple physiological systems, contributing to a multilayered defense against ASFV.

Current ASF control strategies, focused on pre-emptive biosecurity and culling [13], necessarily constrain the collection of large-scale biological samples from natural outbreaks. This has limited the scope of genomic investigations into host resistance. Our study, representing the largest cohort-based analysis under natural infection conditions to date, provides critical insights into the host genetic response to ASFV. While the sample size remains modest compared to standard GWAS and may limit power to detect variants of small effect, we mitigated this constraint through a multidimensional analytical framework that integrated diverse omics datasets. Future studies with expanded cohorts will be crucial to validate these findings and refine the genetic architecture of ASF resistance across diverse populations.

It is important to acknowledge that the genetic signals identified in this study were captured under natural ASFV exposure, where environmental complexities and potential co-infections cannot be fully excluded. Consequently, the candidates identified may represent a combination of ASFV-specific resistance and general disease resistance. While traditional research often focuses on “specific resistance” against a single pathogen [110,111], the quest for “general resistance” remains a major challenge in swine breeding. Our findings, particularly genes involved in the regulation of inflammatory responses and interferon pathways, reflect the host’s capacity to maintain immune homeostasis under multi-pathogen challenges. By integrating these diverse candidate genes into a unified regulatory network, this study provides a systematic map of the host’s genetic defense, spanning from “avoiding infection” and “controlling viral replication” to “tolerating tissue damage and preventing mortality”. This holistic perspective underscores the potential of these loci in breeding programs aimed at improving overall host fitness in complex, real-world production environments.

Functional validation also remains an essential next step. While computational analyses consistently supported the prioritized genes and pathways, approaches such as CRISPR-Cas9 or transgenic models are needed to confirm causal roles. Recent work by Pannhorst et al [112]. demonstrated that the porcine SLA class II complex is indispensable for ASFV infection, with knockout of *SLA-DMA*, *SLA-DMB*, and *RFXANK* leading to profound replication defects across ASFV isolates. In contrast, our study did not identify SLA-related genes. This discrepancy likely reflects the complex architecture, poor annotation, and high polymorphism of the SLA locus, which together complicate the detection of gene-specific signals in genome-wide analyses.

Our findings, derived from a specific population under controlled conditions, may not fully capture the genetic and environmental diversity in global pig populations. Comparative insights from wild suids help contextualize this limitation. African warthogs, which show natural resistance to ASFV, harbor adaptive introgressed immune loci, including MHC and FCGR locus [25], reflecting pathogen-driven selection. Our results independently highlight *FCGR* genes in domestic pigs, suggesting conserved protective functions. Additional overlap was observed for the IL, HERC, and SLC [26,27] gene families, while other wild suid-specific candidates such as *PPEF2* [26], *CFAP69* [26], and *JAKMIP1* [25] appeared in our gene sets to varying degrees. By contrast, genes including *LDHB* [27], *RELA* [113], and members of the TRIM family [27] were not detected in our study population. These comparisons illustrate the value of integrating domestic and wild suid datasets to strengthen biological plausibility.

Lastly, while the APRS demonstrated high discriminatory power in our study, we acknowledge that this evaluation was conducted within the discovery cohort due to the inherent difficulty in obtaining independent validation samples during active ASF

outbreaks. To mitigate potential performance inflation and ensure the scientific validity of the APRS, we employed a multi-layered verification strategy. First, our integrated multi-dimensional prioritization strategy (rather than relying solely on GWAS *p*-values) ensures that the included loci possess intrinsic biological relevance. Second, the failure of randomized loci to replicate this performance further supports the APRS captures specific genetic signals rather than stochastic variation. Third, high APRS scores correspond to a higher statistical likelihood of maintaining immune homeostasis, although absolute survival remains contingent upon the intensity of the viral challenge and environmental stressors. Future studies utilizing independent validation populations or diverse pig breeds will be essential to confirm the cross-population portability of this scoring system and its long-term utility in genomic selection programs. These limitations underscore the importance of continued research to enhance ASF resistance breeding and improve preparedness for ASFV outbreaks.

Our findings lay the foundation for host-targeted ASF control strategies by providing both theoretical insights and practical tools for selective breeding. The integration of ASF-resistance genetic markers into breeding programs could facilitate the development of ASF-resistant pig lines, enhance sustainable swine production and improve outbreak preparedness. Future research should focus on the functional validation of candidate genes through in vitro and in vivo assays and the genomic optimization of resistance loci to refine breeding indices. Collectively, this study advances our understanding of the genetic basis of ASF resistance and offers actionable strategies to mitigate the impact of this devastating disease on the swine industry.

Methods

Experimental population

The experimental population comprised 474 domestic pigs naturally exposed to ASFV across four standardized commercial farms under the same enterprise in Shandong, China. The cohort included a local indigenous breed (LWU, n = 90), a developed commercial breed (LLA, n = 355), and a hybrid population (DLL, n = 29), consisting of 240 boars and 234 sows. All individuals were raised under uniform environmental and management conditions representative of large-scale commercial production.

To ensure accurate classification, infection outcomes were determined through repeated testing: pigs that succumbed to infection were sampled at the point of imminent death, whereas resistant individuals survived for at least two months post-outbreak. Several individuals' status was confirmed via a second round of sampling conducted 2~3 months after the initial test, with 100% concordance.

Blood and ear tissue (0.5 cm^2) were collected for phenotyping and genomic DNA extraction, respectively, by the Institute of Animal Science and Veterinary Medicine, Shandong Academy of Agricultural Sciences (Shandong, China). All samples were heat-inactivated at 70°C for 30 min before transport and analysis. After experimental procedures, all materials were autoclaved and disposed of in accordance with biosafety regulations the China Animal Health and Epidemiology Center.

Phenotyping

To assess the infection status and the nature of the circulating ASFV strain, we employed a diagnostic strategy combining broad-spectrum antibody profiling with targeted antigen DNA detection.

Antibody detection was performed using the ID Screen African Swine Fever Indirect ELISA kit (ID. Vet, France) according to the manufacturer's instructions. This assay targets the ASFV structural proteins P32, P62, and P72, which are highly immunogenic and conserved across a wide range of ASFV lineages, serving as reliable markers for both wild-type and attenuated strain exposure.

Antigen DNA detection was conducted using the RAA fluorescence quantification method (AMPLIFICATION FUTURE, WLE8202KIT). The assay specifically targeted the *KP177R* sequence within the p22 coding region, derived from the ASFV strain China/2018/AnhuiXCGQ (MK128995.1). While p22 is a highly conserved structural protein in wild-type ASFV, the *KP177R* is a recognized hotspot for deletion or mutation in various low-virulence variants and gene-deleted strains. Therefore, the inclusion of this specific target allows for both high-sensitivity detection of viral and the identification of potential gene-deleted variants based on the presence or absence of the p22 genomic fragment. Rapid DNA amplification and fluorescence-based probe detection were employed to ensure maximum sensitivity and specificity in distinguishing ASFV infection patterns.

Based on serological and antigen testing, 474 pigs from an indigenous population were classified into three phenotypic groups (**Table 1** and **Supplementary Table S1**): susceptible-dead pigs (Group A, n = 108), susceptible-resistant pigs (Group B, n = 222), and double-negative pigs (Group C, n = 144).

Concurrent testing of environmental samples from the same farm consistently yielded negative results, indicating that viral exposure was primarily mediated through pig-to-pig transmission rather than environmental contamination. Although differences in viral dose or strain cannot be completely excluded, the contrasting outcomes observed among individuals within a shared environment, together with longitudinal monitoring, support the presence of inherent host resistance differences.

Genotyping

We used whole-genome re-sequencing approach to genotype each individual, achieving an average sequencing depth of 14.3× and a coverage of 0.98 (**Table 1** and **Supplymentary Table S1**). Genomic DNA was isolated from ear tissue of each individual using the CTAB method. Sequencing was conducted on the DNBSEQ-T7 platform (MGI, Shenzhen, China), generating 150-bp paired-end reads with an insert size of 350 bp. For variants calling and quality control, we followed a pipeline as in our previous study [114]. Briefily, the raw FASTQ data underwent quality control, read filtering, and base correction using fastp v0.20.0 with default parameters [115]. High-quality reads were then aligned to the Sscrofa11.1 [116] reference genome using BWA v0.7.17 [117] with the MEM algorithm and parameters optimized for paired-end data. Subsequent processing involved converting SAM files into BAM format and sorting them with samtools v1.10 [118]. Duplicate and unmapped reads were removed with sambamba v0.7.1 [119]. We calculated coverage and depth for each individual with Mosdepth v0.2.9 [120]. Next, we applied GATK v4.1.6 [121] with the HaplotypeCaller function (--read-filter GoodCigarReadFilter) to each sample, generating an intermediate GVCF file, which was then employed in GenotypeGVCFs function for joint genotyping across all samples. The resulting variants were filtered with VCFtools v0.1.13 [122] (--maf 0.05, --max missing 0.9), yielding a total of 23,403,868 variants (including SNPs and indels). Finally, genotypes were phased using BEAGLE v4.1 [123] with default parameters.

To provide a representative genetic background for population-level comparison, 1,730 pigs from the PHARP database [114] were included as controls (Group D, **Table 1** and **Supplementary Table S1**). These individuals were not tested for antibodies or antigens but served as a reference cohort capturing natural allelic variation across diverse commercial and local pig populations, thereby enabling detection of population-specific selection and differentiation signals associated with ASF resistance.

Population structure analysis

Population structure of the experimental pigs was assessed using principal component analysis (PCA), neighbor-joining (NJ) tree construction, and ADMIXTURE analysis. After pruning variants for linkage disequilibrium (LD) with PLINK v1.9 [124] (--indep-pairwise 50 5 0.4), PCA was conducted (--pca). The NJ-tree was built using MEGA v11 [125] and visualized with iTOL v6 [126], and genetic ancestry was inferred via neural-admixture v1.6.3 [127].

Genomic-based identification of ASF-resistance candidate genes

We employed four complementary genomic comparison scenarios to dissect the

multifactorial nature of ASFV resistance (**Fig. 1** and **Table 2**). These contrasts targeted specific defense landscape, from pathogen recognition to adaptive immunity, to minimize phenotypic ambiguity and maximize detection power for potential causal loci under natural infection. To identify potential genetic loci under selection due to ASF infection, we employed two primary methodologies: genome-wide association studies (GWAS) and genetic differentiation analyses combined with allele frequency examination. Initially, GWAS was utilized to identify variants associated with ASF resistance. This method involved scanning the genomes of both resistant and susceptible pigs to identify genetic markers that correlate with resistance to ASF. Recognizing that different resistance or susceptibility to ASFV can shape the genome, we further focused on identifying genomic regions with significant differences in allele frequencies between the resistant and susceptible groups. Specifically, we targeted genomic regions characterized by: i. High differentiation (F_{ST}): genomic regions showing high differentiation between ASF-resistant and ASF-susceptible groups, indicating strong selective pressure. ii. Inverse allele frequency pattern: alleles that display opposite frequency trends between ASF-resistant and ASF-susceptible groups, suggesting divergent selection. iii. Replicable allele frequency pattern: consistent allele frequency trends observed when comparing resistant pigs to control pigs from other breeds that have not experienced ASF outbreaks (e.g., Group D). This replication across different populations strengthens the validity of the identified loci. Variant annotation was performed using R GALLO v1.5 [128].

Genome-wide association analysis

To identify potential genetic loci associated with ASF resistance, we conducted a GWAS comparing the Case group to Control group 1 within the experimental pig population. We used GCTA v1.92.4 software (--make-grm) [129] to calculate the kinship matrix. GWAS was performed uisng GEMMA v0.98.5 software (-lmm 1) [130] with MLMA-LOCO [131] approach. The statistical model applied was:

$$y = \mu + Xb + Zc + \sum_k w_{ik} u_k + e,$$

where y denotes the grouping of an individual (1 for control, 2 for case); μ is the fixed intercept; X is the genotype variable for a SNP (encoded as 0, 1, and 2, corresponding to the three allelic states), b is the fixed effect that is a function of the difference in allele frequencies between the two populations; Z is the design matrix of additional fixed-effect covariates, and c is the vector of effects for sex (2 levels) and farm (4 levels); $\sum_k w_{ik} u_k$ is the fit term for all SNPs on the other chromosomes to control for population differentiation, w_k is the standardized genotype variable for an SNP k, u_k is the corresponding effect size of SNP k, assuming to follow a normal distribution with variance proportional to $p_0(1-p_0)F_{ST}$, where p_0 denotes the allele frequency in the ancestral population; and e is the residual error term, assumed to follow a normal distribution [131]. This modeling framework effectively accounts for population structure and provides greater statistical power than conventional linear regression

approaches [132]. To further mitigate proximal contamination, a LOCO (leave one chromosome out) scheme was implemented, in which SNPs on all other chromosomes were included as random effects when testing the SNP of interest. We retained loci that met the significance threshold of p-value < $\frac{1}{Me}$, where Me represents the total number of loci obtained post-LD pruning. The LD pruning was executed using PLINK v1.9 with parameters --indep-pairwise 50 5 0.4 [124].

Fixation index

We calculated F_{ST} values for each genomic segment to measure genetic differentiation using VCFtools v0.1.13 with parameters --fst-window-size 100000 --fst-window-step 10000. To assess differences in allele frequencies at each locus, we also conducted a chi-square test for allele frequencies at each locus using PLINK v1.9 [124] (--assoc). We considered those that met the following four conditions to be candidate loci: i. the top 1% of highly differentiated segments were retained based on the F_{ST} values; ii. Selected loci with a chi-square test p-value < $\frac{0.05}{N}$, were N is the total number of loci; iii. Ensured that loci met both criteria (i) and (ii) in both comparisons: Case *vs.* Control 1 and Case *vs.* Control 2; iv. Consistent direction of AF changes across comparisons. Namely, $AF_{\mathrm{Case}} > AF_{\mathrm{Control1}}$ and $AF_{\mathrm{Case}} > AF_{\mathrm{Control2}}$ or $AF_{\mathrm{Case}} < AF_{\mathrm{Control1}}$ and $AF_{\mathrm{Case}} < AF_{\mathrm{Control2}}$.

Gene prioritization

Transcriptome-wide association analysis

To prioritize candidate genes by aggregating the cumulative effects of multiple cis-variants on predicted expression levels, we conducted TWAS analysis. Using the FarmGTEx TWAS-server [133], we evaluated associations between candidate genes and ASF resistance across 34 tissues from PigGTEx [31] (**Supplementary Table S6**). Significant associations were identified using a false discovery rate (FDR) < 0.05.

Summary-data-based mendelian randomization and colocalization with gene expression

To identify potential regulatory links by testing the mediation effect of top eQTL signals on ASF resistance, we employed the SMR framework. We used SMR v1.3.1 [134] (--smr-multi) to assess the effect of expression quantitative trait loci (eQTL) (exposure) on ASF resistance (outcome). Instrumental variables were selected based on p-value < 0.005 and LD $r^2 < 0.3$ in the cis-region. Significance was defined by $p_{\mathrm{SMR}} < 0.05$, $p_{\mathrm{SMR.MULTI}} < 0.05$, and $p_{\mathrm{HEIDI}} > 0.05$ to exclude heterogeneity.

To further refine prioritized genes by distinguishing shared genetic control from mere genomic linkage, we performed colocalization analysis. Using the R coloc v5.2.3 [135], we tested variant loci within ±1 Mb of significant eQTLs. Signal pairs with posterior probability of hypothesis 4 (PP4) > 0.75 were considered co-localized (shared variants), while those with posterior probability of hypothesis 3 (PP3) > 0.75 indicated independent variants driving the two signals.

Both SMR and colocalization utilized pooled cis-eQTL data from PigGTEx (34 tissues) [31] (**Supplementary Table S6**).

Bayesian Network genome-wide association study

We employed a Bayesian Network Genome-Wide Association Study (BN-GWAS) to evaluate the network of potential causal relationships between candidate gene expression and ASF resistance traits [41]. BN-GWAS constructs directed gene-gene-phenotype potential causal networks using imputed expression profiles from GWAS and raw expression data from a reference dataset. For this analysis, we used raw expression data from PigGTEx [31] for five tissues with sample sizes exceeding 300: muscle, blood, brain, embryo, and liver (**Supplementary Table S6**).

Omnibus gene prioritization score

We integrated the above inference methods to construct a comprehensive gene prioritization score, which ranks candidate genes based on their potential importance for ASF resistance (**Table 4**). The following criteria were applied:
i. A base score of 1 was assigned to each gene identified by GWAS and F_{ST} methods, with an additional score of 1 for each repetition in a comparison subgroup.
ii. An additional 0.5 score were awarded if the gene was previously reported as an immune gene [49,136] (**Supplementary Table S5**).
iii. A score of 1.2 was given if the gene was supported in TWAS or BN-GWAS, with an additional 1.2 score for each repetition in different tissues.
iv. A count of 1 was assigned if the gene was supported by SMR or coloc, with an additional count for each tissue repetition. Due to the multi-tissue analysis, SMR and coloc inference were log-transformed and weighted by 0.8 to prevent over-representation.
v. Independent inference:
- An additional 3 scores were assigned if a gene was supported in all 5 methods.
- An additional 2 scores were given if the gene was supported in 3 or 4 methods.
- An additional 1 score was awarded if the gene was supported in 2 methods.

Pathway enrichment analysis

Pathway enrichment analyses were performed on the prioritized gene sets using Gene Ontology (GO), Kyoto Encyclopedia of Genes and Genomes (KEGG), Reactome, and QTL databases. GO and KEGG enrichment were carried out using R clusterProfiler v4.6.2 [137], Reactome enrichment was done with R ReactomePA v1.42.0 [138], and QTL annotation and enrichment were conducted using R GALLO v1.5 [128]. All statistical analyses were corrected for multiple comparisons using the Benjamini-Hochberg (BH) method, and results with *p.adj* < 0.05 were considered significant.

Tissue- and cell-type heritability enrichment analysis

We employed the LDSC-SEG [139] model to assess genetic heritability enrichment across 34 tissues from the PigGTEx dataset [31] (**Supplementary Table S6**). Recognizing that porcine alveolar macrophages (PAMs) are primary target cells for ASF infection, we further extended the analysis to eight cell types identified in PAMs. These cell types were characterized using single-cell transcriptomic data from 118,316 cells derived from 13 in vitro samples [49].

The LD reference panel was constructed using PGRP v1 [31], comprising genomic data from 1,602 individuals representing over 100 breeds. Tissue- and cell-type-specific gene regions were defined based on the top 1,000 most highly expressed genes in each tissue or cell type, with an additional 100 kb window to capture surrounding regulatory elements [32,139].

Transcriptome annotation

Multi-tissue bulk transcriptomic analyses

To annotate the prioritized genes within a broader biological context, we analyzed two publicly available bulk RNA-sequencing datasets retrieved from the NCBI SRA [140] (PRJNA960638) and CNCB-NGDC GSA [141,142] (PRJCA003613 [143]) databases (**Supplementary Table S1**). These datasets encompass transcriptomic profiles from 12 pig tissues and cell types at multiple time points after ASFV infection versus uninfected controls, including PAMs, peripheral blood mononuclear cells (PBMC), heart, kidney, liver, lung, inguinal, mandibular, mesenteric, muscle, spleen, and tonsils**.**

i. Quality control and read mapping

Raw RNA sequencing reads underwent quality control using fastp v0.20.0 [115] with default parameters. High-quality reads were then mapped to the Sscrofa11.1 reference genome using HISAT2 v2.1.0 [144]. Read counts were quantified using featureCounts v2.0.3 (-t exon -g gene_id) [145], and gene expression was quantified at the

transcriptional level in transcripts per million (TPM). Genes were considered expressed if TPM > 0.1 in at least 20% of samples. After applying this threshold, 23,331 genes remained available for downstream analyses, including 132 prioritized genes, which were retained for further investigation.

ii. Tissue-specific expression analysis

We assessed the tissue specificity of the prioritized genes using tissue-specific gene expression (TAU) and expression specificity scores (ESS) indices:
TAU Index quantifies the specificity of gene expression across tissues, ranging from 0 to 1, where values closer to 1 indicate higher tissue specificity [146]. It was calculated as:

$$TAU = \frac{\sum_{i=1}^{n}(1-\frac{x_i}{x_{max}})}{n-1},$$

Where n is the number of tissues, x_i represents the expression level of a gene in a given tissue, and x_{max} denotes its highest expression value across all tissues.

ESS Index measures the degree to which a gene is preferentially expressed in a specific tissue, also ranging from 0 to 1, with values closer to 1 indicating stronger expression bias [147]. It was computed as:

$$ESS = \frac{med\ (log_2 TPM)}{\sum med\ (log_2 TPM)}.$$

iii. Differentially expressed genes (DEG) identification

To identify differentially expressed prioritized genes across different infection time points, we performed differential expression analysis using DESeq2 v1.34.0 [148,149] for each tissue separately, incorporating time as a factor in the experimental design. Genes were considered significantly differentially expressed if they met the following criteria:
FDR < 0.05 and the absolute log2FoldChange ($|log_2FC|$) > 1.

iv. Time-series analysis of dynamic gene expression

Given that transcriptomic data were collected at multiple post-infection time points, we conducted time-series analysis using R maSigPro v1.66.0 [150]. To account for temporal trends, we applied polynomial regression models with the "backward" variable selection method, setting different degrees for different tissues:

- PAM: degree = 4
- PBMC: degree = 5
- Other tissues: degree = 2

The selection of polynomial degrees was based on the complexity of the time-course

experimental design. Specifically, PAM and PBMC datasets had a more intricate temporal structure compared to other tissues, necessitating higher-degree polynomials to better capture gene expression dynamics. Preliminary testing indicated that the chosen degrees provided an optimal balance between model fit and the number of significantly dynamic genes identified. Genes were classified as dynamically responsive genes if they satisfied the following thresholds: FDR < 0.05 and Coefficient of determination (R^2) > 0.5.

Viral transcriptomic analysis

Unmapped reads from the host alignment were aligned to ASFV reference sequences (MK333180.1.fa, MT748042.2.fa) using Bowtie2 v2.3.5.1 [151]. Viral read counts were generated with featureCounts v2.0.3 [145] (-t CDS -g gene_name) and normalized to reads per million (RPM) to quantify viral load. Spearman's rank correlation coefficient was used to assess the relationship between viral RPM and host prioritized gene expression (TPM) across tissues.

Single-cell transcriptomic analyses of PAM

i. Data processing

We retrieved ASFV-infected PAM single-cell RNA sequencing data from the NCBI SRA (PRJNA706032 [49]) (**Supplementary Table S1**). The raw sequencing data were processed using Cell Ranger v7.0.1 [152] with the Sscrofa11.1 reference genome [116]. To ensure high data quality, we applied stringent filtering criteria:

- Mitochondrial RNA content < 10% of total RNA
- Number of detected genes per cell: between 500 and 7,500 **[49]**.

After applying this threshold, 118,316 cells and 14,871 genes remained for downstream analyses, including 127 prioritized genes selected for further investigation. Subsequent analyses were performed using R Seurat v5.0.1 [153]. Cell clustering was performed using the FindClusters function with a resolution parameter of 0.2, ensuring a biologically relevant granularity of clusters. Based on marker genes from Zheng et al. [49], cells were classified into five major populations: macrophages (Mac), mast cells (Mast), T cells (T), proliferating cells (Pro), and epithelial cells (Epi). The macrophage population was further subclustered into four subtypes: Mac_HLA_DRA, Mac_CD163, Mac_CREG1, and Mac_PLBD1 (**Fig. 3d**).

ii. Prioritized gene expression scoring

To quantify the expression levels of prioritized genes across different cell types, we employed the AddModuleScore function in Seurat v5.0.1 [153], which calculates a module score for a predefined gene set within individual cells. To assess infection-

induced changes, module scores of infected cells (categorized by cell type and infection time) were compared to control cells using a two-sided Welch's t-test [154].

iii. Differential gene expression analysis

Differential expression analysis of prioritized genes was conducted using the FindMarkers function in Seurat v5.0.1 [153]. Comparisons were conducted between infected and uninfected groups across different cell types and infection time points. Significantly differentially expressed genes were defined as those meeting the following criteria: FDR < 0.05 and $|\log_2 FC| > 1$.

Genetic correlation analysis

We utilized the LD score regression model in LDSC v1.0.1 [103] to estimate the genetic correlation between ASF resistance and other pig traits. A total of 122 GWAS summary datasets were analyzed, encompassing 121 datasets from PigBiobank [32] (initially 268, with 147 excluded due to insufficient sample size or low heritability) and one dataset from a published GWAS on MPS [57].

The LD reference panel was constructed using PGRP v1 [31] , ensuring a comprehensive representation of SNP linkage patterns. Each GWAS summary dataset was standardized by aligning alleles (A1, A2) and effect sizes. Quality control filters were applied, retaining SNP loci with $|Z| < 5$ to exclude potential outliers and enhance result robustness.

Pleiotropy annotation

To evaluate the pleiotropic effects of ASF resistance-associated genes, we analyzed the overlap between the prioritized gene set for ASF resistance and significant genes identified in other phenotypes. This analysis incorporated data from 298 meta-GWAS studies in the PigBiobank [32] and a published GWAS on MPS [57].

The significance of gene overlaps with phenotypes was determined using hypergeometric tests. For overlapping gene-phenotype pairs (from 268 traits), we applied co-localization analysis using the R coloc v5.2.3 [135]. Genetic variants within a 50-kb window upstream and downstream of lead SNPs were examined. Signal pairs with a posterior probability of hypothesis 4 (PP4 > 0.7) were considered co-localized, indicating potential shared causal variants. Conversely, pairs with posterior probability of hypothesis 3 (PP3 > 0.7) were interpreted as significantly associated but driven by distinct potential causal variants.

Construction of ASF polygenic resistance score (APRS) using prioritized loci

Definition of APRS

Identity by state (IBS) refers to the condition where two individuals share the same allele. In this context, IBS states are categorized as 0, 1, or 2 based on the number of shared alleles. Here, we define the IBS-based distance between an experimental and an "ideal" individual as the ASF polygenic resistance score (APRS) (**Fig. 6d**). The APRS evaluates the genetic similarity between an individual and an ideal ASF-resistant genotype, enabling prediction of ASF resistance. The IBS distance is calculated as follows:

$$IBS = \frac{(Number\ of\ IBS2) + (0.5 * Number\ of\ IBS1)}{Total\ Number\ of\ SNPs},$$

where *IBS2* represents loci where both alleles match the ideal genotype, and *IBS1* represents loci where only one allele matches.

Design of ideal genotype

A total of 40 independent loci were selected to define the ideal ASF-resistant genotype. Specifically, within 135 high-confidence gene regions, the most significantly associated (*p*-values) SNPs were first identified as candidate loci. LD clumping (PLINK v1.9 [124], --indep-pairwise 50 5 0.1) was then applied to retain independent, non-redundant lead SNPs for inclusion in the final APRS model. The assignment of ideal alleles was based on statistical evidence from GWAS and F_{ST} analyses (**Fig. 6d**). The specific criteria for ideal allele selection were as follows:

i. GWAS-derived loci:

- If the Z-score of the SNP was positive (Z > 0), the effect allele (minor allele) was designated as the ideal allele.
- If the Z-score was negative (Z < 0), the reference allele (major allele) was assigned as the ideal allele.

The Z-score for each SNP was calculated as:

$$Z = \frac{\beta}{SE},$$

where β represents the effect size, and *SE* is the standard error.

ii. F_{ST}-derived loci:

- The Z-score was defined as the sum of the standardized statistics from two independent comparisons (case/control1 and case/control2).
- The sign of the Z-score (positive or negative) was determined based on the Odds Ratio (OR), ensuring alignment with the direction of selection pressure.

Once the ideal alleles were assigned, the IBS distance between each individual and the ideal genotype was computed using PLINK v1.9 [124] (--cluster-matrix).

Statistical Robustness and Indirect Validation of APRS

Given the logistical and biosecurity constraints of collecting large-scale independent cohorts during active ASF outbreaks, we implemented a multi-layered framework to evaluate the robustness and biological consistency of the APRS within the study population.
i. APRS construction incorporated multi-dimensional evidence, relying on loci prioritized through an integrative framework rather than solely on GWAS significance, thereby ensuring independent functional support for each included locus.
ii. Internal robustness was assessed via permutation testing.
iii. Biological consistency was examined by correlating APRS with polygenic scores (PGS) for 59 independent health-related traits relevant to ASF resistance, including hematological and immune parameters.

Permutation by randomized comparison

To assess the specificity and robustness of the APRS, we performed a randomized permutation within our discovery cohort (the experimental case-control population, n = 474). This involved:
i. Selecting an equal number of random loci using PLINK v1.9 [124] (--thin-count parameter).
ii. Generating "alternative ideal individuals" by disrupting the ideal alleles at the defined loci.
The resistance scores obtained from these random loci served as benchmarks against the APRS values derived from the prioritized ASF-resistance associated loci. This approach ensured that the predictive power of APRS was attributable to ASF-specific genetic variation rather than random genomic background noise.

Polygenic score analysis

Based on the pleiotropy annotation results, 59 health traits from PigBiobank [32] were identified as overlapping with prioritized gene regions.

PGS construction and parameter optimization

PGS for these traits were generated using lassosum [60], with PGRP v1 [31] as the LD reference panel. To ensure model robustness and prevent overfitting, PUMAS [61] was used to subsample PigBiobank GWAS summary statistics, implementing a training-testing data split, cross-validation, and repeated learning. This approach optimized the shrinkage coefficient (s) and lambda (λ) parameters to achieve maximum R^2.

To substantiate consistency, PGS were also constructed using PRSice-2 [62] with the "clumping and thresholding" (C+T) method (--bar-levels 5e-8, 1e-5, 0.001, 0.05, 0.1, 0.5), allowing direct comparison between the two approaches.

Association analysis in the experimental cohort

These externally-trained PGS models were subsequently applied to the pigs in our experimental case-control population. The association between the externally-derived PGS and our internally-constructed APRS was evaluated using the Spearman rank correlation coefficient. To enhance interpretability and highlight extreme genetic profiles, the experimental population was divided into deciles based on APRS values. Linear regression analysis was conducted using APRS as the response variable and standardized PGS as the predictor variable, focusing on statistical significance in the highest decile compared to the fifth and the lowest (bottom) deciles.

Data availability

The whole-genome sequencing data for the 474 experimental pigs generated in this study are available in the NCBI SRA under accession PRJNA1290525. The processed SNP genotype data (VCF format) central to our analysis have been submitted to the GVM under accession PRJCA057587. Additionally, genomic data for other pig breeds are available at PHARP [114]. The public transcriptome datasets utilized in this study can be accessed through the Sequence Read Archive (SRA) and the Gene Expression Omnibus (GEO), as specified in the Methods section. EQTL data can be accessed via PigGTEx [31], and GWAS summary statistics for pleiotropic analyses are available upon request from PigBiobank [32].

Acknowledgements

We thank the pig farms for providing the samples. This work was supported by the Agricultural Improved Varieties Project in Shandong Province of China (2021LZGC001), National Natural Science Foundation of China (31941007).

Author contributions

Conceptualization, Z. Wang and Y. C. Pan; methodology, Z. Wang; validation, X. W. Ye; formal analysis, X. W. Ye and Q. Q. Xie; resources, W.B. Sun; data curation, C. Y. Cao and Shuang Liu; writing—original draft preparation, X. W. Ye and Z. Wang; writing—review and editing, X. W. Ye and Z. Wang; visualization, X. W. Ye;

supervision, Y. C. Pan, Q. S. Wang, Z. Zhang and Z. Wang; project administration, Y. C. Pan and Z. Wang; funding acquisition, Y. C. Pan and Z. Wang. All authors have read and agreed to the published version of the manuscript.

Declaration of interest

The authors declare that they have no competing interests.

References

1. Dixon LK, Sun H, Roberts H. African swine fever. *Antiviral Research*. 2019; doi: 10.1016/j.antiviral.2019.02.018.
2. WOAH: Situation reports for African swine fever (ASF). World Organisation for Animal Health. https://www.woah.org/en/disease/african-swine-fever/ (2025). Accessed 2025 June 29.
3. Sánchez-Cordón PJ, Montoya M, Reis AL, Dixon LK. African swine fever: A re-emerging viral disease threatening the global pig industry. *The Veterinary Journal*. 2018; doi: 10.1016/j.tvjl.2017.12.025.
4. FAO: ASF in Asia: One year on, close to 5 million pigs lost. Professional Pig Community. https://www.pig333.com/latest_swine_news/asf-in-asia-one-year-on-close-to-5-million-pigs-lost_15239/ (2019). Accessed 2025 June 25.
5. FAS-USDA: Russia: economic impact of African Swine Fever. Professional Pig Community. https://www.pig333.com/latest_swine_news/russia-economic-impact-of-african-swine-fever_5019/ (2011). Accessed 2025 June 25.
6. IFIP: ASF draws dangerously close to France: Possible effects on pork trade. Professional Pig Community. https://www.pig333.com/articles/asf-draws-dangerously-close-to-france-impact-on-pork-trade_18542/ (2022). Accessed 2025 June 25.
7. Department of Agriculture, Fisheries, and Forestry, Australia: ASF could cost Australia $2.5 billion. Professional Pig Community. https://www.pig333.com/latest_swine_news/potential-economic-consequences-of-african-swine-fever-in-australia_19171/ (2023). Accessed 2025 June 25.
8. Carriquiry M, Elobeid A, Swenson D, Hayes D: If ASF reached the US it would cost $50 billion. Professional Pig Community. https://www.pig333.com/latest_swine_news/if-asf-reached-the-us-it-would-cost-$50-billion_16043/ (2020). Accessed 2025 June 25.
9. Wang T, Sun Y, Qiu H-J. African swine fever: an unprecedented disaster and challenge to China. *Infectious Diseases of Poverty*. 2018; doi: 10.1186/s40249-018-0495-3.
10. Zhou X, Li N, Luo Y, Liu Y, Miao F, Chen T, et al.. Emergence of African Swine Fever in China, 2018. *Transboundary and Emerging Diseases*. 2018; doi:

10.1111/tbed.12989.

11. Liu J, Liu B, Shan B, Wei S, An T, Shen G, et al.. Prevalence of African Swine Fever in China, 2018-2019. *Journal of Medical Virology*. 2020; doi: 10.1002/jmv.25638.

12. You S, Liu T, Zhang M, Zhao X, Dong Y, Wu B, et al.. African swine fever outbreaks in China led to gross domestic product and economic losses. *Nat Food*. 2021; doi: 10.1038/s43016-021-00362-1.

13. Dixon LK, Stahl K, Jori F, Vial L, Pfeiffer DU. African Swine Fever Epidemiology and Control. *Annual Review of Animal Biosciences*. 2020; doi: 10.1146/annurev-animal-021419-083741.

14. Galindo I, Alonso C. African Swine Fever Virus: A Review. *Viruses*. 2017; doi: 10.3390/v9050103.

15. Wu K, Liu J, Wang L, Fan S, Li Z, Li Y, et al.. Current State of Global African Swine Fever Vaccine Development under the Prevalence and Transmission of ASF in China. *Vaccines (Basel)*. 2020; doi: 10.3390/vaccines8030531.

16. Mazur-Panasiuk N, Żmudzki J, Woźniakowski G. African Swine Fever Virus – Persistence in Different Environmental Conditions and the Possibility of its Indirect Transmission. *J Vet Res*. 2019; doi: 10.2478/jvetres-2019-0058.

17. Wang T, Luo R, Sun Y, Qiu H-J. Current efforts towards safe and effective live attenuated vaccines against African swine fever: challenges and prospects. *Infectious Diseases of Poverty*. 2021; doi: 10.1186/s40249-021-00920-6.

18. Rock DL. Challenges for African swine fever vaccine development—"… perhaps the end of the beginning." *Veterinary Microbiology*. 2017; doi: 10.1016/j.vetmic.2016.10.003.

19. Wang Y, Kang W, Yang W, Zhang J, Li D, Zheng H. Structure of African Swine Fever Virus and Associated Molecular Mechanisms Underlying Infection and Immunosuppression: A Review. *Front Immunol*. 2021; doi: 10.3389/fimmu.2021.715582.

20. Netherton CL, Connell S, Benfield CTO, Dixon LK. The Genetics of Life and Death: Virus-Host Interactions Underpinning Resistance to African Swine Fever, a Viral Hemorrhagic Disease. *Front Genet*. 2019; doi: 10.3389/fgene.2019.00402.

21. Leitão A, Cartaxeiro C, Coelho R, Cruz B, Parkhouse RME, Portugal F, et al.. The non-haemadsorbing African swine fever virus isolate ASFV/NH/P68 provides a model for defining the protective anti-virus immune response. *The Journal of general virology*. 2001; doi: 10.1099/0022-1317-82-3-513.

22. Muñoz-Moreno R, Cuesta-Geijo MÁ, Martínez-Romero C, Barrado-Gil L, Galindo I, García-Sastre A, et al.. Antiviral Role of IFITM Proteins in African Swine Fever Virus Infection. *PLOS ONE*. 2016; doi: 10.1371/journal.pone.0154366.

23. Netherton CL, Simpson J, Haller O, Wileman TE, Takamatsu H-H, Monaghan P, et al.. Inhibition of a Large Double-Stranded DNA Virus by MxA Protein. *Journal of Virology*. 2009; doi: 10.1128/jvi.00781-08.

24. Anderson EC, Hutchings GH, Mukarati N, Wilkinson PJ. African swine fever virus

infection of the bushpig (*Potamochoerus porcus*) and its significance in the epidemiology of the disease. *Veterinary Microbiology*. 1998; doi: 10.1016/S0378-1135(98)00187-4.

25. Garcia-Erill G, Jørgensen CHF, Muwanika VB, Wang X, Rasmussen MS, de Jong YA, et al.. Warthog Genomes Resolve an Evolutionary Conundrum and Reveal Introgression of Disease Resistance Genes. *Mol Biol Evol*. 2022; doi: 10.1093/molbev/msac134.

26. Xie H-B, Yan C, Adeola AC, Wang K, Huang C-P, Xu M-M, et al.. African Suid Genomes Provide Insights into the Local Adaptation to Diverse African Environments. *Mol Biol Evol*. 2022; doi: 10.1093/molbev/msac256.

27. Feng W, Zhou L, Zhao P, Du H, Diao C, Zhang Y, et al.. Comparative Genomic Analysis of Warthog and Sus Scrofa Identifies Adaptive Genes Associated with African Swine Fever. *Biology*. 2023; doi: 10.3390/biology12071001.

28. Ogweng P, Bowden CF, Smyser TJ, Muwanika VB, Piaggio AJ, Masembe C. Ancestry and genome-wide association study of domestic pigs that survive African swine fever in Uganda. *Trop Anim Health Prod*. 2024; doi: 10.1007/s11250-024-04195-5.

29. Qi F, Chen X, Wang J, Niu X, Li S, Huang S, et al.. Genome-wide characterization of structure variations in the Xiang pig for genetic resistance to African swine fever. *Virulence*. 2024; doi: 10.1080/21505594.2024.2382762.

30. Fang L, Teng J, Lin Q, Bai Z, Liu S, Guan D, et al.. The Farm Animal Genotype–Tissue Expression (FarmGTEx) Project. *Nat Genet*. 2025; doi: 10.1038/s41588-025-02121-5.

31. Teng J, Gao Y, Yin H, Bai Z, Liu S, Zeng H, et al.. A compendium of genetic regulatory effects across pig tissues. *Nat Genet*. 2024; doi: 10.1038/s41588-023-01585-7.

32. Zeng H, Zhang W, Lin Q, Gao Y, Teng J, Xu Z, et al.. PigBiobank: a valuable resource for understanding genetic and biological mechanisms of diverse complex traits in pigs. *Nucleic Acids Research*. 2024; doi: 10.1093/nar/gkad1080.

33. Nagarsheth N, Wicha MS, Zou W. Chemokines in the cancer microenvironment and their relevance in cancer immunotherapy. *Nat Rev Immunol*. 2017; doi: 10.1038/nri.2017.49.

34. Dong C, Davis RJ, Flavell RA. MAP Kinases in the Immune Response. *Annual Review of Immunology*. 2002; doi: 10.1146/annurev.immunol.20.091301.131133.

35. Waldmann TA. The biology of interleukin-2 and interleukin-15: implications for cancer therapy and vaccine design. *Nat Rev Immunol*. 2006; doi: 10.1038/nri1901.

36. Kubes P, Jenne C. Immune Responses in the Liver. *Annual Review of Immunology*. Annual Reviews; 2018; doi: 10.1146/annurev-immunol-051116-052415.

37. Kusters YHAM, Barrett EJ. Muscle microvasculature's structural and functional specializations facilitate muscle metabolism. *American Journal of Physiology-Endocrinology and Metabolism*. American Physiological Society; 2016; doi:

10.1152/ajpendo.00443.2015.

38. Andrés G. African Swine Fever Virus Gets Undressed: New Insights on the Entry Pathway. *Journal of Virology*. 2017; doi: 10.1128/jvi.01906-16.

39. Zwick M, Ulas T, Cho Y-L, Ried C, Grosse L, Simon C, et al.. Expression of the Phosphatase Ppef2 Controls Survival and Function of CD8+ Dendritic Cells. *Front Immunol*. 2019; doi: 10.3389/fimmu.2019.00222.

40. Kutuzov MA, Bennett N, Andreeva AV. Protein phosphatase with EF-hand domains 2 (PPEF2) is a potent negative regulator of apoptosis signal regulating kinase-1 (ASK1). *The International Journal of Biochemistry & Cell Biology*. 2010; doi: 10.1016/j.biocel.2010.07.014.

41. Yin L, Feng Y, Shi Y, Lau A, Qiu J, Sham P-C, et al.. Estimation of causal effects of genes on complex traits using a Bayesian-network-based framework applied to GWAS data. *Nat Mach Intell*. 2024; doi: 10.1038/s42256-024-00906-7.

42. Salguero FJ. Comparative Pathology and Pathogenesis of African Swine Fever Infection in Swine. *Front Vet Sci*. 2020; doi: 10.3389/fvets.2020.00282.

43. Zhu Z, Mao R, Liu B, Liu H, Shi Z, Zhang K, et al.. Single-cell profiling of African swine fever virus disease in the pig spleen reveals viral and host dynamics. *Proceedings of the National Academy of Sciences of the United States of America*. 2024; doi: 10.1073/pnas.2312150121.

44. Liu K, Jiang T, Ouyang Y, Shi Y, Zang Y, Li N, et al.. Nuclear EGFR impairs ASPP2-p53 complex-induced apoptosis by inducing SOS1 expression in hepatocellular carcinoma. *Oncotarget*. 2015; doi: 10.18632/Oncotarget.3757.

45. Akan DT, Howes JE, Sai J, Arnold AL, Beesetty Y, Phan J, et al.. Small Molecule SOS1 Agonists Modulate MAPK and PI3K Signaling via Independent Cellular Responses. *ACS Chemical Biology*. 2019; doi: 10.1021/acschembio.8b00869.

46. Tian Y, Wang D, He S, Cao Z, Li W, Jiang F, et al.. Immune cell early activation, apoptotic kinetic, and T-cell functional impairment in domestic pigs after ASFV CADC_HN09 strain infection. *Front Microbiol*. 2024; doi: 10.3389/fmicb.2024.1328177.

47. Machuka EM, Juma J, Muigai AWT, Amimo JO, Pelle R, Abworo EO. Transcriptome profile of spleen tissues from locally-adapted Kenyan pigs (Sus scrofa) experimentally infected with three varying doses of a highly virulent African swine fever virus genotype IX isolate: Ken12/busia.1 (ken-1033). *BMC Genomics*. 2022; doi: 10.1186/s12864-022-08754-8.

48. Gao Q, Xu Y, Feng Y, Zheng X, Gong T, Kuang Q, et al.. Deoxycholic acid inhibits ASFV replication by inhibiting MAPK signaling pathway. *International Journal of Biological Macromolecules*. 2024; doi: 10.1016/j.ijbiomac.2024.130939.

49. Zheng Y, Li S, Li S-H, Yu S, Wang Q, Zhang K, et al.. Transcriptome profiling in swine macrophages infected with African swine fever virus at single-cell resolution. *Proc Natl Acad Sci U S A*. 2022; doi: 10.1073/pnas.2201288119.

50. Barreto de Albuquerque J, Altenburger LM, Abe J, von Werdt D, Wissmann S,

Martínez Magdaleno J, et al.. Microbial uptake in oral mucosa–draining lymph nodes leads to rapid release of cytotoxic CD8+ T cells lacking a gut-homing phenotype. *Science Immunology*. 2022; doi: 10.1126/sciimmunol.abf1861.

51. Jiang M, Zou J, Jin Y, Jiang C, Tu S, Chen T, et al.. Adducin-1 Facilitates Influenza Virus Endosomal Trafficking and Uncoating by Regulating Branched Actin Dynamics and Myosin IIB Activity. *Advanced Science*. 2025; doi: 10.1002/advs.202417318.

52. Zou J, Yue F, Jiang X, Li W, Yi J, Liu L. Mitochondrion-associated protein LRPPRC suppresses the initiation of basal levels of autophagy via enhancing Bcl-2 stability. *Biochem J*. 2013; doi: 10.1042/BJ20130306.

53. Tokunaga R, Zhang W, Naseem M, Puccini A, Berger MD, Soni S, et al.. CXCL9, CXCL10, CXCL11/CXCR3 axis for immune activation - a target for novel cancer therapy. *Cancer Treat Rev*. 2018; doi: 10.1016/j.ctrv.2017.11.007.

54. Watanabe C, Shu GL, Giltiay NV, Clark EA. Regulation of B-lineage cells by caspase 6. *Immunology & Cell Biology*. 2018; doi: 10.1111/imcb.12172.

55. Hulea L, Nepveu A. CUX1 transcription factors: From biochemical activities and cell-based assays to mouse models and human diseases. *Gene*. 2012; doi: 10.1016/j.gene.2012.01.039.

56. Kubota T, Kubota N, Kadowaki T. Imbalanced Insulin Actions in Obesity and Type 2 Diabetes: Key Mouse Models of Insulin Signaling Pathway. *Cell Metabolism*. Elsevier; 2017; doi: 10.1016/j.cmet.2017.03.004.

57. Xu Z, Sun H, Zhang Z, Zhang C-Y, Zhao Q, Xiao Q, et al.. Selection signature reveals genes associated with susceptibility loci affecting respiratory disease due to pleiotropic and hitchhiking effect in Chinese indigenous pigs. *Asian-Australas J Anim Sci*. 2020; doi: 10.5713/ajas.18.0658.

58. Izzi B, Gialluisi A, Gianfagna F, Orlandi S, De Curtis A, Magnacca S, et al.. Platelet Distribution Width Is Associated with P-Selectin Dependent Platelet Function: Results from the Moli-Family Cohort Study. *Cells*. 2021; doi: 10.3390/cells10102737.

59. Salvagno GL, Sanchis-Gomar ,Fabian, Picanza ,Alessandra, and Lippi G. Red blood cell distribution width: A simple parameter with multiple clinical applications. *Critical Reviews in Clinical Laboratory Sciences*. 2015; doi: 10.3109/10408363.2014.992064.

60. Mak TSH, Porsch RM, Choi SW, Zhou X, Sham PC. Polygenic scores via penalized regression on summary statistics. *Genetic Epidemiology*. 2017; doi: 10.1002/gepi.22050.

61. Zhao Z, Yi Y, Song J, Wu Y, Zhong X, Lin Y, et al.. PUMAS: fine-tuning polygenic risk scores with GWAS summary statistics. *Genome Biology*. 2021; doi: 10.1186/s13059-021-02479-9.

62. Choi SW, O'Reilly PF. PRSice-2: Polygenic Risk Score software for biobank-scale data. *GigaScience*. 2019; doi: 10.1093/gigascience/giz082.

63. Gómez-Villamandos JC, Bautista MJ, Sánchez-Cordón PJ, Carrasco L. Pathology of African swine fever: The role of monocyte-macrophage. *Virus Research*. 2013; doi: 10.1016/j.virusres.2013.01.017.

64. Chen K, Magri G, Grasset EK, Cerutti A. Rethinking mucosal antibody responses: IgM, IgG and IgD join IgA. *Nat Rev Immunol*. Nature Publishing Group; 2020; doi: 10.1038/s41577-019-0261-1.
65. Vilček J, Feldmann M. Historical review: Cytokines as therapeutics and targets of therapeutics. *Trends in Pharmacological Sciences*. 2004; doi: 10.1016/j.tips.2004.02.011.
66. Karalyan Z, Zakaryan H, Sargsyan Kh, Voskanyan H, Arzumanyan H, Avagyan H, et al.. Interferon status and white blood cells during infection with African swine fever virus in vivo. *Veterinary Immunology and Immunopathology*. 2012; doi: 10.1016/j.vetimm.2011.12.013.
67. Gabriel C, Blome S, Malogolovkin A, Parilov S, Kolbasov D, Teifke JP, et al.. Characterization of African Swine Fever Virus Caucasus Isolate in European Wild Boars. *Emerging Infectious Diseases*. 2011; doi: 10.3201/eid1712.110430.
68. Walczak M, Żmudzki J, Mazur-Panasiuk N, Juszkiewicz M, Woźniakowski G. Analysis of the Clinical Course of Experimental Infection with Highly Pathogenic African Swine Fever Strain, Isolated from an Outbreak in Poland. Aspects Related to the Disease Suspicion at the Farm Level. *Pathogens*. Multidisciplinary Digital Publishing Institute; 2020; doi: 10.3390/pathogens9030237.
69. Bishop SC, Stear MJ. Modeling of host genetics and resistance to infectious diseases: understanding and controlling nematode infections. *Veterinary Parasitology*. 2003; doi: 10.1016/S0304-4017(03)00204-8.
70. Ozga AJ, Chow MT, Luster AD. Chemokines and the immune response to cancer. *Immunity*. 2021; doi: 10.1016/j.immuni.2021.01.012.
71. Steiner E, Holzmann K, Pirker C, Elbling L, Micksche M, Sutterlüty H, et al.. The major vault protein is responsive to and interferes with interferon-γ-mediated STAT1 signals. *Journal of Cell Science*. 2006; doi: 10.1242/jcs.02773.
72. Losert A, Lötsch D, Lackner A, Koppensteiner H, Peter-Vörösmarty B, Steiner E, et al.. The major vault protein mediates resistance to epidermal growth factor receptor inhibition in human hepatoma cells. *Cancer Letters*. 2012; doi: 10.1016/j.canlet.2012.01.002.
73. Charfi C, Levros L-C, Edouard E, Rassart E. Characterization and identification of PARM-1 as a new potential oncogene. *Mol Cancer*. 2013; doi: 10.1186/1476-4598-12-84.
74. Wang Y-H, Chan Y-T, Hung T-H, Hung J-T, Kuo M-W, Wang S-H, et al.. Transmembrane and coiled-coil domain family 3 (TMCC3) regulates breast cancer stem cell and AKT activation. *Oncogene*. 2021; doi: 10.1038/s41388-021-01729-1.
75. Jiang Y, Cao Y, Wang Y, Li W, Liu X, Lv Y, et al.. Cysteine transporter SLC3A1 promotes breast cancer tumorigenesis. *Theranostics*. 2017; doi: 10.7150/thno.18005.
76. Shi L, Wang L, Wang B, Cretoiu SM, Wang Q, Wang X, et al.. Regulatory mechanisms of betacellulin in CXCL8 production from lung cancer cells. *J Transl Med*. 2014; doi: 10.1186/1479-5876-12-70.

77. Galvez-Cancino F, Simpson AP, Costoya C, Matos I, Qian D, Peggs KS, et al.. Fcγ receptors and immunomodulatory antibodies in cancer. *Nat Rev Cancer*. 2024; doi: 10.1038/s41568-023-00637-8.

78. Küçükköse E, Peters NA, Ubink I, van Keulen VAM, Daghighian R, Verheem A, et al.. KIT promotes tumor stroma formation and counteracts tumor-suppressive TGFβ signaling in colorectal cancer. *Cell Death Dis*. 2022; doi: 10.1038/s41419-022-05078-z.

79. Chen W, Zhang L, Zhong G, Liu S, Sun Y, Zhang J, et al.. Regulation of microglia inflammation and oligodendrocyte demyelination by Engeletin via the TLR4/RRP9/NF-κB pathway after spinal cord injury. *Pharmacological Research*. 2024; doi: 10.1016/j.phrs.2024.107448.

80. Wu X, Fang J, Huang Q, Chen X, Guo Z, Tian L, et al.. Major Vault Protein Inhibits Porcine Reproductive and Respiratory Syndrome Virus Infection in CRL2843CD163 Cell Lines and Primary Porcine Alveolar Macrophages. *Viruses*. 2021; doi: 10.3390/v13112267.

81. Pan X, Taherzadeh M, Bose P, Heon-Roberts R, Nguyen ALA, Xu T, et al.. Glucosamine amends CNS pathology in mucopolysaccharidosis IIIC mouse expressing misfolded HGSNAT. *Journal of Experimental Medicine*. 2022; doi: 10.1084/jem.20211860.

82. Wang K, Li Y, Wang J, Chen R, Li J. A novel 12-gene signature as independent prognostic model in stage IA and IB lung squamous cell carcinoma patients. *Clin Transl Oncol*. 2021; doi: 10.1007/s12094-021-02638-1.

83. Huang C, Wang Y, Li D, Li Y, Luo J, Yuan W, et al.. Inhibition of transcriptional activities of AP-1 and c-Jun by a new zinc finger protein ZNF394. *Biochemical and Biophysical Research Communications*. 2004; doi: 10.1016/j.bbrc.2004.06.080.

84. Metsu S, Rainger JK, Debacker K, Bernhard B, Rooms L, Grafodatskaya D, et al.. A CGG-Repeat Expansion Mutation in ZNF713 Causes FRA7A: Association with Autistic Spectrum Disorder in Two Families. *Human Mutation*. 2014; doi: 10.1002/humu.22683.

85. Sun L, Ren C, Leng H, Wang X, Wang D, Wang T, et al.. Peripheral Blood Mononuclear Cell Biomarkers for Major Depressive Disorder: A Transcriptomic Approach. *Depress Anxiety*. 2024; doi: 10.1155/2024/1089236.

86. Meyer-Lindenberg A, Mervis CB, Sarpal D, Koch P, Steele S, Kohn P, et al.. Functional, structural, and metabolic abnormalities of the hippocampal formation in Williams syndrome. *J Clin Invest*. 2005; doi: 10.1172/JCI24892.

87. Wong M-L, Arcos-Burgos M, Liu S, Vélez JI, Yu C, Baune BT, et al.. The PHF21B gene is associated with major depression and modulates the stress response. *Mol Psychiatry*. 2017; doi: 10.1038/mp.2016.174.

88. Li Q, Ye L, Guo W, Wang M, Huang S, Peng X. PHF21B overexpression promotes cancer stem cell-like traits in prostate cancer cells by activating the Wnt/β-catenin signaling pathway. *Journal of Experimental & Clinical Cancer Research*. 2017; doi:

10.1186/s13046-017-0560-y.

89. Hamshere ML, Walters JTR, Smith R, Richards AL, Green E, Grozeva D, et al.. Genome-wide significant associations in schizophrenia to ITIH3/4, CACNA1C and SDCCAG8, and extensive replication of associations reported by the Schizophrenia PGC. *Mol Psychiatry*. 2013; doi: 10.1038/mp.2012.67.

90. Liu Z, Li H, Luo Y, Li J, Sun A, Ahmed Z, et al.. Comprehensive whole-genome resequencing unveils genetic diversity and selective signatures of the Xiangdong black goat. *Front Genet*. 2024; doi: 10.3389/fgene.2024.1326828.

91. Nonneman D, Lents CA, Rempel LA, Rohrer GA. Potential functional variants in AHR signaling pathways are associated with age at puberty in swine. *Animal Genetics*. 2021; doi: 10.1111/age.13051.

92. Cho K-H, Hong S-K, Kim D-Y, Jang M-K, Kim J-H, Lee H, et al.. Pathogenicity and Pathological Characteristics of African Swine Fever Virus Strains from Pig Farms in South Korea from 2022 to January 2023. *Pathogens*. 2023; doi: 10.3390/pathogens12091158.

93. Sokol CL, Luster AD. The Chemokine System in Innate Immunity. *Cold Spring Harb Perspect Biol*. 2015; doi: 10.1101/cshperspect.a016303.

94. Fawkner-Corbett D, Antanaviciute A, Parikh K, Jagielowicz M, Gerós AS, Gupta T, et al.. Spatiotemporal analysis of human intestinal development at single-cell resolution. *Cell*. 2021; doi: 10.1016/j.cell.2020.12.016.

95. Xie C, Lin Y, Qi C, Wang W, Yuan Y, Song D, et al.. Neuro-endocrine-immune regulation of metabolic homeostasis. *Cytokine & Growth Factor Reviews*. 2025; doi: 10.1016/j.cytogfr.2025.08.001.

96. Andrés G. African Swine Fever Virus Gets Undressed: New Insights on the Entry Pathway. *J Virol*. 2017; doi: 10.1128/JVI.01906-16.

97. Lv Y, Chen C, Han M, Tian C, Song F, Feng S, et al.. CXCL2: a key player in the tumor microenvironment and inflammatory diseases. *Cancer Cell International*. 2025; doi: 10.1186/s12935-025-03765-3.

98. Liu M, Guo S, Hibbert JM, Jain V, Singh N, Wilson NO, et al.. CXCL10/IP-10 in infectious diseases pathogenesis and potential therapeutic implications. *Cytokine & Growth Factor Reviews*. 2011; doi: 10.1016/j.cytogfr.2011.06.001.

99. Zakaryan H, Karalova E, Voskanyan H, Ter-Pogossyan Z, Nersisyan N, Hakobyan A, et al.. Evaluation of hemostaseological status of pigs experimentally infected with African swine fever virus. *Veterinary Microbiology*. 2014; doi: 10.1016/j.vetmic.2014.08.029.

100. Bel S, and Hooper LV. Secretory autophagy of lysozyme in Paneth cells. *Autophagy*. 2018; doi: 10.1080/15548627.2018.1430462.

101. Kalafati L, Kourtzelis I, Schulte-Schrepping J, Li X, Hatzioannou A, Grinenko T, et al.. Innate Immune Training of Granulopoiesis Promotes Anti-tumor Activity. *Cell*. 2020; doi: 10.1016/j.cell.2020.09.058.

102. Visscher PM, Wray NR, Zhang Q, Sklar P, McCarthy MI, Brown MA, et al.. 10

Years of GWAS Discovery: Biology, Function, and Translation. *The American Journal of Human Genetics*. 2017; doi: 10.1016/j.ajhg.2017.06.005.

103. Bulik-Sullivan B, Finucane HK, Anttila V, Gusev A, Day FR, Loh P-R, et al.. An atlas of genetic correlations across human diseases and traits. *Nat Genet*. 2015; doi: 10.1038/ng.3406.

104. Eustace Montgomery R. On A Form of Swine Fever Occurring in British East Africa (Kenya Colony). *Journal of Comparative Pathology and Therapeutics*. 1921; doi: 10.1016/S0368-1742(21)80031-4.

105. Yamamoto A, Nagano T, Takehara S, Hibi M, Aizawa S. Shisa Promotes Head Formation through the Inhibition of Receptor Protein Maturation for the Caudalizing Factors, Wnt and FGF. *Cell*. Elsevier; 2005; doi: 10.1016/j.cell.2004.11.051.

106. Ouyang Y-B, Crawley JTB, Aston CE, Moore KL. Reduced Body Weight and Increased Postimplantation Fetal Death in Tyrosylprotein Sulfotransferase-1-deficient Mice. *Journal of Biological Chemistry*. Elsevier; 2002; doi: 10.1074/jbc.M202420200.

107. Li Y-T, Shao W-Q, Chen Z-M, Ma X-C, Yi C-H, Tao B-R, et al.. GOLM1 promotes cholesterol gallstone formation via ABCG5-mediated cholesterol efflux in metabolic dysfunction-associated steatohepatitis livers. *Clin Mol Hepatol*. The Korean Association for the Study of the Liver; 2024; doi: 10.3350/cmh.2024.0657.

108. Khera AV, Chaffin M, Aragam KG, Haas ME, Roselli C, Choi SH, et al.. Genome-wide polygenic scores for common diseases identify individuals with risk equivalent to monogenic mutations. *Nat Genet*. 2018; doi: 10.1038/s41588-018-0183-z.

109. Torkamani A, Wineinger NE, Topol EJ. The personal and clinical utility of polygenic risk scores. *Nat Rev Genet*. 2018; doi: 10.1038/s41576-018-0018-x.

110. Whitworth KM, Rowland RRR, Ewen CL, Trible BR, Kerrigan MA, Cino-Ozuna AG, et al.. Gene-edited pigs are protected from porcine reproductive and respiratory syndrome virus. *Nat Biotechnol*. Nature Publishing Group; 2016; doi: 10.1038/nbt.3434.

111. Xu K, Zhou Y, Mu Y, Liu Z, Hou S, Xiong Y, et al.. CD163 and pAPN double-knockout pigs are resistant to PRRSV and TGEV and exhibit decreased susceptibility to PDCoV while maintaining normal production performance. Davenport MP, Carette JE, Tuggle CK, editors. *eLife*. eLife Sciences Publications, Ltd; 2020; doi: 10.7554/eLife.57132.

112. Pannhorst K, Carlson J, Hölper JE, Grey F, Baillie JK, Höper D, et al.. The non-classical major histocompatibility complex II protein SLA-DM is crucial for African swine fever virus replication. *Sci Rep*. 2023; doi: 10.1038/s41598-023-36788-9.

113. Palgrave CJ, Gilmour L, Lowden CS, Lillico SG, Mellencamp MA, Whitelaw CBA. Species-Specific Variation in RELA Underlies Differences in NF-κB Activity: a Potential Role in African Swine Fever Pathogenesis. *J Virol*. 2011; doi: 10.1128/JVI.00331-11.

114. Wang Z, Zhang Z, Chen Z, Sun J, Cao C, Wu F, et al.. PHARP: a pig haplotype reference panel for genotype imputation. *Sci Rep*. 2022; doi: 10.1038/s41598-022-

15851-x.

115. Chen S, Zhou Y, Chen Y, Gu J. fastp: an ultra-fast all-in-one FASTQ preprocessor. *Bioinformatics*. 2018; doi: 10.1093/bioinformatics/bty560.

116. Warr A, Affara N, Aken B, Beiki H, Bickhart DM, Billis K, et al.. An improved pig reference genome sequence to enable pig genetics and genomics research. *Gigascience*. 2020; doi: 10.1093/gigascience/giaa051.

117. Li H, Durbin R. Fast and accurate short read alignment with Burrows-Wheeler transform. *Bioinformatics*. 2009; doi: 10.1093/bioinformatics/btp324.

118. Li H, Handsaker B, Wysoker A, Fennell T, Ruan J, Homer N, et al.. The Sequence Alignment/Map format and SAMtools. *Bioinformatics*. 2009; doi: 10.1093/bioinformatics/btp352.

119. Tarasov A, Vilella AJ, Cuppen E, Nijman IJ, Prins P. Sambamba: fast processing of NGS alignment formats. *Bioinformatics*. 2015; doi: 10.1093/bioinformatics/btv098.

120. Pedersen BS, Quinlan AR. Mosdepth: quick coverage calculation for genomes and exomes. *Bioinformatics*. 2018; doi: 10.1093/bioinformatics/btx699.

121. McKenna A, Hanna M, Banks E, Sivachenko A, Cibulskis K, Kernytsky A, et al.. The Genome Analysis Toolkit: A MapReduce framework for analyzing next-generation DNA sequencing data. *Genome Research*. Cold Spring Harbor Laboratory Press; 2010; doi: 10.1101/gr.107524.110.

122. Danecek P, Auton A, Abecasis G, Albers CA, Banks E, DePristo MA, et al.. The variant call format and VCFtools. *Bioinformatics*. 2011; doi: 10.1093/bioinformatics/btr330.

123. Browning SR, Browning BL. Rapid and accurate haplotype phasing and missing-data inference for whole-genome association studies by use of localized haplotype clustering. *Am J Hum Genet*. 2007; doi: 10.1086/521987.

124. Chang CC, Chow CC, Tellier LC, Vattikuti S, Purcell SM, Lee JJ. Second-generation PLINK: rising to the challenge of larger and richer datasets. *Gigascience*. 2015; doi: 10.1186/s13742-015-0047-8.

125. Tamura K, Stecher G, Kumar S. MEGA11: Molecular Evolutionary Genetics Analysis Version 11. *Mol Biol Evol*. 2021; doi: 10.1093/molbev/msab120.

126. Letunic I, Bork P. Interactive Tree Of Life (iTOL) v5: an online tool for phylogenetic tree display and annotation. *Nucleic Acids Res*. 2021; doi: 10.1093/nar/gkab301.

127. Dominguez Mantes A, Mas Montserrat D, Bustamante CD, Giró-i-Nieto X, Ioannidis AG. Neural ADMIXTURE for rapid genomic clustering. *Nat Comput Sci*. 2023; doi: 10.1038/s43588-023-00482-7.

128. Fonseca PAS, Suárez-Vega A, Marras G, Cánovas Á. GALLO: An R package for genomic annotation and integration of multiple data sources in livestock for positional candidate loci. *Gigascience*. 2020; doi: 10.1093/gigascience/giaa149.

129. Yang J, Lee SH, Goddard ME, Visscher PM. GCTA: A Tool for Genome-wide Complex Trait Analysis. *Am J Hum Genet*. 2011; doi: 10.1016/j.ajhg.2010.11.011.

130. Zhou X, Stephens M. Genome-wide efficient mixed-model analysis for association studies. *Nat Genet*. Nature Publishing Group; 2012; doi: 10.1038/ng.2310.
131. Yang J, Jin Z-B, Chen J, Huang X-F, Li X-M, Liang Y-B, et al.. Genetic signatures of high-altitude adaptation in Tibetans. *Proc Natl Acad Sci U S A*. 2017; doi: 10.1073/pnas.1617042114.
132. Yang J, Zaitlen NA, Goddard ME, Visscher PM, Price AL. Advantages and pitfalls in the application of mixed-model association methods. *Nat Genet*. Nature Publishing Group; 2014; doi: 10.1038/ng.2876.
133. Zhang Z, Chen Z, Teng J, Liu S, Lin Q, Wu J, et al.. FarmGTEx TWAS-server: An Interactive Web Server for Customized TWAS Analysis. *Genomics, Proteomics & Bioinformatics*. 2025; doi: 10.1093/gpbjnl/qzaf006.
134. Wu Y, Zeng J, Zhang F, Zhu Z, Qi T, Zheng Z, et al.. Integrative analysis of omics summary data reveals putative mechanisms underlying complex traits. *Nat Commun*. 2018; doi: 10.1038/s41467-018-03371-0.
135. Wallace C. A more accurate method for colocalisation analysis allowing for multiple causal variants. *PLOS Genetics*. 2021; doi: 10.1371/journal.pgen.1009440.
136. Klunk J, Vilgalys TP, Demeure CE, Cheng X, Shiratori M, Madej J, et al.. Evolution of immune genes is associated with the Black Death. *Nature*. 2022; doi: 10.1038/s41586-022-05349-x.
137. Yu G, Wang L-G, Han Y, He Q-Y. clusterProfiler: an R Package for Comparing Biological Themes Among Gene Clusters. *OMICS*. 2012; doi: 10.1089/omi.2011.0118.
138. Yu G, He Q-Y. ReactomePA: an R/Bioconductor package for reactome pathway analysis and visualization. *Mol BioSyst*. 2016; doi: 10.1039/C5MB00663E.
139. Finucane HK, Reshef YA, Anttila V, Slowikowski K, Gusev A, Byrnes A, et al.. Heritability enrichment of specifically expressed genes identifies disease-relevant tissues and cell types. *Nat Genet*. 2018; doi: 10.1038/s41588-018-0081-4.
140. Sayers EW, Bolton EE, Brister JR, Canese K, Chan J, Comeau DC, et al.. Database resources of the national center for biotechnology information. *Nucleic Acids Research*. 2022; doi: 10.1093/nar/gkab1112.
141. Chen T, Chen X, Zhang S, Zhu J, Tang B, Wang A, et al.. The Genome Sequence Archive Family: Toward Explosive Data Growth and Diverse Data Types. *Genomics, Proteomics & Bioinformatics*. 2021; doi: 10.1016/j.gpb.2021.08.001.
142. CNCB-NGDC Members and Partners. Database Resources of the National Genomics Data Center, China National Center for Bioinformation in 2024. *Nucleic Acids Research*. 2024; doi: 10.1093/nar/gkad1078.
143. Zhao D, Wang N, Feng X, Zhang Z, Xu K, Zheng T, et al.. Transcription regulation of African swine fever virus: dual role of M1249L. *Nat Commun*. 2024; doi: 10.1038/s41467-024-54461-1.
144. Kim D, Paggi JM, Park C, Bennett C, Salzberg SL. Graph-Based Genome Alignment and Genotyping with HISAT2 and HISAT-genotype. *Nat Biotechnol*. 2019; doi: 10.1038/s41587-019-0201-4.

145. Liao Y, Smyth GK, Shi W. featureCounts: an efficient general purpose program for assigning sequence reads to genomic features. *Bioinformatics*. 2014; doi: 10.1093/bioinformatics/btt656.

146. Yanai I, Benjamin H, Shmoish M, Chalifa-Caspi V, Shklar M, Ophir R, et al.. Genome-wide midrange transcription profiles reveal expression level relationships in human tissue specification. *Bioinformatics*. 2005; doi: 10.1093/bioinformatics/bti042.

147. Thériault S, Li Z, Abner E, Luan J, Manikpurage HD, Houessou U, et al.. Integrative genomic analyses identify candidate causal genes for calcific aortic valve stenosis involving tissue-specific regulation. *Nat Commun*. 2024; doi: 10.1038/s41467-024-46639-4.

148. Leek JT, Johnson WE, Parker HS, Jaffe AE, Storey JD. The sva package for removing batch effects and other unwanted variation in high-throughput experiments. *Bioinformatics*. 2012; doi: 10.1093/bioinformatics/bts034.

149. Love MI, Huber W, Anders S. Moderated estimation of fold change and dispersion for RNA-seq data with DESeq2. *Genome Biol*. 2014; doi: 10.1186/s13059-014-0550-8.

150. Nueda MJ, Tarazona S, Conesa A. Next maSigPro: updating maSigPro bioconductor package for RNA-seq time series. *Bioinformatics*. 2014; doi: 10.1093/bioinformatics/btu333.

151. Langmead B, Salzberg SL. Fast gapped-read alignment with Bowtie 2. *Nat Methods*. Nature Publishing Group; 2012; doi: 10.1038/nmeth.1923.

152. Zheng GXY, Terry JM, Belgrader P, Ryvkin P, Bent ZW, Wilson R, et al.. Massively parallel digital transcriptional profiling of single cells. *Nat Commun*. 2017; doi: 10.1038/ncomms14049.

153. Hao Y, Stuart T, Kowalski MH, Choudhary S, Hoffman P, Hartman A, et al.. Dictionary learning for integrative, multimodal and scalable single-cell analysis. *Nat Biotechnol*. 2023; doi: 10.1038/s41587-023-01767-y.

154. Edahiro R, Shirai Y, Takeshima Y, Sakakibara S, Yamaguchi Y, Murakami T, et al.. Single-cell analyses and host genetics highlight the role of innate immune cells in COVID-19 severity. *Nat Genet*. 2023; doi: 10.1038/s41588-023-01375-1.

Figure legends

Figure 1. Study overview.

A schematic representation of the study design and analytical framework:
Experimental grouping and comparative analyses (red): Summarizes experimental groupings, comparisons, and analyses, including GWAS, F_{ST}, AF tests, and gene identification. Pig icons are color-coded by clinical phenotype: blue for uninfected (Antigen-/Antibody-), green for survived (Antigen-/Antibody+), and red for deceased (Antigen+). Grey icons represent a background population used for F_{ST} and AF tests. The "+" sign indicates the integration of association signals from GWAS and population genetic differentiation.
Gene prioritization (yellow): ASF resistance candidate genes were prioritized using five independent methods—overlap with reported immune genes, TWAS, SMR, Coloc, and BN-GWAS. These analyses identified 135 high-priority genes, which were further analyzed through pathway enrichment to elucidate their biological roles. Numerical ratios (e.g., 31/64) indicate the yield of each method: the numerator denotes prioritized genes, while the denominator represents total candidates processed by that analysis. The double-headed arrow (⟷) in TWAS denotes associations between gene expression and traits; the single-headed arrow (→) in SMR/Coloc indicates potential causal or regulatory effects from eQTL to phenotype. The network icon in BN-GWAS represents inferred gene-gene-phenotype causal relationships.
Tissue and cell-Specific analyses (green): Heritability enrichment pinpointed tissues and cell types (e.g., PAM) associated with ASF resistance. Bulk and single-cell transcriptomics data integration revealed basal tissue-specific expression patterns (ESS, TAU) and dynamic gene responses (DEG) post-infection. The meaning of numerical ratios is the same as in the yellow section.
Polygenic associations (blue): Global genetic correlations between ASF resistance and 269 other pig traits were investigated. Shared genomic regions, trait enrichment, and cross-trait colocalization analyses highlighted the polygenic nature of prioritized genes. Associations between the APRS and PGS for 59 pig health traits were evaluated to assess the biological consistency and robustness of the genetic prediction.
AF test: allele frequency chi-square test. GWAS: genome-wide association study. F_{ST}: fixaxion index. TWAS: transcriptome-wide association study. SMR: summary-based Mendelian randomization. Coloc: colocalization. BN-GWAS: bayesian network GWAS. PAM: porcine alveolar macrophage. ESS: expression specificity scores. TAU: tissue-specific gene expression. DEG: differentially expressed genes. PAM: porcine alveolar macrophage. PBMC: peripheral blood mononuclear cell. PGS: polygenic score. APRS: African swine fever resistance prediction score.

Figure 2. Integrative identification and genomic landscape of candidate genes associated with ASF resistance.

a. Overlap of candidate genes across analytical comparisons. The Upset plot illustrates the intersection of genes identified in four clinical comparisons (1–4). The vertical bars indicate the number of genes unique to or shared between comparisons, as indicated by the connected dots below.
b. Fuji plot showing the genomic distribution of identified loci. The outermost circle displays chromosomes with annotated prioritized genes. In the middle layers, each dot represents a significant locus associated with a specific comparison (1-4). Within each categorical track, the concentric rings (from outer to inner) represent five distinct statistical methods: GWAS, F_{ST} (case *vs.* control 1), AF test (case *vs.* control 1), F_{ST} (case *vs.* control 2), and AF test (case *vs.* control 2).
c. Methodological support for candidate loci. The inner stacked bar plot summarizes the cumulative number of statistical methods (out of the five mentioned above) supporting the candidate loci within each genomic region. The height and color composition of each bar directly correspond to the loci displayed in part **b**.
Comparison 1: (Uninfected + Survived) *vs.* Dead (General resistance/protection); Comparison 2: Uninfected vs. (Survived + Dead) (Pathogen recognition/avoidance); Comparison 3: Uninfected vs. Dead (Strict protection); Comparison 4: Survived vs. Dead (Adaptive immunity/recovery). GWAS: genome-wide association study; F_{ST}: fixation index; AF test: allele frequency chi-square test.

Figure 3. Gene prioritization and functional analyses.

a. Overview of the gene prioritization framework for ASF resistance. Genes are ranked by their aggregate prioritization scores (from high to low). The heatmap displays individual scores across various genomic methods (GWAS, F_{ST}, Reported immune genes, TWAS, SMR, colocalization, BN-GWAS), with color intensity representing score values. The lollipop plot illustrates the aggregate prioritization score, calculated by summing individual scores. Genes were identified as prioritized candidates if they achieved an aggregate score $\geqslant$ 3.5 or were supported by two or more independent methods.
b. Venn diagram illustrating the overlap among 1,102 candidate genes (light blue), 135 prioritized genes (dark blue), and 1,150 previously reported immune-related genes (green).
c. Manhattan plot of 13 candidate genes identified by transcriptome-wide association study (TWAS) (FDR < 0.05, red dashed line), with 11 categorized as prioritized genes. The y-axis shows $-\log_{10}$FDR, the x-axis represents genomic locations, and dot colors indicate correlation direction: orange for positive and blue for negative.
d. 42 causal pairs supported by colocalization (coloc) and summary-based Mendelian randomization (SMR) analyses, demonstrating a causal relationship between gene

expression in tissues and ASF resistance. Outer to inner rings represent: tissue sectors, -log10p-value from SMR, PP4 from coloc, PP3 from coloc, SMR beta value (red for positive, blue for negative), and a cross-tissue gene network. PP4: posterior probability of hypothesis 4; PP3: posterior probability of hypothesis 3.

e. Inference of 14 candidate genes by Bayesian network GWAS (BN-GWAS), with 12 identified as prioritized genes. The x-axis shows the effect size.

f. Bubble plot showing significant enrichment of 135 prioritized genes. The x-axis represents enrichment fold, bubble color denotes $-\log_{10}$FDR, and bubble shape reflects database categories.

Figure 4. Tissue- and cell-specificity and gene expression analyses.

a. Manhattan plot depicting genetic enrichment significance for four comparisons across 34 tissues and eight PAM cell types. The y-axis shows -log10p-value, and the x-axis represents tissues/cell types. Blue and red dashed lines indicate significance thresholds (p-value < 0.05 and p-value < 0.01, respectively). Significant tissues and cell types are highlighted in orange and labeled.

b. Baseline expression and dynamic response of prioritized genes to ASF infection in bulk RNA data. The y-axis lists prioritized genes, while the x-axis, from left to right, displays: primary tissue expression distribution (color-coded squares); expression specificity scores (ESS) values across tissues, with heatmap colors indicating magnitude (red: high, blue: low); tissue-specific gene expression (TAU) values, where TAU > 0.8 denotes high tissue specificity, and TAU < 0.6 indicates widespread expression; differential expression of prioritized genes at various infection time points, with point size representing $-\log_{10}$FDR and color indicating $\log_2$FC. Time units are hours post-infection (hpi) for PAM and days post-infection (dpi) for other tissues.

c. UMAP visualization of cell types in PAM scRNA-seq data.

d. Module scoring of the prioritized gene subset in PAM scRNA-seq. The score represents the aggregate expression difference of 127 prioritized genes (a detectable subset of the 135 total prioritized genes) pre- and post-infection. Results are derived using a two-sided Welch t-test (*p-value < 0.001, **p-value < 1×10^{-8}). The y-axis indicates infection time (hpi), the x-axis shows cell types, dot size reflects $-\log_{10}p$-value, and dot color represents statistical measures.

e. Differential expression of prioritized genes across PAM cell types. Square colors represent $\log_2$FC values, with significance marked as *p-value < 0.05 and $|\log_2\text{FC}| > 1$. Mac: macrophages. Mast: mast cells. T: T cells. Pro: proliferating cells. Epi: epithelial cells. Four macrophage subtypes: Mac_CD163, Mac_HLA_DRA, Mac_CREG1, and Mac_PLBD1.

Figure 5. Analyses of ASFV replication and host correlations.

a. ASFV load, measured by RNA reads per million (RPM), across multiple tissues at

various time points post-infection. **b.** Spearman correlation analysis between ASFV RPM and the expression (TPM) of prioritized host genes.

Figure 6. Pleiotropic associations.

a. Forest plot illustrating traits with significant genome-wide genetic correlations with ASF resistance (*p*-value < 0.05). Orange dots indicate positive correlations, and blue dots indicate negative correlations. Each dot represents the effect value, and lines indicate the 95% confidence intervals (CIs). S_PLDWID: platelet distribution width. S_RCDW: red cell distribution width.

b. Bubble plot illustrating the enrichment of prioritized genes in various pig traits. The y-axis lists traits, while the x-axis represents the enrichment fold. Bubble size corresponds $-\log_{10}$FDR, and bubble color reflects the trait categories.

c. Cross-trait colocalization analysis highlighting genes shared between ASF resistance and other pig traits. The y-axis lists traits, and the x-axis represents genes. Shades of orange denote PP4 values (PP4 > 0.7, evidence for shared causal variants), while shades of blue denote PP3 values (PP3 > 0.7, evidence for alternative causal variants). PP4: posterior probability of hypothesis 4; PP3: posterior probability of hypothesis 3.

d. Schematic representation of the construction and evaluation process for the ASF resistance prediction score (APRS) and polygenic scores (PGS) related to other pig health traits.

e. Comparative analysis of "ideal" APRS versus randomly constructed APRS. The Wilcoxon rank-sum test with Bonferroni correction was used (***p*.adjusted < 0.01, *****p*.adjusted < 1×10^{-5}, ns: not significant). The y-axis shows APRS scores, and the x-axis differentiates experimental groupings. Colors distinguish APRS methods: Ideal (ideal APRS), Random allele (APRS using random alleles), and Random loci (APRS using random loci).

f. Phenome-wide association study (PheWAS) comparing APRS and PGS for pig health traits. The x-axis lists health traits, while the y-axis displays association metrics: Outer Plot (Panels 1 and 4): The y-axis shows $-\log_{10}p$-values from Spearman rank correlation analysis. Dot colors indicate association direction (red for positive, blue for negative), with shades reflecting correlation strength (|ρ|). The black dashed line represents the significance threshold (*p*-value < 0.05/236, Bonferroni correction). Significant traits are labeled. Inner Plot (Panels 2 and 3): Regression analysis of PGS values for individuals grouped by APRS deciles (top 10%, mid-50%, bottom 10%). The y-axis represents effect estimates, with dots showing effect sizes (**p*-value < 0.05, ***p*-value < 0.01, ****p*-value < 0.05/236, Bonferroni correction). Line segments represent 95% confidence intervals (CIs). Dot colors indicate effect magnitude (red for positive, blue for negative), and the red dashed line marks an effect of 0. Trait abbreviations and details are provided in the PigBiobank database.

Tables

Table 1. Grouping of individuals in experimental pig herds.

Group	Antigen[1]	Antibody[2]	Situations	Meaning	Sample size
A	/	+	Dead	Antigen infection → immune response → production of antibodies → pigs die	108
B	-	+	Alive	Antigen infection → immune response → production of antibodies → antigen clearance.	222
C	-	-	Alive	1 Antigen uninfected → no immune response → no antibody<br>2 Antigen infection → immune response → antibody → antigen clearance → antibody inactivation	144
D	/	/	/	The remaining control varieties in PHARP (except Laiwu, Lulai and Duroc x Lulai pigs)	1730

[1] ASFV antigen test: Detected via the RAA fluorescence method targeting the *KP177R* (p22 protein), - for negative and + for positive.
[2] ASFV antibody test: Detected via indirect ELISA targeting structural proteins P32, P62, and P72, - for negative and + for positive.

Table 2. Grouping of experimental comparisons between groups.

Comparison	Case	Control		Phenotype	Potential Meaning
		1	2		
1	B+C	A	D	(Uninfected + survived infection) *vs.* (Dead)	General resistance, Protection

2	C	A+B	D	(Uninfected) *vs.* (Survived infection + dead)	Pathogen recognition, Avoidance
3	C	A	D	(Uninfected) *vs.* (Dead)	Strict protection
4	B	A	D	(Survived infection) *vs.* (Dead)	Adaptive immunity, Recovery

Table 3. Statistical overview of candidate loci associated with African swine fever.

Comparison[1]	N[2]	Me[3]	Candidate loci		Genes	
			GWAS[4]	F_{ST}[5]	GWAS	F_{ST}
1	23,290,599	2,581,021	719	3,490	43	44
2	23,290,599	2,581,021	56	5,163	15	33
3	23,160,238	2,537,788	2,445	9,453	715	51
4	23,403,868	2,571,190	72	1,848	12	30

[1]Comparisons 1–4 are defined as follows based on clinical outcomes: 1, (Uninfected + Survived) *vs.* Dead; 2, Uninfected *vs.* (Survived + Dead); 3, Uninfected *vs.* Dead; 4, Survived *vs.* Dead. Detailed groupings are provided in Table 2.
[2]The number of all loci.
[3]The number of loci after LD pruning.
[4]The number of loci identified by GWAS method.
[5]The number of loci identified by F_{ST} method.

Table 4. Scoring framework for ASF resistance gene prioritization.

Method	Repeat count	Score Range	Scoring Formula
GWAS	1 per group	0-3	count * 1
F_{ST} + AF test	1 per group	0-3	count * 1
Previously Reported Immune Genes	/	0-1	count * 0.5
TWAS	1 per group, per tissue	0-4	count * 1.2
SMR	1 per group, per tissue	0-43	$\log_2$(count +1) * 0.8
Colocalization	1 per group, per tissue	0-25	$\log_2$(count +1) * 0.8
Bayesian Network GWAS	1 per group, per tissue	0-4	count * 1.2
Independent Inference	/	0-3	2 methods supported: +1; 3–4 methods supported: +2; ≥5 methods supported: +3

Supplementary files

Supplementary Figure S1-S19

Supplementary Table S1-S22

Figures

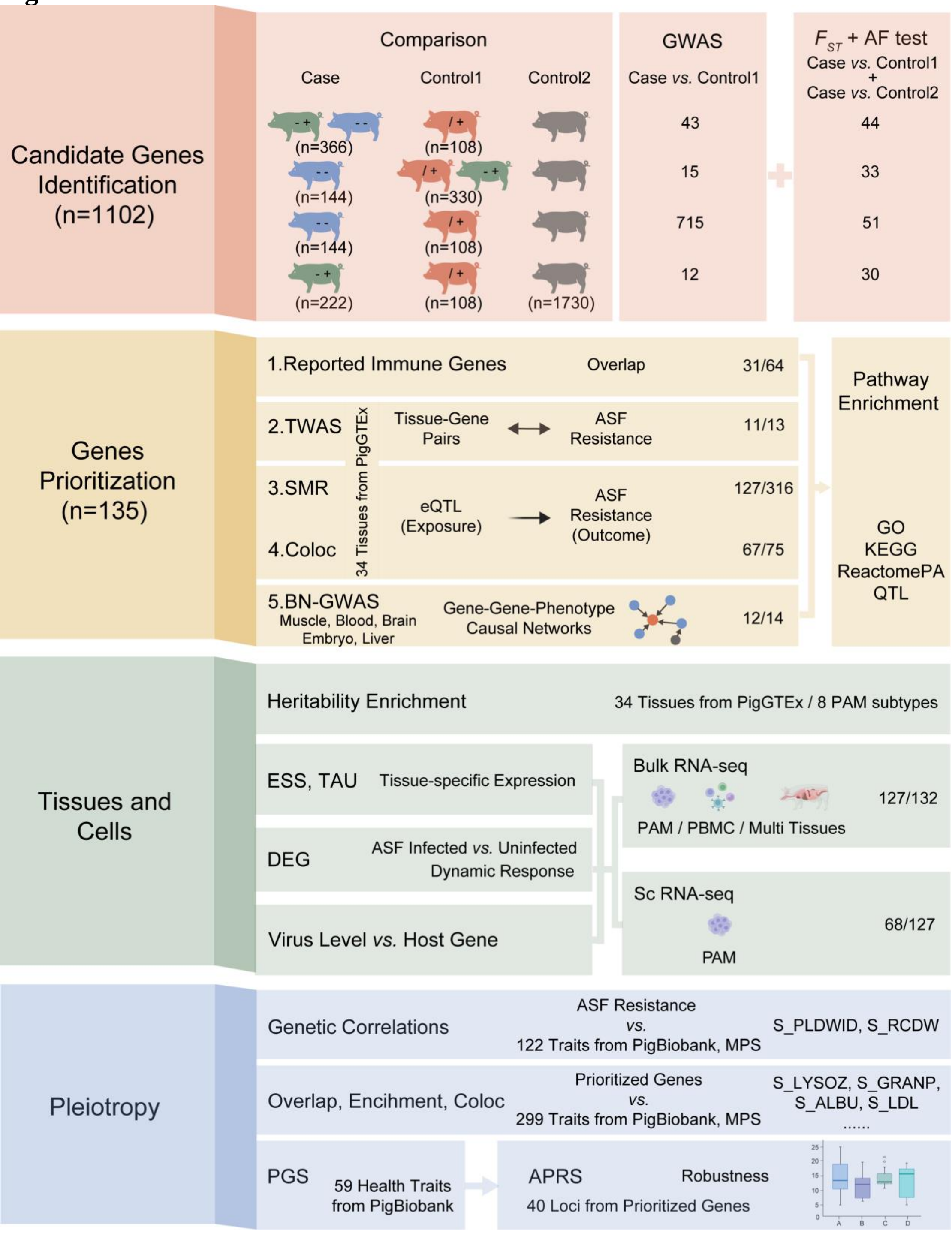

Figure 1.

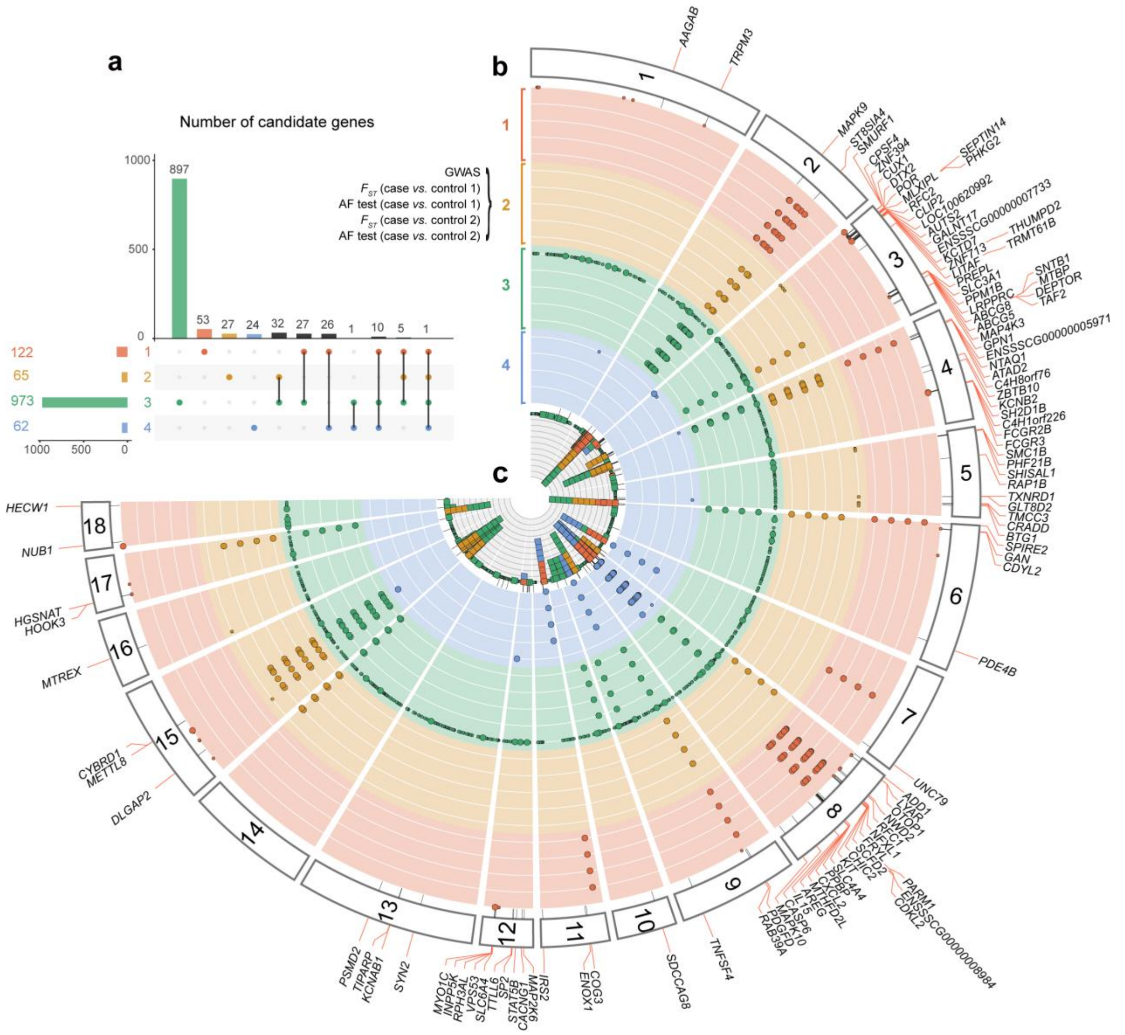
a
Number of candidate genes
GWAS
F_{ST} (case vs. control 1)
AF test (case vs. control 1)
F_{ST} (case vs. control 2)
AF test (case vs. control 2)
1000
500
0
897
53
27
24
32
27
26
1
10
5
1
122
65
973
62
1000 500 0
b
c
1
2
3
4
AAGAB
TRPM3
MAPK9
PDE4B
UNC79
SDCCAG8
TNFSF4
COG3
ENOX1
IRS2
SYN2
PSMD2
TIPARP
KCNAB1
DLGAP2
CYBRD1
METTL8
MTREX
HGSNAT
HOOK3
NUB1
HECW1

Figure 2.

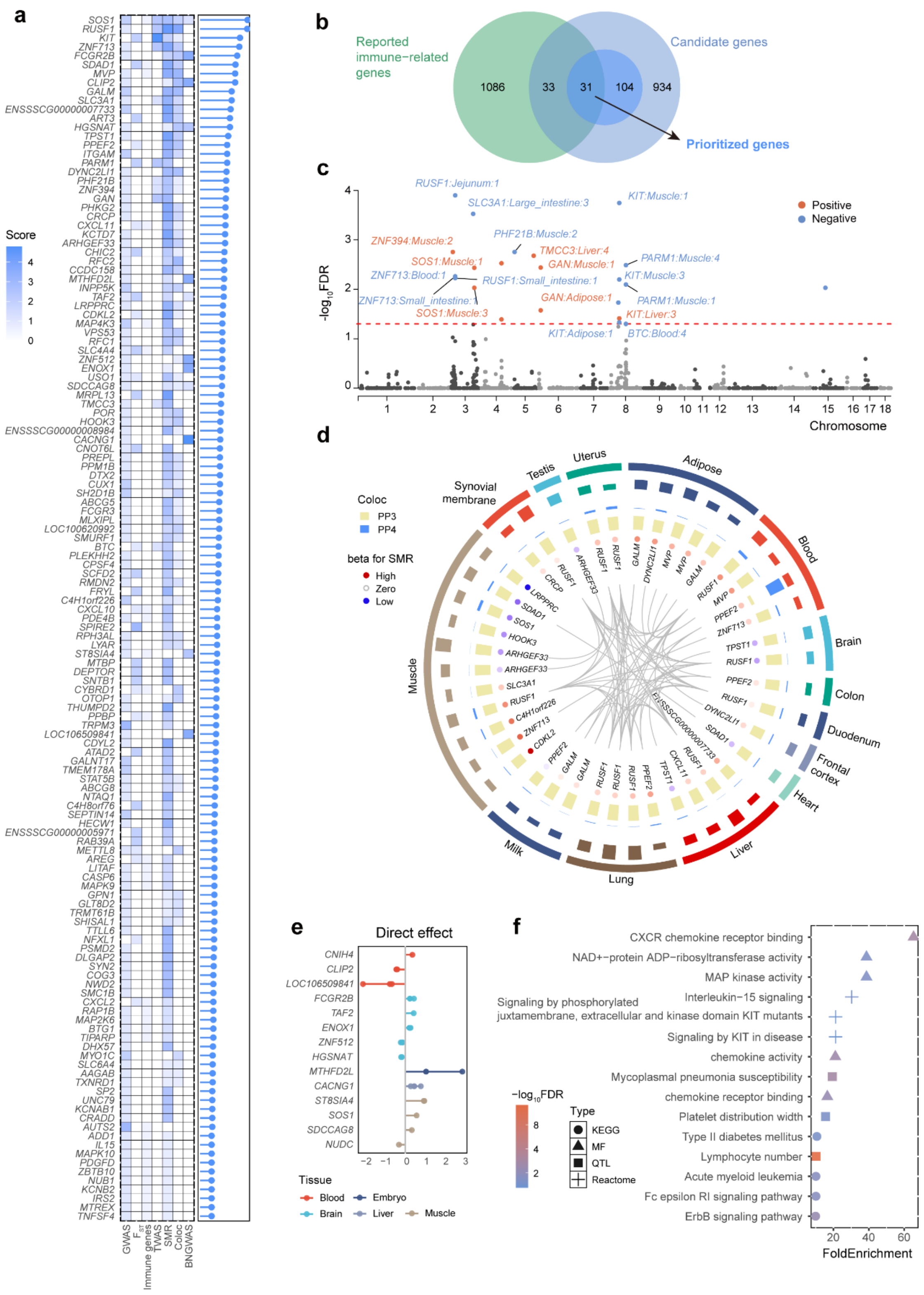

1789

1790 **Figure 3.**

1791

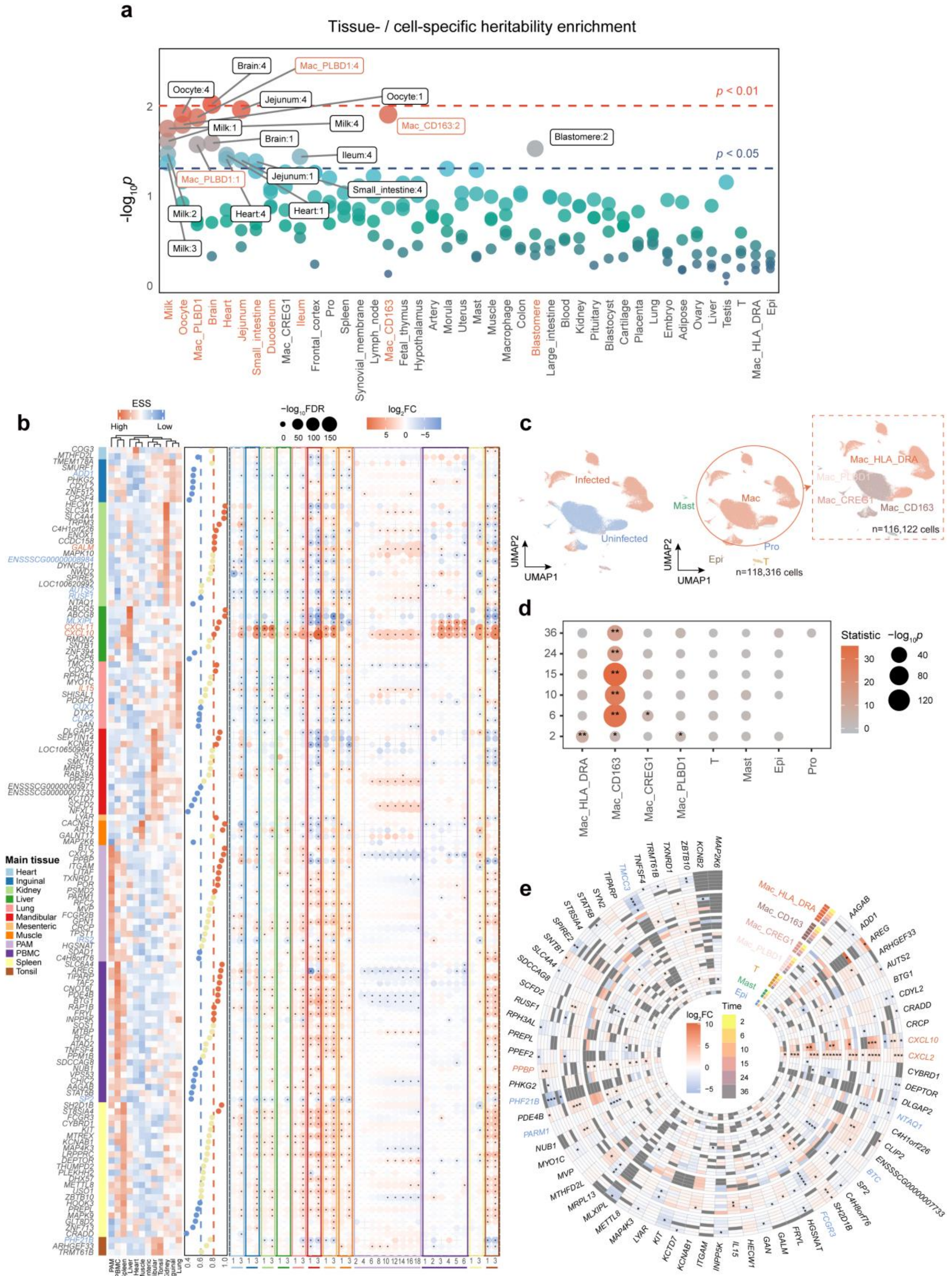
a
Tissue- / cell-specific heritability enrichment
p < 0.01
p < 0.05
-log10p
Brain:4
Mac_PLBD1:4
Oocyte:4
Jejunum:4
Oocyte:1
Milk:1
Milk:4
Mac_CD163:2
Brain:1
Ileum:4
Blastomere:2
Jejunum:1
Mac_PLBD1:1
Small_intestine:4
Milk:2
Heart:4
Heart:1
Milk:3
Milk
Oocyte
Mac_PLBD1
Brain
Heart
Jejunum
Small_intestine
Duodenum
Mac_CREG1
Ileum
Frontal_cortex
Pro
Spleen
Synovial_membrane
Lymph_node
Mac_CD163
Fetal_thymus
Hypothalamus
Artery
Morula
Uterus
Mast
Muscle
Macrophage
Colon
Blastomere
Large_intestine
Blood
Kidney
Pituitary
Blastocyst
Cartilage
Placenta
Lung
Embryo
Adipose
Ovary
Liver
Testis
T
Mac_HLA_DRA
Epi
b
ESS
High
Low
−log10FDR
log2FC
Main tissue
Heart
Inguinal
Kidney
Liver
Lung
Mandibular
Mesenteric
Muscle
PAM
PBMC
Spleen
Tonsil
c
Infected
Uninfected
Mast
Mac
Pro
Epi
T
n=118,316 cells
Mac_HLA_DRA
Mac_PLBD1
Mac_CREG1
Mac_CD163
n=116,122 cells
UMAP1
UMAP2
d
Statistic
−log10p
Mac_HLA_DRA
Mac_CD163
Mac_CREG1
Mac_PLBD1
T
Mast
Epi
Pro
e
log2FC
Time
CXCL10
CXCL2

Figure 4.

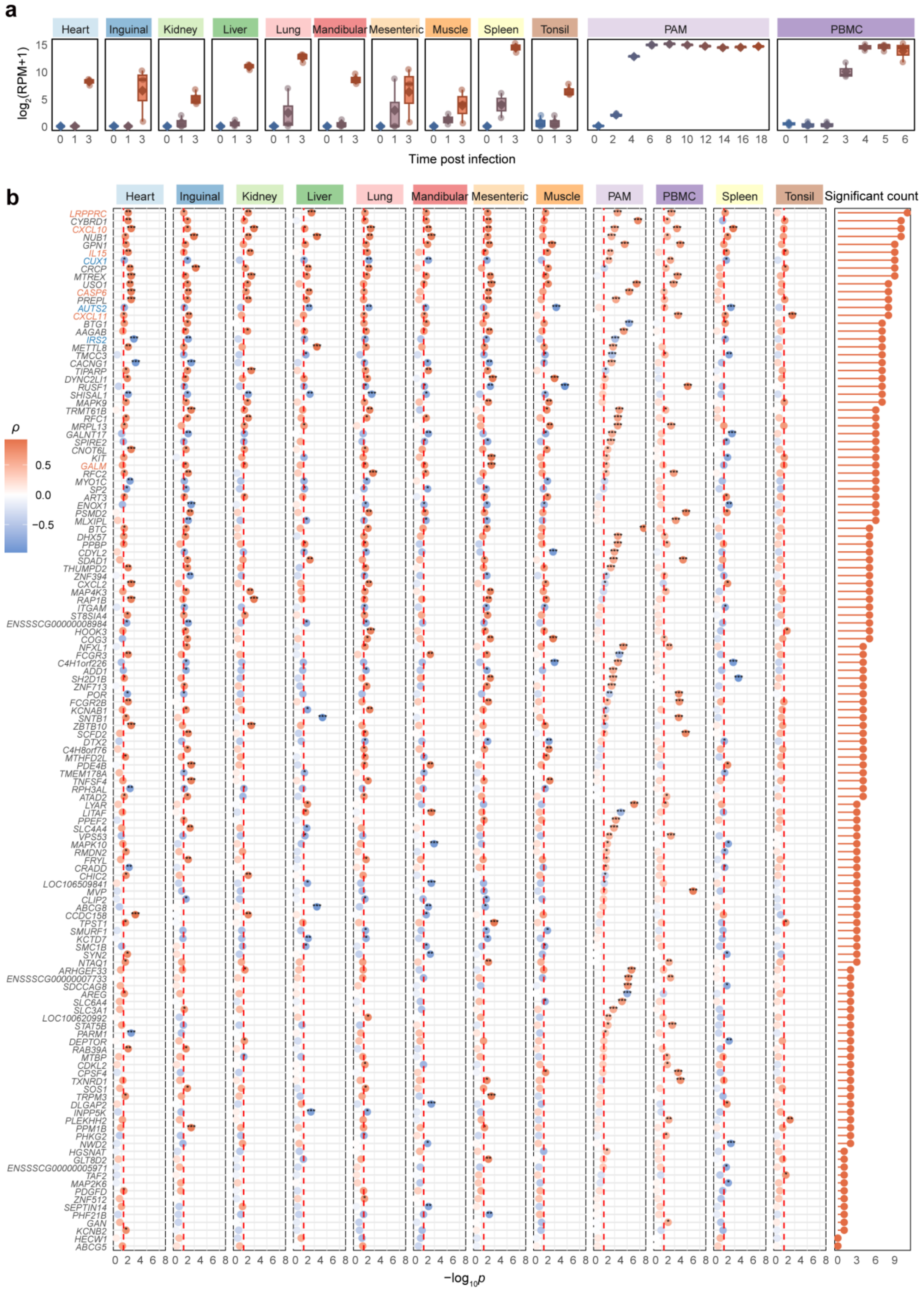

a
Heart
Inguinal
Kidney
Liver
Lung
Mandibular
Mesenteric
Muscle
Spleen
Tonsil
PAM
PBMC
log2(RPM+1)
Time post infection
b
Heart
Inguinal
Kidney
Liver
Lung
Mandibular
Mesenteric
Muscle
PAM
PBMC
Spleen
Tonsil
Significant count
ρ
LRPPRC
CYBRD1
CXCL10
NUB1
GPN1
IL15
CUX1
CRCP
MTREX
USO1
CASP6
PREPL
AUTS2
CXCL11
BTG1
AAGAB
IRS2
METTL8
TMCC3
CACNG1
TIPARP
DYNC2LI1
RUSF1
SHISAL1
MAPK9
TRMT61B
RFC1
MRPL13
GALNT17
SPIRE2
CNOT6L
KIT
GALM
RFC2
MYO1C
SP2
ART3
ENOX1
PSMD2
MLXIPL
BTC
DHX57
PPBP
CDYL2
SDAD1
THUMPD2
ZNF394
CXCL2
MAP4K3
RAP1B
ITGAM
ST8SIA4
ENSSSCG00000008984
HOOK3
COG3
NFXL1
FCGR3
C4H1orf226
ADD1
SH2D1B
ZNF713
POR
FCGR2B
KCNAB1
SNTB1
ZBTB10
SCFD2
DTX2
C4H8orf76
MTHFD2L
PDE4B
TMEM178A
TNFSF4
RPH3AL
ATAD2
LYAR
LITAF
PPEF2
SLC4A4
VPS53
MAPK10
RMDN2
FRYL
CRADD
CHIC2
LOC106509841
MVP
CLIP2
ABCG8
CCDC158
TPST1
SMURF1
KCTD7
SMC1B
SYN2
NTAQ1
ARHGEF33
ENSSSCG00000007733
SDCCAG8
AREG
SLC6A4
SLC3A1
LOC100620992
STAT5B
PARM1
DEPTOR
RAB39A
MTBP
CDKL2
CPSF4
TXNRD1
SOS1
TRPM3
DLGAP2
INPP5K
PLEKHH2
PPM1B
PHKG2
NWD2
HGSNAT
GLT8D2
ENSSSCG00000005971
TAF2
MAP2K6
PDGFD
ZNF512
SEPTIN14
PHF21B
GAN
KCNB2
HECW1
ABCG5
−log10p

Figure 5.

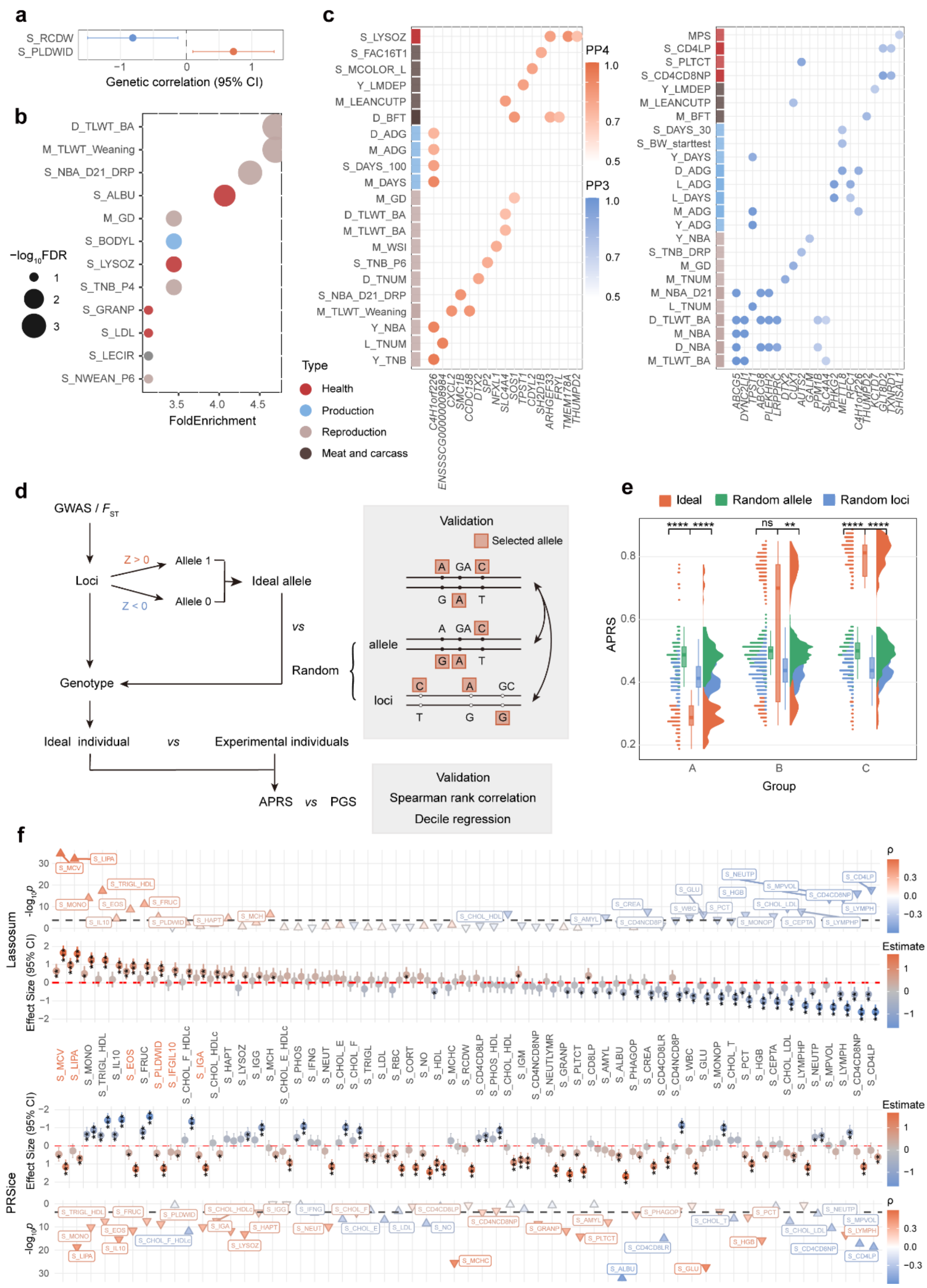
a
S_RCDW
S_PLDWID
Genetic correlation (95% CI)
b
D_TLWT_BA
M_TLWT_Weaning
S_NBA_D21_DRP
S_ALBU
M_GD
S_BODYL
S_LYSOZ
S_TNB_P4
S_GRANP
S_LDL
S_LECIR
S_NWEAN_P6
−log10FDR
FoldEnrichment
c
PP4
PP3
Type
Health
Production
Reproduction
Meat and carcass
d
GWAS / FST
Loci
Z > 0
Z < 0
Allele 1
Allele 0
Ideal allele
vs
Random
allele
loci
Genotype
Ideal individual
Experimental individuals
APRS
PGS
Validation
Selected allele
Validation
Spearman rank correlation
Decile regression
e
Ideal
Random allele
Random loci
APRS
Group
f
Lassosum
PRSice
Effect Size (95% CI)
-log10p
Estimate
ρ

Figure 6.

Figure

Click here to access/download;Figure;Figure 1-abstract.tif

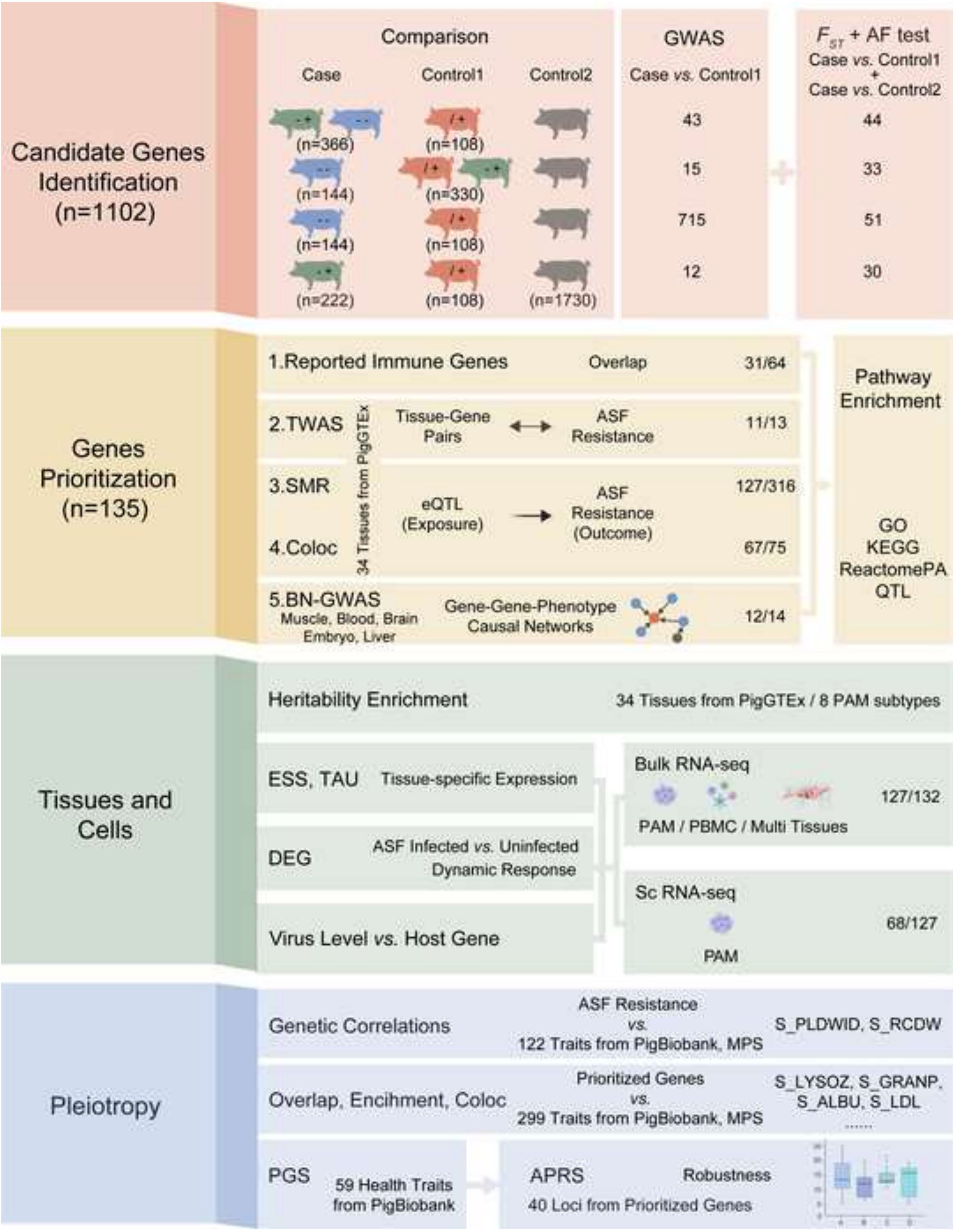

Figure

Click here to access/download;Figure;Figure 2-fuji.tif

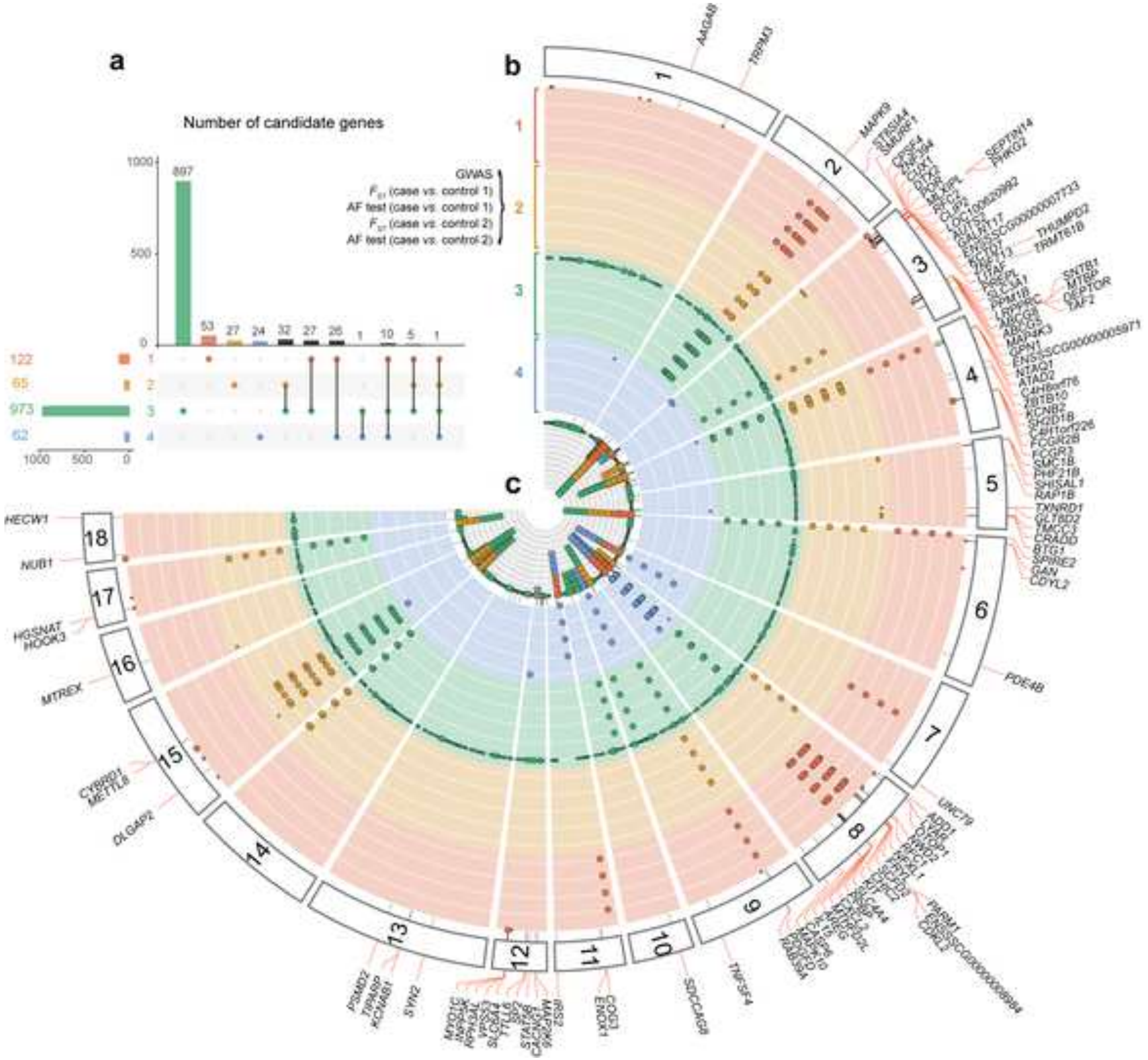

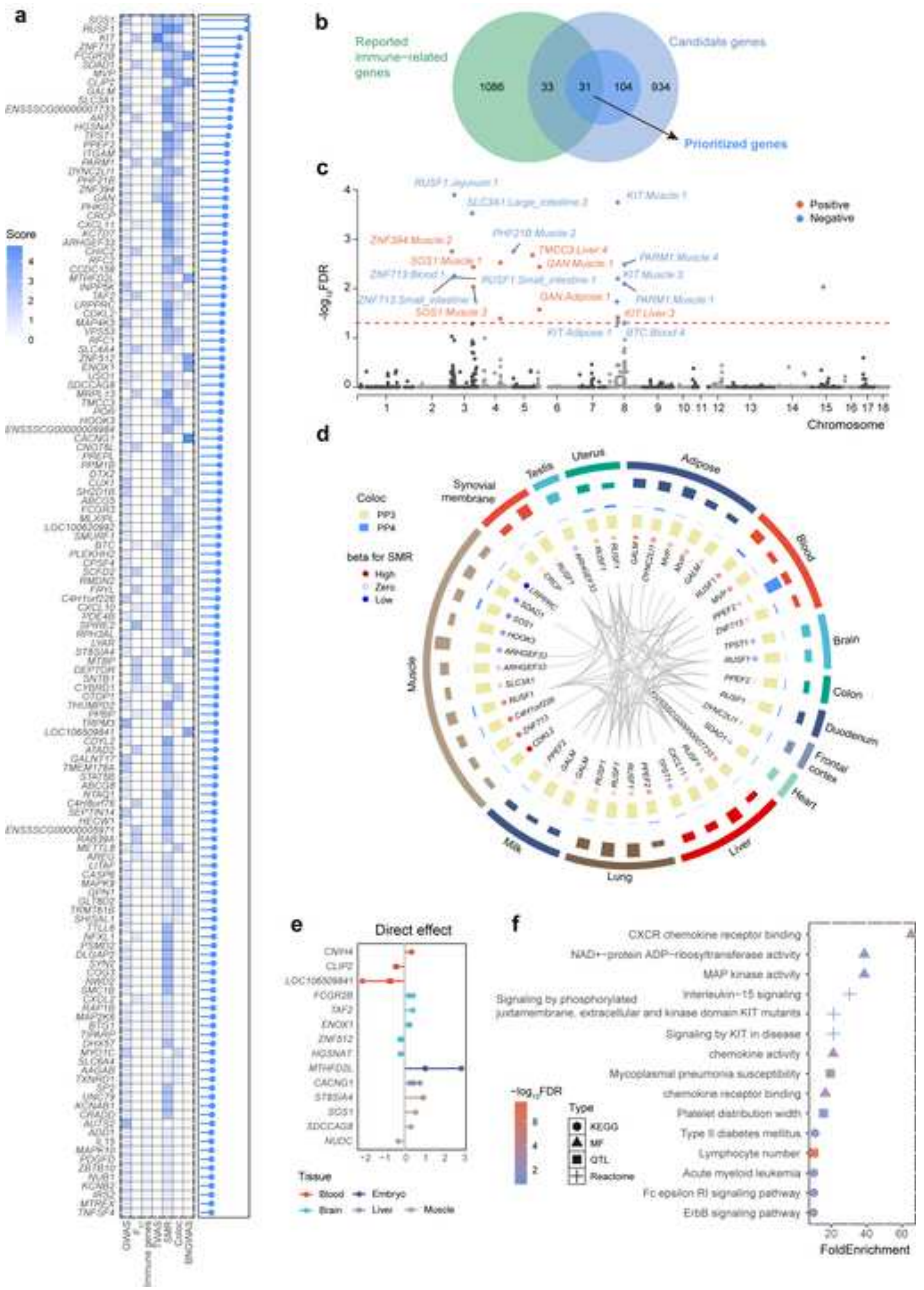
a
Score
b
Reported immune-related genes
Candidate genes
1086
33
31
104
934
Prioritized genes
c
Positive
Negative
Chromosome
d
Coloc
PP3
PP4
beta for SMR
High
Zero
Low
Synovial membrane
Testis
Uterus
Adipose
Blood
Brain
Colon
Duodenum
Frontal cortex
Heart
Liver
Lung
Milk
Muscle
e
Direct effect
Tissue
Blood
Embryo
Brain
Liver
Muscle
f
CXCR chemokine receptor binding
NAD+-protein ADP-ribosyltransferase activity
MAP kinase activity
Interleukin-15 signaling
Signaling by phosphorylated juxtamembrane, extracellular and kinase domain KIT mutants
Signaling by KIT in disease
chemokine activity
Mycoplasmal pneumonia susceptibility
chemokine receptor binding
Platelet distribution width
Type II diabetes mellitus
Lymphocyte number
Acute myeloid leukemia
Fc epsilon RI signaling pathway
ErbB signaling pathway
Type
KEGG
MF
QTL
Reactome
FoldEnrichment

Figure

Click here to access/download;Figure;Figure 4-tissue.tif

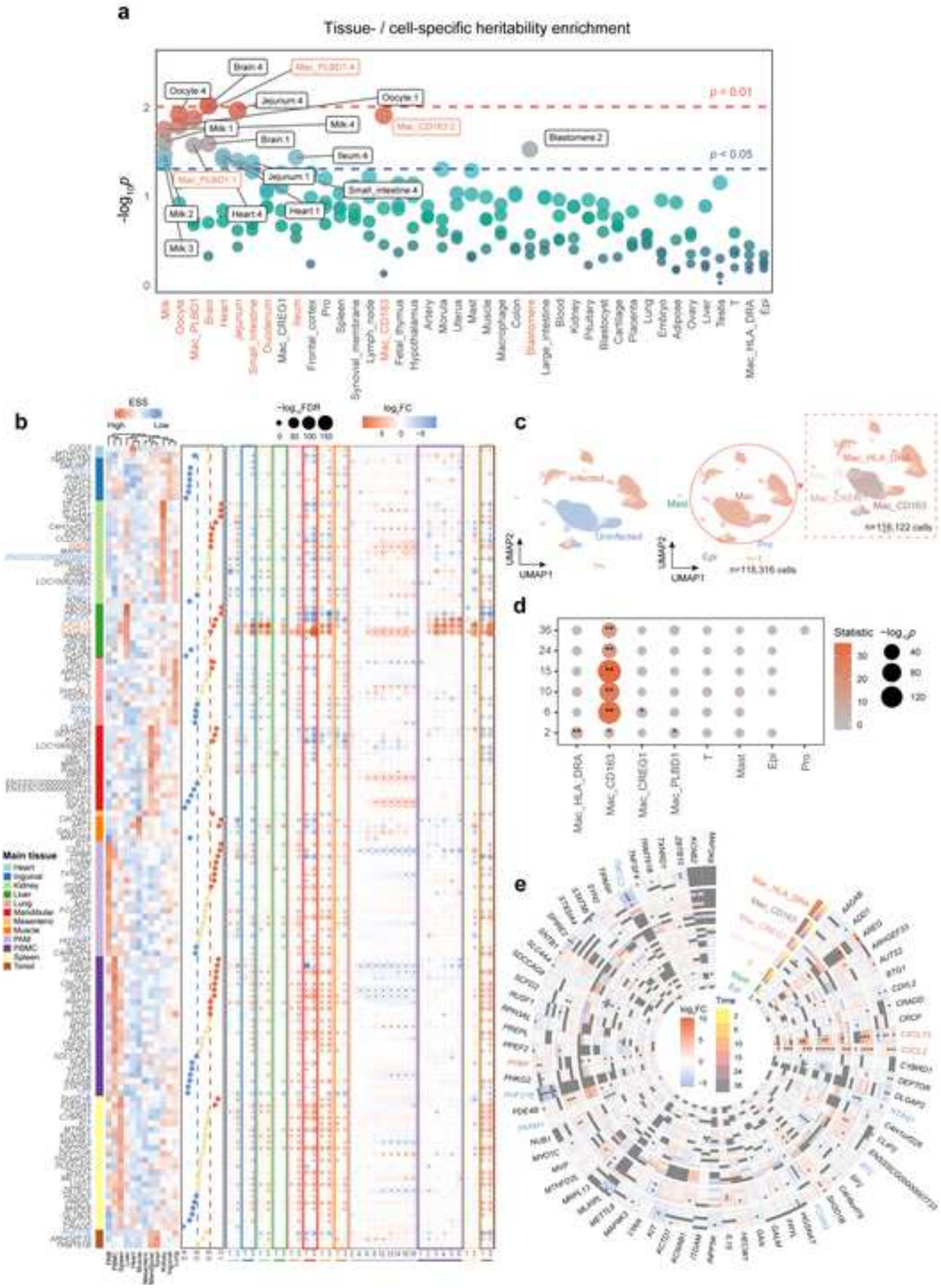

 Click here to access/download;Figure;Figure 5-virus RNA.tif

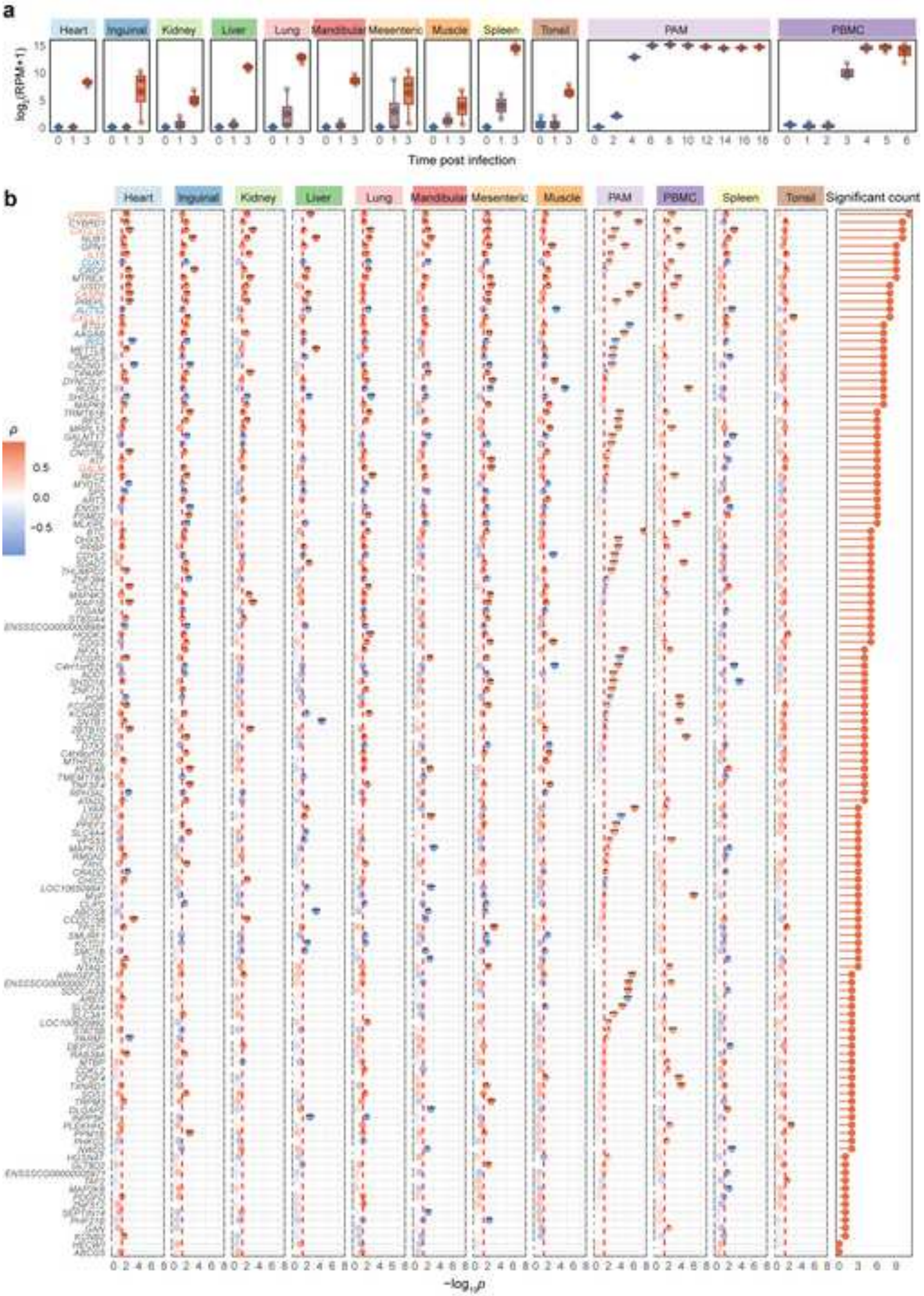

Figure

Click here to access/download;Figure;Figure 6-pleiotropy.tif

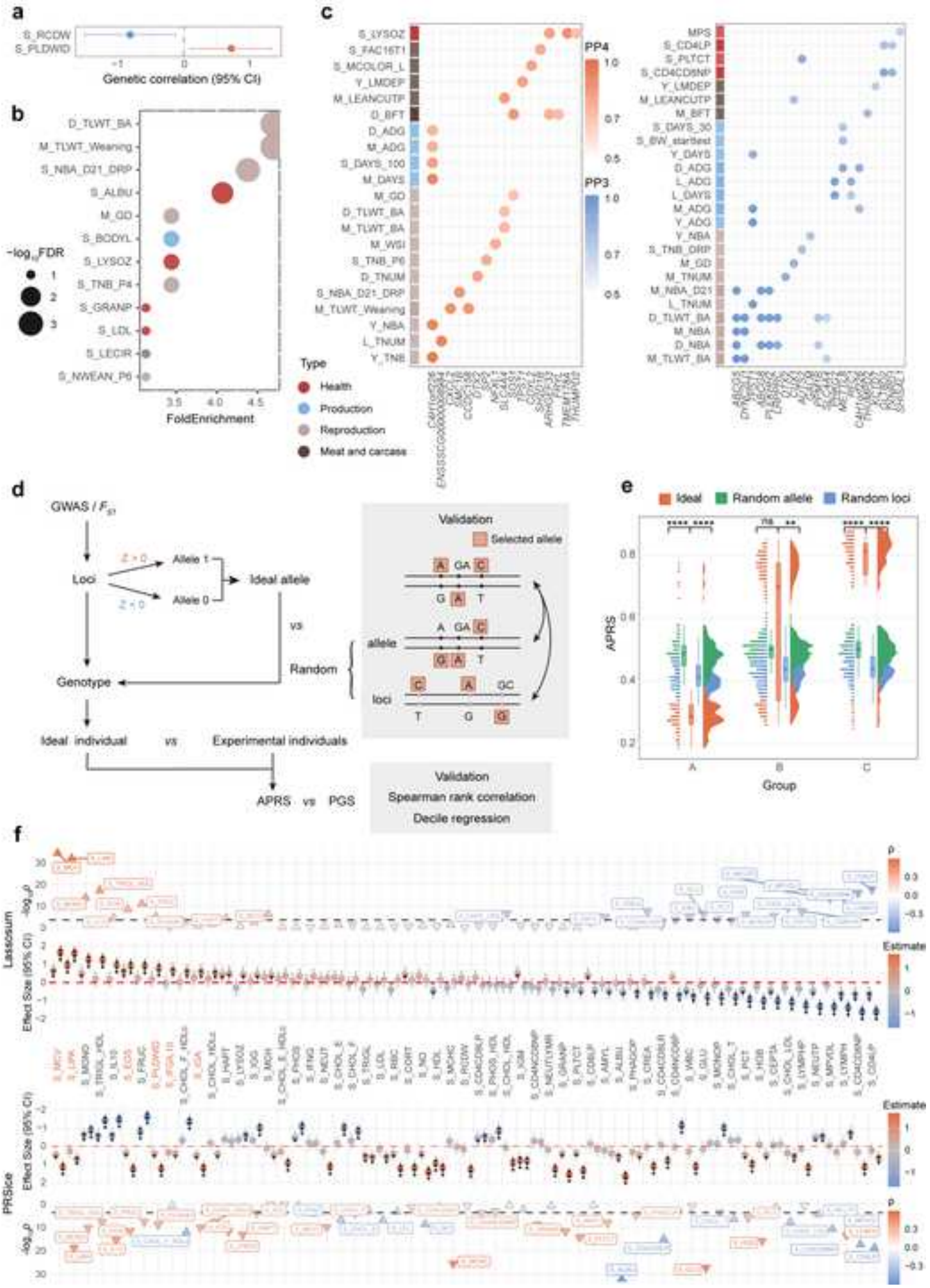

Click here to access/download
Supplementary Material
Supplementary table.xlsx

Click here to access/download
Supplementary Material
Supplementary information-revised.docx
