# Supplementary material for: Integrated multi-omics profiling identifies genetic loci of African swine fever resistance in pigs
Source: Gigascience. 2026 May 30;15:giag066. doi: 10.1093/gigascience/giag066 (PMC13289747; doi:10.1093/gigascience/giag066)
Supplement: giag066_Authors_Response_To_Reviewer_Comments_original_submission [file giag066_authors_response_to_reviewer_comments_original_submission.pdf]

Author's Response To Reviewer Comments

Please refer to the Response file.

Reviewer #1:
Line 70-71: It would be good to clarify that the project losses for the US are hypothetical if ASFV ever arrived in the US (which it has not yet). As written, this is not clear.
Response: Thank you for this critical clarification. We agree that the previous phrasing did not sufficiently distinguish between historical costs and hypothetical projections. We have revised the text to explicitly state that the $50 billion figure for the US is an estimated loss based on a simulated outbreak scenario where the disease persists for ten years and halts exports.
Revision excerpts: In lines 70-74, we changed the following sentence
"…, while projections for 2023 suggested potential losses of 2.5 billion USD in Australia and up to 50 billion USD in the United States." to
"For 2023, projections suggested potential losses of 2,500 million USD in Australia 7 and, in the event of an outbreak, economic modeling estimates potential losses of up to 5,000 million USD in the United States, assuming the disease persists for ten years and leads to a prolonged suspension of exports 8."

Line 101: This is the start of a passage that states "to explore this hypothesis" in reference to the previous paragraph, but there is no hypothesis proposed before this statement. Please clarify the text here to clearly state the objectives of the study.
Response: Thank you for pointing this out. We have rephrased the transition to clearly state the objectives of the study. Instead of referring to a "hypothesis", we now specify that our goal was to systematically identify the genetic basis of resistance traits in indigenous pig breeds.
Revision excerpts: In line 130, we changed the following sentence "To explore this hypothesis, …" to "To systematically identify the genetic basis of these resistance traits, …"

Line 113-116: The text states, "our study bridges variant discovery with functional validation and predictive modeling, offering a more mechanistic understanding of ASF resistance." The authors need to be very careful here as neither functional no mechanistic data is used in this study. Only gene expression and GWAS data are used in the presented analyses. No assays that directly address viral or host immune function or gene function through knockout, enzymatic or true functional assays are used in this study. I understand what the authors are trying to say, but the analyses conducted are not functional or mechanistic, but instead based on functional enrichment or integrative, high-throughput omics analyses which is not the same as the targeted molecular biology assays implied by the text. Please update the language throughout to differentiate between causal statistical inference and candidate causal mutations.
Response: We sincerely thank the reviewer for this critical distinction. As suggested, we have thoroughly revised the manuscript to avoid the terms "functional validation" and "mechanistic understanding" when referring to our bioinformatic predictions. We have replaced these with more accurate terms such as "functional enrichment", "integrative prioritization", and "multi-dimentional inference". We have also clarified the distinction between candidate causal mutations and statistically inferred signals throughout the text.
Revision excerpts: In lines 143-144, we changed the following sentence
"…, our study bridges variant discovery with functional validation and predictive modeling, offering a more mechanistic understanding of ASF resistance." to
"…, our study bridges variant discovery with functional analyses and predictive modeling, offering more biological insights of ASF resistance."
We added "potential" before every instance of "causal" (e.g. lines 214, 223).
We changed "validation" to "inference/permutation/evaluation" (e.g. line 184).
We changed "validate" to "support/substantiate" (e.g. line 213).
We changed "mechanism" to "basis/signature/pathway" (e.g. line 342).
…

Line 151-172: Regarding the TWAS and SMR analysis, are these two methods truly independent analyses? They both appear to rely on the same pigGTEx gene expression data. Does this mean they destined to

generate similar results because similar input information is used in these analyses? Or are they actually using different datasets? Please explain how these analyses compliment each other and provide unique (and not common) information in their results.
Response: Thank you for this insightful question regarding the relationship between TWAS and SMR. We agree that both methods utilize the PigGTEx expression data and our GWAS summary statistics. However, they are methodologically distinct and complementary rather than redundant, providing different layers of genetic evidence:
Different statistical principles: TWAS aggregates the effects of multiple SNPs into a single gene-level test. It aims to identify associations between the predicted expression levels of a gene and the resistance trait, which enhances power to detect genes with multiple weak eQTLs 1. SMR focuses on identifying whether the top eQTL and the top GWAS SNP at a locus share the same underlying causal variant using a Mendelian Randomization framework 2.
Complementary insights (association vs. inferred mediation): TWAS is an association-based tool that captures the overall genetic correlation at the gene level 1. SMR, when combined with HEIDI/Colocalization tests, is specifically designed to distinguish pleiotropy (a single variant affecting both gene expression and resistance trait) from linkage (different variants in LD) 2. TWAS cannot easily distinguish between these two scenarios.
Unique information: In our study, TWAS helped us identify genes where the cumulative effect of cis-variants is significant, even if no single SNP reaches genome-wide significance. SMR and colocalization provided a more stringent filter to prioritize “high-confidence” candidate genes (like PPEF2) where the evidence suggests a potential shared causal signature.
In summary, TWAS provides a broader screen for gene-trait associations, while SMR/colocalization provides a locus-specific refinement to prioritize candidate genes with evidence of shared genetic control. We have added a brief clarification in the Results and Methods section to explain how these two approaches complement each other.
Revision excerpts:
Result:
In lines 192-194, we added the following sentence
“To prioritize candidate genes based on the aggregate association between predicted expression levels and ASF resistance, we first performed TWAS across 34 tissues 31 (Supplementary Table S6).”
In lines 209-210, we added the following sentence
“In parallel, to identify high-confidence genes by testing the potential mediation effects of specific top eQTLs, we employed SMR and colocalization analysis.”
Method:
In lines 834-839, we changed the following sentence
“To evaluate the association between our candidate genes and ASF across various tissue types (including 34 tissues from PigGTEx, Supplementary Table S6) and to elucidate their functional roles, we conducted transcriptome-wide association studies (TWAS) analysis using the online tool FarmGTEx TWAS-server (https://twas.farmgtex.org/). Genomic loci demonstrating significant associations were identified by retaining those with a false discovery rate (FDR) < 0.05.” to
“To prioritize candidate genes by aggregating the cumulative effects of multiple cis-variants on predicted expression levels, we conducted TWAS analysis. Using the FarmGTEx TWAS-server 134 (https://twas.farmgtex.org/), we evaluated associations between candidate genes and ASF resistance across 34 tissues from PigGTEx 31 (Supplementary Table S6). Significant associations were identified using a false discovery rate (FDR) < 0.05.”
In lines 842-843, we added the following sentence
“To identify potential regulatory links by testing the mediation effect of top eQTL signals on ASF resistance, we employed the SMR framework.”
In lines 849-850, we added the following sentence
“To further refine prioritized genes by distinguishing shared genetic control from mere genomic linkage, we performed colocalization analysis.”

Line 152: What does “prioritized genes” mean in this case? It looks like these are the top genes based on FDR significance from the TWAS. How are the genes identified based on muscle and liver gene expression justified in the context of ASFV? What biological roles do these tissues play in ASFV infection or how do they change in ASFV that would provide signal for incidence of ASF? Please update the text accordingly.
Response: Thank you for these insightful comments. We have addressed the concerns regarding the

definition of “prioritized genes” and the biological relevance of liver and muscle:
We have clarified in the revised text that “prioritized genes” refers to the 135 high-priority candidates identified through our integrated prioritization framework (integrating TWAS, SMR, Coloc, etc.). The 11 genes mentioned in the TWAS section are those from this prioritized set that also reached significance in the TWAS analysis. We have updated the terminology to ensure consistency.
Regarding the biological justification for liver and muscle signals, we have updated the text to reflect the known pathogenesis of ASFV. ASFV predominantly infects macrophages and monocytes, but also targets secondary cells such as vascular endothelial cells, hepatocytes, and epithelial cells 3. The liver serves as a core replication hub (targeting Kupffer cells and hepatocytes) 4, while muscle signals likely reflect the physiological response of secondary target cells (e.g., microvascular endothelium 5) and the infiltration of primary target cells during systemic infection. This biological context justifies why gene expression variations in these tissues are statistically linked to the ASF resistance phenotype.
We have added these biological justifications to the Results section to better contextualize our findings.
Revision excerpts: In line 182, we added the “(hereafter referred to as “prioritized genes”)” after “high-priority candidate genes”.
In lines 194-195, we changed the following sentence “This analysis revealed significant tissue-specific associations for 11 prioritized genes across muscle, liver, blood, and intestinal tissues” to
“This analysis revealed that 11 of the 135 prioritized genes exhibited significant tissue-specific associations.”
In line 197-203, we added the following sentence “… (Fig. 3c, Supplementary Fig. S3 and Supplementary Table S7). The liver is a primary site of viral replication, involving both resident Kupffer cells and hepatocytes 36, whereas signals in muscle likely reflect the systemic nature of infection, capturing the responses of secondary target cells, such as vascular endothelium 37, alongside the infiltration of primary target cells like monocytes and macrophages 38. These findings align with the systemic pathology of ASFV, where genetic variation in both primary and secondary target tissues modulates overall host resistance. ...”

Line 164: The text states, “confirming the presence of shared causal variants associated with ASF resistance (Fig. 3d).” I can appreciate that causal statistical modeling is used within the analyses of this manuscript. However, it is very important to note there is not evidence presented to determine that these are causal genes. Causal genes and causal variants can only be identified through specific functional molecular biology assays linking specific gene functions to trait phenotypes (e.g., knockouts, gene editing, enzymatic assays, RNAi, etc. that alter sub processes of trait phenotypes or the trait phenotype itself). No such biological evidence is presented so the identified variants cannot be called causal variants. What is presented are enrichment or conditioned statistical tests to provide a probability that a gene may be a causal variant (i.e., to identify candidate causal variants). Please update the text where appropriate throughout the manuscript.
Response: We sincerely thank the reviewer for this critical comment regarding the use of the term “causal”. We completely agree that while colocalization analysis provides strong statistical support for shared genetic signals, definitive identification of causal genes or variants requires direct functional validation, which is beyond the scope of the current statistical study. In accordance with your suggestion, we have thoroughly revised the manuscript to ensure a more precise use of terminology.
Revision excerpts: In line 213-214, we changed the following sentence
“Colocalization analysis further validated 42 of these pairs, confirming the presence of shared causal variants associated with ASF resistance.” to
“Colocalization analysis further supported 42 of these pairs, suggesting the presence of shared potential causal variants associated with ASF resistance (Fig. 3d).”

In lines 178, 237, and 241, we changed “validation methods” to “lines of evidence”.
In line 182, we added “candidate” to “high-priority candidate genes”.
In line 208, we changed “Causal relationships identification via SMR and colocalization” to “Inference of putative regulatory genes via SMR and colocalization”.
In line 224, we changed “Causal effects of ASF-resistance gene validating via BN-GWAS analysis” to “Regulatory network inference via BN-GWAS”.
In line 231, we changed “direct or indirect causal” to “putative regulatory”.
In line 235, we changed “validation” to “cross-evaluation”.
In line 445, we changed “functional validation” to “functional annotation”.
In line 462, we changed “causally influence” to “potentially mediate”.

Line 175: Please explain in the context of ASFV how muscle, brain, embryo and liver gene expression data is relevant. We know from previous studies that immune tissues are relevant and that CD163 in cell types such as macrophage are important in ASFV, but do we know similar information for the other tissues?
Response: We appreciate the reviewer's comment regarding the tissue selection for BN-GWAS. Our choice was guided by two primary considerations:
Statistical reliability: Bayesian Network (BN) inference is highly sensitive to sample size 6. To avoid false-positive regulatory links and ensure model stability, we restricted our analysis to tissues in the PigGTEx database with sample size > 300. Other immune-related tissues with smaller cohorts were excluded to maintain the rigor of the gene prioritization framework.
Systemic Nature of ASFV: While macrophages are the primary targets, ASFV infection is a systemic disease characterized by a cytokine storm, vascular damage, and multi-organ failure 7,8. Liver and blood: As the reviewer noted, these are primary sites for viral replication and immune response 4. Brain and embryo: ASFV can cross the blood-brain barrier and the placental barrier, leading to neurological symptoms and abortion, which are both hallmark clinical signs of acute ASF 7,8. Muscle: As a highly vascularized tissue, it serves as a sentinel for systemic inflammation and microvascular integrity 5.
By using high-quality data from these diverse tissues, we aimed to capture the broad regulatory landscape of the host's response. We have updated the text to clarify this rationale.
Revision excerpts:
In lines 226-231, we changed "Using RNA-seq data from five tissues (muscle, blood, brain, embryo, and liver) with sample sizes exceeding 300, …" to
"To ensure robust causal inference, we utilized RNA-seq data from five tissues (muscle, blood, brain, embryo, and liver) with sample sizes exceeding 300, as sufficient power is critical for stable expression imputation. These tissues represent both primary viral replication sites (liver and blood) and organs that reflect the systemic physiological disruptions caused by ASFV 42,43."

Line 187: Please list the 3 specific validation methods here to help the reader as there is so much information presented, it's hard to keep the different analyses used clear.
Response: We appreciate the reviewer's suggestion to clarify the specific methods for these 13 genes. Because these genes were prioritized based on different combinations of the five analytical lines (including reported immune genes, TWAS, SMR, colocalization, and BN-GWAS), listing every unique combination in the text would be cumbersome for the reader. To address this, we have:
Updated the text to clarify that these genes are supported by varying combinations of three evidence lines.
Added a reference to Supplementary Table S4 in line 243 to explicitly check off which specific methods support each gene. This allows the reader to easily track the exact evidence for any gene of interest at a glance.
Revision excerpts: In line 241-243, we changed the following sentence
"… were supported by three validation methods, highlighting their robustness (Fig. 3a)." to
"… were supported by three independent lines of evidence, such as combinations of TWAS, SMR, colocalization, highlighting the robustness of their prioritization (Fig. 3a and Supplementary Table S4)."

Line 198-201: The text states, "Additional, signaling by KIT in disease (FDR) and lymphocyte number (FDR) emerged as key pathways potentially driving immune responses against ASF. These findings reinforce the involvement of specific immune signaling mechanisms in ASF resistance." I'm not sure what you are trying to say in the second sentence here. If the intent is to say this is a new signaling pathway identified, please make this more clear in the text. If the intent is to say this shows that immune functions are enriched and this is an example, then I recommend deleting this sentence as that is obvious from the result.
Response: We appreciate the reviewer's comment. Our intent was not to state the obvious fact that immune functions are enriched, but rather to highlight specific regulatory signatures that are critically relevant to ASFV pathology. We have revised the text to collectively present these key pathways, emphasizing their strong alignment with established ASFV-induced pathologies. Specifically, the enrichment of pathways like CXCR/MAPK signaling and lymphocyte number directly reflects the host's attempt to regulate the inflammatory response and counteract the profound lymphopenia characteristic of acute ASF. This consolidation clarifies how our prioritized genes are biologically relevant to the known infection mechanisms of the virus.
Revision excerpts: In lines 249-256, we changed the following sentence

“Notably, pathways such as CXCR chemokine receptor binding (FDR = 1.7×10-3), MAP kinase activity (FDR = 2.3×10-2), and interleukin-15 signaling (FDR = 3.2×10-2) were among the most significantly enriched. These pathways, previously implicated immune gene prioritization, underscore the critical roles of chemokine signaling, MAP kinases, and IL-15 in mediating ASF resistance. Additionally, signaling by KIT in disease (FDR = 4.9×10-2) and lymphocyte number (FDR= 7.7×10-15) emerged as key pathways potentially driving immune responses against ASF. These findings reinforce the involvement of specific immune signaling mechanisms in ASF resistance.”to

“Notably, the most enriched functional clusters, including CXCR chemokine receptor binding (FDR = 1.7×10-3), MAP kinase activity (FDR = 2.3×10-2), interleukin-15 signaling (FDR = 3.2×10-2), and lymphocyte number (FDR= 7.7×10-15), exhibit high concordance with the characterized pathological features of ASFV infection, particularly regarding the systemic inflammatory cascades and the profound lymphopenia that define acute ASF 46–48. By pinpointing these pathways, our results underscore the critical role of maintaining immune homeostasis and cellular signaling integrity in mediating host resistance to ASFV.”

Line 211-213: This text discusses potential cross-tissue resistance networks. Is this biologically founded? Correlations can be founded across many types of data despite a lack of causal relationships. Are there other examples of this in other biological processes? For instance, are there extracellular signaling molecules identified that would make sense as cross-tissue signaling molecules? Alternatively, maybe this passage can be worded in a slightly different way to highlight this may be worth future investigation. The best place to address this may be at lines 433-435 in the discussion.

Response: We thank the reviewer for this insightful comment. ASFV infection is a systemic disease characterized by a “cytokine storm” and multi-organ dysfunction 7, providing a biological basis for our findings.

The pathology of ASF is widespread: the brain is affected by neuro-inflammation (leading to apathy and depression), the heart by epicardial hemorrhage, and the intestines by severe mucosal damage7,9. We identified several prioritized genes, such as CXCL10 and IL15, which encode circulating cytokines/chemokines. These molecules act as systemic signals that coordinate the immune response across tissues, regardless of local viral replication levels 10,11. We believe these findings provide a valuable starting point for understanding the systemic nature of ASF.

We have revised the manuscript to frame the “cross-tissue resistance network” as a hypothesis where genetic variation modulates systemic homeostasis and inter-organ communication to ASF resistance.

Revision excerpts:

Result: In lines 260-266, we changed the following sentence

“Significant heritability enrichment was observed in PAM subtypes (Mac_CD163 and Mac_PLBD1) and the small intestine (including jejunum and ileum) (Fig. 4a and Supplementary Table S12). As the primary target of ASF, PAMs play a central role in infection 12, while the intestine is vital for mucosal immunity and host-microbiota interactions that may influence viral attachment and invasion 13. These results align with established ASF infection mechanisms 7,12. Interestingly, enrichment in non-traditional tissues such as the brain, heart, and milk suggested a potential cross-tissue resistance network, highlighting broader systemic contributions to ASF resistance beyond primary immune tissues.” to

“Significant heritability enrichment was observed in PAM subtypes (Mac_CD163 and Mac_PLBD1) (Fig. 4a and Supplementary Table S12), consistent with the established role of PAMs as the primary targets of ASFV infection 49. Interestingly, heritability enrichment was also detected in non-immune tissues, including the small intestine, brain, heart, and milk. This suggests that genetic contributions to ASF resistance may extend beyond primary immune sites, potentially reflecting a systemic, cross-tissue defensive architecture.”

Discussion: In lines 496-518, we changed the following sentence

“ASFV infection manifests as a severe systemic disease, marked by cytokine storm and extensive hemorrhages and lesions across multiple organs, such as apathy, anorexia, depression (brain), epicardium hemorrhage, hydropericardium (heart), diarrhea (intestinal), orchitis, and abortion (reproductive organs)14. Our time-course transcriptomic data confirm a widespread host response, with the majority of prioritized genes (127/132) exhibiting dynamic expression changes post-infection. While these responses were most prominent in lymphoid tissues, such as mandibular lymph nodes, tonsils, and mesenteric lymph nodes, underscoring the central role of immune organs, viral quantification revealed a distinct spatial pattern. Specifically, tissues not primarily linked to immune functions harbored substantially lower ASFV RNA levels compared to primary targets like the spleen and PAMs. Consequently, the elevated expression

of immune-related genes in these non-target tissues likely reflects systemic inflammatory signaling rather than significant local viral replication, for example, several prioritized genes (e.g., CXCL10, IL15) encode secreted proteins that could potentially serve as mediators for such cross-tissue communication. The detection of low-level virus in these regions is plausibly due to secondary seeding via viremia as the infection progresses. The heritability enrichment in non-immune organs like the intestines, brain, and heart suggests the existence of a cross-tissue resistance network that extends beyond canonical immune sites. The intestine is vital for mucosal immunity and host-microbiota interactions that may influence viral attachment and invasion 13. Notably, approximately 27% of the prioritized genes (36/132), including ADD1, ZNF family members, and RUSF1, were broadly expressed across tissues, providing molecular support for a systemic and interconnected host defense mechanism." to

"ASFV infection triggers a severe systemic crisis, characterized by a cytokine storm and multi-organ hemorrhaging 42,43,92. Clinical manifestations such as depression (brain), hydropericardium (heart), and diarrhea (intestines) underscore the widespread nature of the disease 42,43,92. Our time-course transcriptomic data supports this systemic involvement, with the majority (127/132) of prioritized genes exhibiting dynamic expression changes post-infection. While these responses were most robust in lymphoid tissues, viral quantification revealed that non-canonical tissues harbored substantially lower ASFV RNA levels compared to primary targets like the spleen or PAMs. Consequently, the gene expression changes in these non-target tissues likely reflect physiological responses to systemic inflammatory signaling rather than local viral replication. This is supported by the identification of several prioritized genes such as CXCL10 and IL15, which encode secreted proteins that can act as systemic mediators of inter-organ communication 35,93. The presence of low-level viral RNA in these tissues is likely a secondary effect of viremia. Crucially, the significant heritability enrichment observed in non-immune organs, including the intestines, brain, and heart, provides genetic support for a resistance network that extends beyond canonical immune sites. Furthermore, approximately 27% (36/132) of the prioritized genes (e.g., ADD1, ZNF family members, and RUSF1) were broadly expressed across all examined tissues. Together, these findings suggest that ASF resistance is not solely a localized immune event but involves a multi-organ genetic architecture that maintains systemic integrity. Such a cross-tissue network, potentially involving mucosal immunity in the intestine 94 or neuro-endocrine-immune crosstalk 95, warrants further functional investigation to fully elucidate the basis of host resistance."

Line 249-252: What is the biological significance of these relationships between the viral gene expression and the 4 host transcripts listed? Please provide more explanation here of the context or meaning of the result. Is this meant to indicate these genes may play a role in host response?

Response: We appreciate the reviewer's request for a clearer explanation of the biological context of these results. Our analysis was designed to bridge the gap between genetic prioritization and biological function through a three-step logic:

Genetic priority & resistance: These genes were first identified as prioritized candidates influencing ASF resistance based on aggregate genetic evidence (TWAS / SMR / colocalization / BN-GWAS).

Infection response: Their significant differential expression across multiple tissues confirms their active role in responding to ASFV, rather than being mere bystanders.

Viral coupling & biological context: The correlation with viral transcript abundance provides a "abundance-dependent response" dimension to their function: Positive Correlations: These genes (e.g., CXCL10, IL15, CASP6) represent the escalation of the host's defensive and inflammatory signaling. As viral replication intensifies, the host ramps up these pathways, which often leads to a pathological cytokine storm and extensive cell death in the case of ASFV. Negative Correlations: These genes (e.g., IRS2, RUSF1) represent the progressive depletion of essential host functions. Their decline in direct proportion to viral increase reflects the systemic metabolic collapse and functional deprivation caused by the virus. On "contradictory" signals (e.g., autophagy/metabolism): The presence of both positive and negative correlations within the same functional category reflects the biological tug-of-war between host-mediated clearance and viral-driven subversion.

By demonstrating that these genes' expression levels are precisely synchronized with viral transcript abundance, we provide strong evidence that they are core components of the host-pathogen interface and key drivers of the resistance phenotypes observed. We have updated the manuscript to incorporate these biological insights.

Revision excerpts: In lines 298-308, we changed the following sentence

"To explore host-pathogen interactions, a Spearman correlation was performed, identifying positive associations between viral RPM and the transcriptional expression (TPM) of GALM, CXCL10, IL15, and

CXCL11 in several tissues (Fig. 5b).” to
“To further characterize host–pathogen interactions, we performed Spearman correlation analysis between viral transcript abundance (RPM) and host gene expression (TPM). A subset of consistently upregulated genes, including those involved in host defense and immune signaling (e.g., LRPPRC 52, CXCL10, CXCL11 53, CASP6 54, IL15 35), showed strong positive correlations with viral RPM (Fig. 5b). In contrast, consistently downregulated genes involved in critical host functions, such as transcriptional regulation (CUX1 55) and metabolic homeo stasis (IRS2 56), exhibited significant negative correlations (Fig. 5b). This coordinated, abundance-depe ndent response indicates that these prioritized genes are not merely reactive but are likely integral to ASFV-driven biological processes, representing key determinants of host resistance and disease severity.”

Line 258: Which “prioritized genes” are referred to here? There are so many lists of prioritized genes, it is hard to keep track of them all.
Response: We appreciate the reviewer’s comment regarding the clarity of our gene lists. To avoid confusion, we have clarified that the “prioritized genes” analyzed in the single-cell data refer to the final consensus set of 135 high-confidence candidates identified by integrating multiple genomic lines of evidence (e.g., TWAS, SMR, colocalizatin, and BN-GWAS), as defined in line 182.
Due to the inherent sparsity of scRNA-seq data, 127 out of these 135 genes passed our quality control and stable expression filters. We have updated the text and the legend of Fig. 4e to explicitly state that this analysis focuses on this 127-gene prioritized subset. This ensures consistency between our integrated genetic findings and the single-cell validation. We have also provided the full list of these 127 genes in Supplementary Table S17 for better tracking.
Revision excerpts:
Result: In lines 310-316, we changed the following sentence
“Single-cell RNA-seq analysis in PAMs (target cells for ASFV) identified 127 prioritized genes in various celltypes post-infection. The Mac_CD163 cells, a transcriptionally distinct PAM subpopulation characterized by high baseline expression of CD163, MARCO, S100A8, and S100A9, exhibited sustained upregulation of the prioritized genes throughout ASFV infection (Fig. 4e and Supplementary Table S16).” to
“Single-cell RNA-seq analysis of PAMs 49 (target cells for ASFV) identified 127 prioritized genes from a subset of 135 total prioritized genes, filtered for stable expression in the scRNA-seq dataset (Supplementary Tables S1 and S17). Among various celltypes post-infection, the Mac_CD163 cells, a transcriptionally distinct PAM subpopulation characterized by high baseline expression of CD163, MARCO, S100A8, and S100A9 49, exhibited sustained upregulation of 127-gene prioritized module throughout ASFV infection course (Fig. 4e and Supplementary Table S16).”
Figure legend: In lines 1612-1614, we changed the following sentence
“Scoring of prioritized gene sets pre- and post-infection in PAM scRNA-seq. …” to
“Module scoring of the prioritized gene subset in PAM scRNA-seq. The score represents the aggregate expression difference of 127 prioritized genes (a detectable subset of the 135 total prioritized genes) pre- and post-infection. …”

Line 254-274: Are these results novel or consistent with past studies? I apologize if I’ve missed commentary on this aspect of the identified results in the discussion.
Response: We thank the reviewer for the opportunity to clarify the novelty of our single-cell analysis. While we utilized a published scRNA-seq dataset 12 and adopted its established cell-type annotations, our findings provide novel insights distinct from the original study:
New genetic context: The original study focused on a general transcriptomic overview of ASFV infection. In contrast, our analysis overlayed a genetically-prioritized resistance gene set (135 genes) onto this cellular framework to identify specific “resistance modules”.
Novel candidates: We identified 127 prioritized genes as key cellular responders, highlighting candidates like CXCL2, CXCL10, TMCC3, and PHF21B that were not previously emphasized as resistance-related in the original publication.
Biological link: Our analysis bridges the gap between systemic genetic heritability and single-cell behavior, showing that the resistance alleles identified in our study are specifically active in the Mac_CD163 subpopulation.
We have refined the Results and Discussion sections to more clearly articulate how our targeted analysis of prioritized genes builds upon and extends the existing cellular knowledge of ASF.
Revision excerpts:

Result: In lines 321-323, we changed the following sentence
"Differential expression analysis further revealed that 68 prioritized genes were significantly differentially expressed in at least one cell type at specific time point." to
"Our differential expression analysis specifically focused on this gene set further revealed that 68 prioritized genes were significantly modulated in at least one cell type at specific time points ..."
In lines 331-333, we changed the following sentence "…, potentially reflecting virus-induced suppression mechanisms targeting host immune pathways." to
"…, potentially reflecting virus-induced suppression signatures targeting these genetically prioritized host pathways."
Discussion: In lines 521-537, we changed the following sentence
"Consistently, our heritability enrichment analysis identified Mac_CD163 and Mac_PLBD1 subtypes as critical mediators of resistance mechanisms. Consistent with Zheng et al.'s findings, the Mac_CD163 subpopulation is pivotal in the early host defense against ASFV, characterized by a massive transcriptomic response and dynamic population shifts. Despite an initial sharp decline in prevalence post-infection, this subset demonstrated a remarkable ability to restrict viral replication, with a consistently low proportion of cells carrying a high viral load. This coordinated response, featuring profound DEG enrichment and effective viral control, establishes Mac_CD163 cells as a key mediator of innate antiviral immunity. Single-cell transcriptomic profiling revealed that robust and sustained activation of Mac_CD163 in response to ASFV infection, with CXCL2 and CXCL10 genes showing continuous upregulation in these cells, reinforcing their roles as key innate immune effectors. Basal expression analysis also identified genes such as CXCL2, CXCL7, and BTC as exhibiting high specificity to PAM, which are critical sites for viral entry and replication. Collectively, these findings emphasize the interplay between tissue-specific and systemic immune responses in driving ASFV resistance." to
"Our study extends this understanding by demonstrating that the heritability of ASF resistance is specifically enriched in macrophage subtypes, particularly Mac_CD163 and Mac_PLBD1. Consistent with Zheng et al.'s cellular landscape 49, we found the Mac_CD163 subpopulation to be pivotal in the host response. Despite their initial sharp decline in prevalence post-infection, this subset demonstrated a remarkable ability to restrict viral replication, characterized by a low proportion of high-viral-load cells 49. Our analysis adds a critical layer to this observation: the robust antiviral capacity of Mac_CD163 cells is coupled with the sustained activation of our prioritized resistance genes. Specifically, the continuous upregulation of CXCL2 and CXCL10 within this subset reinforces their role as key innate immune effectors 97,98. Furthermore, our basal expression analysis identified genes like CXCL2, CXCL7, and BTC as highly specific to PAMs, which are critical sites for ASFV entry 49. By linking these specific genetic candidates to established cellular responders, we show that Mac_CD163 cells are not just transcriptomic markers of infection, but are the primary cellular vehicles through which these prioritized resistance factors operate. Collectively, these findings emphasize the interplay between tissue-specific genetic architecture and systemic immune responses in driving ASFV resistance."

Line 296: The pleiotropy analysis is interesting, but how comparable is MPS to ASFV infection? There needs to be some discussion of this somewhere in the manuscript. As written, it's unclear if MPS is a relevant trait (thought it would seem it should be).
Response: We appreciate the reviewer's attention to the relationship between MPS and ASF resistance. While both diseases affect the porcine respiratory system, our analysis suggests that their shared genetic basis is highly localized and likely represents linkage rather than pleiotropy.
At the SHISAL1 locus, we observed a high PP3 (0.7153) but a near-zero PP4 (0.0085). This statistical pattern confirms that while both traits have significant signals in this region, they are driven by distinct potential causal variants. Our search of the SHISAL1 functional literature indicates its role in modulating Wnt and FGF signaling pathways, primarily during embryogenesis and head formation 15. There is currently limited evidence linking SHISAL1 directly to broad-spectrum anti-pathogen immunity.
The overlapping signals likely reflect the presence of multiple, independent regulatory elements within this genomic "hotspot" that separately influence lung-related phenotypes. Therefore, despite the superficial similarity between MPS and ASF as respiratory challenges, our results suggest their genetic architectures at this locus are largely independent. We have clarified this distinction in the revised Results and Discussion.
Revision excerpts:
Result: In lines 369-372, we changed the following sentence
"Conversely, distinct causal variants (PP3 > 0.7) were identified for traits like CD4+ leukocyte (GLT8D2, TXNRD1), platelet counts (AUST2), and MPS traits (SHISAL1), highlighting the complex genetic interactions

underlying ASF resistance (Fig. 6c).” to
“Conversely, distinct potential causal variants (PP3 > 0.7) were identified for traits like CD4+ leukocyte (GLT8D2, TXNRD1), platelet counts (AUST2), and MPS traits (SHISAL1), indicating genetic linkage, highlighting the locus-specific complexity of the genetic architecture of ASF resistance (Fig. 6c).”
Discussion: In lines 559-570, we added the following sentence
“The overlapping signals for ASF resistance and MPS at the SHISAL1 locus provide insight into multi-trait genetic architecture. Despite both diseases involving respiratory pathology 42,57,104, colocalization analysis strongly supports the PP3 model (PP3 = 0.7153, PP4 = 0.0085), indicating that the associations are driven by distinct potential causal variants in close proximity. This suggests that the SHISAL1 region represents a multi-trait locus with independent regulatory elements modulating responses to different stressors (bacterial for MPS and viral for ASF), rather than a shared genetic signature. Consistent with this, SHISAL1 is primarily implicated in Wnt and FGF signaling during development, rather than canonical immune pathways 105. Together, these findings indicate that the observed overlap reflects local linkage within a complex regulatory landscape rather than true pleiotropy.”

Line 315-318: Are these novel findings, or is there past evidence that ASF impacts these traits? (Realizing, you may never know for a trait like meat quality).
Response: We appreciate the reviewer’s suggestion to contextualize these findings. While the specific genetic associations identified in our study are novel, they align well with known clinical observations of ASFV infection.
Past studies have documented that chronic or subacute ASFV infection often leads to persistent growth retardation, emaciation (impacting production and meat traits), and reproductive failures such as abortions (impacting reproductive traits) 7.
Our study provides a novel genetic basis for these observations by identifying specific prioritized genes (e.g., TPST1, ABCG5) that are associated with both ASF resistance and these production/reproductive traits. We argue that the extensive genetic overlap and pleiotropic effects typically seen in complex traits 16 provide a theoretical basis for our observation that ASF resistance profiles are intrinsically linked to growth and reproductive traits.
This suggests that the observed clinical symptoms in infected pigs might be partly mediated by the pleiotropic effects or genetic linkage of resistance-related loci. We have updated the Discussion to include this context.
Revision excerpts: In lines 555-559, we added the following sentence
“Complex traits are inherently polygenic, and the finite number of genomic variants gives rise to widespread genetic overlap and pleiotropy across traits. Many phenotypes are associated with hundreds to thousands of loci, implying the presence of shared causal variants 102. Consistently, genetic correlation analyses indicate that some variants exert concordant effects on multiple traits 103.”
In lines 572-584, we added the following sentence
“Beyond immune-related functions, we observed that ASF resistance-prioritized genes are also associated with reproductive (e.g., litter weight) and production traits (e.g., body length). These genetic associations mirror the clinical manifestations of chronic ASFV infection, where affected pigs often exhibit progressive emaciation, growth stunting, and reproductive disturbances such as abortion 42. The involvement of key genes like TPST1 (TPST1-deficient mice experience decreased body weight and reproductive performance 106) and ABCG5 (mediates cholesterol metabolism 107) suggests that the physiological trade-offs observed during infection may have a partially shared genetic architecture. Our findings provide a genetic framework for understanding how disease resistance signatures may intersect with host fitness and performance. This underscores the necessity of balanced selection in breeding programs to enhance antiviral resilience without compromising essential production and reproductive outcomes.”

Line 330: Comment 1- It’s a nice result the APRS differentiates the pigs into groups. I assume this analysis is conducted with the SNP case-control study population. The population used to test the discriminatory power of the APRS needs to be defined here in the text.
Response: We thank the reviewer for pointing out the need for a clearer definition of the study population. We have now explicitly stated in the Results and Methods section that the discriminatory power of the APRS was evaluated using the original experimental case-control study population (n = 474 pigs), which served as our discovery cohort.
Revision excerpts:
Result: In lines 382-385, we changed the following sentence

“To facilitate the practical implementation of our findings in genetic improvement programs for ASF resistance, we constructed an ASF polygenic resistance score (APRS) based on the ASF-resistance prioritized loci identified in this study.” to
“To evaluate the practical implementation of our findings, we constructed an ASF polygenic resistance score (APRS) based on the ASF-resistance prioritized loci identified in this study and tested its performance within our discovery cohort (the experimental case-control population, n = 474).”
Method: In lines 1068-1070, we added the following sentence
“To assess the specificity and robustness of the APRS, we performed a randomized validation within our discovery cohort (the experimental case-control population, n = 474).”

Line 474: How were the SNPs selected within genes when there were multiple SNP? This was unclear to me. There is some explanation in the methods, but this seems lost in the results and discussion.
Response: We appreciate the reviewer’s comment regarding the SNP selection process within the prioritized genes. We have now clarified this in the Results and Methods sections. Briefly, our strategy followed a two-step approach: We identified 135 prioritized genes through an integrated multi-dimensional framework. Within the genomic regions of these genes, we selected the lead SNP (based on the lowest p-value) and applied LD clumping to retain 40 independent loci for the final APRS construction. This ensures that the APRS is based on the most statistically significant and independent signals within our high-confidence gene set.
Revision excerpts:
Result: In lines 385-388, we changed the following sentence
“The APRS was defined by aggregating favorable alleles across prioritized loci and calculating each individual pig’s score based on its identity-by-state (IBS) similarity to this ideal resistant genotype (Fig. 6d).” to
“The APRS was constructed by aggregating favorable alleles at independent lead loci within prioritized gene regions, with each individual’s score calculated based on identity-by-state (IBS) similarity to an ideal resistant genotype (Fig. 6d).”
Method: In lines 1034-1037, we changed the following sentence
“A total of 40 independent loci corresponding to the prioritized genes were selected to define the ideal ASF-resistant genotype.” to
“A total of 40 independent loci were selected to define the ideal ASF-resistant genotype. Specifically, within 135 high-confidence gene regions, the most significantly associated (p-values) SNPs were first identified as candidate loci. LD clumping (PLINK v1.9 124, --indep-pairwise 50 5 0.1) was then applied to retain independent, non-redundant lead SNPs for inclusion in the final APRS model.”

Line 331-336: I do not find the randomization analysis useful unless it was being used in the same genomic regions to show selected SNPs outperform those simply in LD. A better comparison would be to compare the discriminatory power of a SNP set identified from a genomic selection model for ASFV resilience. Why would the randomization analysis identify a relationship with ASFV prevalence?
Response: We appreciate the reviewer’s professional rigor. However, we would like to clarify the fundamental logic of our APRS construction, which differs from conventional black-box prediction models like Genomic Selection (GS).
Why APRS is not redundant with LD signals: While many SNPs in a genomic region may show statistical association due to LD, our 40 loci were not selected solely based on their p-values. They are anchored in 135 prioritized functional genes identified through an integrated framework (TWAS, SMR, colocalization and BN-GWAS). By choosing the lead SNPs from these biologically-relevant regions, the APRS serves as a basically-grounded index rather than a mere statistical aggregator. Our randomization analysis (random genotypes and random loci) confirms that the discriminatory power is specific to this functional gene set, which cannot be achieved by capturing general genomic noise.
APRS vs. GS: The primary objective of APRS is not to outperform GS in total genomic prediction accuracy, but to provide a parsimonious and interpretable tool for early screening of high-resistance individuals. Unlike GS, which requires genome-wide high-density markers, our APRS relies on a curated set of 40 functional anchors. This makes it more practical for targeted breeding and demonstrates that our multi-dimensional prioritization successfully condensed complex polygenic resistance into a concentrated genetic score.
Revision excerpts:
In lines 393-397, we changed the following sentence

“Randomized APRS scores failed to differentiate between resistant and deceased individuals (Fig. 6e and Supplementary Tables S21), confirming that the identified loci significantly contribute to ASF resistance and are not influenced by random variation.” to
“The randomized scores failed to differentiate between resistant and deceased individuals (Fig. 6e and Supplementary Tables S21), confirming that the the predictive power of APRS arises from biologically prioritized functional loci rather than stochastic inflation, and that it meaningfully contributes to the observed resistance phenotype.”

Line 338-341: Wasn’t the SNP set identified in the APRS developed using datasets from the PigBiobank? Were different studies used for validation or were the same datasets used for validation that were used to prioritize SNPs within the APRS?
Response: We appreciate the reviewer’s request for clarification on the relationship between the datasets. We have clarified this in the revised manuscript.
APRS: The SNPs used in APRS were derived from our experimental cohort, but their prioritization was based on a multi-dimensional framework integrating external functional evidence (e.g., TWAS, SMR, and etc., related to eQTL statistics from PigGTEx), ensuring that they are biologically grounded rather than merely reflecting cohort-specific statistical associations.
PGS: The GWAS summary statistics used to build the PGS for other health traits were derived from the independent PigBiobank dataset, which is entirely separate from our experimental population.
Validation strategy: The association analysis was conducted by applying these externally-derived PGS models to our experimental cohort. Therefore, the correlation between APRS and these PGS represents a cross-trait validation: it demonstrates that the genomic regions we prioritized for ASF resistance are also genetically linked to broader health and immune landscapes in the same individuals, using models trained on independent data.
Revision excerpts:
Result:
In lines 400-401, we added the following sentence
“To validate the reliability of the APRS, we evaluated its association with polygenic scores (PGS) for health traits derived from PigBiobank and subsequently applied to our experimental cohort.”
Method: In line 1082, we added the subtitle “PGS construction and parameter optimization”.
In lines 1084-1085, we added the following words “To ensure model robustness and prevent overfitting, PUMAS was used to subsample PigBiobank GWAS summary statistics, …”
In line 1092, we added the subtitle “Association analysis in the experimental cohort”.
In lines 1093-1097, we changed the following sentence
“The association between PGS and the APRS was evaluated using the Spearman rank correlation coefficient. To further examine the relationship, APRS values were divided into deciles, which highlights extreme phenotypes for better interpretability.” to
“These externally-trained PGS models were subsequently applied to the pigs in our experimental case-control population. The association between the externally-derived PGS and our internally-constructed APRS was evaluated using the Spearman rank correlation coefficient. To enhance interpretability and highlight extreme genetic profiles, the experimental population was divided into deciles based on APRS values.”

Line 330-Comment 2: Doesn’t a portion of the data used for this risk score (i.e. the first data filter) come from data from this same dataset used to do the discriminatory analysis using the APRS? So wouldn’t we expect this result given where the data came from to create the polygenic risk score? I realize you don’t per se have an independent population for cross-validation (or I didn’t understand there was an one in the text), but if the same data set used to create a filter to identify the SNPs in the APRS is also used in the validation of the risk score, then I think we have to take this result with some caution. Please clarify this point and update the manuscript as appropriate.
Line 474: If you are using the same population to create the APRS as you test the score in to discriminate samples, can you truly say it is robust? Please clarify.
Response: We appreciate the reviewer’s point regarding the importance of independent validation and the potential for overfitting. In the context of ASF, collecting high-quality biological samples from natural outbreaks is exceptionally challenging due to strict biosecurity protocols and compulsory culling policies. As a result, a separate, independent validation cohort was not available for this study. However, to ensure the scientific rigor and biological relevance of our findings, we implemented a multi-layered internal and

indirect validation framework:
Addressing potential overfitting: The prioritized loci were not selected solely based on their association p-values in this specific cohort, instead, they were prioritized using multiple independent lines of evidence, including TWAS, SMR, colocalization and BN-GWAS. This "biological filtering" ensures that the loci in the APRS represent high-confidence functional candidates rather than mere statistical noise from the current dataset.
Validation strategy: To further address this, we have emphasized the results of our randomization test (permutation), which showed that scores based on the same number of random loci and random allele failed to differentiate the groups. This confirms that the observed discriminatory power is specific to our prioritized gene set. We have also added a statement acknowledging this limitation and emphasizing the need for future validation in independent populations.
Cross-trait support: Beyond differentiating the case-control groups, we found that the APRS significantly correlates with the PGS of independent health-related traits (e.g., S_PLDWID, S_IGA, S_IFGIL10). These immune and physiological parameters were measured independently and were not used as filters during the initial gene prioritization. The fact that an APRS constructed for ASF resistance can predict these relevant immune landscapes strongly supports its biological robustness and functional utility.
Revision excerpts:
Result:
In lines 391-397, we changed the following sentence
"To evaluate the robustness of our scoring model, we compared APRS based on selected loci to scores derived from randomly selected loci or genotypes. Randomized APRS scores failed to differentiate between resistant and deceased individuals (Fig. 6e and Supplementary Tables S21), confirming that the identified loci significantly contribute to ASF resistance and are not influenced by random variation." to
"To further infer that this performance was not a byproduct of random chance or cohort-specific bias, we compared APRS to scores derived from randomly selected loci or alleles. The randomized scores failed to differentiate between resistant and deceased individuals (Fig. 6e and Supplementary Tables S21), confirming that the predictive power of APRS arises from biologically prioritized loci rather than stochastic inflation, and that it meaningfully contributes to the observed resistance phenotype."
In lines 419-424, we changed the following sentence
"These results highlight the utility of APRS in identifying traits associated with ASF resistance and provide indirect validation of its potential role in disease resistance breeding programs. Furthermore, our findings demonstrate that APRS is a robust genomic tool for evaluating ASF resistance potential, offering a promising approach for marker-assisted selection in pig breeding programs." to
"The concordance between APRS and independent immune-related traits indicates that APRS captures a biologically meaningful signal of host resistance rather than cohort-specific noise. Collectively, these findings support its utility as a robust genomic metric for evaluating ASF resistance potential and highlight its promise for application in marker-assisted breeding programs."
Discussion: In lines 655-669, we changed the following sentence
"Lastly, the APRS, though robust, focuses on known SNPs and may overlook contributions from rare variants or epigenetic factors, which future research should address through rare variant analysis and epigenomic profiling." to
"Lastly, while the APRS demonstrated high discriminatory power in our study, we acknowledge that this evaluation was conducted within the discovery cohort due to the inherent difficulty in obtaining independent validation samples during active ASF outbreaks. To mitigate potential performance inflation and ensure the scientific validity of the APRS, we employed a multi-layered verification strategy. First, our integrated multi-dimensional prioritization strategy (rather than relying solely on GWAS p-values) ensures that the included loci possess intrinsic biological relevance. Second, the failure of randomized loci to replicate this performance further supports the APRS captures specific genetic signals rather than stochastic variation. Third, high APRS scores correspond to a higher statistical likelihood of maintaining immune homeostasis, although absolute survival remains contingent upon the intensity of the viral challenge and environmental stressors. Future studies utilizing independent validation populations or diverse pig breeds will be essential to confirm the cross-population portability of this scoring system and its long-term utility in genomic selection programs."
Method: In lines 1055-1066, we added the following section
"Statistical Robustness and Indirect Validation of APRS
Given the logistical and biosecurity constraints of collecting large-scale independent cohorts during active ASF outbreaks, we implemented a multi-layered framework to evaluate the robustness and biological

consistency of the APRS within the study population.
i. APRS construction incorporated multi-dimensional evidence, relying on loci prioritized through an integrative framework rather than solely on GWAS significance, thereby ensuring independent functional support for each included locus.
ii. Internal robustness was assessed via permutation testing.
iii. Biological consistency was examined by correlating APRS with polygenic scores (PGS) for 59 independent health-related traits relevant to ASF resistance, including hematological and immune parameters."

Line 345-353: I think this paragraph needs some context. As written, the relevance is not clear. If these phenotypes are of high relevance to ASF based on published literature, then these findings are important. Can you add a little text here to help connect these results to ASF in some way with references that connect these traits to ASF please.
Line 478-486: These traits appear relevant to ASF, but are they? This needs to be better documented with references to tie the relevant pleiotropic traits to ASF to show the power of what is being done.
Response: We appreciate the reviewers' suggestion to provide more clinical and pathological context for the PGS traits correlated with APRS. We have updated both the Results and Discussion to explicitly connect these traits to known ASFV pathogenesis:
Coagulation & vascular homeostasis: ASFV infection triggers severe thrombocytopenia, disseminated intravascular coagulation (DIC), and vascular damage, which are reflected in alterations of platelet parameters (e.g., S_PLDWID) and red blood cell metrics (e.g., S_MCV).
Leukocyte dynamics: ASFV induces profound lymphopenia and leukopenia, with a general decline in white blood cell subsets, including eosinophils (S_EOS) 17.
Mucosal & systemic immunity: Host resistance relies on effective mucosal and systemic immune coordination. IgA plays a critical role in limiting viral entry at mucosal surfaces 18, while a balanced cytokine response, particularly the IFN-γ/IL-10 axis, helps control viral replication while preventing excessive inflammation and cytokine-mediated pathology 11. Consistent with these roles, ASFV infection is accompanied by increased IgA levels and concurrent upregulation of IFN-γ and IL-10, reflecting activation of both antiviral defense and immunoregulatory pathways 17,19.
Our findings that APRS positively correlates with the PGS of these traits suggest that the identified resistance loci may bolster the host's innate physiological resilience against these specific ASFV-induced pathologies. These additions clarify the biological significance of our APRS-PGS correlations and highlight the predictive power of the identified loci.
Revision excerpts:
Result: In lines 406-419, we changed the following sentence
"Our analysis revealed a significant positive correlation between the PGS for platelet distribution width (S_PLDWID) and APRS, consistent with the results from the genetic correlation analysis (Fig. 6a, f, Supplementary Table S18 and S22). Individuals in the top decile of APRS exhibited significantly higher PGS_S_PLDWID values compared to those in the bottom decile. Additionally, positive correlations were observed between APRS and the PGS for several other traits, including mean corpuscular volume (S_MCV), lipases (S_LIPA), eosinophil number (S_EOS), IFG-IL10 ratio (S_IFGIL10), and IgA level (S_IGA), as determined by both Lassosum and PRSice-2 (Fig. 6f and Supplementary Table S22). to
"Our analysis revealed a significant positive correlation between APRS and the PGS for platelet distribution width (S_PLDWID), consistent with the results from the genetic correlation analysis (Fig. 6a, f, Supplementary Table S18 and S22). Individuals in the top decile of APRS exhibited significantly higher PGS_S_PLDWID values compared to those in the bottom decile. This is highly relevant as ASFV infection typically leads to thrombocytopenia and disseminated intravascular coagulation (DIC) 63, suggesting that a genetic predisposition for robust coagulation regulation may support resistance. Additionally, APRS positively correlated with PGS for traits critical to viral defense and host homeostasis, such as IgA levels (S_IGA), which serve as a primary mucosal barrier 64, and the IFN-γ/IL-10 ratio (S_IFGIL10), a marker of the pro- vs. anti-inflammatory balance 65 often disrupted by ASFV-induced cytokine storms 63,66. We also observed correlations with mean corpuscular volume (S_MCV) and eosinophil counts (S_EOS), traits known to be impacted by ASFV-mediated vascular damage and leukopenia 63,66 (Fig. 6f and Supplementary Table S22)."
Discussion: In lines 590-604, we changed the following sentence
"… The pleiotropic effects of the prioritized genes and the utility of the APRS were further validated through the association between APRS and polygenic scores (PGS) derived from various health-related traits.

Notably, APRS exhibited significant correlations with PGS for traits such as S_PLDWID, mean corpuscular volume (S_MCV), lipase activity (S_LIPA), eosinophil counts (S_EOS), the IFG-IL10 ratio (S_IFGIL10), and IgA levels (S_IGA). These traits reflect key aspects of systemic immune regulation, metabolic stability, and adaptive immune responses, reinforcing the notion that loci associated with ASF resistance exert pleiotropic effects across multiple biological systems." to
"… Its utility is further supported by concordance with the polygenic architecture of traits relevant to ASFV pathogenesis. Polygenic scores (PGS) capture the cumulative effects of multiple variants and are widely used to estimate genetic predisposition, stratify risk, and interrogate trait associations 102,108,109. The positive correlation of APRS with S_PLDWID and mean corpuscular volume (S_MCV) PGS suggests a genetic link between ASF resistance and the maintenance of hemostatic and vascular integrity, counteracting virus-induced coagulopathy and endothelial damage 63. Associations with IgA levels (S_IGA) and the IFG-IL10 ratio (S_IFGIL10) PGS highlight the importance of coordinated immune responses, integrating mucosal defense and cytokine balance to limit viral entry and immunopathology 64,65. Moreover, given ASFV-induced lymphopenia and leukopenia, including reduced eosinophils (S_EOS) 66, the positive correlation between APRS and leukocyte-related PGS indicates that resistant individuals may harbor more resilient hematopoietic and immune systems. Collectively, these findings support pleiotropic effects of prioritized loci across multiple physiological systems, contributing to a multilayered defense against ASFV."

Line 406: Which 3 methods? Define them here in the text to clarify.
Response: We appreciate the reviewer's suggestion to clarify the specific validation methods. We have updated the text to clarify that these genes are supported by varying combinations of three evidence lines.
Revision excerpts: In lines 481-483, we changed the following sentence
"In addition, functional insights into genes validated by three methods suggest diverse biological roles: …" to
"In addition, functional insights into genes supported by at least three independent lines of evidence (drawn from a pool of five methods: Reported immune genes, TWAS, SMR, colocalization, and BN-GWAS), suggest diverse biological roles: …"

Line 550: Please provide the breed composition of these pigs.
Response: We thank the reviewer for this request. The study population (n = 474) was sampled from four commercial farms under the same enterprise in Jinan, Shandong Province, between April 2021 and June 2022. The breed composition consists of three groups: a local Shandong breed (LWU, n = 90), a developed commercial breed (LLA, n = 355), and their hybrids (DLL, n = 29). The cohort included 240 boars and 234 sows, all maintained under identical commercial management and natural infection exposure. Due to confidentiality agreements regarding commercial genetic lines, we have used these abbreviations (LWU, LLA, and DLL) throughout the manuscript. However, we have added a more detailed description of the population structure and sampling process in the Methods section and provided individual-level data (anonymized for breed names) in Supplementary Table 1 to ensure transparency.
As for population stratification, we performed a PCA specifically on our experimental cohort to assess potential confounding effects on the downstream GWAS. As shown in the updated manuscript and Supplementary Fig. S1, the PCA results revealed:
Limited Breed Stratification: While some dispersion was observed between breeds, the population showed weak overall structure.
No Phenotypic Clustering: Crucially, resistant and susceptible individuals largely overlapped in the PCA plot, indicating that disease status is not confounded by major genetic background differences.
These results confirm that the population structure is modest and does not drive phenotypic stratification, providing strong support for the robustness of our subsequent GWAS analyses. We have added these details to the Results sections.
Revision excerpts:
Results: In line 161-165, we added the following sentence
"PCA within the experimental population showed minimal stratification by breed and extensive overlap among disease phenotypes, indicating weak population structure and no evident confounding by genetic background (Supplementary Fig. S1). Together, these results support the suitability of this cohort for genome-wide association studies (GWAS)."
Methods: In lines 683-689, we changed the following sentence
"All pigs were maintained under standardized management and environmental conditions representative of

commercial production.” to
“The experimental population comprised 474 domestic pigs naturally exposed to ASFV across four standardized commercial farms under the same enterprise in Shandong, China. The cohort included a local indigenous breed (LWU, n = 90), a developed commercial breed (LLA, n = 355), and their crossbred offspring (DLL, n = 29), consisting of 240 boars and 234 sows. All individuals were raised under uniform environmental and management conditions representative of large-scale commercial production.”

Line 583: The sequencing technology needs a reference.
Response: Thank you for the suggestion. We have updated the manuscript to include the manufacturer information for the sequencing platform (MGI, Shenzhen, China) (line 742). As the DNBSEQ-T7 is a standardized hardware platform, we have provided its full technical specifications and manufacturer details to ensure reproducibility, which is the standard practice in recent literature.

Line 634: The software Gallo needs a reference.
Response: Thank you for this reminder. We have now provided the appropriate reference for the GALLO package in line 794.

Line 1290-1291- Figure 3 legend: What is the difference between score value and total prioritization score?
Response: We thank the reviewer for the valuable feedback on our terminology. We have clarified the distinction between “individual scores” (scores assigned by each genomic method, including GWAS, FST, TWAS, SMR, etc.) and the “aggregate prioritization score” (the sum of individual scores for a given gene). Additionally, we have replaced “validated” with “supported” to more accurately reflect the computational nature of our gene prioritization process.
Revision excerpts: In lines 1570-1577, we changed the following sentence
“Gene prioritization overview for the top prioritized genes for ASF resistance. Genes are ranked by their total prioritization scores (high to low). The heat map displays scores across various validation methods, with the y-axis representing prioritized genes and the x-axis showing different scoring methods. Color intensity indicates the score value. The lollipop plot shows the total prioritization score for each gene. Genes with scores ≥ 3.5 or validated by two or more methods are classified as prioritized genes.” to
“Overview of the gene prioritization framework for ASF resistance. Genes are ranked by their aggregate prioritization scores (from high to low). The heatmap displays individual scores across various genomic methods (GWAS, FST, Reported immune genes, TWAS, SMR, colocalization, BN-GWAS), with color intensity representing score values. The lollipop plot illustrates the aggregate prioritization score, calculated by summing individual scores. Genes were identified as prioritized candidates if they achieved an aggregate score ≥ 3.5 or were supported by two or more independent methods.”

Line 1333- part f of Figure 4: The cell types (listed as abbreviations in the actual figure) used in this analysis need to be defined in the figure legend.
Response: We thank the reviewer for pointing this out. We have updated the legend for Figure 4 to explicitly define all cell type abbreviations used in parts d, e, and f.
Revision excerpts: In lines 1620-1622, we added the following sentence
“Mac: macrophages. Mast: mast cells. T: T cells. Pro: proliferating cells. Epi: epithelial cells. Four macrophage subtypes: Mac_CD163, Mac_HLA_DRA, Mac_CREG1, and Mac_PLBD1.”

Line 1348- figure 6 part c: the PP3 and PP4 need to be defined in the figure legend.
Response: Thank you for this helpful suggestion. We have now provided the full definitions for PP3 (Posterior Probability of hypothesis 3) and PP4 (Posterior Probability of hypothesis 4) in the revised figure legend (lines 1638-1639, 1589-1590). Besides, we updated the legend title to “Cross-trait colocalization analysis” to more accurately reflect the scope of this analysis and avoid any ambiguity (line 1635).

Line 1355: The asterisks in the legend are not very visible in the actual figure at line 1403.
Response: We appreciate the reviewer’s careful observation. To improve visibility, we have enlarged the asterisks representing statistical significance in both Figure 6e, f (line 1693). Additionally, we will provide high-resolution graphics (Tiff format) during the final submission to ensure that all symbols and annotations remain sharp and clearly legible in the published version.

Line 1380- Table 1: An explanation of what comparison 1, 2, 3, 4 are, is needed in the footnotes of the

table.
Response: Thank you for this helpful suggestion. To improve the clarity and self-explanatory nature of Table 3, we have added a brief definition of Comparisons 1, 2, 3, and 4 to the footnotes. These definitions specify the clinical phenotypes being compared in each analysis (e.g., Uninfected vs. Dead). We have also included a reference to Table 2, where the detailed experimental grouping and biological rationale for each comparison are described.
Revision excerpts: In line 1670, we added the following sentence
"Comparisons 1–4 are defined as follows based on clinical outcomes: 1, (Uninfected + Survived) vs. Dead; 2, Uninfected vs. (Survived + Dead); 3, Uninfected vs. Dead; 4, Survived vs. Dead. Detailed groupings are provided in Table 2."

Line 1387- Figure 1: Not all details in the figure are clear. For example, in the top red portion, why are the pigs in the experimental data used for analysis shaded in different colors? The authors should make sure the other details within this diagram are well described as well.
Response: We appreciate the reviewer's feedback on improving the clarity of Figure 1. We have updated the figure legend and the schematic details as follows:
Red section: color coding of pigs; refinement of methodological labels (antigen/antibody status); symbol meaning of "+".
Yellow and green sections: numerical notation (e.g., 31/64); symbol meanings of "↔/→"/network.
Abbreviations: AF, PAM, PBMC, PGS, APRS.
Revision excerpts: In lines 1516-1520 (red), we added the following sentence
"Pig icons are color-coded by clinical phenotype: blue for uninfected (Antigen-/Antibody-), green for survived (Antigen-/Antibody+), and red for deceased (Antigen+). Grey icons represent a background population used for FST and AF tests. The "+" sign indicates the integration of association signals from GWAS and population genetic differentiation."
In lines 1524-1530 (yellow), we added the following sentence
"Numerical ratios (e.g., 31/64) indicate the yield of each method: the numerator denotes prioritized genes, while the denominator represents total candidates processed by that analysis. The double-headed arrow (↔) in TWAS denotes associations between gene expression and traits; the single-headed arrow (→) in SMR/Coloc indicates potential causal or regulatory effects from eQTL to phenotype. The network icon in BN-GWAS represents inferred gene-gene-phenotype causal relationships."
In lines 1541-1547 (abbreviations), we added the following sentence
"AF test: allele frequency chi-square test. PAM: porcine alveolar macrophage. PBMC: peripheral blood mononuclear cells. PGS: polygenic score. APRS: African swine fever resistance prediction score."

Figure 2: I appreciate that the authors have color coordinated between figure 1 and 2, but figure 2 needs to be able to stand alone. For example, in part a, it's not clear what comparisons or analyses the different bars and lines below the bars correspond to. For part b, what is comparison 1, 2, 3, and 4? For part c, there needs to be a list of what these different methods are based on color used in the figure that are displayed.
Response: We thank the reviewer for the suggestion to make Figure 2 more self-explanatory. We have thoroughly revised the figure legend to ensure it can stand alone:
Part a: We added an explicit legend within the text to define the symbols and colors used in the Upset plot.
Part b: We adopted a description style similar to the "Fuji plot" to better characterize the genomic distribution of the loci.
Part c: We provided a detailed explanation of the stacked bar plot, clarifying that it represents the cumulative support from multiple analytical methods.
Terminology consistency: A dedicated section defining Comparisons 1–4 has been added to the end of the legend to ensure clarity without redundant text in each sub-panel description.
Revision excerpts: In line 1548-1568, we changed the following sentence
"Figure 2. Identification and characterization of candidate genes associated with ASF resistance.
a. Upset plot showing the overlap of candidate genes identified across different comparisons. The black lines and colored dots below the bar graph represent specific comparisons, while the bar graph indicates the number of candidate genes identified in those comparisons.
b. Genomic distribution of identified genetic loci. The outermost circle displays chromosomes with annotated prioritized genes. The middle layer uses different colors to represent different comparisons: red for comparison 1, yellow for comparison 2, green for comparison 3, and blue for comparison 4. Within each

comparison group, loci identified by specific methods are depicted in sequential rings from outer to inner: GWAS, FST (case vs. control 1), MAF chi-square test (case vs. control 1), FST (case vs. control 2), and MAF chi-square test (case vs. control 2).
c. Inner stacked plot summarizing the number of validation methods supporting candidate loci within each comparison group." to
"Figure 2. Integrative identification and genomic landscape of candidate genes associated with ASF resistance.
a. Overlap of candidate genes across analytical comparisons. The Upset plot illustrates the intersection of genes identified in four clinical comparisons (1–4). The vertical bars indicate the number of genes unique to or shared between comparisons, as indicated by the connected dots below.
b. Fuji plot showing the genomic distribution of identified loci. The outermost circle displays chromosomes with annotated prioritized genes. In the middle layers, each dot represents a significant locus associated with a specific comparison (1-4). Within each categorical track, the concentric rings (from outer to inner) represent five distinct statistical methods: GWAS, FST (case vs. control 1), AF test (case vs. control 1), FST (case vs. control 2), and AF test (case vs. control 2).
c. Methodological support for candidate loci. The inner stacked bar plot summarizes the cumulative number of statistical methods (out of the five mentioned above) supporting the candidate loci within each genomic region. The height and color composition of each bar directly correspond to the loci displayed in part b.
Comparison 1: (Uninfected + Survived) vs. Dead (General resistance/protection); Comparison 2: Uninfected vs. (Survived + Dead) (Pathogen recognition/avoidance); Comparison 3: Uninfected vs. Dead (Strict protection); Comparison 4: Survived vs. Dead (Adaptive immunity/recovery). GWAS: genome-wide association study; FST: fixation index; AF test: allele frequency chi-square test."

General comment regarding the figures: Please check that these images are of high enough image quality to meet the standards of Gigascience. When they are enlarged, the text and images begin to become rasterized.
Response: We apologize for the resolution issues in the previous version. We have now uploaded all figures as high-resolution Tiff files (at least 300 DPI) to ensure that all text and graphical elements remain sharp and clear, even when enlarged, meeting the publication standards of Gigascience.
Reviewer #2:
The authors have conducted a comprehensive study integrating whole-genome sequencing, GWAS, selection scans, and multiple functional genomic approaches to investigate host resistance to African Swine Fever. Through this multi-dimensional strategy, they prioritized 135 high-confidence resistance genes, revealed tissue- and cell-type-specific expression patterns, identified key immune pathways and cellular mediators, and developed a polygenic resistance score that discriminates phenotypic outcomes. Overall, this work represents a substantial effort to establish a genomic foundation for precision breeding of ASF-resistant pigs. This study addresses host resistance to ASF, a topic of high significance. The authors have made considerable efforts to expand the scope of the research and enhance its scientific rigor. However, several details require further clarification or revision to strengthen the conclusiveness of the findings:

Major:
1. My major concern regarding this study is about the types of the ASF virus that had induced the immune response in the pig population of focus. The authors failed to detect the DNA fragment of p22 but found the antibody ELISA test gave positive results, and this pattern implies that this is not a typical infection by the wild type virus. The Eurasian domestic pigs (Sus scrofa) usually cannot survive two weeks after the infection of the wild type ASF except for some local/indigenous pigs in Africa, and the observed high fraction of antibody positives in the sampled Chinese indigenous pigs is not likely caused by the wild types. In the antibody test, the positive results indicate the detection of any of the P32, P62, and P72 ASF proteins. The encoding genes of the three proteins were usually not included in typical attenuated vaccine development. For the KP177R of p22 in the antigen DNA test, the situation is a bit different, and the p22 gene was widely used in the development of live attenuated vaccine (p22-knockout). The p22-knockout is usually used to indicate the difference between a wild-type virus (positive) or an attenuated vaccine (negative).
Despite the immune response induced in the survived pigs is less likely caused by the wild-type ASF virus, the observation here did contribute substantial importance to the swine industry. The multi-gene deletion ASF strains have consequential impacts even more significant than the wild-type virus given the challenges in detecting them under long incubation period, mild-to-modest levels of symptom after infection. This

constitutes a new challenge due to the "hidden" nature. Actually, this study provides some important clues to understand the genetics behind such low-virulence infection.

I strongly suggest the authors to clarify the pattern of antibody and antigen tests, and revise the manuscript to specify the scope of this study to the immune response associated with putative low-virulence infection induced by the attenuated virus. This is an important topic for this swine industry.
Response: We sincerely thank the reviewer for pointing out the infection pattern. We agree that the p22 antigen-negative/antibody-positive profile strongly indicates a low-virulence or gene-deleted ASFV infection. We have refined the manuscript as follows:
Scope refinement (abstract & results): We explicitly specified that the host genetic resistance identified refers to low-virulence ASFV strains. We emphasized that the divergent outcomes under shared exposure highlight the host's genetic contribution to disease resistance.
Methodological clarification (methods & Table 1): We clarified the diagnostic strategy: ELISA (P32/P62/P72) serves as a broad-spectrum exposure marker, while the absence of p22 (KP177R) acts as an indicator for gene-deleted variants.
Enhanced discussion: A dedicated section was added to discuss the "hidden" nature of low-virulence strains and why host immune homeostasis is critical for managing sub-acute viral persistence.
Narrative consistency: While retaining the broad title for visibility, the text now consistently reflects the low-virulence infection context.
Revision excerpts:
Abstrat: In line 35, we added the following words
"Observations of surviving individuals within low-virulence infected herds suggest that host genetic resistance plays a crucial role."
Result: In lines 150-157, we changed the following sentence
"We analyzed a genomic dataset comprising 474 individuals, retaining 23,290,599 …" to
"To characterize genomic differences underlying ASF infection outcomes, we analyzed 474 pigs with antigen/antibody phenotyping and WGS (Supplementary Table S1). In a herd naturally exposed to low-virulence ASFV, 108 individuals died, 222 survived, and 144 remained uninfected (Table 1). These divergent phenotypes, observed under consistent exposure conditions and supported by longitudinal sampling, indicate a substantial host genetic contribution to disease variability.
For genomic analysis, the WGS dataset yielded 23,290,599 …"
Discussion: In lines 427-447, we changed the following sentence
"To address this gap, our study employed a multi-dimensional strategy integrating whole-genome sequencing, functional annotation, transcriptomic annotation, and predictive modeling to dissect the genomic architecture of ASF resistance in pigs." to
"While acute infections with wild-type ASFV typically result in near-complete mortality within two weeks 42,67,68, the high proportion of antibody-positive (P32, P62, P72) but antigen-negative (p22) individuals observed among survivors in this study suggests exposure to low-virulence, gene-deleted, or variant strains rather than classical virulent isolates. Such attenuated strains represent a "hidden" epidemiological challenge: they cause milder symptoms and prolonged infection courses, complicating detection and control. Elucidating host genetic responses under these conditions is therefore critical for developing effective breeding strategies in endemic settings.
In this context, our study delineates the genetic architecture underlying host responses to low-virulence ASFV infection. … By integrating WGS with functional and transcriptomic annotations, alongside predictive modeling, we established a comprehensive framework to dissect the polygenic nature of ASF resistance in pigs, ..."
Method: In lines 704-724, we changed the following sentence
"Blood samples were collected from 474 individuals of an indigenous pig population for phenotypic testing. Antibody and antigen detection were performed using the Indirect ELISA colloidal gold test strip method (ID. Vet, France) and the RAA fluorescence quantification method (AMPLIFICATION FUTURE, WLE8202KIT), respectively, following the manufacturers' instructions. For antibody detection, the ID. Vet Indirect ELISA kit (https://www.innovative-diagnostics.com/produit/id-screen-african-swine-fever-indirect/) was used to target ASFV structural proteins P32, P62, and P72, which are highly conserved and widely adopted in ASF diagnostics. For viral DNA detection, the assay targeted the KP177R sequence within the p22 coding region of the ASFV strain China/2018/AnhuiXCGQ (MK128995.1), a region known for its high sequence conservation. Amplification was conducted using a rapid DNA amplification kit, and fluorescence-based probe detection was employed for confirmation and quantification, ensuring high sensitivity and specificity

in ASFV identification.” to
“To assess the infection status and the nature of the circulating ASFV strain, we employed a diagnostic strategy combining broad-spectrum antibody profiling with targeted antigen DNA detection.
Antibody detection was performed using the ID Screen African Swine Fever Indirect ELISA kit (ID. Vet, France) according to the manufacturer’s instructions. This assay targets the ASFV structural proteins P32, P62, and P72, which are highly immunogenic and conserved across a wide range of ASFV lineages, serving as reliable markers for both wild-type and attenuated strain exposure.
Antigen DNA detection was conducted using the RAA fluorescence quantification method (AMPLIFICATION FUTURE, WLE8202KIT). The assay specifically targeted the KP177R sequence within the p22 coding region, derived from the ASFV strain China/2018/AnhuiXCGQ (MK128995.1). While p22 is a highly conserved structural protein in wild-type ASFV, the KP177R is a recognized hotspot for deletion or mutation in various low-virulence variants and gene-deleted strains. Therefore, the inclusion of this specific target allows for both high-sensitivity detection of viral and the identification of potential gene-deleted variants based on the presence or absence of the p22 genomic fragment. Rapid DNA amplification and fluorescence-based probe detection were employed to ensure maximum sensitivity and specificity in distinguishing ASFV infection patterns.”
Table: In line 1666, we changed the following table note
“1 African swine fever virus antigen test, - for negative and + for positive.
2 African swine fever virus antibody test, - for negative and + for positive.” to
“1 ASFV antigen test: Detected via the RAA fluorescence method targeting the KP177R (p22 protein), - for negative and + for positive
2 ASFV antibody test: Detected via indirect ELISA targeting structural proteins P32, P62, and P72, - for negative and + for positive”

2. The definitions of ‘resistance’ and ‘tolerance’ are not clearly distinguished. Based on the current results, the phenotype described may align more closely with ‘tolerance’ rather than true resistance (i.e., limiting disease severity rather than pathogen burden). The authors are encouraged to clarify the conceptual framework and explicitly justify the use of ‘resistance’, or alternatively consider adopting ‘tolerance’ where appropriate. Expanding this discussion would help avoid potential misinterpretation and better align the study with established terminology in host-pathogen interaction research.
Response: We appreciate the reviewer’s insightful comment on the terminology. We agree that in the context of evolutionary biology, “resistance” and “tolerance” represent distinct biological mechanisms. However, in the field of animal breeding and livestock genetics, “disease resistance” is a well-established and widely used umbrella term that encompasses the host’s overall ability to survive or remain healthy under pathogen pressure. This terminology aligns with existing studies on host genetics in ASF infection 20,21.
Nevertheless, we acknowledge the importance of conceptual clarity. In the revised manuscript, we have now included an explicit definition of “resistance” as used in this study (General disease resistance) to justify our terminology and discuss how our findings may involve both reduced viral impact and enhanced survival.
Revision excerpts:
In lines 438-443, we added the following sentence
““Resistance” refers to the host’s ability to prevent or control viral infection, including reducing the likelihood of infection or limiting viral replication and spread once infected 69. In contrast, “tolerance” describes the host’s ability to withstand the effects of established infection, with a tolerant individual experiencing attenuated disease impact 69. In this study, “resistance” is defined as the host’s ability to survive infection.”

3. The current study does not fully exclude the potential influence of other pathogens or environmental factors, and therefore the identified signals may not be strictly ASF-specific. It would be beneficial for the authors to more explicitly acknowledge this limitation and discuss its potential impact on the interpretation of results. In particular, reframing the findings in the context of general or multi-pathogen immune responses, or revising the title and scope accordingly, would improve conceptual accuracy and strengthen the overall narrative.
Response: We appreciate the reviewer’s insightful suggestion. We acknowledge that in a natural exposure environment, the identified genetic signals may encompass both ASFV-specific responses and general immune landscape triggered by potential co-infections or environmental stressors.

However, we believe this “limitation” reflects the biological reality of swine production. Most genes identified in controlled, single-pathogen challenges represent “specific resistance”, while our study conducted under natural exposure captures the host’s integrated survival capacity. The identified pathways, such as interferon signaling and inflammatory regulation, likely contribute to the host’s general disease resilience and maintenance of immune homeostasis under complex challenges. Following your suggestion, we have added a dedicated section in the Discussion to explicitly acknowledge this and reframe our findings within the broader context of multi-pathogen immune defense.
Revision excerpts:
In lines 617-630, we added the following paragraph
“It is important to acknowledge that the genetic signals identified in this study were captured under natural ASFV exposure, where environmental complexities and potential co-infections cannot be fully excluded. Consequently, the candidates identified may represent a combination of ASFV-specific resistance and general disease resistance. While traditional research often focuses on “specific resistance” against a single pathogen 110,111, the quest for “general resistance” remains a major challenge in swine breeding. Our findings, particularly genes involved in the regulation of inflammatory responses and interferon pathways, reflect the host’s capacity to maintain immune homeostasis under multi-pathogen challenges. By integrating these diverse candidate genes into a unified regulatory network, this study provides a systematic map of the host’s genetic defense, spanning from “avoiding infection” and “controlling viral replication” to “tolerating tissue damage and preventing mortality”. This holistic perspective underscores the potential of these loci in breeding programs aimed at improving overall host fitness in complex, real-world production environments.”

4. In the Introduction, additional context on the feasibility and current progress of breeding for host resistance to ASF would help better position this study within the existing literature. For example, a brief overview of known genetic resistance mechanisms, previous GWAS or functional studies, and the practical challenges in defining ASF resistance phenotypes would provide useful background. Expanding this section would enhance the significance and motivation of the study.
Response: We sincerely thank the reviewer for this constructive suggestion. We have significantly expanded the Introduction to provide a more comprehensive background on ASF host resistance:
A discussion on the biological feasibility of resistance, citing evidence from survivors of ASFV outbreaks and the role of innate immune regulation.
A summary of previous GWAS and functional studies that identified key candidate regions, noting both their contributions and the remaining gaps in our understanding of the underlying causal signatures.
The definition of “resistance”.
These additions better position our study within the current landscape of ASF research and emphasize the necessity of our integrated prioritization strategy.
Revision excerpts: In lines 97-106, we added the following sentence
“…, which likely modulates immune activation, coordination, and regulation to limit immunopathology and promote survival or infection tolerance 20. In this study, we define “resistance” broadly as the host’s capacity to survive infection, encompassing both the ability to limit viral replication and to mitigate disease severity. Host immune responses play a central role in shaping the heterogeneous clinical outcomes of ASFV infection, reflecting complex virus–host interactions. For example, asymptomatic pigs exhibit higher NK cell activity but lower IgA and virus-specific antibody levels compared with susceptible individuals 21. Functional studies further demonstrate that innate antiviral effectors, such as MxA and IFITM proteins, can directly inhibit ASFV replication 22,23. …”
In lines 113-128, we added the following sentence
“Current research on ASF host resistance has largely focused on identifying candidate genes, particularly through interspecies comparisons. The contrasting responses between warthogs and domestic pigs represent a well-established model: despite similar viral replication levels, clinical outcomes differ markedly, highlighting a dominant role for host factors 24. Genomic studies have revealed adaptive divergence in immune-related genes, including Mx1, Mx2, and PTGS2, implicating ASF as a selective pressure in warthog evolution 25. Comparative and functional analyses have further identified candidate resistance genes such as ISG15, HERC5, TRIM21, and RELA, as well as structural variants affecting loci like LDHB and TRIM family genes that modulate viral replication 26,27. In domestic pigs, emerging evidence also supports a genetic basis for variation in ASF outcomes, with distinct disease progression observed across breeds such as Ugandan pigs and Xiang pigs 28,29. However, most studies rely on limited sample sizes or single-omics approaches, resulting in inconsistent findings and incomplete genetic insight. A

comprehensive, large-scale, multi-omics framework is therefore required to systematically resolve the genetic architecture and molecular basis underlying host variation in ASF infection."

5. Additional details on the GWAS model are needed to improve methodological transparency. In particular, the assumed distribution of effect sizes (e.g., normally distributed SNP effects in a mixed linear model) should be explicitly described. Providing this information would enhance reproducibility and allow readers to better assess the robustness of the analysis.
Response: Thank you for this valuable suggestion regarding methodological transparency. We have now expanded the description of our GWAS model in the Methods section.
Revision excerpts: In line 801-816, we changed the following sentence
"$y=\mu+xb+\sum_k w_{ik} u_k+e$,
where y denotes the grouping of an individual (1 for control, 2 for case), μ is the fixed mean term, x is the genotype variable for a SNP, b is the fixed effect that is a function of the difference in allele frequencies between the two populations, $\sum_k w_{ik} u_k$ is the fit term for all SNPs on the other chromosomes to control for population differentiation, w_k is the standardized genotype variable for an SNP k, u_k is the corresponding effect size of SNP k, and e is the residual error term." to
"$y=\mu+Xb+Zc+\sum_k w_{ik} u_k+e$,
where y denotes the grouping of an individual (1 for control, 2 for case); μ is the fixed intercept; X is the genotype variable for a SNP (encoded as 0, 1, and 2, corresponding to the three allelic states), b is the fixed effect that is a function of the difference in allele frequencies between the two populations; Z is the design matrix of additional fixed-effect covariates, and c is the vector of effects for sex (2 levels) and farm (4 levels); $\sum_k w_{ik} u_k$ is the fit term for all SNPs on the other chromosomes to control for population differentiation, w_k is the standardized genotype variable for an SNP k, u_k is the corresponding effect size of SNP k, assuming to follow a normal distribution with variance proportional to $p0(1-p0)FST$, where $p0$ denotes the allele frequency in the ancestral population; and e is the residual error term, assumed to follow a normal distribution 132. This modeling framework effectively accounts for population structure and provides greater statistical power than conventional linear regression approaches 133. To further mitigate proximal contamination, a LOCO (leave one chromosome out) scheme was implemented, in which SNPs on all other chromosomes were included as random effects when testing the SNP of interest."

6. In the population structure analysis, while differences between breeds are described, it remains unclear whether population stratification exists within the experimental cohort itself. Since this could influence downstream association analyses, it is recommended to perform and present a PCA specifically on the study population. Including these results would strengthen confidence that population structure has been adequately controlled.
Response: We appreciate the reviewer's constructive suggestion regarding population stratification. Following this recommendation, we performed a PCA specifically on our experimental cohort to assess potential confounding effects on the downstream GWAS. As shown in the updated manuscript and Supplementary Fig. S1, the PCA results revealed:
Limited breed stratification: While some dispersion was observed between breeds, the population showed weak overall structure.
No phenotypic clustering: Crucially, resistant and susceptible individuals largely overlapped in the PCA plot, indicating that disease status is not confounded by major genetic background differences.
These results confirm that the population structure is modest and does not drive phenotypic stratification, providing strong support for the robustness of our subsequent GWAS analyses. We have added these details to the Results sections.
Revision excerpts: In line 161-165, we added the following sentence
"PCA within the experimental population showed minimal stratification by breed and extensive overlap among disease phenotypes, indicating weak population structure and no evident confounding by genetic background (Supplementary Fig. S1). Together, these results support the suitability of this cohort for genome-wide association studies (GWAS)."

Minor:
7. Line 130 Correct spelling 'fixation'.
Response: Thank you for pointing out this typo. We have corrected the spelling of "fixation" on line 170 as suggested.

8. Line 196 Change 'implicated' to 'implicated in'.
Response: Thank you for pointing out this typo. In line 215-223, we changed the following sentence "These pathways, previously implicated immune gene prioritization, underscore the critical roles of chemokine signaling, MAP kinases, and IL-15 in mediating ASF resistance. to
"..., exhibit high concordance with the characterized pathological features of ASFV infection, particularly regarding the systemic inflammatory cascades and the profound lymphopenia that define acute ASF 46–48."

9. Line 205 Clarify that 'enrichment' refers to heritability enrichment (h^2 enrichment) to avoid confusion with expression enrichment.
Response: We agree with the reviewer that "enrichment" could be ambiguous here. As suggested, we have revised the text to explicitly state "heritability enrichment" (lines 260, 263) to distinguish it from expression enrichment.

10. Line 214-216 Consider adding a sentence to clarify the rationale for using mouse embryo spatial transcriptomic mapping, e.g., that it serves as a "spatial expression reference framework" rather than a direct disease model.
Response: We appreciate the reviewer's constructive suggestion regarding the rationale for the mouse embryo spatial transcriptomic analysis. Upon careful re-evaluation of the manuscript's scope and the methodological rigor required for cross-species comparisons, we agree that using mouse embryonic data as a spatial reference for ASF resistance in pigs might introduce interpretational uncertainties. To maintain a more focused and rigorous narrative, we have decided to remove this specific analysis and the related discussion from the revised manuscript. This allows the study to remain centered on the porcine-specific multi-omics evidence and functional validation.

11. Line 222 Change 'exhibited' to 'exhibiting'.
Response: Thank you for pointing out this typo. We have corrected the spelling of "exhibiting" on line 270 as suggested.

12. Line 254 Delete the 2nd 'in'.
Response: Thank you for pointing out this typo. We have delete the 2nd "in" on line 310 as suggested.

13. In the discussion, the mention of APRS appropriately emphasizes its role in capturing "polygenic resistance tendencies" rather than implying absolute disease resistance.
Response: We are grateful for the reviewer's recognition of our interpretation of the APRS. We agree that the genetic basis of ASFV resistance is complex and polygenic; therefore, the APRS is designed to capture the cumulative genetic propensity for survival rather than to serve as a binary predictor of absolute resistance. As suggested, we have ensured that the Discussion emphasizes the APRS as a tool for ranking individuals within a breeding population to increase the overall frequency of resilience-associated alleles.
Revision excerpts: In lines 655-669, we changed the following sentence
"Lastly, the APRS, though robust, focuses on known SNPs and may overlook contributions from rare variants or epigenetic factors, which future research should address through rare variant analysis and epigenomic profiling." to
"Lastly, while the APRS demonstrated high discriminatory power in our study, we acknowledge that this evaluation was conducted within the discovery cohort, which may introduce a degree of inflation in the performance metrics. However, our use of an integrated multi-dimensional prioritization strategy (rather than relying solely on GWAS p-values) helps ensure that the included loci possess intrinsic biological relevance to ASF resistance. The failure of randomized loci to replicate this performance further supports the specificity of our APRS. High APRS scores correspond to a higher statistical likelihood of maintaining immune homeostasis, although absolute survival remains contingent upon the intensity of the viral challenge and environmental stressors. Future studies utilizing independent validation populations or diverse pig breeds will be essential to confirm the cross-population portability of this scoring system and its long-term utility in genomic selection programs."

References:
1. Gamazon, E. R. et al. A gene-based association method for mapping traits using reference transcriptome data. Nat Genet 47, 1091–1098 (2015).

2. Wu, Y. et al. Integrative analysis of omics summary data reveals putative mechanisms underlying complex traits. Nat Commun 9, 918 (2018).
3. Andrés, G. African Swine Fever Virus Gets Undressed: New Insights on the Entry Pathway. Journal of Virology 91, e01906-16 (2017).
4. Kubes, P. & Jenne, C. Immune Responses in the Liver. Annual Review of Immunology 36, 247–277 (2018).
5. Kusters, Y. H. A. M. & Barrett, E. J. Muscle microvasculature's structural and functional specializations facilitate muscle metabolism. American Journal of Physiology-Endocrinology and Metabolism 310, E379–E387 (2016).
6. Yin, L. et al. Estimation of causal effects of genes on complex traits using a Bayesian-network-based framework applied to GWAS data. Nat Mach Intell 6, 1231–1244 (2024).
7. Salguero, F. J. Comparative Pathology and Pathogenesis of African Swine Fever Infection in Swine. Front Vet Sci 7, 282 (2020).
8. Zhu, Z. et al. Single-cell profiling of African swine fever virus disease in the pig spleen reveals viral and host dynamics. Proceedings of the National Academy of Sciences of the United States of America 121, e2312150121 (2024).
9. Cho, K.-H. et al. Pathogenicity and Pathological Characteristics of African Swine Fever Virus Strains from Pig Farms in South Korea from 2022 to January 2023. Pathogens 12, 1158 (2023).
10. Sokol, C. L. & Luster, A. D. The Chemokine System in Innate Immunity. Cold Spring Harb Perspect Biol 7, a016303 (2015).
11. Vilček, J. & Feldmann, M. Historical review: Cytokines as therapeutics and targets of therapeutics. Trends in Pharmacological Sciences 25, 201–209 (2004).
12. Zheng, Y. et al. Transcriptome profiling in swine macrophages infected with African swine fever virus at single-cell resolution. Proc Natl Acad Sci U S A 119, e2201288119 (2022).
13. Fawkner-Corbett, D. et al. Spatiotemporal analysis of human intestinal development at single-cell resolution. Cell 184, 810-826.e23 (2021).
14. Sehl-Ewert, J., Friedrichs, V., Carrau, T., Deutschmann, P. & Blome, S. Pathology of African Swine Fever in Reproductive Organs of Mature Breeding Boars. Viruses 15, 729 (2023).
15. Yamamoto, A., Nagano, T., Takehara, S., Hibi, M. & Aizawa, S. Shisa Promotes Head Formation through the Inhibition of Receptor Protein Maturation for the Caudalizing Factors, Wnt and FGF. Cell 120, 223–235 (2005).
16. Visscher, P. M. et al. 10 Years of GWAS Discovery: Biology, Function, and Translation. The American Journal of Human Genetics 101, 5–22 (2017).
17. Karalyan, Z. et al. Interferon status and white blood cells during infection with African swine fever virus in vivo. Veterinary Immunology and Immunopathology 145, 551–555 (2012).
18. Chen, K., Magri, G., Grasset, E. K. & Cerutti, A. Rethinking mucosal antibody responses: IgM, IgG and IgD join IgA. Nat Rev Immunol 20, 427–441 (2020).
19. Gómez-Villamandos, J. C., Bautista, M. J., Sánchez-Cordón, P. J. & Carrasco, L. Pathology of African swine fever: The role of monocyte-macrophage. Virus Research 173, 140–149 (2013).
20. Ogweng, P. et al. Ancestry and genome-wide association study of domestic pigs that survive African swine fever in Uganda. Trop Anim Health Prod 56, 366 (2024).
21. Qi, F. et al. Genome-wide characterization of structure variations in the Xiang pig for genetic resistance to African swine fever. Virulence 15, 2382762 (2024).

Editorial Manager®

Cookie Preference Center

We use cookies which are necessary to make our site work. We may also use additional cookies to analyze, improve and personalize our content and your digital experience. For more information, see our Cookie Policy.

You may choose not to allow some types of cookies. However, blocking some types may impact your experience of our site and the services we are able to offer. See the different category headings below to find out more or change your settings.

Allow all

Manage Consent Preferences

Strictly Necessary Cookies

Always active

These cookies are necessary for the website to function and cannot be switched off in our systems. They are usually only set in response to actions made by you which amount to a request for services, such as setting your privacy preferences, logging in or filling in forms. You can set your browser to block or alert you about these cookies, but some parts of the site will not then work.

Functional Cookies

These cookies enable the website to provide enhanced functionality and personalisation. They may be set by us or by third party providers whose services we have added to our pages. If you do not allow these cookies then some or all of these services may not function properly.

Performance Cookies

These cookies allow us to count visits and traffic sources so we can measure and improve the performance of our site. They help us to know which pages are the most and least popular and see how visitors move around the site.

Targeting Cookies

These cookies may be set through our site by our advertising partners. They may be used by those companies to build a profile of your interests and show you relevant adverts on other sites. If you do not allow these cookies, you will experience less targeted advertising.

Cookie List

Clear

Apply Cancel
Consent Leg.Interest

Confirm my choices

Power
