# Supplementary material for: Integrated multi-omics profiling identifies genetic loci of African swine fever resistance in pigs
Source: Gigascience. 2026 May 30;15:giag066. doi: 10.1093/gigascience/giag066 (PMC13289747; doi:10.1093/gigascience/giag066)
Supplement: giag066_Reviewer_1_Report_original_submission — Reviewer 1 -- 3/1/2026 [file giag066_reviewer_1_report_original_submission.pdf]

Reviewer Report

Title: Integrated multi-omics profiling identifies genetic loci of African swine fever resistance in pigs

Version: Original Submission **Date:** 3/1/2026

Reviewer name: Reviewer 1

Reviewer Comments to Author:

Revisions and responses requested of the authors:
Line 70-71: It would be good to clarify that the project losses for the US are hypothetical if ASFV ever arrived in the US (which it has not yet). As written, this is not clear.
Line 101: This is the start of a passage that states "to explore this hypothesis" in reference to the previous paragraph, but there is no hypothesis proposed before this statement. Please clarify the text here to clearly state the objectives of the study.
Line 113-116: The text states, "our study bridges variant discovery with functional validation and predictive modeling, offering a more mechanistic understanding of ASF resistance." The authors need to be very careful here as neither functional no mechanistic data is used in this study. Only gene expression and GWAS data are used in the presented analyses. No assays that directly address viral or host immune function or gene function through knockout, enzymatic or true functional assays are used in this study. I understand what the authors are trying to say, but the analyses conducted are not functional or mechanistic, but instead based on functional enrichment or integrative, high-throughput omics analyses which is not the same as the targeted molecular biology assays implied by the text. Please update the language throughout to differentiate between causal statistical inference and candidate causal mutations.
Line 151-172: Regarding the TWAS and SMR analysis, are these two methods truly independent analyses? They both appear to rely on the same pig GTEx gene expression data. Does this mean they destined to generate similar results because similar input information is used in these analyses? Or are they actually using different datasets? Please explain how these analyses compliment each other and provide unique (and not common) information in their results.
Line 152: What does "prioritized genes" mean in this case? It looks like these are the top genes based on FDR significance from the TWAS. How are the genes identified based on muscle and liver gene expression justified in the context of ASFV? What biological roles do these tissues play in ASFV infection or how do they change in ASFV that would provide signal for incidence of ASF? Please update the text accordingly.
Line 164: The text states, "confirming the presence of shared causal variants associated with ASF resistance (Fig. 3d)." I can appreciate that causal statistical modeling is used within the analyses of this manuscript. However, it is very important to note there is not evidence presented to determine that these are causal genes. Causal genes and causal variants can only be identified through specific functional molecular biology assays linking specific gene functions to trait phenotypes (e.g., knockouts, gene editing, enzymatic assays, RNAi, etc. that alter sub processes of trait phenotypes or the trait phenotype itself). No such biological evidence is presented so the identified variants cannot be called

causal variants. What is presented are enrichment or conditioned statistical tests to provide a probability that a gene may be a causal variant (i.e., to identify candidate causal variants). Please update the text where appropriate throughout the manuscript.

Line 175: Please explain in the context of ASFV how muscle, brain, embryo and liver gene expression data is relevant. We know from previous studies that immune tissues are relevant and that CD163 in cell types such as macrophage are important in ASFV, but do we know similar information for the other tissues?

Line 187: Please list the 3 specific validation methods here to help the reader as there is so much information presented, it's hard to keep the different analyses used clear.

Line 198-201: The text states, "Additional, signaling by KIT in disease (FDR) and lymphocyte number (FDR) emerged as key pathways potentially driving immune responses against ASF. These findings reinforce the involvement of specific immune signaling mechanisms in ASF resistance." I'm not sure what you are trying to say in the second sentence here. If the intent is to say this is a new signaling pathway identified, please make this more clear in the text. If the intent is to say this shows that immune functions are enriched and this is an example, then I recommend deleting this sentence as that is obvious from the result.

Line 211-213: This text discusses potential cross-tissue resistance networks. Is this biologically founded? Correlations can be founded across many types of data despite a lack of causal relationships. Are there other examples of this in other biological processes? For instance, are there extracellular signaling molecules identified that would make sense as cross-tissue signaling molecules? Alternatively, maybe this passage can be worded in a slightly different way to highlight this may be worth future investigation. The best place to address this may be at lines 433-435 in the discussion.

Line 249-252: What is the biological significance of these relationships between the viral gene expression and the 4 host transcripts listed? Please provide more explanation here of the context or meaning of the result. Is this meant to indicate these genes may play a role in host response?

Line 258: Which "prioritized genes" are referred to here? There are so many lists of prioritized genes, it is hard to keep track of them all.

Line 254-274: Are these results novel or consistent with past studies? I apologize if I've missed commentary on this aspect of the identified results in the discussion.

Line 296: The pleiotropy analysis is interesting, but how comparable is MPS to ASFV infection? There needs to be some discussion of this somewhere in the manuscript. As written, it's unclear if MPS is a relevant trait (thought it would seem it should be).

Line 315-318: Are these novel findings, or is there past evidence that ASF impacts these traits? (Realizing, you may never know for a trait like meat quality).

Line 330: Comment 1- It's a nice result the APRS differentiates the pigs into groups. I assume this analysis is conducted with the SNP case-control study population. The population used to test the discriminatory power of the APRS needs to be defined here in the text.

Line 330-Comment 2: Doesn't a portion of the data used for this risk score (i.e. the first data filter) come from data from this same dataset used to do the discriminatory analysis using the APRS? So wouldn't we expect this result given where the data came from to create the polygenic risk score? I realize you don't per se have an independent population for cross-validation (or I didn't understand there was an one in the text), but if the same data set used to create a filter to identify the SNPs in the APRS is also used in

the validation of the risk score, then I think we have to take this result with some caution. Please clarify this point and update the manuscript as appropriate.
Line 331-336: I do not find the randomization analysis useful unless it was being used in the same genomic regions to show selected SNPs outperform those simply in LD. A better comparison would be to compare the discriminatory power of a SNP set identified from a genomic selection model for ASFV resilience. Why would the randomization analysis identify a relationship with ASFV prevalence?
Line 338-341: Wasn't the SNP set identified in the APRS developed using datasets from the PigBiobank? Were different studies used for validation or were the same datasets used for validation that were used to prioritize SNPs within the APRS?
Line 345-353: I think this paragraph needs some context. As written, the relevance is not clear. If these phenotypes are of high relevance to ASF based on published literature, then these findings are important. Can you add a little text here to help connect these results to ASF in some way with references that connect these traits to ASF please.
Line 406: Which 3 methods? Define them here in the text to clarify.
Line 474: How were the SNPs selected within genes when there were multiple SNP? This was unclear to me. There is some explanation in the methods, but this seems lost in the results and discussion.
Line 474: If you are using the same population to create the APRS as you test the score in to discriminate samples, can you truly say it is robust? Please clarify.
Line478-486: These traits appear relevant to ASF, but are they? This needs to be better documented with references to tie the relevant pleiotropic traits to ASF to show the power of what is being done.
Line 550: Please provide the breed composition of these pigs.
Line 583: The sequencing technology needs a reference.
Line 634: The software Gallo needs a reference.
Line 1290-1291- Figure 3 legend: What is the difference between score value and total prioritization score?
Line 1333- part f of Figure 4: The cell types (listed as abbreviations in the actual figure) used in this analysis need to be defined in the figure legend.
Line 1348- figure 6 part c: the PP3 and PP4 need to be defined in the figure legend.
Line 1355: The asterisks in the legend are not very visible in the actual figure at line 1403.
Line 1380- Table 1: An explanation of what comparison 1, 2, 3, 4 are, is needed in the footnotes of the table.
Line 1387- Figure 1: Not all details in the figure are clear. For example, in the top red portion, why are the pigs in the experimental data used for analysis shaded in different colors? The authors should make sure the other details within this diagram are well described as well.
Figure 2: I appreciate that the authors have color coordinated between figure 1 and 2, but figure 2 needs to be able to stand alone. For example, in part a, it's not clear what comparisons or analyses the different bars and lines below the bars correspond to. For part b, what is comparison 1, 2, 3, and 4? For part c, there needs to be a list of what these different methods are based on color used in the figure that are displayed.
General comment regarding the figures: Please check that these images are of high enough image quality to meet the standards of Gigascience. When they are enlarged, the text and images begin to

become rasterized.

Methods

Are the methods appropriate to the aims of the study, are they well described, and are necessary controls included? Choose an item.

Conclusions

Are the conclusions adequately supported by the data shown? Choose an item.

Reporting Standards

Does the manuscript adhere to the journal's guidelines on minimum standards of reporting? Choose an item.

Choose an item.

Statistics

Are you able to assess all statistics in the manuscript, including the appropriateness of statistical tests used? Choose an item.

Quality of Written English

Please indicate the quality of language in the manuscript: Choose an item.

Declaration of Competing Interests

Please complete a declaration of competing interests, considering the following questions:

- Have you in the past five years received reimbursements, fees, funding, or salary from an organisation that may in any way gain or lose financially from the publication of this manuscript, either now or in the future?
- Do you hold any stocks or shares in an organisation that may in any way gain or lose financially from the publication of this manuscript, either now or in the future?
- Do you hold or are you currently applying for any patents relating to the content of the manuscript?
- Have you received reimbursements, fees, funding, or salary from an organization that holds or has applied for patents relating to the content of the manuscript?
- Do you have any other financial competing interests?
- Do you have any non-financial competing interests in relation to this paper?

If you can answer no to all of the above, write 'I declare that I have no competing interests' below. If your reply is yes to any, please give details below.

I declare that I have no competing interests

I agree to the open peer review policy of the journal. I understand that my name will be included on my report to the authors and, if the manuscript is accepted for publication, my named report including any attachments I upload will be posted on the website along with the authors' responses. I agree for my report to be made available under an Open Access Creative Commons CC-BY license (http://creativecommons.org/licenses/by/4.0/). I understand that any comments which I do not wish to be included in my named report can be included as confidential comments to the editors, which will not be published.

I agree to the open peer review policy of the journal

To further support our reviewers, we have joined with Publons, where you can gain additional credit to further highlight your hard work (see: https://publons.com/journal/530/gigascience). On publication of this paper, your review will be automatically added to Publons, you can then choose whether or not to claim your Publons credit. I understand this statement.

Yes Choose an item.
