# Supplementary material for: Integrated multi-omics profiling identifies genetic loci of African swine fever resistance in pigs
Source: Gigascience. 2026 May 30;15:giag066. doi: 10.1093/gigascience/giag066 (PMC13289747; doi:10.1093/gigascience/giag066)
Supplement: giag066_Reviewer_2_Report_original_submission — Reviewer 2 -- 3/30/2026 [file giag066_reviewer_2_report_original_submission.pdf]

**Reviewer Report**

**Title: Integrated multi-omics profiling identifies genetic loci of African swine fever resistance in pigs**

**Version: Original Submission** **Date:** 3/30/2026

**Reviewer name: Reviewer 2**

**Reviewer Comments to Author:**

The authors have conducted a comprehensive study integrating whole-genome sequencing, GWAS, selection scans, and multiple functional genomic approaches to investigate host resistance to African Swine Fever. Through this multi-dimensional strategy, they prioritized 135 high-confidence resistance genes, revealed tissue- and cell-type-specific expression patterns, identified key immune pathways and cellular mediators, and developed a polygenic resistance score that discriminates phenotypic outcomes. Overall, this work represents a substantial effort to establish a genomic foundation for precision breeding of ASF-resistant pigs. This study addresses host resistance to ASF, a topic of high significance. The authors have made considerable efforts to expand the scope of the research and enhance its scientific rigor. However, several details require further clarification or revision to strengthen the conclusiveness of the findings:

Major:

1. My major concern regarding this study is about the types of the ASF virus that had induced the immune response in the pig population of focus. The authors failed to detect the DNA fragment of p22 but found the antibody ELISA test gave positive results, and this pattern implies that this is not a typical infection by the wild type virus. The Eurasian domestic pigs (Sus scrofa) usually cannot survive two weeks after the infection of the wild type ASF except for some local/indigenous pigs in Africa, and the observed high fraction of antibody positives in the sampled Chinese indigenous pigs is not likely caused by the wild types. In the antibody test, the positive results indicate the detection of any of the P32, P62, and P72 ASF proteins. The encoding genes of the three proteins were usually not included in typical attenuated vaccine development. For the KP177R of p22 in the antigen DNA test, the situation is a bit different, and the p22 gene was widely used in the development of live attenuated vaccine (p22-knockout). The p22-knockout is usually used to indicate the difference between a wild-type virus (positive) or an attenuated vaccine (negative).

Despite the immune response induced in the survived pigs is less likely caused by the wild-type ASF virus, the observation here did contribute substantial importance to the swine industry. The multi-gene deletion ASF strains have consequential impacts even more significant than the wild-type virus given the challenges in detecting them under long incubation period, mild-to-modest levels of symptom after infection. This constitutes a new challenge due to the "hidden" nature. Actually, this study provides some important clues to understand the genetics behind such low-virulence infection.

I strongly suggest the authors to clarify the pattern of antibody and antigen tests, and revise the manuscript to specify the scope of this study to the immune response associated with putative low-virulence infection induced by the attenuated virus. This is an important topic for this swine industry.

2. The definitions of 'resistance' and 'tolerance' are not clearly distinguished. Based on the current

results, the phenotype described may align more closely with 'tolerance' rather than true resistance (i.e., limiting disease severity rather than pathogen burden). The authors are encouraged to clarify the conceptual framework and explicitly justify the use of 'resistance', or alternatively consider adopting 'tolerance' where appropriate. Expanding this discussion would help avoid potential misinterpretation and better align the study with established terminology in host-pathogen interaction research.
3. The current study does not fully exclude the potential influence of other pathogens or environmental factors, and therefore the identified signals may not be strictly ASF-specific. It would be beneficial for the authors to more explicitly acknowledge this limitation and discuss its potential impact on the interpretation of results. In particular, reframing the findings in the context of general or multi-pathogen immune responses, or revising the title and scope accordingly, would improve conceptual accuracy and strengthen the overall narrative.
4. In the Introduction, additional context on the feasibility and current progress of breeding for host resistance to ASF would help better position this study within the existing literature. For example, a brief overview of known genetic resistance mechanisms, previous GWAS or functional studies, and the practical challenges in defining ASF resistance phenotypes would provide useful background. Expanding this section would enhance the significance and motivation of the study.
5. Additional details on the GWAS model are needed to improve methodological transparency. In particular, the assumed distribution of effect sizes (e.g., normally distributed SNP effects in a mixed linear model) should be explicitly described. Providing this information would enhance reproducibility and allow readers to better assess the robustness of the analysis.
6. In the population structure analysis, while differences between breeds are described, it remains unclear whether population stratification exists within the experimental cohort itself. Since this could influence downstream association analyses, it is recommended to perform and present a PCA specifically on the study population. Including these results would strengthen confidence that population structure has been adequately controlled.
Minor:
7. Line 130 Correct spelling 'fixation'.
8. Line 196 Change 'implicated' to 'implicated in'.
9. Line 205 Clarify that 'enrichment' refers to heritability enrichment (hÂ$^2$ enrichment) to avoid confusion with expression enrichment.
10. Line 214-216 Consider adding a sentence to clarify the rationale for using mouse embryo spatial transcriptomic mapping, e.g., that it serves as a "spatial expression reference framework" rather than a direct disease model.
11. Line 222 Change 'exhibited' to 'exhibiting'.
12. Line 254 Delete the 2nd 'in'.
13. In the discussion, the mention of APRS appropriately emphasizes its role in capturing "polygenic resistance tendencies" rather than implying absolute disease resistance.

**Methods**

Are the methods appropriate to the aims of the study, are they well described, and are necessary controls included? Choose an item.

**Conclusions**

Are the conclusions adequately supported by the data shown? Choose an item.

**Reporting Standards**

Does the manuscript adhere to the journal's guidelines on minimum standards of reporting? Choose an item.

Choose an item.

**Statistics**

Are you able to assess all statistics in the manuscript, including the appropriateness of statistical tests used? Choose an item.

**Quality of Written English**

Please indicate the quality of language in the manuscript: Choose an item.

**Declaration of Competing Interests**

Please complete a declaration of competing interests, considering the following questions:

- Have you in the past five years received reimbursements, fees, funding, or salary from an organisation that may in any way gain or lose financially from the publication of this manuscript, either now or in the future?
- Do you hold any stocks or shares in an organisation that may in any way gain or lose financially from the publication of this manuscript, either now or in the future?
- Do you hold or are you currently applying for any patents relating to the content of the manuscript?
- Have you received reimbursements, fees, funding, or salary from an organization that holds or has applied for patents relating to the content of the manuscript?
- Do you have any other financial competing interests?
- Do you have any non-financial competing interests in relation to this paper?

If you can answer no to all of the above, write 'I declare that I have no competing interests' below. If your reply is yes to any, please give details below.

I declare that I have no competing interests

I agree to the open peer review policy of the journal. I understand that my name will be included on my report to the authors and, if the manuscript is accepted for publication, my named report including any attachments I upload will be posted on the website along with the authors' responses. I agree for my

report to be made available under an Open Access Creative Commons CC-BY license (http://creativecommons.org/licenses/by/4.0/). I understand that any comments which I do not wish to be included in my named report can be included as confidential comments to the editors, which will not be published.

I agree to the open peer review policy of the journal

To further support our reviewers, we have joined with Publons, where you can gain additional credit to further highlight your hard work (see: https://publons.com/journal/530/gigascience). On publication of this paper, your review will be automatically added to Publons, you can then choose whether or not to claim your Publons credit. I understand this statement.

Yes Choose an item.
